# Leveraging the n→π* Interaction in Alkene Isomerization by Selective Energy Transfer Catalysis

**DOI:** 10.1002/anie.202113600

**Published:** 2021-11-26

**Authors:** Tomáš Neveselý, John J. Molloy, Calum McLaughlin, Linda Brüss, Constantin G. Daniliuc, Ryan Gilmour

**Affiliations:** ^1^ Organisch-Chemisches Institut Westfälische Wilhelms-Universität Münster Corrensstrasse 36 48149 Münster Germany; ^2^ Current address: Max Planck Institute of Colloids and Interfaces Am Mühlenberg 1 14476 Potsdam Germany

**Keywords:** alkenes, bioinspired reactions, catalysis, isomerization, stereochemistry

## Abstract

Examples of geometric alkene isomerization in nature are often limited to the net exergonic direction (Δ*G*°<0), with the antipodal net endergonic processes (Δ*G*°>0) comparatively under‐represented. Inspired by the expansiveness of the maleate to fumarate (*Z*→*E*) isomerization in biochemistry, we investigated the inverse *E*→*Z* variant to validate n_O_→π_C=O_* interactions as a driving force for contra‐thermodynamic isomerization. A general protocol involving selective energy transfer catalysis with inexpensive thioxanthone as a sensitizer (*λ*_max_=402 nm) is disclosed. Whilst in the enzymatic process n_O_→π_C=O_* interactions commonly manifest themselves in the substrate, these same interactions are shown to underpin directionality in the antipodal reaction by shortening the product alkene chromophore. The process was validated with diverse fumarate derivatives (>30 examples, up to *Z:E*>99:1), including the first examples of tetrasubstituted alkenes, and the involvement of n_O_→π_C=O_* interactions was confirmed by X‐ray crystallography.

## Graphical abstract

Fumarate to maleate (*E*→*Z*) isomerization was investigated to validate n_O_→ π_C=O_* interactions as a driving force for contra‐thermodynamic isomerization. A general protocol based on selective energy transfer catalysis was developed with inexpensive thioxanthone as a sensitizer for the conversion of diverse fumarate derivatives, including tetrasubstituted alkenes. The involvement of n_O_→π_C=O_* interactions was confirmed by X‐ray crystallography.

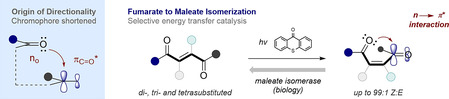

Nature has evolved a powerful inventory of complex isomerases to regulate alkene configuration with iterative spatiotemporal precision.^[1]^ Perfected through evolution, these remarkable ensembles have been integrated into all facets of health and metabolism where alkene stereochemistry manifests itself in function.[^2^, ^3^] An inherent feature of natural isomerases is the ability to impart directionality in a two‐component system where the substrate and product isomers are partitioned by very small energy differences, or the net process is essentially thermoneutral. Despite the importance of alkene stereochemistry in (bio)synthesis, replicating these subtle structural editing events with small molecule catalysts remains a conspicuous challenge.^[4]^

Although much can be gleaned from studying enzymatic isomerization mechanisms,[^2c^, ^5^] most biochemical ensembles are unidirectional and selectively convert one stereoisomer to the other. Since this is often the net *exergonic* process, involving formation of a discrete covalent intermediate, replication in a laboratory paradigm can be achieved. In contrast, biochemical intimations to enable the antipodal (*endergonic*) back reaction are noticeably absent and thus strategies to drive the reaction out of equilibrium are advantageous.[^6^, ^7^] It was envisaged that the noncovalent interactions that manifest themselves in the substrates of naturally occurring isomerases might be leveraged to achieve this goal.^[8]^ Inspired by the venerable maleate (*Z*) to fumarate (*E*) isomerization in biology,[^2a^, ^9^] and mindful of the value of Z‐configured alkene building blocks for contemporary synthesis,^[10]^ efforts to reverse the process using a small molecule catalyst, and delineate the origin of directionality, were undertaken (Figure 1).

**Figure 1:**
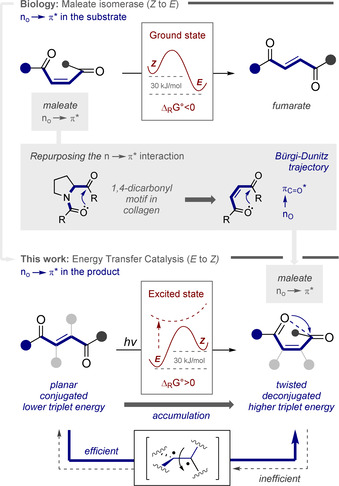
Top: The *Z*→*E* isomerization of maleate to fumarate enabled by maleate isomerase. Centre: The n_O_→π_C=O_* interaction in 1,4‐dicarbonyl derivatives. Bottom: An energy transfer platform for the *E*→Z isomerization where directionality is enabled by the n_O_→π_C=O_* interaction.

Cognizant of the vital structural roles that stabilizing n_O_→π_C=O_* interactions play in complex biomolecules,^[11]^ particularly in a 1,4‐relationship as exemplified in collagen,[^11c^, ^12^] it was envisaged that these effects would satisfy the stereoelectronic requirements for a gating mechanism: this would provide a structural foundation to enable discrimination of fumarate and maleate chromophores in a photosensitization paradigm. Further confidence in this selectivity blueprint stemmed from a report in 1964, in which a single example of ethyl maleate to ethyl fumarate through photosensitization is disclosed.[^13^, ^14^] As a working hypothesis, it was reasoned that the *E→Z* directionality is a consequence of the planar, conjugated fumarate chromophore participating in efficient, selective energy transfer from an excited state photocatalyst.^[15]^ Upon isomerization, the product *Z*‐configured maleate would adopt a twisted conformation, enabling stabilizing n_O_→π_C=O_* interactions consistent with the Bürgi–Dunitz trajectory.^[16]^ This deconjugative shortening of the chromophore would raise the triplet energy,^[17]^ render re‐excitation inefficient, and ultimately lead to an accumulation of the *Z* isomer. Collectively, this repurposing of the n_O_→π_C=O_* interaction associated with 1,4‐dicarbonyl derivatives would provide an *endergonic* antipode to maleate isomerase (Figure 1).^[18]^ To identify a suitable photocatalyst, a process of reaction optimization was conducted with a nonsymmetric derivative of fumaric acid: this would render the two possible n_O_→π_C=O_* interactions non‐equivalent (***E*** **‐1→*Z*‐1**). Reactions were performed in degassed acetonitrile under an argon atmosphere with an irradiation time of 1 h (Table 1).

**Table 1:** Reaction optimization in the isomerization of fumarate scaffold ***E*** **‐1**.^[a]^ 
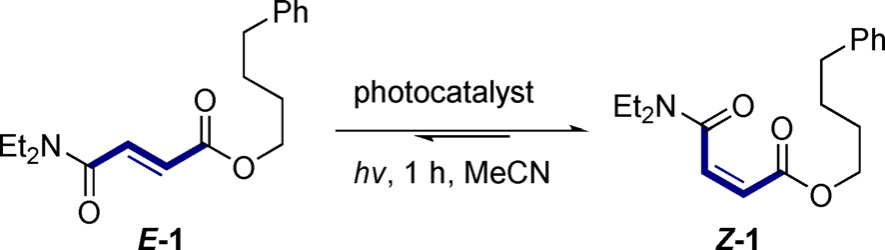

Entry	Catalyst	*E*_T_ [kJ mol^−1^]	λ [nm]	*E:Z*
1	benzophenone	289	365	79:21
2	anthracene	178	365	99:1
3	lumichrome	220	402	99:1
4	benzil	227	450	99:1
**5**	**thioxanthone**	**265**	**402**	**1:99**
6	–	–	402	99:1
7^[b]^	thioxanthone	265	402	67:33
8^[c]^	thioxanthone	265	402	35:65
9^[d]^	thioxanthone	265	402	3:97

[a] Reactions were performed on a 0.1 mmol scale with 5 mol % of the photocatalyst in dry and degassed acetonitrile (3 mL) and were irradiated for 1 h. The ratio of isomers was determined by ^1^H NMR spectroscopy with 1,3,5‐trimethoxybenzene as an internal standard. [b] Irradiated for 1 min. [c] Irradiated for 2 min. [d] Irradiated for 5 min.

Benzophenone was found to promote isomerization, albeit with poor efficiency (*E:Z=*79:21, entry 1). Reactions performed with anthracene (entry 2), lumichrome (entry 3) and benzil (entry 4) did not lead to any detectible formation of the *Z* isomer. Inspection of the triplet energies of these catalysts (178–227 kJ mol^−1^) indicate that a larger value is necessary and therefore thioxanthone (*E*_T_ 265 kJ mol^−1^) was investigated. Gratifyingly, irradiation at 402 nm led to exclusive formation of the desired *Z* isomer (*E:Z*=1:99, entry 5).

A control experiment in the absence of thioxanthone confirmed its catalytic role in the transformation (entry 6). To explore the efficiency of this process, the reactions were repeated at short time intervals of 1, 2 and 5 min (entries 7, 8 and 9, respectively). At the 0.1 mmol scale used for optimization, the reaction was shown to be efficient reaching high levels of selectivity after only 5 min (*E*:*Z=*3:97, entry 9).

A series of fumarate derivatives were exposed to the standard reaction conditions to establish the scope and limitations of the process (Figure 2). Initially, commercially available diethyl fumarate was isomerized to the corresponding diethyl maleate ***Z*** **‐2** (73 %, 94:6). The high levels of selectivity observed with these 1,2‐disubstituted alkenes are noteworthy given the requirement for a third substituent in most isomerization reactions mediated by selective energy transfer catalysis. Whereas leveraging destabilizing A^1, 3^‐strain remains the most common strategy to deconjugating the alkene chromophore and raise the triplet energy,[^3^, ^6^, ^15^] this approach is conceptually distinct in that it harnesses stabilizing carbonyl interactions. Gratifyingly, allyl ethyl fumarate and ethyl (4‐phenylbutyl)fumarate were converted into their *Z* stereoisomers in 89 % (***Z*** **‐3**) and 93 % (***Z*** **‐4**) yield, respectively. To explore the conformational impact of pendant alkyl substituents on isomerization efficiency, the methyl derivative (***Z*** **‐5**) was examined. Under the standard catalysis conditions the product was generated in 73 % yield. Similarly, the 2‐phenylethyl derivative (***Z*** **‐6**) was compatible with the protocol and was formed in 90 % yield. Given the prominence of fumarate‐derived γ‐lactones as neuroprotective agents,^[19]^ ***Z*** **‐7** and ***Z*** **‐8** were explored. Whereas derivative ***Z*** **‐7** was obtained in 75 % yield (photostationary state *Z:E=*82:18), an enhanced yield (91 %) and selectivity (93:7=*Z:E*) was observed for derivative ***Z*** **‐8**. In contrast to many energy transfer processes where tetrasubstituted patterns are not compatible due to chromophore deconjugation in the starting material, alkene ***Z*** **‐9** could be generated in 70 % yield (*Z:E* 83:17). Furthermore, the fluorinated alkene ***E*** **‐10**, and sterically congested ***Z*** **‐11**, could be formed with high levels of selectivity (<5:95 and 94:6, respectively). To the best of our knowledge, this constitutes the first report of a highly selective, photocatalyzed isomerization of tetrasubstituted alkenes.

**Figure 2:**
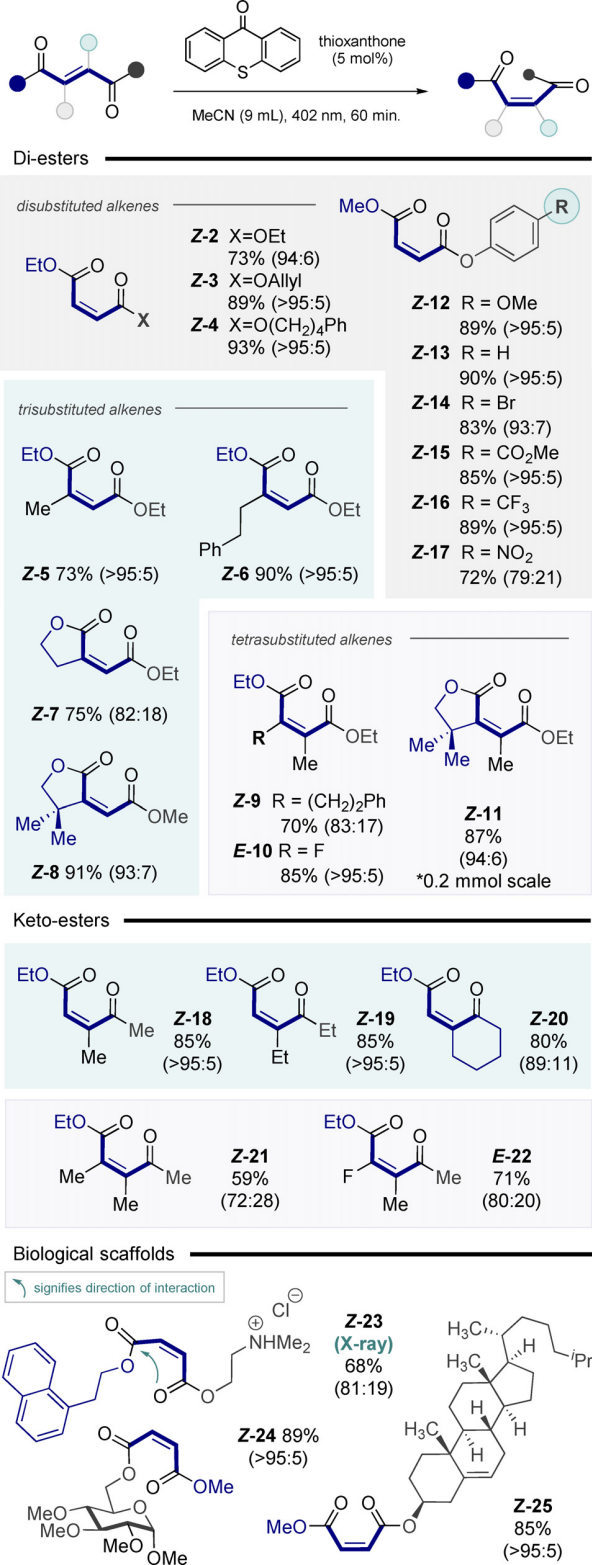
Exploring the substrate scope. Reactions were performed on a 0.3 mmol scale with 5 mol % of thioxanthone in 9 mL of acetonitrile and irradiated for 1 h with a 402 nm LED. The ratio of isomers in the photostationary state was determined by ^1^H NMR spectroscopy. Yields are for the isolated *Z* isomer unless otherwise stated. The *E*‐designation of ***E*** **‐10 and** ***E*** **‐22** reflects the higher priority of F than C.

Cognizant of the electronic impact of substituent effects on n_O_→π_C=O_* interactions in proteins and peptides,^[20]^ a series of 4‐substituted phenol‐derived esters were prepared and subjected to energy transfer catalysis. Derivatives such as OMe (***Z*** **‐12**), H (***Z*** **‐13**), CO_2_Me (***Z*** **‐15**) and CF_3_ (***Z*** **‐16**) all reached photostationary compositions of *Z*:*E* ≥95:5 and were isolated in yields of 89 %, 90 %, 85 % and 89 %, respectively. The 4‐Br derivative (***Z*** **‐14**) was isolated in 83 % yield with a ratio of *Z*:*E=*93:7. The final entry in this short series, ***Z*** **‐17** bearing a nitro group, was isolated in 72 % (*Z*:*E=*79:21). Keto esters proved to be effective substrates for this transformation, enabling derivatives ***Z*** **‐18** and ***Z*** **‐19** to be generated with synthetically useful yields and selectivities (85 %, *Z*:*E*>95:5). Furthermore, the cyclic ketone ***Z*** **‐20** could also be isolated in 80 % yield (*Z*:*E=*89:11). Although more challenging, the tetrasubstituted derivatives ***Z*** **‐21** and ***E*** **‐22** could be accessed through this strategy. The importance of protonated amines in the life sciences prompted us to explore the compatibility of such systems in photosensitized isomerization. To that end, the salt ***Z*** **‐23** was prepared in 68 % yield (*Z:E* 81:19). The carbohydrate and steroid derivatives ***Z*** **‐24** and ***Z*** **‐25** further underscore the compatibility of this method with biologically relevant scaffolds (89 % and 85 %, both *Z:E*>95:5, respectively).

Efforts were then focused on investigating the isomerization behavior of mixed fumarates containing ester and amide *termini* (Figure 3). In general, the efficiency of the process was more pronounced than with the diesters. Substrate ***Z*** **‐1**, which was used for the reaction optimization process (0.1 mmol scale), was obtained in 95 % isolated yield on a 0.3 mmol scale. Similarly, the mixed ester amide ***Z*** **‐26** was obtained in 98 % yield as a single stereoisomer. This reaction was repeated on a 3 mmol scale, allowing 0.94 g (96 %) of material to be generated after 10 h of irradiation. Pendant alkynes and amino alcohols were found to be compatible with the reaction conditions, as is illustrated by compound ***Z*** **‐27** (92 %). Comparable results were noted for derivative ***Z*** **‐28**: gratifyingly, the structure was unequivocally established by single crystal X‐ray diffraction (vide infra). Finally, compounds ***Z*** **‐29** and ***Z*** **‐30**, which contain two fumarate moieties, were generated efficiently (93 % and 96 %, respectively), as was the bisamide ***Z*** **‐31** (96 %, >95:5 *Z:E*).

**Figure 3:**
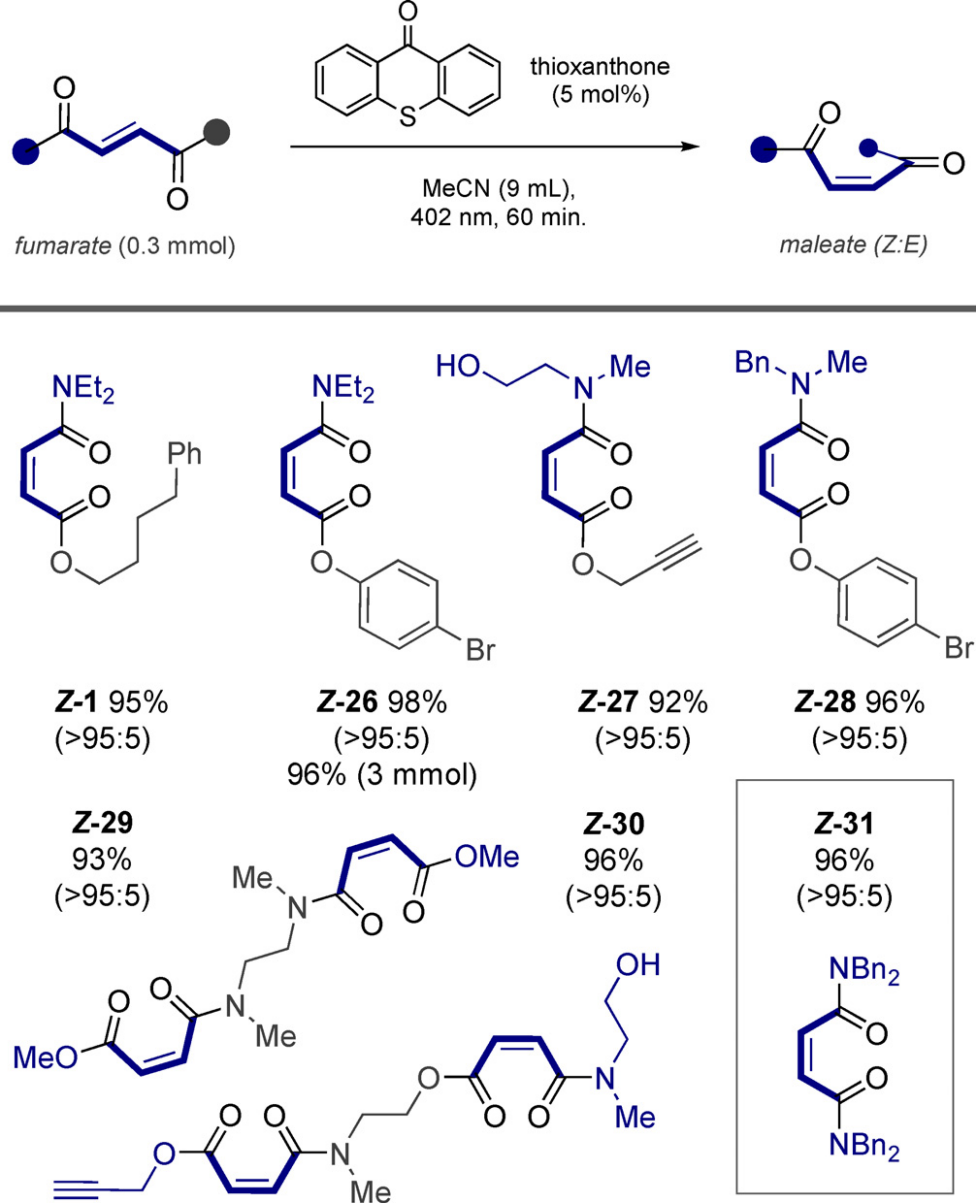
Exploring the scope of ester–amide derivatives. Reactions were performed on a 0.3 mmol scale with 5 mol % of thioxanthone in 9 mL of acetonitrile and irradiated for 1 h with a 402 nm LED. The ratio of isomers in the photostationary state was determined by ^1^H NMR spectroscopy. Yields are for the isolated product.

To establish the importance of carbonyl interactions in deconjugating the chromophore, nitrile control substrates were prepared and subjected to the isomerization protocol (Figure 4 A). Whilst these systems are geometrically predisposed to allow n→π* interactions in the product, the replacement of the C(sp^2^)O acceptor carbon by C(sp)N does not lead to the conformational twist that shortens the chromophore. Consistent with this hypothesis, these materials were found to be susceptible to energy transfer, but photostationary compositions of ca. 1:1 in ***Z*** **‐32** and ***Z*** **‐33** were observed: this underscores the need for a conformational bias to drive the equilibrium to favor the *Z* isomer. Whereas in the fumarate to maleate isomerization the isomers are partitioned by 20 kJ mol^−1^, the triplet energies in the corresponding nitrile systems are identical (251 kJ mol^−1^).^[21]^ This rationalizes the experimental observation that diethyl fumarate undergoes efficient energy transfer from excited state thioxanthone (265 kJ mol^−1^). In contrast, the triplet energy of diethyl malonate lies beyond the capabilities of the photocatalyst, thus rendering the reverse process inefficient.

**Figure 4:**
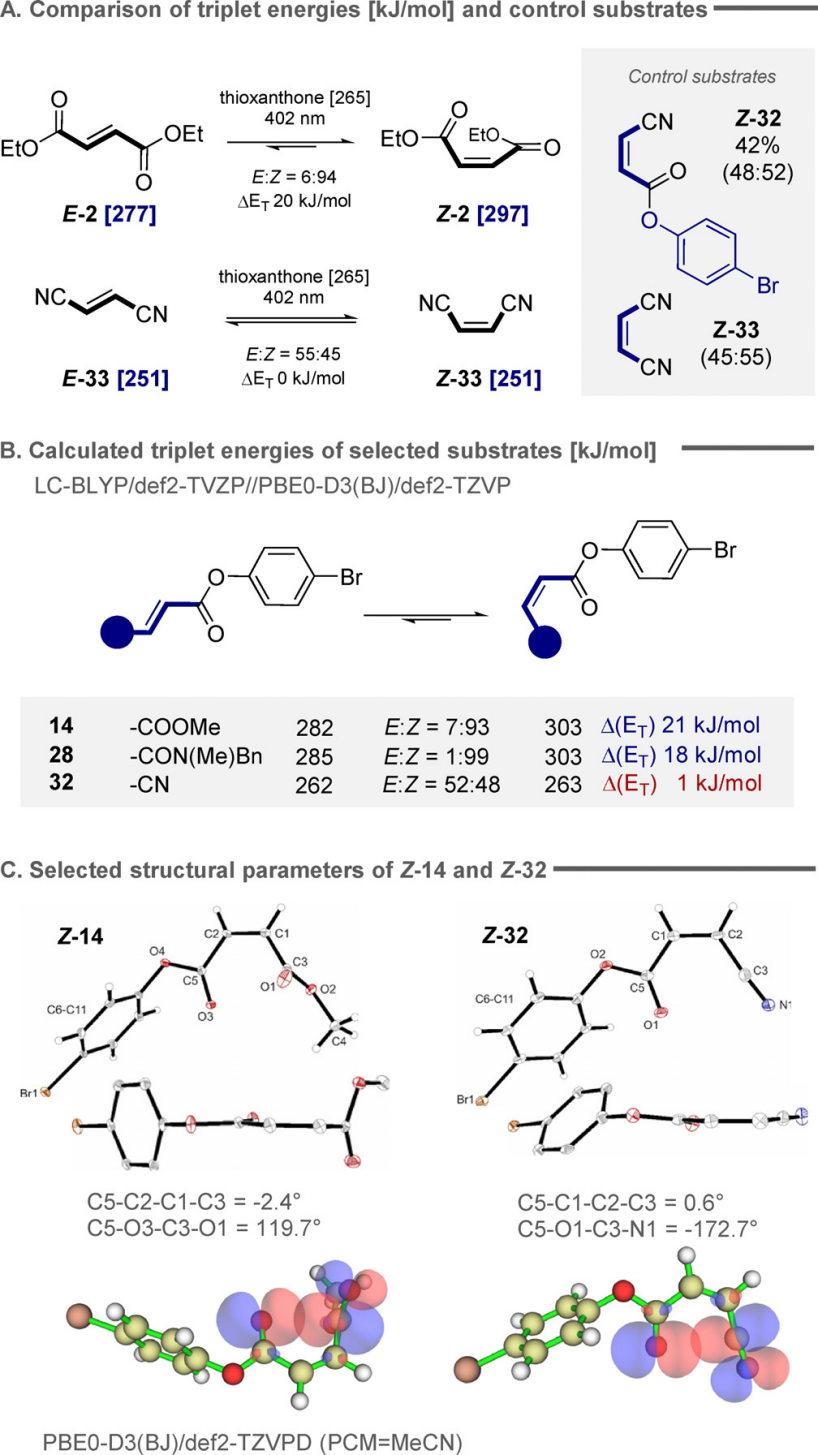
A) Comparison of triplet energy with the isomeric ratio in the photostationary state for dimethyl fumarate and fumaronitrile isomerization. B) Calculated triplet energies for substrates investigated in this study showing the same trend. C) Crystal structures of ***Z*** **‐14** and ***Z*** **‐32** showing key parameters together with depiction of their molecular orbitals obtained by NBO analysis (isovalue=0.035).

A time‐dependent density functional theory (TD‐DFT) approach was used to estimate the triplet energies of the starting *E* isomers of **14**, **28** and **32** (Figure 4 B). Ester ***E*** **‐14** was determined to have triplet energy of 282 kJ mol^−1^ and amide ***E*** **‐28** *E*_T_=285 kJ mol^−1^. These values compare favorably with the experimentally determined energies of dimethyl fumarate.^[21]^ The calculated energy of the respective *Z* isomers was determined to be 303 kJ mol^−1^ for both ***Z*** **‐14** and ***Z*** **‐28**. This is closely similar to the energy gap that partitions dimethyl fumarate and dimethyl maleate. In line with the structural studies in Figure 4 A, the triplet energy difference observed for the *E* and *Z* isomers of **32** was calculated to be 1 kJ mol^−1^ (*Z:E* ratio 48:52). X‐ray crystallographic analyses of ***Z*** **‐14**, ***Z*** **‐32** (Figure 4 C) and ***Z*** **‐23**, ***Z*** **‐28** (Figure 5) clearly reveal the importance of n_O_→π_C=O_* interactions in deconjugating the product (maleate) chromophore,^[22]^ thereby raising the triplet energy: this places directionality on a structural foundation. In the case of ***Z*** **‐14**, the dihedral angle of the carbonyl groups was measured to be 119.7°, whilst in compounds ***Z*** **‐23** and ***Z*** **‐28** it was −109.9° and −156.4°, respectively. In contrast, substitution of the methyl ester by a nitrile moiety led to a planar, conjugated structure with a dihedral angle of 0.6°. The impact on the triplet energy is negligible and so energy transfer is equally efficient with both isomers leading to the experimentally observed statistical mixture of stereoisomers. Finally, to visualize the decisive n_O_→π_C=O_* interactions in this isomerization, natural bond orbital (NBO) analysis was employed. Analyses of structures ***Z*** **‐14** and ***Z*** **‐32** (Figure 4 C) shows a clear overlap between oxygen lone pair and the unoccupied antibonding orbital of the second ester or nitrile moiety, respectively.

**Figure 5:**
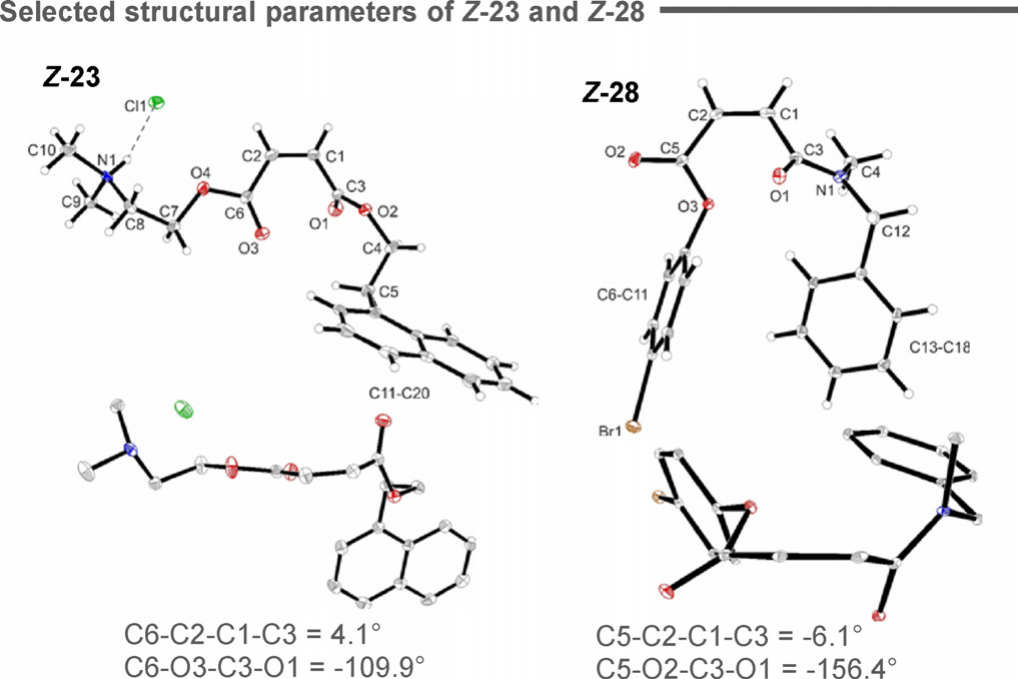
Crystal structures of ***Z*** **‐23** and ***Z*** **‐28** showing key angles.

In conclusion, the net endergonic *E*→*Z* isomerization of fumarates to maleates is reported to complement the *exergonic* antipode that is pervasive in biology. Directionality is determined by stabilizing n_O_→π_C=O_* interactions which shorten the chromophore of the product isomer: this enables selective energy transfer catalysis to be leveraged using inexpensive thioxanthone as the catalyst.

The transformation is general and has enabled the first examples of highly selective tetrasubstituted alkene isomerization. These findings contribute to the growing arsenal of stabilizing interactions that can be leveraged to facilitate contra‐thermodynamic alkene isomerization (Figure 6).^[23]^ Furthermore, the study further validates the importance of n→π* interactions in achieving structural preorganization.

**Figure 6:**
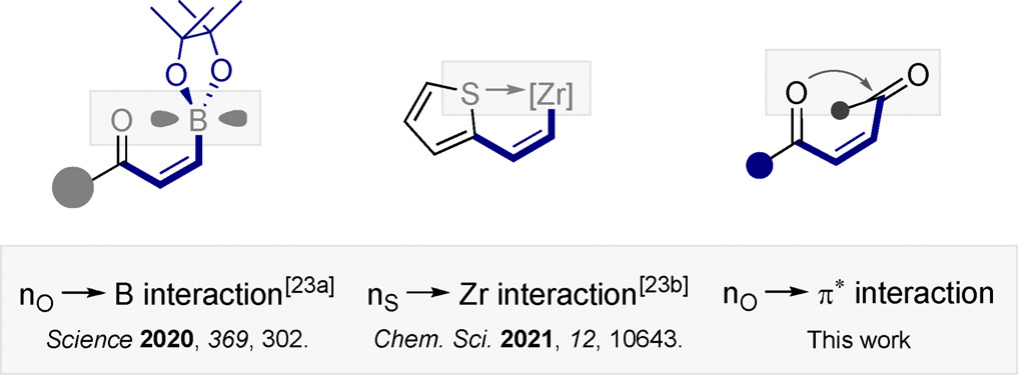
Expanding the arsenal of stabilizing noncovalent interactions to facilitate contra‐thermodynamic alkene isomerization through selective energy transfer catalysis.

## Conflict of interest

The authors declare no conflict of interest.

## Supporting information

As a service to our authors and readers, this journal provides supporting information supplied by the authors. Such materials are peer reviewed and may be re‐organized for online delivery, but are not copy‐edited or typeset. Technical support issues arising from supporting information (other than missing files) should be addressed to the authors.

Supporting Information
