# Supplementary material for: Leveraging the n→π* Interaction in Alkene Isomerization by Selective Energy Transfer Catalysis
Source: Angew Chem Int Ed Engl. 2021 Nov 26;61(2):e202113600. doi: 10.1002/anie.202113600 (PMC9299456; doi:10.1002/anie.202113600)

Angewandte Chemie
Eine Zeitschrift der Gesellschaft Deutscher Chemiker

Supporting Information

Leveraging the n→π* Interaction in Alkene Isomerization by Selective Energy Transfer Catalysis

*Tomáš Neveselý, John J. Molloy, Calum McLaughlin, Linda Brüss, Constantin G. Daniliuc, and Ryan Gilmour**

anie_202113600_sm_miscellaneous_information.pdf

Contents

General Information.......... 2

Experimental section.......... 3

General procedures.......... 3

Analytical information.......... 5

Isomerized compounds.......... 5

Starting materials.......... 20

Crystal structures.......... 37

Computational data.......... 44

References.......... 60

NMR-Spectra of Key Compounds.......... 62

General Information

All chemicals were purchased as reagent grade and used without further purification. Solvents for purification (extraction and chromatography) were purchased as technical grade and distilled on the rotary evaporator prior to use. For column chromatography SiO_2 (40-63 μm for Flash-Chromatography, *VWR Chemicals*) was used as the stationary phase. Analytical thin layer chromatography (TLC) was performed on aluminium foil pre-coated with *SiO_2-60 F_{254}* (*Merck*) and visualized with a UV-lamp (254 nm) or permanganate staining solution. NMR spectra were measured by the NMR service of the Organisch-Chemisches Institut, Westfälische Wilhelms-Universität Münster on a *Bruker AV300*, *Bruker AV400, Agilent DD2 500* or an *Agilent DD2 600* spectrometer at room temperature. The chemical shifts are referenced to the residual solvent peak as internal standard ($CDCl_3$ δ_H = 7.26 ppm, δ_C = 77.16 ppm; DMSO-d_6 δ_H = 2.50 ppm, δ_C = 39.52 ppm; CD_3CN δ_H = 1.94 ppm, δ_C = 118.26 ppm). The resonance multiplicity is abbreviated as: s (singlet), d (doublet), t (triplet), q (quartet), p (pentet), sext (sextet), sep (septet) and m (multiplet). Assignments of unknown compounds are based on ^{1}H, ^{13}C, ^{19}F, COSY, HSQC, HMBC, TOCSY, NOESY and HOESY (^{1}H-^{19}F) spectra. IR spectra were recorded on a *Perkin-Elmer 100 FT-IR* spectrometer, selected adsorption bands are reported in wavenumbers (cm^{-1}) and peaks are reported as: w (weak), m (medium), s (strong) and br (broad). High-resolution mass spectra (HRMS-ESI) were measured by the MS service of the Insitute for Organic Chemsitry, Westfälische Wilhelms-Universität Münster. Melting points were measured on a Büchi B-545 melting-point apparatus in open capillaries and are uncorrected. Photochemical reactions were performed utilizing a set-up of 4 *Winger WEPUV3-S2 UV Power LED Star (Schwarzlicht) 1.2 W* lamps (emission spectrum see Figure **1**). The forward current per chip was set to 700 mA, the resulting forward voltage was 3.4 V while the resulting radiant flux was 1200 mW. The distance between the reaction vessels and the LEDs was set at approximately 0.5 cm for all reactions.

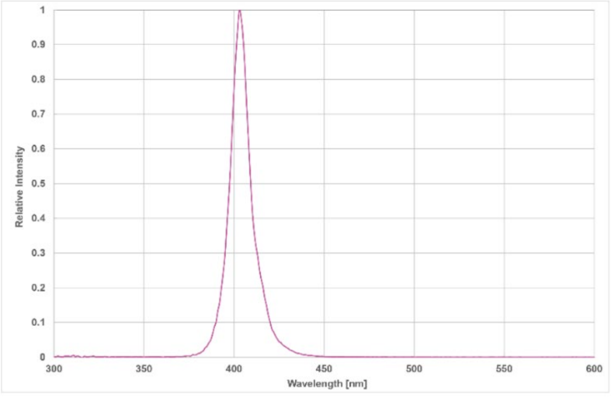

Figure 1 Emission spectrum of Winger WEPUV3-S2 UV Power LED Star (Schwarzlicht) 1.2 W.

Experimental section

General procedure A – Isomerization of fumarate derivatives

To an oven dried round bottom flask, the corresponding fumarate derivative (0.3 mmol, 1 eq.) was placed together with thioxanthone (3.2 mg, 0.015 mmol, 0.05 eq.). The flask was closed with a septum and purged by flow of argon. Dried and degassed acetonitrile (9 mL) was added to the flask and the reaction mixture was placed to an LED setup and stirred for 1-hour under irradiation. Acetonitrile was then removed under reduced pressure. The residue was dissolved in deuterated chloroform and an aliquot was taken for ^{1}H NMR to determine the E : Z ratio. All fractions were then combined and purified using column chromatography on silica.

General procedure B – Isomerization of fumarate derivatives on large scale

To an oven dried round bottom flask, the corresponding fumarate derivative (3 mmol, 1 eq.) was placed together with thioxanthone (32 mg, 0.15 mmol, 0.05 eq.). The flask was closed with a septum and purged by flow of argon. Dried and degassed acetonitrile (90 mL) was added to the flask and the reaction mixture was placed to an LED setup and stirred for 10-hour under irradiation. Acetonitrile was then removed with use of rotary evaporator. The crude material was purified via column chromatography on silica.

General procedure C – Preparation of unsymmetrical derivatives (amide and ester) from fumaryl chloride

To a Schlenk tube in an acetone/dry ice bath under an argon atmosphere, dry and degassed THF (20 mL) was placed followed by fumaryl chloride (0.76 g, 5 mmol, 1 eq.). The corresponding alcohol or phenol (5 mmol, 1 eq.) was dissolved in THF (5 mL) and added dropwise to the stirred solution of fumaryl chloride. The reaction mixture was vigorously stirred for 15 minutes. DIPEA (0.65 g, 5 mmol, 1 eq.) was then added dropwise to the reaction mixture over 20 minutes and the mixture was stirred for an additional 40 minutes. The corresponding amine (5 mmol, 1 eq.) was mixed with DIPEA (0.65 g, 5 mmol, 1 eq.) and added dropwise to the reaction mixture, which was then allowed to warm to room temperature. The reaction mixture was diluted with water (150 mL) and extracted with DCM (3x50 mL). The organic layer was dried with magnesium sulfate and concentrated with use of rotary evaporator. The crude product was purified using column chromatography on silica unless stated otherwise.

General procedure D – Preparation of unsymmetrical derivatives (ester and ester) from mono-methyl fumarate via dicyclohexylcarbodiimide (DCC) coupling

The corresponding alcohol or phenol (1.5 mmol, 1 eq.) and mono-methyl fumarate (195 mg, 1.5 mmol, 1 eq.) were dissolved in DCM (15 mL). To the reaction mixture DMAP (18 mg, 0.15 mmol, 0.1 eq.) followed by DCC (371 mg, 1.8 mmol, 1.2 eq.) were added. The reaction mixture was stirred for 16 hours at room temperature. The reaction mixture was diluted with DCM (20 mL) and washed successively with aqueous sodium hydroxide solution (20 mL, 0.5 M) and water (20 mL). The organic layer was dried with magnesium sulfate and concentrated on a rotary evaporator. The crude product was purified via column chromatography on silica unless stated otherwise.

General procedure E– Preparation of unsymmetrical derivatives (ester and ester) from mono-methyl fumarate via 1-Ethyl-3-(3-dimethylaminopropyl)carbodiimide (EDCl) coupling

The corresponding alcohol (1.5 mmol, 1 eq.) and mono-methyl fumarate (195 mg, 1.5 mmol, 1 eq.) were dissolved in MeCN (10 mL). To the reaction mixture EDCl (345 mg, 1.8 mmol, 1.2 eq.) was added. The reaction mixture was stirred for 3 hours at room temperature. Then it was diluted with DCM (50 mL) and washed successively with an aqueous hydrochloric acid solution (20 mL, 0.5 M) then water (2x20 mL). The organic layer was dried with magnesium sulfate and concentrated on a rotary evaporator. The crude product was purified via column chromatography on silica unless stated otherwise.

General procedure F– Preparation of unsymmetrical derivatives from stabilized phosphoranes and 1,2-dicarbonyl compounds

To an oven dried Schlenk tube under an argon atmosphere the corresponding phosphorane (1 eq.) and dry toluene (1 mL per 1 mmol of phosphorene) were added. To the formed suspension, the corresponding dicarbonyl compound (1 eq., alpha-ketoester or 1,2 dione) was added in one portion unless stated otherwise. The reaction mixture was stirred for 3 hours at 100 °C. Afterwards, the mixture allowed to cool to room temperature and was diluted with Et_2O (3 volumes), which caused triphenylphospine oxide to precipitate out. The precipitate was filtered off,filtride was washed with Et_2O and the filtrate was concentrated with use of rotary evaporator. The crude material was purified via column chromatography on silica.

Analytical information

Isomerized compounds

4-Phenylbutyl (Z)-4-(diethylamino)-4-oxobut-2-enoate (Z-1)

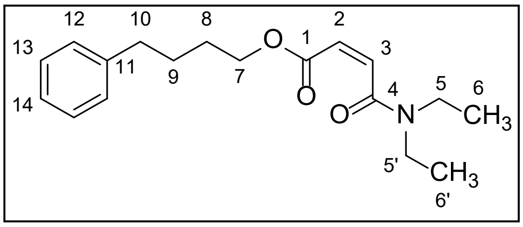

Compound was prepared according to the **General procedure A**. Starting material (**E-1**) (0.3 mmol, 91 mg).

Ratio of isomers in crude mixture determined via ^{1}H NMR - *E:Z* = 1:99. Obtained as a clear oil (86 mg, 95 %).

R*f*= 0.21 (pentane : diethyl ether = 6:4); **^{1}H NMR** (500 MHz, Chloroform-d) δ 7.31 – 7.24 (m, 2H, 13), 7.21 – 7.14 (m, 3H, 12, 14), 6.55 (d, J = 12.0 Hz, 1H, 3), 5.97 (d, J = 12.0 Hz, 1H, 2), 4.15 (ddt, J = 6.5, 4.1, 2.5 Hz, 2H, 7), 3.44 (q, J = 7.2 Hz, 2H, 5), 3.28 (q, J = 7.2 Hz, 2H, 5'), 2.66 – 2.60 (m, 2H, 10), 1.71 – 1.65 (m, 4H, 8, 9), 1.18 (t, J = 7.2 Hz, 3H, 6), 1.11 (t, J = 7.2 Hz, 3H, 6'). **^{13}C NMR** (126 MHz, Chloroform-d) δ 166.3 (4), 164.9 (1), 142.1 (11), 138.3 (3), 128.54 (12), 128.47 (13), 126.0 (14), 123.1 (2), 64.9 (7), 42.5 (5'), 39.0 (5), 35.5 (10), 28.2 (8 or 9), 27.8 (8 or 9), 14.1 (6'), 12.8 (6). **IR (ATR)** ṽ = 2968 (m), 2936 (m), 1721 (s), 1626 (s), 1475 (m), 1143 (s), 1099 (w), 905 (w), 748 (m), 699 (s).

HRMS-ESI (m/z): 326.17213 ([M+Na]$^+$, calcd. for $C_{18}H_{25}NO_3Na^+$: 326.17213);

Dimethyl maleate (Z-2)

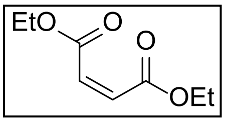

Compound was prepared according to the **General procedure A**. Starting material (diethyl fumarate, E-2) (0.3 mmol, 52 mg).

Ratio of isomers in crude mixture determined via ^{1}H NMR - *E:Z* = 6:94. Obtained as a clear oil (38 mg, 73 %).

R*f*= 0.43 (pentane : diethyl ether = 8:2); **^{1}H NMR** (400 MHz, Chloroform-d) δ 6.22 (s, 2 H), 4.24 (q, J = 7.1 Hz, 4 H), 1.30 (t, J = 7.1 Hz, 6 H). **^{13}C NMR** (100 MHz, Chloroform-d): δ = 165.4, 129.9, 61.4, 14.1.

Analytical data is in agreement with literature.[1]

Allyl ethyl maleate (Z-3)

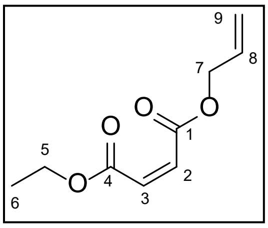

Compound was prepared according to the **General procedure A**. Starting material (**E-3**) (0.3 mmol, 55 mg).

Ratio of isomers in crude mixture determined via ^{1}H NMR - *E:Z* = 2:98. Obtained as a clear oil (49 mg, 89 %).

R*f*= 0.42 (pentane : diethyl ether = 8:2); **^{1}H NMR** (500 MHz, Chloroform-d) δ 6.30 (d, J = 0.7 Hz, 2H), 5.95 (ddt, J = 17.2, 10.4, 5.9 Hz, 1H), 5.36 (dq, J = 17.2, 1.5 Hz, 1H), 5.26 (dq, J = 10.4, 1.2 Hz, 1H), 4.69 (dt, J = 5.9, 1.4 Hz, 2H), 4.24 (q, J = 7.1 Hz, 2H), 1.30 (t, J = 7.1 Hz, 3H). **^{13}C NMR** (126 MHz, Chloroform-d) δ 165.3 (4), 165.0 (1), 131.7 (8), 130.4 (2 or 3), 129.4 (2 or 3), 119.0 (9), 66.0 (7), 61.4 (5), 14.1 (6). **IR (ATR)** ῡ = 2986 (w), 2946 (w), 1722 (s), 1646 (m), 1402 (m), 1206 (s), 1025 (m), 809 (m).

HRMS-ESI (m/z): 207.06246 ([M+Na]$^+$, calcd. for $C_9H_{12}O_4Na^+$: 207.06278);

Ethyl (4-phenylbutyl) maleate (Z-4)

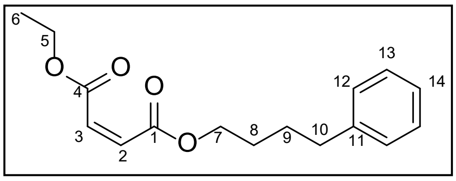

Compound was prepared according to the **General procedure A**. Starting material (**E-4**) (0.3 mmol, 83 mg).

Ratio of isomers in crude mixture determined via ^{1}H NMR - *E:Z* = 2:98. Obtained as a clear oil (77 mg, 93 %).

R*f*= 0.25 (pentane : diethyl ether = 8:2); **^{1}H NMR** (599 MHz, Chloroform-d) δ 7.30 – 7.26 (m, 2H, 13), 7.21 – 7.15 (m, 3H, 12, 14), 6.23 (s, 2H, 2, 3), 4.25 – 4.19 (m, 4H, 5, 7), 2.68 – 2.63 (m, 2H, 10), 1.75 – 1.69 (m, 4H, 8, 9), 1.29 (t, J = 7.1 Hz, 3H, 6). ^{13}C NMR (151 MHz, Chloroform-d) δ 165.5 (1), 165.3 (4), 142.1 (11), 130.0 (2 or 3), 129.9 (2 or 3), 128.52, (12)128.47 (13), 126.0 (14), 65.3 (7), 61.4 (5), 35.5 (10), 28.2 (8 or 9), 27.8 (8 or 9), 14.1 (6).

HRMS-ESI (m/z): 299.12525 ([M+Na]$^+$, calcd. for $C_{16}H_{20}O_4Na^+$: 299.12538);

Diethyl citraconate (Z-5)

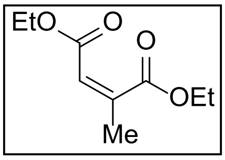

Compound was prepared according to the **General procedure A**. Starting material (diethyl mesaconate) (0.3 mmol, 56 mg).

Ratio of isomers in crude mixture determined via ^{1}H NMR - *E:Z* = 2:98. Obtained as a clear oil (41 mg, 73 %).

R_f= 0.51 (pentane : diethyl ether = 8:2); **^{1}H NMR** (300 MHz, Chloroform-d) δ 5.84 (q, J = 1.7 Hz, 1H), 4.28 (q, J = 7.1 Hz, 2H), 4.17 (q, J = 7.2 Hz, 2H), 2.05 (d, J = 1.7 Hz, 3H), 1.29 (dt, J = 17.1, 7.2 Hz, 6H).

Analytical data is in agreement with literature.[2]

Diethyl 2-phenethylmaleate (Z-6)

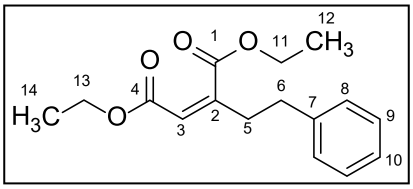

Compound was prepared according to the **General procedure A**. Starting material (**E-6**) (0.3 mmol, 83 mg).

Ratio of isomers in crude mixture determined via ^{1}H NMR - *E:Z* = 2:98. Obtained as a clear oil (75 mg, 90 %).

R_f = 0.66 (pentane : diethyl ether = 8:2); **^{1}H NMR** (599 MHz, Chloroform-d) δ 7.29 (t, J = 7.5 Hz, 2H, 9), 7.23 – 7.16 (m, 3H, 8, 10), 5.79 (d, J = 1.7 Hz, 1H, 3), 4.30 (q, J = 7.1 Hz, 2H, 11), 4.18 (q, J = 7.1 Hz, 2H, 13), 2.82 (dd, J = 9.4, 6.6 Hz, 2H, 6), 2.68 – 2.62 (m, 2H, 5), 1.34 (t, J = 7.1 Hz, 3H, 12), 1.27 (t, J = 7.1 Hz, 3H, 14). **^{13}C NMR** (151 MHz, Chloroform-d) δ 168.8 (1), 165.0 (4), 149.2 (2), 140.3 (7), 128.7 (9), 128.5 (8), 126.5 (10), 120.4 (3), 61.6 (11), 60.9 (13), 36.3 (5), 33.5 (6), 14.24 (14), 14.18(12). **IR (ATR)** ῦ = 2982 (w), 2936 (w), 2906 (w), 2865 (w), 1717 (s), 1647 (m), 1377 (m), 1256 (s), 1103 (m), 1032 (m), 745 (m).

HRMS-ESI (m/z): 299.12548 ($[M+Na]^+$, calcd. for $C_{16}H_{20}O_4Na^+$: 299.12538);

Ethyl (Z)-2-(5,5-dimethyl-2-oxodihydrofuran-3(2H)-ylidene)acetate (Z-7)

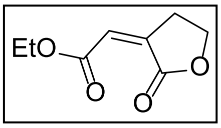

Compound was prepared according to the **General procedure A**. Starting material (**E-7**) (0.3 mmol, 51 mg).

Ratio of isomers in crude mixture determined via ^{1}H NMR - *E:Z* = 18:82. Obtained as a clear oil (18 mg, 75 %).

R_f= 0.21 (pentane : diethyl ether = 3:2, **^{1}H NMR** (599 MHz, Chloroform-d) δ 6.36 (t, J = 2.6 Hz, 1H), 4.40 (td, J = 7.3, 0.6 Hz, 2H), 4.32 (q, J = 7.2 Hz, 2H), 3.03 (tdd, J = 7.5, 2.6, 0.6 Hz, 2H), 1.34 (tt, J = 7.2, 0.6 Hz, 3H). **^{13}C NMR** (151 MHz, Chloroform-d) δ 167.5, 165.3, 131.9, 127.7, 65.5, 61.9, 28.2, 14.1.

HRMS-ESI (m/z): 193.0480 ($[M+Na]^+$, calcd. for $C_8H_{10}O_4Na^+$: 193.0471);

Analytical data is in agreement with literature.[3]

Methyl (Z)-2-(5,5-dimethyl-2-oxodihydrofuran-3(2H)-ylidene)acetate (Z-8)

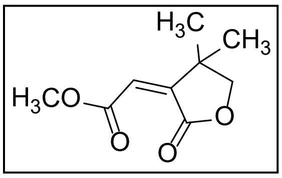

Compound was prepared according to the **General procedure A**. Starting material (**E-8**) (0.3 mmol, 55 mg).

Ratio of isomers in crude mixture determined via ^{1}H NMR - *E:Z* = 7:93. Obtained as a clear oil (50 mg, 91 %).

R*f* = 0.14 (pentane : diethyl ether = 8:2); **^{1}H NMR** (599 MHz, Chloroform-d) δ 6.17 (s, 1H), 4.05 (d, J = 0.5 Hz, 2H), 3.83 (d, J = 0.5 Hz, 3H), 1.29 (d, J = 0.7 Hz, 6H). **^{13}C NMR** (151 MHz, Chloroform-d) δ 167.5, 165.9, 143.3, 125.1, 78.0, 52.6, 40.0, 26.4.

HRMS-ESI (m/z): 207.0643 ($[M+Na]^+$, calcd. for $C_9H_{12}O_4Na^+$: 207.0628);

Analytical data is in agreement with literature.[4]

Diethyl 2-methyl-3-phenethylmaleate (Z-9)

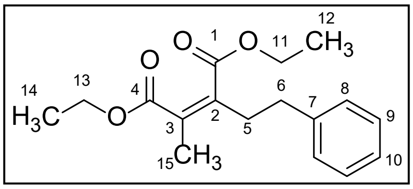

Compound was prepared according to the **General procedure A**. Starting material (**E-9**) (0.3 mmol, 87 mg).

Ratio of isomers in crude mixture determined via ^{1}H NMR - *E:Z* = 17:83. Obtained as a clear oil (61 mg, 70 %).

Rf = 0.47 (pentane : diethyl ether = 8:2); **^{1}H NMR** (400 MHz, Chloroform-d) δ 7.31 – 7.26 (m, 2H, 9), 7.24 – 7.16 (m, 3H, 8, 10), 4.22 (dq, J = 8.8, 7.2 Hz, 4H, 11, 13), 2.77 (dd, J = 9.4, 6.4 Hz, 2H, 6), 2.63 (dd, J = 9.6, 6.3 Hz, 2H, 5), 1.80 (s, 3H, 15), 1.30 (q, J = 7.1 Hz, 6H, 12, 14). **^{13}C NMR** (151 MHz, Chloroform-d) δ 169.0 (1 or 4), 168.8 (1 or 4), 141.0 (7), 137.1 (2), 134.0 (3), 128.64 (8 or 9), 128.60 (8 or 9), 126.4 (10), 61.24 (11 or 13), 61.19 (11 or 13), 34.2 (5), 32.2 (6), 15.3 (15), 14.22 (14), 14.16 (12). **IR (ATR)** ῡ = 2981 (w) 2932 (w), 2869 (w), 1714 (s), 1454 (w), 1256 (s), 1176 (m), 1051 (m), 863 (m), 700 (s).

HRMS-ESI (m/z): 313.14147 ($[M+Na]^+$, calcd. for $C_{17}H_{22}O_4Na^+$: 313.14103);

Diethyl 2-fluoro-3-methylmaleate (E-10)

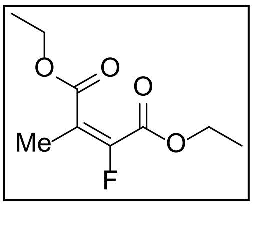

Compound was prepared according to the **General procedure A**. Starting material (**Z-10**) (0.3 mmol, 61 mg).

Ratio of isomers in crude mixture determined via ^{1}H NMR - *E:Z* = 96:4. Obtained as a clear oil (52 mg, 85 %).

R_f = 0.22 (pentane : diethyl ether = 9:1); **^{1}H NMR** (400 MHz, Chloroform-d) δ 4.30 – 4.21 (m, 4H), 2.00 (d, J = 3.8 Hz, 3H), 1.32 (td, J = 7.2, 1.4 Hz, 6H). **^{13}C-{^{19}F} NMR** (151 MHz, Chloroform-d) δ 167.1, 159.8, 147.9, 121.9, 62.2, 61.9, 14.1, 14.0, 13.1. **^{19}F NMR** (376 MHz, Chloroform-d) δ -125.4 (q, J = 3.8 Hz). **IR (ATR)** ῦ = 2986 (w), 2944 (w), 1729 (s), 1675 (m), 1370 (m), 1286 (s), 1017 (m), 774 (m).

Analytical data is in agreement with literature.[5]

Ethyl (Z)-2-(4,4-dimethyl-2-oxodihydrofuran-3(2H)-ylidene)propanoate (Z-11)

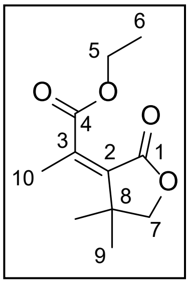

Compound was prepared according to the **General procedure A.** Starting material (**E-11**) (0.2 mmol, 42 mg).

Ratio of isomers in crude mixture determined via ^{1}H NMR - *E*:*Z* = 6:94. Obtained as a clear oil (37 mg, 87 %).

R_f = 0.16 (pentane : diethyl ether = 7:3); **^{1}H NMR** (599 MHz, Chloroform-d) δ 4.29 (q, *J* = 7.1 Hz, 2H, 5), 3.97 (s, 2H, 7), 2.13 (s, 3H, 10), 1.37 (s, 6H, 9), 1.32 (t, *J* = 7.2 Hz, 3H, 6). **^{13}C NMR** (151 MHz, Chloroform-d) δ 170.0 (4), 169.0 (1), 141.1 (3), 132.0 (2), 78.6 (7), 61.8 (5), 39.1 (8), 25.6 (9), 16.5 (10), 14.0 (6). **IR** (ATR) ῦ = 2969 (w), 1752 (s), 1725 (s), 1669 (m), 1465 (w), 1367 (m), 1290 (s), 1254 (s), 1135 (s), 1023 (s), 856 (w), 786 (m), 770 (m), 722 (w), 684 (w).

HRMS-ESI (m/z): 235.0937 ([M+Na]$^+$, calcd. for $C_{11}H_{16}O_4Na^+$: 235.0941).

4-Methoxyphenyl methyl maleate (Z-12)

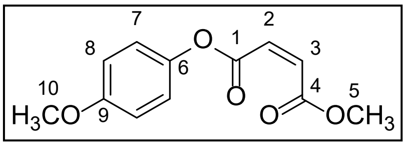

Compound was prepared according to the **General procedure A**. Starting material (**E-12**) (0.3 mmol, 71 mg).

Ratio of isomers in crude mixture determined via ^{1}H NMR - *E:Z* = 4:96. Obtained as a clear oil (63 mg, 89 %).

R_f = 0.31 (pentane : diethyl ether = 8:2); **^{1}H NMR** (500 MHz, Chloroform-d) δ 7.14 – 7.06 (m, 2H, 7), 6.94 – 6.87 (m, 2H, 8), 6.45 (d, J = 11.9 Hz, 1H, 2), 6.38 (d, J = 11.9 Hz, 1H, 3), 3.81 (s, 3H, 5), 3.80 (s, 3H, 10). **^{13}C NMR** (126 MHz, Chloroform-d) δ 165.6 (4), 164.3 (1), 157.7 (9), 143.8 (6), 130.5 (3), 129.8 (2), 122.3 (7), 114.7 (8), 55.7 (10), 52.5 (5). **IR (ATR)** ῦ = 3074 (w), 2956 (w), 1725 (s), 1505 (s), 1389 (m), 1146 (s), 807 (m).

HRMS-ESI (m/z): 259.05716 ([M+Na]$^+$, calcd. for $C_{12}H_{12}O_5Na^+$: 259.05769);

Phenyl methyl maleate (Z-13)

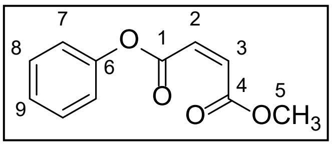

Compound was prepared according to the **General procedure A**. Starting material (**E-13**) (0.3 mmol, 62 mg).

Ratio of isomers in crude mixture determined via ^{1}H NMR - *E:Z* = 4:96. Obtained as a clear oil (56 mg, 90 %).

R*f*= 0.26 (pentane : diethyl ether = 8:2); **^{1}H NMR** (500 MHz, Chloroform-d) δ 7.44 – 7.37 (m, 2H, 7), 7.28 – 7.22 (m, 1H, 9), 7.22 – 7.16 (m, 2H, 8), 6.47 (dd, J = 11.9, 0.2 Hz, 1H, 2), 6.40 (d, J = 11.9 Hz, 1H, 3), 3.82 (t, J = 0.2 Hz, 3H, 5). **^{13}C NMR** (126 MHz, Chloroform-d) δ 165.6 (4), 163.9 (1), 150.4 (6), 130.6 (3), 129.76 (2), 129.67 (7), 126.3 (9), 121.5 (8), 52.5 (5). **IR (ATR)** ῡ = 3072 (w), 2954 (w), 1725 (s), 1646 (w), 1388 (m) 1143 (s), 751 (s).

HRMS-ESI (m/z): 229.04680 ([M+Na]$^+$, calcd. for $C_{11}H_{10}O_4Na^+$: 229.04713);

4-Bromophenyl methyl maleate (Z-14)

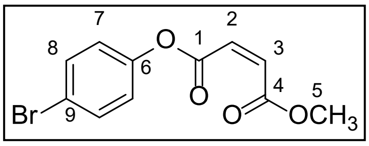

Compound was prepared according to the **General procedure A**. Starting material (**E-14**) (0.3 mmol, 86 mg).

Ratio of isomers in crude mixture determined via ^{1}H NMR - *E:Z* = 7:93. Obtained as a colourless oil (71 mg, 83 %). Upon prolonged standing the material solidified. The material was crystallised using slow diffusion of pentane into the etheric solution to obtain crystals for X-ray analysis.

R*f*= 0.23 (pentane : diethyl ether = 8:2); **^{1}H NMR** (500 MHz, Chloroform-d) δ 7.55 – 7.48 (m, 2H, 7), 7.13 – 7.06 (m, 2H, 8), 6.46 (d, J = 11.9 Hz, 1H, 2), 6.40 (d, J = 11.9 Hz, 1H, 3), 3.81 (s, 3H, 5). **^{13}C NMR** (126 MHz, Chloroform-d) δ 165.4 (4), 163.6 (1), 149.4 (6), 132.7 (7), 130.7 (3), 129.7 (2), 123.4 (7), 119.5 (9), 52.5 (5). **IR (ATR)** ῡ = 3096 (w), 3058 (w), 2951 (w), 1732 (s), 1644 (m), 1481 (m), 1066 (s), 718 (m).

HRMS-ESI (m/z): 306.95745 ([M+Na]$^+$, calcd. for $C_{11}H_9O_4BrNa^+$: 306.95764);

4-(Methoxycarbonyl)phenyl methyl maleate (Z-15)

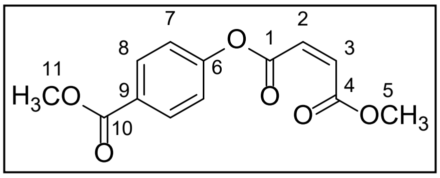

Compound was prepared according to the **General procedure A**. Starting material (**E-15**) (0.3 mmol, 79 mg).

Ratio of isomers in crude mixture determined via ^{1}H NMR - *E:Z* = 4:96. Obtained as a clear oil (67 mg, 85 %).

R*f*= 0.11 (pentane : diethyl ether = 8:2); **^{1}H NMR** (599 MHz, Chloroform-d) δ 8.12 – 8.07 (m, 2H, 8), 7.31 – 7.26 (m, 2H, 7), 6.47 (dd, J = 11.9, 0.8 Hz, 1H, 2), 6.42 (dd, J = 11.9, 0.8 Hz, 1H, 3), 3.92 (d, J = 0.9 Hz, 3H, 11), 3.82 (d, J = 1.0 Hz, 3H, 5). **^{13}C NMR** (151 MHz, Chloroform-d) δ 166.4 (1), 165.4 (4), 163.3 (1), 154.0 (6), 131.4 (8), 130.9 (3), 129.5 (2), 128.3 (9), 121.6 (7), 52.6 (11), 52.4 (5). **IR (ATR)** ῦ = 3069 (w), 3010 (w), 2962 (w), 1736 (s), 1719 (s), 1600 (m), 1436 (m), 1252 (m), 765 (s), 675 (w).

HRMS-ESI (m/z): 287.085219 ($[M+Na]^+$, calcd. for $C_{13}H_{12}O_6Na^+$: 306.9576);

Methyl (4-(trifluoromethyl)phenyl) maleate (Z-16)

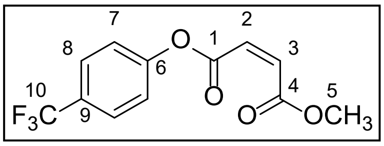

Compound was prepared according to the **General procedure A**. Starting material (**E-16**) (0.3 mmol, 82 mg).

Ratio of isomers in crude mixture determined via ^{1}H NMR - *E:Z* = 4:96. Obtained as a clear oil (73 mg, 89 %).

R*f*= 0.29 (pentane : diethyl ether = 8:2); **^{1}H NMR** (500 MHz, Chloroform-d) δ 7.71 – 7.65 (m, 2H, 8), 7.37 – 7.31 (m, 2H, 7), 6.48 (d, J = 11.8 Hz, 1H, 2), 6.42 (d, J = 11.9 Hz, 1H, 3), 3.82 (s, 3H, 5). **^{13}C-{^{19}F} NMR** (126 MHz, Chloroform-d) δ 165.4 (4), 163.4 (1), 152.8 (6), 130.9 (3), 129.6 (2), 128.7 (9), 127.1 (8), 124.0 (10), 122.1 (7), 52.6 (5). **^{19}F NMR** (376 MHz, Chloroform-d) δ -62.30. **IR (ATR)** ῦ = 2957 (w), 1729 (s), 1612 (m), 1388 (m), 1323 (s), 1062 (s), 949 (m), 626 (m).

HRMS-ESI (m/z): 297.03414 ($[M+Na]^+$, calcd. for $C_{12}H_9O_4F_3Na^+$: 297.03451);

4-Nitrophenyl methyl maleate (Z-17)

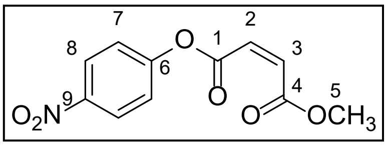

Compound was prepared according to the **General procedure A**. Starting material (**E-17**) (0.3 mmol, 75 mg).

Ratio of isomers in crude mixture determined via ^{1}H NMR - *E:Z* = 21:79. Obtained as slightly yellow needles (54 mg, 72 %).

R_f= 0.14 (pentane : diethyl ether = 8:2); **^{1}H NMR** (500 MHz, Chloroform-d) δ 8.33 – 8.27 (m, 2H, 8), 7.44 – 7.37 (m, 2H, 7), 6.50 (d, J = 11.8 Hz, 1H, 2), 6.44 (d, J = 11.8 Hz, 1H, 3), 3.83 (s, 3H, 5). **^{13}C NMR** (126 MHz, Chloroform-d) δ 165.3 (4), 163.1 (1), 155.0 (6), 145.5 (9), 131.0 (3), 129.5 (2), 125.5 (8), 122.6 (7), 52.6 (5). **IR (ATR)** ῦ =3115 (w), 3078 (w), 2960 (w), 1733 (s), 1591 (m), 1348 (s), 1206 (s), 997 (m), 704 (m).

HRMS-ESI (m/z): 274.03198 ($[M+Na]^+$, calcd. for $C_{11}H_9NO_6Na^+$: 274.03221);

Diethyl 2-methyl-3-phenethylmaleate (Z-18)

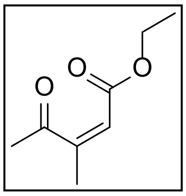

Compound was prepared according to the **General procedure A**. Starting material (**E-18**) (0.3 mmol, 55 mg).

Ratio of isomers in crude mixture determined via ^{1}H NMR - *E:Z* =3:97. Obtained as a clear oil (40 mg, 85 %).

R_f = 0.25 (pentane : diethyl ether = 9:1); **^{1}H NMR** (400 MHz, Chloroform-d) δ 5.68 (q, J = 1.7 Hz, 1H), 4.15 (q, J = 7.1 Hz, 2H), 2.35 (s, 3H), 1.98 (d, J = 1.7 Hz, 3H), 1.26 (t, J = 7.1 Hz, 3H). **^{13}C NMR** (101 MHz, Chloroform-d) δ 206.3, 165.3, 157.3, 117.2, 60.8, 28.7, 20.3, 14.2.

Analytical data is in agreement with literature.[6]

Diethyl 2-methyl-3-phenethylmaleate (Z-19)

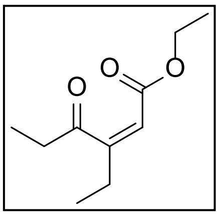

Compound was prepared according to the **General procedure A**. Starting material (**E-19**) (0.3 mmol, 55 mg).

Ratio of isomers in crude mixture determined via ^{1}H NMR - *E:Z* = 1:99. Obtained as a white powder (47 mg, 85 %).

R_f = 0.37 (pentane : diethyl ether = 9:1); **^{1}H NMR** (500 MHz, Chloroform-d) δ 5.65 (t, J = 1.8 Hz, 1H), 4.14 (q, J = 7.1 Hz, 2H), 2.61 (q, J = 7.2 Hz, 2H), 2.30 (qd, J = 7.4, 1.8 Hz, 2H), 1.25 (t, J = 7.1 Hz, 3H), 1.15 (t, J = 7.2 Hz, 3H), 1.11 (t, J = 7.4 Hz, 3H). **^{13}C NMR** (126 MHz, Chloroform-d) δ 209.3, 165.6, 163.4, 115.5, 60.8, 35.2, 27.5, 14.3, 11.4, 7.5. **IR (ATR)** ῦ = 2980 (w), 2941 (w), 2902 (w), 1705 (s), 1639 (m), 1374 (m), 1220 (s), 1140 (s), 962 (w), 872 (m), 729 (w).

Analytical data is in agreement with literature.[7]

Ethyl (Z)-2-(2-oxocyclohexylidene)acetate (Z-20)

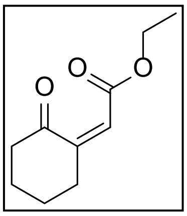

Compound was prepared according to the **General procedure A**. Starting material (**E-20**) (0.3 mmol, 55 mg).

Ratio of isomers in crude mixture determined via ^{1}H NMR - *E:Z* = 11:89. Obtained as a clear oil (44 mg, 80 %).

R$_f$ = 0.37 (pentane : diethyl ether = 9:1); **^{1}H NMR** (500 MHz, Acetonitrile-d3) δ 5.71 (t, J = 1.5 Hz, 1H), 4.08 (q, J = 7.1 Hz, 2H), 2.59 – 2.45 (m, 4H), 1.94 – 1.81 (m, 4H), 1.19 (t, J = 7.1 Hz, 3H). **^{13}C NMR** (126 MHz, Acetonitrile-d3) δ 205.12, 166.53, 156.04, 119.08, 61.31, 44.27, 37.61, 27.51, 27.48 14.20. **IR (ATR)** ṽ = 2983 (w), 2942 (w), 1720 (s), 1702 (s), 1445 (m). 1371 (m), 1176 (s), 944 (m), 823 (w), 714 (w).

Analytical data is in agreement with literature.[8]

Ethyl (Z)-2,3-dimethyl-4-oxopent-2-enoate (Z-21)

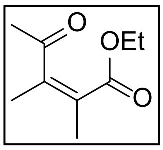

Compound was prepared according to the **General procedure A.** Starting material (**E-21**) (0.3 mmol, 51 mg). Please note: the compound is volatile, handle with care under reduced pressure.

Ratio of isomers in crude mixture determined via ^{1}H NMR - *E*:*Z* = 28:72. Obtained as a clear oil (30 mg, 59 %).

R$_f$ = 0.28 (pentane : diethyl ether = 8:2); **^{1}H NMR** (400 MHz, Chloroform-d) δ 4.17 (q, J = 7.2 Hz, 2H), 2.30 (s, 3H), 1.91 (q, J = 1.2 Hz, 3H), 1.88 (q, J = 1.1 Hz, 3H), 1.27 (t, J = 7.2 Hz, 3H). **^{13}C NMR** (101 MHz, Chloroform-d) δ 206.3, 167.6, 148.4, 125.3, 61.3, 28.7, 16.6, 14.1, 14.1.

Analytical data is in agreement with literature.[9]

Ethyl (E)-2-fluoro-3-methyl-4-oxopent-2-enoate (E-22)

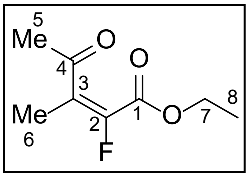

Compound was prepared according to the **General procedure A**. Starting material (**Z-22**) (0.3 mmol, 61 mg).

Ratio of isomers in crude mixture determined via ^{1}H NMR - *E:Z* = 80:20. Obtained as a clear oil (37 mg, 71 %).

R_f= 0.22 (pentane : diethyl ether = 9:1); **^{1}H NMR** (599 MHz, Chloroform-d) δ 4.28 (q, J = 7.2 Hz, 2H, 7), 2.36 (s, 3H, 5), 1.95 (d, J = 3.6 Hz, 3H, 6), 1.32 (t, J = 7.1 Hz, 3H, 8). **^{13}C-{^{19}F} NMR** (151 MHz, Chloroform-d) δ 201.2 (4), 160.2 (1), 143.8 (2), 131.7 (3), 62.3 (7), 29.4 (5), 14.1 (8), 13.2 (6). **^{19}F NMR** (564 MHz, Chloroform-d) δ -130.44 (q, J = 3.8 Hz). **IR (ATR)** ῡ = 2986 (w), 1724 (s), 1709 (s), 1668 (m), 1376 (m), 1306 (s), 1160 (s), 1062 (s), 994 (m), 860(m), 774 (m).

HRMS-ESI (m/z): 197.05844 ($[M+Na]^+$, calcd. for $C_8H_{11}FO_3Na^+$: 197.05844);

2-(dimethylamino)ethyl (2-(naphthalen-1-yl)ethyl) maleate hydrochloride salt (Z-23)

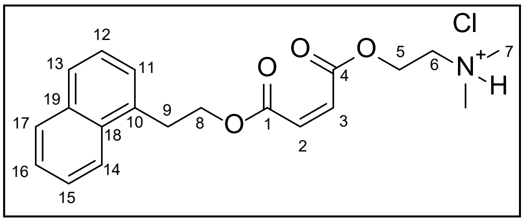

Compound was prepared according to the **General procedure A**. Starting material (**E-23**) (0.3 mmol, 113 mg).

Ratio of isomers in crude mixture determined via ^{1}H NMR - *E:Z* = 19 :81. Obtained as a white powder (77 mg, 68 %). The material was crystalized using slow diffusion of pentane into the etheric solution to obtain crystals for X-ray analysis

Melting point: Compound decomposes above 95 °C. **^{1}H NMR** (599 MHz, Chloroform-d) δ 12.48 (s, 1H, H^+), 8.03 (dd, J = 8.3, 1.2 Hz, 1H, 14), 7.85 (dd, J = 8.1, 1.4 Hz, 1H, 17), 7.75 (d, J = 8.1 Hz, 1H, 13), 7.52 (ddd, J = 8.4, 6.8, 1.4 Hz, 1H, 15), 7.48 (ddd, J = 8.0, 6.8, 1.2 Hz, 1H, 16), 7.40 (dd, J = 8.2, 7.0 Hz, 1H, 12), 7.35 (dd, J = 6.9, 1.3 Hz, 1H, 11), 6.40 (d, J = 11.8 Hz, 1H, 3), 6.20 (d, J = 11.9 Hz, 1H, 2), 4.64 – 4.59 (m, 2H, 5), 4.50 (t, J = 7.2 Hz, 2H, 8), 3.44 (t, J = 7.2 Hz, 2H, 9), 3.31 (q, J = 5.1 Hz, 2H, 6), 2.75 (d, J = 4.9 Hz, 6H, 7). **^{13}C NMR** (151 MHz, Chloroform-d) δ 165.1 (4), 164.9 (1), 133.9 (19), 133.3 (10), 132.0 (18), 131.6 (3), 129.0 (17), 128.5 (2), 127.7 (13), 127.2 (11), 126.4 (15), 125.9 (10), 125.7 (12), 123.4 (14), 65.3 (8), 59.1 (5), 55.6 (6), 43.3 (7), 32.0 (9). **IR (ATR)** ῡ = 3401 (br), 2964 (w), 2584 (w), 1717 (s), 1595 (w), 1464 (w), 1390 (m), 1210 (s), 1160 (s), 987 (m), 777 (s).

HRMS-ESI (m/z): 342.16994 ($[M+Na]^+$, calcd. for $C_{20}H_{24}NO_4^+$: 342.16998);

Methyl (((2R,3R,4S,5R,6S)-3,4,5,6-tetramethoxytetrahydro-2H-pyran-2-yl)methyl) maleate (Z-24)

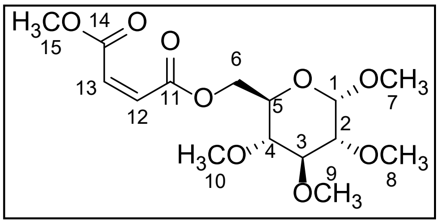

Compound was prepared according to the **General procedure A**. Starting material (**E-24**) (0.3 mmol, 105 mg).

Ratio of isomers in crude mixture determined via ^{1}H NMR - *E:Z* = 3:97. Obtained as a clear viscous oil (93 mg, 89 %).

R*f*= 0.10 (cyclohexane : ethyl acetate = 7:3); **^{1}H NMR** (599 MHz, Chloroform-d) δ 6.27 (s, 2H, 12, 13), 4.79 (d, J = 3.6 Hz, 1H, 1), 4.43 – 4.35 (m, 2H, 6), 3.79 (d, J = 0.5 Hz, 3H, 15), 3.72 (ddd, *J* = 10.1, 4.6, 2.7 Hz, 1H, 5), 3.62 (s, 3H, 9), 3.53 (s, 3H, 10), 3.52 – 3.50 (m, 4H, 3, 8), 3.40 (s, 3H, 7), 3.19 (dd, J = 9.6, 3.6 Hz, 1H, 2), 3.07 (dd, J = 10.1, 8.8 Hz, 1H, 4). **^{13}C NMR** (151 MHz, Chloroform-d) δ 165.7 (14), 165.1 (11), 130.4 (12 or 13), 129.4 (12 or 13), 97.6 (1), 83.6 (3), 81.8 (2), 79.7 (4), 68.6 (5), 64.1 (6), 61.0 (9), 60.7 (10), 59.2 (8), 55.4 (7), 52.4 (15). **IR (ATR)** ῦ = 2935 (w), 2836 (w), 1728 (s), 1645 (w), 1439 (m), 1214 (m), 1156 (s), 1042 (s), 813 (m), 744 (w).

HRMS-ESI (m/z): 371.1327 ($[M_2+Na]^+$, calcd. for $(C_{15}H_{24}O_9)_2Na^+$: 371.1313);

Cholesteryl methyl maleate (Z-25)

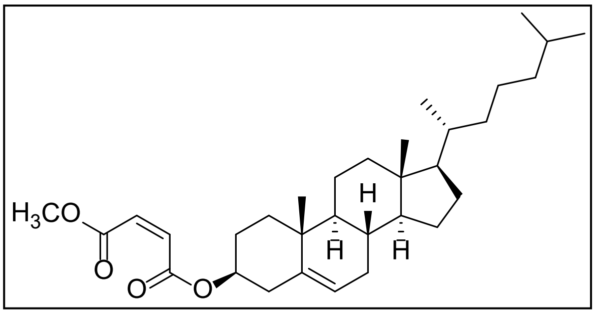

Compound was prepared according to the **General procedure A**. Starting material (**E-25**) (0.3 mmol, 149 mg).

Ratio of isomers in crude mixture determined via ^{1}H NMR - *E:Z* = 3:97. Obtained as a white wax (127 mg, 85 %).

R*f*= 0.21 (pentane : diethyl ether = 8:2); **^{1}H NMR** (500 MHz, Chloroform-d) δ 6.24 (d, J = 12.0 Hz, 1H), 6.21 (d, J = 12.0 Hz, 1H), 5.40 (dd, J = 5.2, 2.3 Hz, 1H), 4.79 – 4.67 (m, 1H), 3.79 (s, 3H), 2.44 – 2.31 (m, 2H), 2.05 – 1.90 (m, 3H), 1.90 – 1.78 (m, 2H), 1.69 – 1.44 (m, 7H), 1.44 – 1.21 (m, 5H), 1.21 – 1.04 (m, 7H), 1.02 (s, 3H), 1.02 – 0.93 (m, 2H), 0.92 (s, 3H), 0.87 (d, J = 2.3 Hz, 3H), 0.86 (d, J = 2.2 Hz, 3H), 0.68 (s, 3H). **^{13}C NMR** (126 MHz, Chloroform-d) δ 165.9, 164.8, 139.5, 130.6, 129.1, 123.1, 75.3, 56.8, 56.3, 52.2, 50.2, 42.5, 39.9, 39.7, 38.0, 37.1, 36.7, 36.3, 35.9, 32.1, 32.0, 28.4, 28.2, 27.7, 24.4, 24.0, 23.0, 22.7, 21.2, 19.5, 18.9, 12.0. **IR (ATR)** ῦ = 2935 (m), 2903 (m), 2861 (m), 2822 (w), 1734 (s), 1713 (s), 1250 (m), 1217 (s), 1000 (m), 834 (m), 686 (w).

HRMS-ESI (m/z): 521.36043 ([M+Na]$^+$, calcd. for $C_{32}H_{50}O_4Na^+$: 521.36031);

4-Bromophenyl (Z)-4-(diethylamino)-4-oxobut-2-enoate (Z-26)

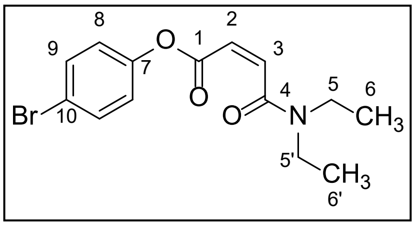

Compound was prepared according to the **General procedure A**. Starting material (**E-26**) (0.3 mmol, 98 mg).

Ratio of isomers in crude mixture determined via ^{1}H NMR - *E:Z* = 1:99. Obtained as a clear oil (96 mg, 98 %).

Large scale experiment was done according to the **General procedure B**. Product was obtained as a clear oil (0.94 g, 96 %).

R*f*= 0.68 (pentane : diethyl ether = 1:1); **^{1}H NMR** (599 MHz, Chloroform-d) δ 7.51 – 7.45 (m, 2H, 8), 7.05 – 6.99 (m, 2H, 9), 6.76 (d, J = 11.9 Hz, 1H, 3), 6.18 (d, J = 11.9 Hz, 1H, 2), 3.45 (q, J = 7.1 Hz, 2H, 5), 3.33 (q, J = 7.2 Hz, 2H, 5’), 1.14 (dt, J = 14.9, 7.2 Hz, 6H, 6, 6’). **^{13}C NMR** (151 MHz, Chloroform-d) δ 165.6 (4), 163.0 (1), 149.4 (7), 140.2 (3), 132.6 (8), 123.4 (9), 122.4 (2), 119.3 (10), 42.6 (5’), 39.2 (5), 14.2 (6’), 12.8 (6). **IR (ATR)** ῦ = 3090 (w), 3072 (w), 2987 (w), 2970 (w), 1745 (s), 1608 (s), 1477 (m), 1164 (s), 1012 (m), 812 (m), 672 (w).

HRMS-ESI (m/z): 348.02053 ([M+Na]$^+$, calcd. for $C_{14}H_{16}NO_3BrNa^+$: 348.02058);

Prop-2-yn-1-yl (Z)-4-((2-hydroxyethyl)(methyl)amino)-4-oxobut-2-enoate (Z-27)

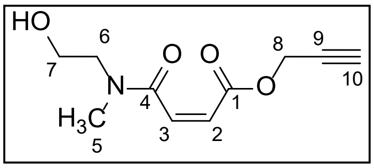

Compound was prepared according to the **General procedure A**. Starting material (**E-27**) (0.3 mmol, 63 mg).

Ratio of isomers in crude mixture determined via ^{1}H NMR - *E:Z* = 1:99. Obtained as a clear oil (58 mg, 92 %).

NMR spectra contain two sets of signals due to the presence of two rotamers in ratio of 72:23.

R*f*= 0.11 (DCM : MeOH = 9:1); Major rotamer: **^{1}H NMR** (500 MHz, Chloroform-d) δ 6.68 (d, J = 11.9 Hz, 1H, 3), 6.09 (d, J = 12.0 Hz, 1H, 2), 4.77 (d, J = 2.4 Hz, 2H, 8), 3.92 (q, J = 5.4 Hz, 2H, 7), 3.64 (t, J = 5.0 Hz, 2H, 6), 3.07 (s, 3H, 5), 3.02 (brs, 1H, -OH), 2.51 (d, J = 2.6 Hz, 1H, 10). **^{13}C NMR** (126 MHz, Chloroform-d) δ 167.6 (4), 164.2 (1), 139.9 (3), 121.6 (2), 77.0 (9), 75.5 (10), 60.2 (7), 52.6 (8), 50.5 (6), 37.2 (5). Minor rotamer: **^{1}H NMR**

(500 MHz, Chloroform-d) δ 6.74 (d, J = 11.9 Hz, 1H), 6.07 (d, J = 12.3 Hz, 1H), 4.77 – 4.72 (m, 2H), 3.75 (q, J = 5.0 Hz, 2H), 3.46 (t, J = 5.3 Hz, 2H), 3.07 (s, 3H), 2.97 (s, 1H), 2.50 (d, J = 3.0 Hz, 1H). **^{13}C NMR** (126 MHz, Chloroform-d) δ 140.2, 122.2, 75.4, 59.3, 52.6, 52.4, 37.1(Quarternary carbons were not observed for minor isomer). **IR (ATR)** ῡ =3270 (br), 2925 (m), 2853 (w), 2127 (w), 1726 (s), 1607 (s), 1389 (m), 1168 (s), 946 (w), 818 (m).

HRMS-ESI (m/z): 234.07325 ($[M+Na]^+$, calcd. for $C_{10}H_{13}NO_4Na^+$: 234.07368);

4-Bromophenyl (Z)-4-(benzyl(methyl)amino)-4-oxobut-2-enoate (Z-28)

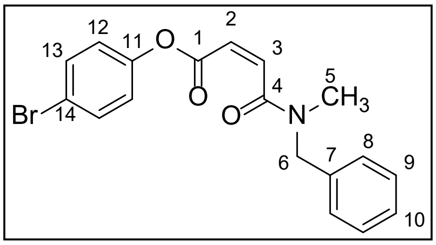

Compound was prepared according to the **General procedure A**. Starting material (**E-28**) (0.3 mmol, 112 mg).

Ratio of isomers in crude mixture determined via ^{1}H NMR - *E:Z* = 1:99. Obtained as a clear oil which spontaneously solidified upon standing (108 mg, 96 %). Material was crystallised using slow diffusion of pentane into the etheric solution to obtain crystals for X-ray analysis.

NMR spectra contain two sets of signals due to the presence of two rotamers in ratio of 55:45.

R_f= 0.11 (pentane : diethyl ether = 4:6); Major rotamer: **^{1}H NMR** (500 MHz, Chloroform-d) δ 7.51 – 7.48 (m, 2H, 12), 7.32 – 7.22 (m, 5H, 8, 9, 10), 7.03 – 6.99 (m, 2H, 13), 6.81 (d, J = 11.9 Hz, 1H, 3), 6.25 (d, J = 12.0 Hz, 1H, 2), 4.66 (s, 2H, 6), 2.92 (s, 3H, 5). **^{13}C NMR** (126 MHz, Chloroform-d) δ 166.5 (4), 163.0 (1), 149.4 (11), 139.8 (3), 136.3 (7), 132.6 (12), 128.7 (8 or 9), 128.5 (8 or 9), 127.7 (10), 123.4 (13), 122.6 (2), 119.3 (14), 50.1 (6), 34.9 (5). Minor rotamer: **^{1}H NMR** (500 MHz, Chloroform-d) δ 7.54 – 7.51 (m, 2H), 7.38 – 7.31 (m, 3H), 7.23 – 7.20 (m, 2H), 7.09 – 7.03 (m, 2H), 6.83 (d, J = 11.9 Hz, 1H), 6.25 (d, J = 11.9 Hz, 1H), 4.54 (s, 2H), 2.96 (s, 3H). **^{13}C NMR** (126 MHz, Chloroform-d) δ 166.7, 162.9, 149.4, 139.8, 136.0, 132.7, 129.1, 128.0, 127.2, 123.4, 123.2, 119.4, 54.2, 32.5. **IR (ATR)** ῡ =

HRMS-ESI (m/z): 396.02150 ($[M+Na]^+$, calcd. for $C_{18}H_{16}NO_3BrNa^+$: 396.02058);

Dimethyl 4,4'-(ethane-1,2-diylbis(methylazanediyl))(2Z,2'Z)-bis(4-oxobut-2-enoate) (Z-29)

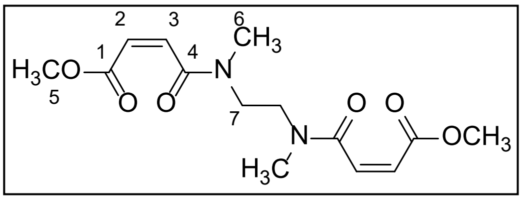

Compound was prepared according to the modified **General procedure A**. Starting material (**E-29**) (0.15 mmol, 94 mg). Starting material was used in 0.15 mmol amount which corresponds to 0.3 mmol of isomerisable double bonds.

Ratio of isomers in crude mixture determined via ^{1}H NMR - *E:Z* = 1:99. Obtained as a clear oil (87 mg, 93 %).

NMR spectra contain three sets of signals due to the presence of three rotamers in ratio of 52:27:21.

R*f* = 0.11 (DCM : MeOH = 9:1); Major rotamer: **^{1}H** NMR (500 MHz, Chloroform-d) δ 6.56 (d, J = 11.9 Hz, 2H, 3), 5.97 (d, J = 11.9 Hz, 2H, 2), 3.69 (s, 6H, 5), 3.68 (s, 4H, 7), 3.00 (s, 6H, 6). **^{13}C NMR** (126 MHz, Chloroform-d) δ 167.4 (4), 165.1 (1), 138.7 (3), 122.5 (2), 51.9 (5), 43.2 (7), 35.7 (6). Minor rotamers (signals combined): **^{1}H NMR** (500 MHz, Chloroform-d) δ 6.65 (d, J = 11.9 Hz, 2H), 6.47 (d, J = 11.9 Hz, 2H), 6.04 (d, J = 11.9 Hz, 2H), 6.00 (d, J = 12.0 Hz, 2H), 3.70 (d, J = 0.5 Hz, 6H), 3.69 (s, 6H), 3.52 (s, 4H), 3.37 (s, 4H), 3.05 (d, J = 0.5 Hz, 6H), 2.92 (s, 6H). **^{13}C NMR** (126 MHz, Chloroform-d) δ 167.44, 167.37, 167.14, 167.07, 165.49, 165.15, 165.12, 165.11, 138.69, 138.16, 137.99, 137.79, 123.78, 123.27, 123.07, 122.53, 52.07, 51.95, 51.95, 51.88, 48.35, 47.20, 45.54, 43.17, 36.59, 35.70, 32.69, 32.59. **IR (ATR)** ῡ =2986 (w), 2954(w), 1722 (s), 1619 (s), 1488 (m), 1410 (m), 1219 (s), 1172 (s), 998 (w), 824 (m).

HRMS-ESI (m/z): 335.12161 ([M+Na]$^+$, calcd. for $C_{14}H_{20}N_2O_4Na^+$: 335.12136);

2-((Z)-N-methyl-4-oxo-4-(prop-2-yn-1-yloxy)but-2-enamido)ethyl (Z)-4-((2-hydroxyethyl)(methyl)amino)-4-oxobut-2-enoate (Z-30)

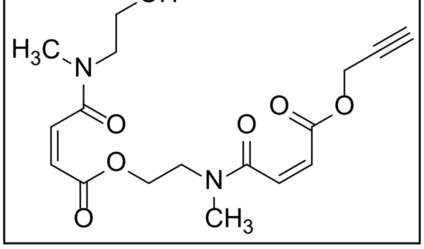

Compound was prepared according to the **General procedure A**. Starting material (**E-30**) (0.15 mmol, 55 mg). Starting material was used in 0.15 mmol amount which corresponds to 0.3 mmol of isomerisable double bonds.

Ratio of isomers in crude mixture determined via ^{1}H NMR - *E:Z* = 1:99. Obtained as a clear oil (53 mg, 96 %).

NMR spectra are complicated due to the presence of four conformers in the solution.

R$_f$= 0.43 (DCM : MeOH = 9:1); **^{1}H NMR** (500 MHz, Chloroform-d) δ 6.74 – 6.58 (m, 2H), 6.09 – 5.99 (m, 2H), 4.73 (t, J = 2.7 Hz, 2H), 4.43 – 4.22 (m, 2H), 3.94 – 3.37 (m, 6H), 3.22 (d, J = 25.0 Hz, 1H), 3.08 – 2.99 (m, 6H), 2.50 (td, J = 2.4, 0.9 Hz, 1H). **^{13}C NMR** (126 MHz, Chloroform-d) δ 167.81, 167.58, 167.27, 167.09, 165.61, 165.08, 164.92, 163.89, 163.75, 163.73, 140.09, 139.54, 139.45, 139.13, 139.04, 138.93, 123.23, 122.81, 122.76, 122.39, 122.33, 122.31, 121.89, 77.30, 75.54, 75.52, 62.23, 62.18, 61.91, 61.52, 60.27, 60.21, 59.38, 59.31, 52.61, 52.55, 52.54, 52.50, 52.48, 50.41, 50.40, 48.96, 48.90, 45.78, 45.72, 37.26, 37.10, 36.68, 36.55, 32.57, 32.29. **IR (ATR)** ῦ = 3432 (br), 3268 (br), 2934 (w), 2124 (w), 1722 (s), 1611 (s), 1437 (m), 1203 (s), 1049 (m), 744 (m), 673 (m).

HRMS-ESI (m/z): 389.13173 ([M+Na]$^+$, calcd. for $C_{17}H_{22}N_2O_7Na^+$: 389.13192);

N1,N1,N4,N4-tetrabenzylmaleamide (Z-31)

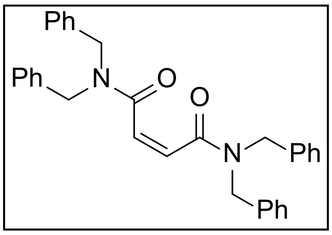

Compound was prepared according to the **General procedure A**. Starting material (**E-31**) (0.3 mmol, 142 mg).

Ratio of isomers in crude mixture determined via ^{1}H NMR - *E:Z* = 96:4. Obtained as a yellowish oil (136 mg, 96 %).

R$_f$= 0.16 (pentane : diethyl ether = 1:1);

^{1}H NMR (599 MHz, Chloroform-d) δ 7.37 – 7.26 (m, 16H), 7.23 – 7.19 (m, 4H), 6.48 (s, 2H), 4.67 (s, 4H), 4.50 (s, 4H). **^{13}C NMR** (151 MHz, Chloroform-d) δ 167.4, 136.8, 136.4, 130.5, 129.0, 128.72, 128.65, 127.8, 127.5, 127.1, 50.7, 47.6. **IR (ATR)** ῦ =3062 (w), 3027 (w), 2924 (w), 1631 (s), 1615 (s), 1451 (s), 1360 (m), 1206 (s), 1028 (m), 954 (m), 726 (s).

HRMS-ESI (m/z): 497.21993 ([M+Na]$^+$, calcd. for $C_{32}H_{30}N_2O_2Na^+$: 497.21995);

Analytical data is in agreement with literature.[10]

4-Bromophenyl (Z)-3-cyanoacrylate (Z-32)

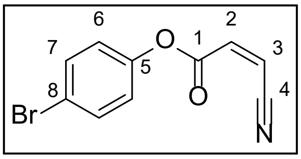

Compound was prepared according to the **General procedure A**. Starting material (**E-32**) (0.3 mmol, 79 mg).

Ratio of isomers in crude mixture determined via ^{1}H NMR - *E:Z* = 52:48. Obtained as a clear viscous oil (31 mg, 42 %). Material was crystallised using slow diffusion of pentane into the etheric solution to obtain crystals for X-ray analysis.

R$_f$= 0.19 (pentane : diethyl ether = 7:3); **^{1}H NMR** (500 MHz, Chloroform-d) δ 7.57 – 7.50 (m, 2H, 6), 7.16 – 7.09 (m, 2H, 7), 6.76 (d, J = 11.5 Hz, 1H, 2), 6.12 (d, J = 11.5 Hz, 1H, 3). **^{13}C NMR** (126 MHz, Chloroform-d) δ 160.5 (1), 149.0 (5), 137.2 (2), 132.8 (6), 123.0 (7), 119.9 (8), 114.0 (4), 112.8 (3). **IR (ATR)** ῦ = 3099 (w), 3065 (w), 2229 (w), 1742 (s), 1622 (m), 1480 (s), 1188 (s), 771 (m).

HRMS-ESI (m/z): 273.94737 ([M+Na]$^+$, calcd. for $C_{10}H_6NO_2BrNa^+$: 273.94741);

Starting materials

4-Phenylbutyl (E)-4-(diethylamino)-4-oxobut-2-enoate (E-1)

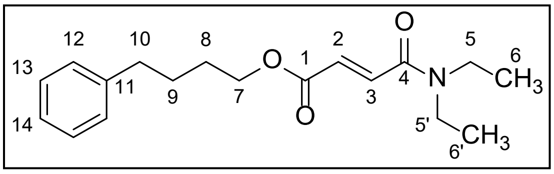

Compound was prepared according to the modified **General procedure C**. 4-Phenyl-1-butanol (5 mmol, 0.75 g) and diethylamine (5 mmol, 0.37 g) were used in this procedure. Product was obtained as thick oil (0.89 g, 59 %).

R$_f$= 0.18 (pentane : diethyl ether = 7:3); **^{1}H NMR** (500 MHz, Chloroform-d) δ 7.34 (d, J = 15.3 Hz, 1H, 3), 7.30 – 7.24 (m, 2H, 13), 7.20 – 7.15 (m, 3H, 14, 12), 6.81 (d, J = 15.2 Hz, 1H, 2), 4.24 – 4.17 (m, 2H, 7), 3.46 (q, J = 7.1 Hz, 2H, 5), 3.41 (q, J = 7.2 Hz, 2H, 5’), 2.69 – 2.61 (m, 2H, 10), 1.76 – 1.66 (m, 4H, 8, 9), 1.21 (t, J = 7.2 Hz, 3H, 6’), 1.17 (t, J = 7.1 Hz, 3H, 6). **^{13}C NMR** (126 MHz, Chloroform-d) δ 166.1 (4), 164.0 (1), 142.0 (11), 134.3 (3), 131.1 (2), 128.49 (13), 128.47 (12), 126.0 (14), 65.1 (7), 42.6 (5’), 41.1 (5), 35.5 (10), 28.2 (8 or 9), 27.8 (8 or 9), 15.1 (6’), 13.1 (6). **IR (ATR)** ῦ = 2980 (w), 2936 (w), 1719 (s), 1622 (s), 1447 (m), 1269 (m), 1136 (m), 1028 (w), 973 (m), 764 (w).

HRMS-ESI (m/z): 326.17213 ([M+Na]$^+$, calcd. for $C_{18}H_{25}NO_3Na^+$: 326.17266);

Allyl ethyl fumarate (E-3)

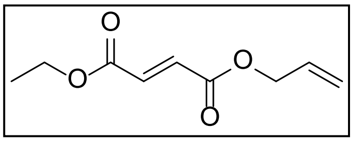

Compound was prepared according to the **General procedure D**. Allyl alcohol (20 mmol, 1.16 g) and mono-ethyl fumarate (10 mmol, 1.44 g) was used. Product was obtained as a clear oil (1.45 g, 79 %).

R$_f$= 0.60 (pentane : diethyl ether = 8:2); **^{1}H NMR** (500 MHz, Chloroform-d) δ 6.87 (s, 2H), 5.94 (ddt, J = 17.2, 10.4, 5.7 Hz, 1H), 5.36 (dq, J = 17.2, 1.5 Hz, 1H), 5.28 (dq, J = 10.4, 1.2 Hz, 1H), 4.70 (dt, J = 5.7, 1.4 Hz, 2H), 4.26 (q, J = 7.1 Hz, 2H), 1.32 (t, J = 7.1 Hz, 3H).

^{13}**C NMR** (126 MHz, Chloroform-d) δ 165.1, 164.8, 134.2, 133.4, 131.7, 119.0, 66.0, 61.5, 14.2. **IR (ATR)** ῦ = 2982 (w), 2943 (w), 1718 (s), 1647 (w), 1296 (s), 1224 (m), 1029 (m), 979 (m), 860 (w), 775 (w).

Analytical data is in agreement with literature.[11]

Ethyl (4-phenylbutyl) fumarate (E-4)

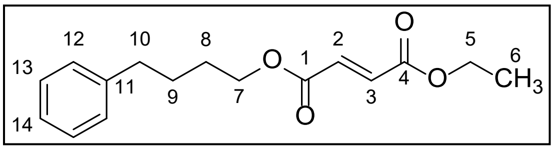

Compound was prepared according to the modified **General procedure C**. 4-Phenyl-1-butanol (5 mmol, 0.75 g) and ethanol (5 mmol, 0.23 g) were used in this procedure. Product was obtained as thick oil (1.28 g, 93 %).

R$_f$= 0.85 (pentane : diethyl ether = 7:3); ^{1}**H NMR** (500 MHz, Chloroform-d) δ 7.29 (tt, J = 6.4, 1.2 Hz, 2H, 13), 7.23 – 7.15 (m, 3H, 12, 14), 6.84 (s, 2H, 2, 3), 4.26 (q, J = 7.1 Hz, 2H, 5), 4.23 – 4.20 (m, 2H, 7), 2.65 (dq, J = 7.1, 3.5 Hz, 2H, 10), 1.72 (p, J = 3.4 Hz, 4H, 8, 9), 1.32 (t, J = 7.1 Hz, 3H, 6). ^{13}**C NMR** (126 MHz, Chloroform-d) δ 165.19 (1), 165.13 (4), 142.0 (11), 133.8 (2 or 3), 133.7 (2 or 3), 128.5 (12, 13), 126.0 (14), 65.3 (7), 61.5 (5), 35.5 (10), 28.2 (8 or 9), 27.8 (8 or 9), 14.3 (6).

HRMS-ESI (m/z): 299.12521 ($[M+Na]^+$, calcd. for $C_{16}H_{20}O_4Na^+$: 299.12538);

Diethyl 2-phenethylfumarate (E-6)

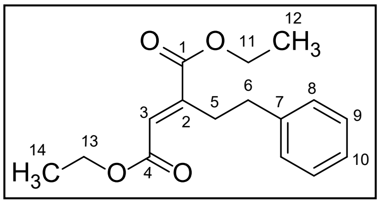

Compound was prepared according to the modified **General procedure F**. Ethyl 2-oxo-4-phenylbutanoate (4 mmol, 0.82 g), (Carbethoxymethylene)triphenylphosphorane (6 mmol, 1.66 g) and toluene (6 mL). After purification, product was obtained as a clear oil (0.67 g, 61 %).

R$_f$= 0.83 (pentane : diethyl ether = 8:2); ^{1}**H NMR** (599 MHz, Chloroform-d) δ 7.29 – 7.17 (m, 5H, 8, 9, 10), 6.78 (s, 1H, 3), 4.22 (dq, J = 21.4, 7.1 Hz, 4H, 11, 13), 3.16 – 3.04 (m, 2H, 6), 2.78 (dd, J = 9.5, 6.6 Hz, 2H, 5), 1.31 (dt, J = 12.5, 7.1 Hz, 6H, 12, 14). ^{13}**C NMR** (151 MHz, Chloroform-d) δ 166.9 (4), 165.7 (1), 147.3 (2), 141.5 (7), 128.8 (9), 128.4 (8), 127.3 (3), 126.1 (10), 61.7 (13), 60.8 (11), 35.6 (5), 30.3 (6), 14.33 (12 or 14), 14.27 (12 or 14). **IR (ATR)** ῦ = 3029 (w), 2982 (w), 2939 (w), 2902 (w), 1715 (s), 1644 (w), 1455 (w), 1203 (s), 1091 (m), 1032 (s), 905 (w), 747 (m).

HRMS-ESI (m/z): 299.12519 ([M+Na]$^+$, calcd. for $C_{16}H_{20}O_4Na^+$: 299.12538);

Ethyl (E)-2-(5,5-dimethyl-2-oxodihydrofuran-3(2H)-ylidene)acetate (E-7)

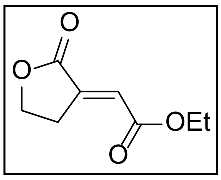

To the solution of alcohol **S-1** (3 mmol, 565 mg) and triethylamine (6.6 mmol, 668 mg) in chloroform (10 mL) phosphorus oxychloride (3.3 mmol, 506 mg) was added dropwise. After addition mixture was stirred for 2 hours at 50 °C. Then, the reaction mixture was cooled to RT and washed with water (20 mL) and evaporated. Crude material was purified with a column chromatography using pentane and diethyl ether (3 : 2). Product was obtained as a clear oil (110 mg, 21 %).

R_f= 0.26 (pentane : diethyl ether = 3:2); **^{1}H NMR** (599 MHz, Chloroform-d) δ 6.78 (t, J = 3.2 Hz, 1H), 4.50 – 4.44 (m, 2H), 4.26 (q, J = 7.1 Hz, 2H), 3.38 (ddd, J = 7.3, 6.9, 3.2 Hz, 2H), 1.33 (t, J = 7.1 Hz, 3H). **^{13}C NMR** (151 MHz, Chloroform-d) δ 170.2, 165.5, 141.2, 124.8, 66.5, 61.4, 27.9, 14.3.

Analytical data is in agreement with literature.[3]

Methyl (E)-2-(4,4-dimethyl-2-oxodihydrofuran-3(2H)-ylidene)acetate (E-8)

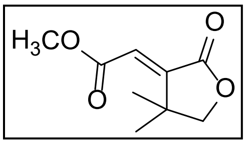

To a suspension of sodium hydride (60 % in mineral oil, 5.5 mmol, 0.22 g) in THF (5 mL) trimethylphosphono acetate (5.5 mmol, 1.00 g) was added dropwise with vigorous stirring. After evolution of hydrogen was ceased, the mixture was stirred for an additional 10 minutes and before (**S-2**) was added in one portion and the reaction was stirred for an additional 4 hours. Afterwards, reaction mixture was diluted with DCM (20 mL) and washed with brined. The organic phase was dried with magnesium sulphate and evaporated. The crude material was purified via chromatography to give a clear oil (264 mg (29 %).

R_f= 0.35 (pentane : diethyl ether = 7:3); **^{1}H NMR** (599 MHz, Chloroform-d) δ 6.80 (s, 1H), 4.06 (s, 2H), 3.81 (s, 3H), 1.46 (s, 6H). **^{13}C NMR** (151 MHz, Chloroform-d) δ 170.9, 165.0, 148.4, 125.6, 79.6, 52.2, 40.0, 24.8.

Analytical data is in agreement with literature.[12]

Diethyl 2-methyl-3-phenethylfumarate (E-9)

Compound was prepared according to the modified **General procedure F**. Ethyl 2-oxo-4-phenylbutanoate (2 mmol, 0.41 g), ((1-Ethoxycarbonylethyliden)-triphenylphosphorane (3 mmol, 1.09 g) and toluene (3 mL). After purification, product was obtained as a clear oil (0.39 g, 66 %).

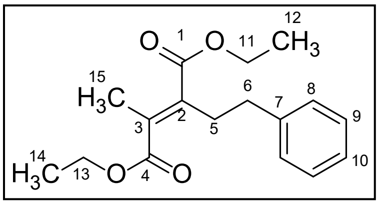

R_f= 0.76 (pentane : diethyl ether = 8:2); **^{1}H NMR** (599 MHz, Chloroform-d) δ 7.31 – 7.23 (m, 2H, 9), 7.23 – 7.15 (m, 3H, 8, 10), 4.23 (dq, J = 12.7, 7.2 Hz, 4H, 11, 13), 2.80 – 2.72 (m, 4H, 5, 6), 2.05 (s, 3H, 15), 1.31 (dt, *J* = 18.5, 7.1 Hz, 6H, 12, 14). **^{13}C NMR** (151 MHz, Chloroform-d) δ 169.0 (4), 168.8 (1), 141.5 (7), 137.3 (2 or 3), 133.9 (2 or 3), 128.6 (9), 128.5 (8), 126.1 (10), 61.1 (11 or 13), 61.0 (11 or 13), 35.3 (5), 33.7 (6), 17.7 (15), 14.36 (12 or 14), 14.34 (12 or 14). **IR (ATR)** ῡ = 3030 (w), 2981 (m), 2936 (w), 1715 (s), 1497 (w), 1366 (w), 1237 (s), 1094 (m), 1018 (m), 860 (w), 771 (w).

HRMS-ESI (m/z): 313.14139 ($[M+Na]^+$, calcd. for $C_{17}H_{22}O_4Na^+$: 313.14103);

Diethyl 2-fluoro-3-methylmaleate (Z-10)

To the solution of lithium perchlorate (3.6 mmol, 0.38 g) in acetonitrile (10 mL) cooled to -10°C in an ice bath and salt, triethyl 2-fluoro-2-phosphonoacetate (3 mmol, 0.73 g) was added followed by diazabicycloundecene (DBU) (3.3 mmol, 0.51 g). The reaction mixture was stirred for 15 minutes and afterwards ethyl pyruvate (3 mmol, 0.35 g) was added in one portion. The reaction was stirred for 2 hours at room temperature, subsequently, it was diluted with diethyl ether (30 mL) and washed with saturated solution of ammonium chloride (40 mL). Organic phase was separated, dried with magnesium sulfate and evaporated under reduced pressure. Crude material was purified via column chromatography on silica. Product was obtained as a clear oil (152 mg 25%).

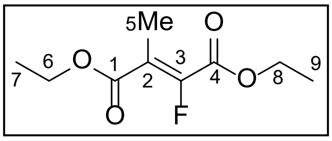

R_f= 0.35 (pentane : diethyl ether = 9:1); **^{1}H NMR** (599 MHz, Chloroform-d) δ 4.32 (q, J = 7.0 Hz, 2H, 8), 4.29 (q, J = 6.9 Hz, 2H, 6), 2.23 (d, J = 3.6 Hz, 3H, 5), 1.35 (t, J = 7.1 Hz, 3H, 7), 1.33 (t, J = 7.1 Hz, 3H, 9). **^{13}C-{^{19}F} NMR** (151 MHz, Chloroform-d) δ 166.3 (1), 160.8 (4), 147.6 (3), 121.8 (2), 62.2 (8), 61.9 (6), 14.23 (9), 14.20 (7), 13.8 (5). **^{19}F NMR** (564 MHz, Chloroform-d) δ -114.53 (q, J = 3.6 Hz). **IR (ATR)** ῡ = 2986 (w), 2944 (w), 1726 (s), 1467 (w), 1371 (m), 1240 (s), 1137 (s), 1059 (s), 860 (m), 772 (m).

HRMS-ESI (m/z): 227.06893 ($[M+Na]^+$, calcd. for $C_9H_{13}FO_4Na^+$: 227.06901);

Ethyl (E)-2-(4,4-dimethyl-2-oxodihydrofuran-3(2H)-ylidene)propanoate (E-11)

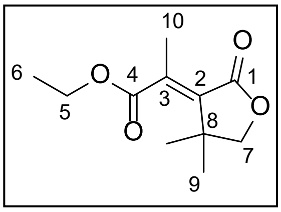

To a solution of lithium perchlorate (6 mmol, 0.64 g) in acetonitrile (14 mL) cooled to -10°C in an ice bath and salt, triethyl 2-phosphonopropionate (5 mmol, 1.1 mL) was added followed by diazabicycloundecene (DBU) (5.5 mmol, 0.84 g). The reaction mixture was stirred for 15 minutes and afterwards **S-2** (5 mmol, 0.64 g) was added in one portion. The reaction was stirred for 2 hours at room temperature, subsequently, it was diluted with diethyl ether (30 mL) and washed with saturated solution of ammonium chloride (30 mL). Organic phase was separated, dried with magnesium sulfate and evaporated under reduced pressure. Crude material was purified via column chromatography on silica. Product was obtained as a clear oil (55 mg 5%).

R$_f$ = 0.53 (pentane : diethyl ether = 7:3); **^{1}H NMR** (599 MHz, Chloroform-d) δ 4.28 (q, J = 7.2 Hz, 2H, 5), 3.90 (s, 2H, 7), 2.37 (s, 3H, 10), 1.34 (t, J = 7.2 Hz, 3H, 6), 1.26 (s, 6H, 9). **^{13}C NMR** (151 MHz, Chloroform-d) δ 170.7 (1), 168.9 (4), 141.0 (3), 133.2 (2), 77.9 (7), 61.6 (5), 40.9 (8), 24.9 (9), 16.6 (10), 14.2 (6). **IR** (ATR) ṽ = 2964 (w), 1759 (s), 1725 (s), 1661 (w), 1463 (w), 1368 (w), 1298 (m), 1247 (s), 1155 (s), 1027 (m), 782 (w).

HRMS-ESI (m/z): 235.0939 ([M+Na]$^+$, calcd. for $C_{11}H_{16}O_4Na^+$: 235.0941).

4-Methoxyphenyl methyl fumarate (E-12)

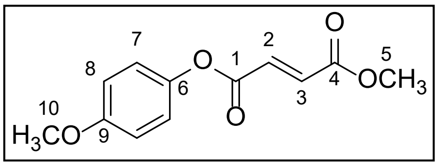

Compound was prepared according to the **General procedure D**. 4-Methoxyphenol (1.5 mmol, 186 mg) was used. The product was obtained as a slightly yellow solid material (174 mg, 49 %).

R$_f$= 0.31 (pentane : diethyl ether = 8:2); **Melting point:** 120-122°C **^{1}H NMR** (500 MHz, Chloroform-d) δ 7.08 – 7.04 (m, 2H, 7), 7.04 (d, J = 1.7 Hz 2H, 2, 3)*, 6.94 – 6.87 (m, 2H, 8), 3.85 (s, 3H, 5), 3.81 (s, 3H, 10). **^{13}C NMR** (126 MHz, Chloroform-d) δ 165.4 (4), 163.9 (1), 157.7 (9), 143.9 (6), 134.8 (2 or 3), 133.3 (2 or 3), 122.2 (7), 114.7 (8), 55.8 (10), 52.6 (5). **IR (ATR)** ṽ = 3070 (w), 3013 (w), 2965 (w), 2845 (w), 1742 (s), 1719 (s). 1508 (s), 1220 (m), 1029 (m), 920 (m), 797 (w).

*Strong roof effect. Interaction constant of alkene coupling is 15.8 Hz.

HRMS-ESI (m/z): 259.0588 ([M+Na]$^+$, calcd. for $C_{12}H_{12}O_5Na^+$: 259.0577);

Phenyl methyl fumarate (E-13)

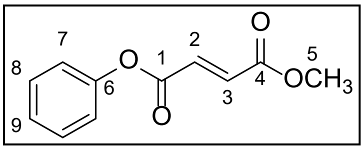

Compound was prepared according to the **General procedure D**. Phenol (1.5 mmol, 141 mg) was used. The product was obtained as a white crystalline powder (104 mg, 50 %).

R$_f$= 0.51 (pentane : diethyl ether = 8:2); **Melting point:** 56-58 °C. **^{1}H NMR** (599 MHz, Chloroform-d) δ 7.45 – 7.37 (m, 2H, 8), 7.29 – 7.24 (m, 1H, 9), 7.17 – 7.12 (m, 2H, 7), 7.07 (d, J = 15.8 Hz, 1H, 2 or 3)*, 7.04 (d, J = 15.7 Hz, 1H, 2 or 3)*, 3.85 (s, 3H, 5). **^{13}C NMR** (151 MHz, Chloroform-d) δ 165.3 (4), 163.5 (1), 150.4 (6), 134.9 (2 or 3), 133.2 (2 or 3), 129.7 (8), 126.4 (9), 121.4 (7), 52.6 (5). **IR (ATR)** ῡ = 3073 (w), 3059 (w), 3008 (w), 2956 (w), 1742 (m), 1717 (s), 1497 (w), 1297 (s), 1161 (s), 982 (s), 765 (m).

*Strong roof effect.

HRMS-ESI (m/z): 229.2485 ([M+Na]$^+$, calcd. for $C_{11}H_{10}O_4Na^+$: 229.0471);

4-Bromophenyl methyl fumarate (E-14)

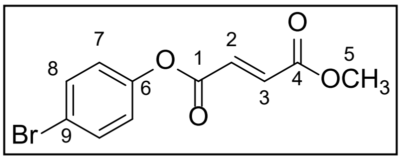

Compound was prepared according to the **General procedure D**. 4-Bromophenol (1.5 mmol, 260 mg) was used. The product was obtained as a white crystalline solid (279 mg, 68 %).

R$_f$= 0.48 (pentane : diethyl ether = 8:2); **Melting point:** 71-72°C **^{1}H NMR** (500 MHz, Chloroform-d) δ 7.56 – 7.49 (m, 2H, 8), 7.05 (d, J = 9.2 Hz, 4H, 2, 3, 7), 3.85 (s, 3H, 5). **^{13}C NMR** (126 MHz, Chloroform-d) δ 165.2 (4), 163.2 (1), 149.4 (6), 135.4 (2 or 3), 132.78 (2 or 3), 132.76 (8), 123.2 (7), 119.5 (9), 52.7 (5). **IR (ATR)** ῡ = 3073 (w), 2959 (w), 1721 (s), 1642 (m), 1486 (m), 131 (s), 1094 (m), 911 (m), 703 (m).

HRMS-ESI (m/z): 306.9576 ([M+Na]$^+$, calcd. for $C_{11}H_9O_4BrNa^+$: 306.9576);

4-(Methoxycarbonyl)phenyl methyl fumarate (E-15)

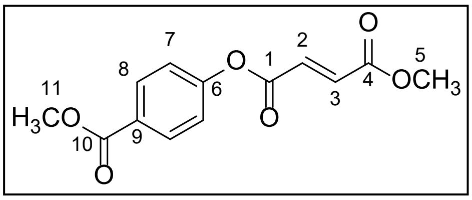

Compound was prepared according to the **General procedure D**. 4-(Methoxycarbonyl)phenol (1.5 mmol, 209 mg) was used. The product was obtained as a white wax (234 mg, 59 %).

R$_f$= 0.26 (pentane : diethyl ether = 6:4); ^{1}**H NMR** (599 MHz, Chloroform-d) δ 8.13 – 8.08 (m, 2H, 8), 7.26 – 7.22 (m, 2H, 7), 7.06 (d, J = 0.8 Hz, 2H, 2, 3), 3.93 (s, 3H, 11), 3.86 (s, 3H, 5). ^{13}**C NMR** (151 MHz, Chloroform-d) δ 166.3 (10), 165.1 (4), 162.9 (1), 153.9 (6), 135.5 (2 or 3), 132.7 (2 or 3), 131.4 (8), 128.3 (9), 121.5 (7), 52.69 (5), 52.4 (11). **IR (ATR)** ῡ = 2965 (w), 1721 (s), 1603 (w), 1438 (m), 1312 (m), 1276 (s), 1106 (m), 944 (m), 701 (m).

HRMS-ESI (m/z): 287.0534 ([M+Na]$^+$, calcd. for $C_{13}H_{12}O_6Na^+$: 287.0526);

Methyl (4-(trifluoromethyl)phenyl) fumarate (E-16)

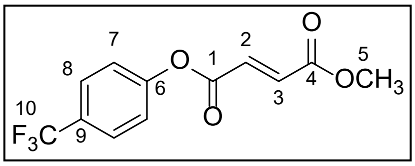

Compound was prepared according to the **General procedure D**. 4- (Trifluoromethyl)phenol (1.5 mmol, 243 mg) was used. Product was obtained as a white solid material (279 mg, 68 %).

R$_f$= 0.54 (pentane : diethyl ether = 8:2); ^{1}**H NMR** (500 MHz, Chloroform-d) δ 7.71 – 7.66 (m, 2H), 7.31 – 7.27 (m, 2H), 7.06 (s, 2H), 3.86 (s, 3H). ^{13}**C-{**^{19}**F} NMR** (126 MHz, Chloroform-d) δ 165.08, 162.95, 152.85, 135.66, 132.54, 128.74, 127.08, 123.90, 121.99, 52.69. ^{19}**F NMR** (470 MHz, Chloroform-d) δ -62.35. **IR (ATR)** ῡ = 3074 (w), 1738 (s), 1724 (s), 1514 (w), 1280 (m), 1179 (m), 989 (s), 912 (m), 684 (m).

Methyl (4-nitrophenyl) fumarate (E-17)

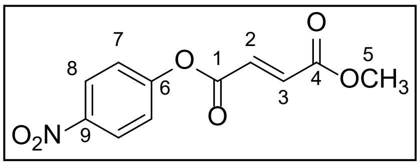

Compound was prepared according to the **General procedure D**. 4-Nitrophenol (1.5 mmol, 209 mg) was used. Product was obtained as a white crystalline solid (204 mg, 54 %).

R$_f$= 0.29 (pentane : diethyl ether = 8:2); ^{1}**H NMR** (599 MHz, Chloroform-d) δ 8.47 – 8.19 (m, 2H, 8), 7.41 – 7.31 (m, 2H, 7), 7.08 (d, J = 15.8 Hz, 1H, 2 or 3)*, 7.05 (d, J = 15.9 Hz, 1H, 2 or 3)*, 3.87 (s, 3H, 5). ^{13}**C NMR** (151 MHz, Chloroform-d) δ 164.9 (4), 162.5 (1), 155.0 (6), 145.8 (9), 136.2 (2 or 3), 132.1 (2 or 3), 125.5 (8), 122.4 (7), 52.8 (5). **IR (ATR)** ῡ = 3116 (w), 3080 (w), 2965 (w), 1748 (m), 1713 (s), 1594 (m), 1493 (s), 1312 (m), 1212 (m), 934 (m), 819 (m).

HRMS-ESI (m/z): 274.0325 ([M+Na]$^+$, calcd. for $C_{11}H_9NO_6Na^+$: 274.0322);

Ethyl (E)-3-methyl-4-oxohex-2-enoate (E-18)

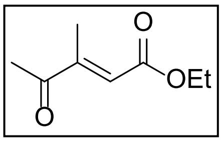

Compound was prepared according to the modified **General procedure F**. Butane-2,3-dione (3 mmol, 0.26 g), (Carbethoxymethylene)triphenylphosphorane (3 mmol, 1.05 g) and toluene (3 mL). After purification, the product was obtained as a clear oil (0.24 g, 51 %).

R*f*= 0.36 (pentane : diethyl ether = 9:1); **^{1}H NMR** (500 MHz, Chloroform-d) δ 6.57 (q, J = 1.5 Hz, 1H), 4.25 (q, J = 7.1 Hz, 2H), 2.38 (s, 3H), 2.21 (d, J = 1.5 Hz, 3H), 1.32 (t, J = 7.1 Hz, 3H). **^{13}C NMR** (126 MHz, Chloroform-d) δ 200.0, 166.3, 150.6, 126.7, 60.9, 26.3, 14.3, 13.2.

Analytical data is in agreement with literature.[13]

Ethyl (E)-3-ethyl-4-oxohex-2-enoate (E-19)

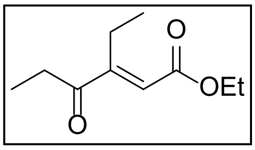

Compound was prepared according to the modified **General procedure F**. Hexane-3,4-dione (2 mmol, 0.23 g), (Carbethoxymethylene)triphenylphosphorane (2 mmol, 0.70 g) and toluene (2 mL). After purification, the product was obtained as a clear oil (0.19 g, 52 %).

R*f*= 0.54 (pentane : diethyl ether = 9:1); **^{1}H NMR** (500 MHz, Chloroform-d) δ 6.47 (s, 1H), 4.25 (q, J = 7.1 Hz, 2H), 2.78 (q, J = 7.5 Hz, 2H), 2.72 (q, J = 7.2 Hz, 2H), 1.33 (t, J = 7.1 Hz, 3H), 1.13 (t, J = 7.2 Hz, 3H), 1.04 (t, J = 7.5 Hz, 3H). **^{13}C NMR** (126 MHz, Chloroform-d) δ 202.9, 166.1, 156.5, 124.6, 60.9, 32.0, 20.6, 14.3, 13.8, 8.4.

Analytical data is in agreement with literature.[14]

Ethyl (E)-2-(2-oxocyclohexylidene)acetate (E-20)

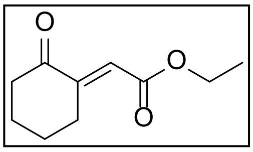

Compound was prepared according to the modified **General procedure F**. Cyclohexane-1,2-dione (5 mmol, 0.56 g), (Carbethoxymethylene)triphenylphosphorane (5 mmol, 1.74 g) and toluene (5 mL). After purification, product was obtained as a clear oil (0.59 g, 65 %).

R*f*= 0.76 (pentane : diethyl ether = 8:2); **^{1}H NMR** (599 MHz, Chloroform-d) δ 6.47 (t, J = 2.3 Hz, 1H), 4.20 (q, J = 7.1 Hz, 2H), 3.09 (td, J = 6.5, 2.3 Hz, 2H), 2.53 (t, J = 6.7 Hz, 2H), 1.92 (p, J = 6.5 Hz, 2H), 1.80 (p, J = 6.3 Hz, 2H), 1.29 (t, J = 7.1 Hz, 3H). **^{13}C NMR** (151 MHz, Chloroform-d) δ 201.4, 166.3, 151.4, 122.3, 60.7, 41.2, 28.9, 23.60, 23.59,z 14.3.

Analytical data is in agreement with literature.[6]

Ethyl (E)-2,3-dimethyl-4-oxopent-2-enoate (E-21)

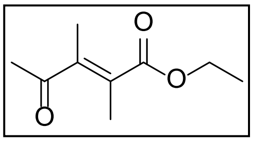

Compound was prepared according to the modified **General procedure F**. Butane-2,3-dione (5 mmol, 0.44 mL), (carbethoxyethylidene)triphenylphosphorane (5 mmol, 1.81 g) and toluene (5 mL). After purification, product was obtained as a clear oil (188 mg, 22 %).

R$_f$ = 0.41 (pentane : ethyl acetate = 9:1); **^{1}H NMR** (400 MHz, Chloroform-d) δ 4.23 (q, J = 7.1 Hz, 2H), 2.29 (s, 3H), 2.07 (q, J = 1.5 Hz, 3H), 1.89 (q, J = 1.6 Hz, 3H), 1.31 (t, J = 7.1 Hz, 3H). **^{13}C NMR** (101 MHz, Chloroform-d) δ 205.8, 168.7, 144.0, 127.0, 60.9, 29.2, 16.9, 16.7, 14.3.

Analytical data is in agreement with literature.[9]

Ethyl (Z)-2-fluoro-3-methyl-4-oxopent-2-enoate (Z-22)

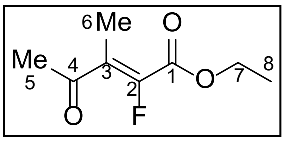

To the solution of lithium perchlorate (3.6 mmol, 0.38 g) in acetonitrile (10 mL) cooled to -10°C in an ice bath and salt, triethyl 2-fluoro-2-phosphonoacetate (3 mmol, 0.73 g) was added followed by diazabicycloundecene (DBU) (3.3 mmol, 0.51 g). The reaction mixture was stirred for 15 minutes and afterwards diacetyl (3 mmol, 0.26 g) was added in one portion. The reaction was stirred for 2 hours at room temperature, subsequently, it was diluted with diethyl ether (30 mL) and washed with saturated solution of ammonium chloride (40 mL). Organic phase was separated, dried with magnesium sulfate and evaporated under reduced pressure. Crude material was purified via column chromatography on silica. Product was obtained as a clear oil (166 mg 32%).

R$_f$= 0.36 (pentane : diethyl ether = 9:1); **^{1}H NMR** (599 MHz, Chloroform-d) δ 4.34 (q, J = 7.1 Hz, 2H, 7), 2.45 (d, J = 5.4 Hz, 3H, 5), 2.12 (d, J = 3.4 Hz, 3H, 6), 1.37 (t, J = 7.1 Hz, 3H, 8). **^{13}C-{^{19}F} NMR** (151 MHz, Chloroform-d) δ 199.6 (4), 161.1 (1), 149.0 (2), 127.7 (3), 62.3 (7), 31.6 (5), 14.2 (8), 12.7 (6). **^{19}F-{^{1}H} NMR** (564 MHz, Chloroform-d) δ -113.09. **IR (ATR)** ṽ = 2986 (w), 2937 (w), 1730 (s), 1686 (m), 1362 (m), 1316 (s), 1227 (s), 1062 (m), 1016 (m), 860 (m), 776 (m).

HRMS-ESI (m/z): 197.05837 ([M+Na]$^+$, calcd. for $C_8H_{11}FO_3Na^+$: 197.05844);

2-(dimethylamino)ethyl (2-(naphthalen-1-yl)ethyl) fumarate hydrochloride salt (E-23)

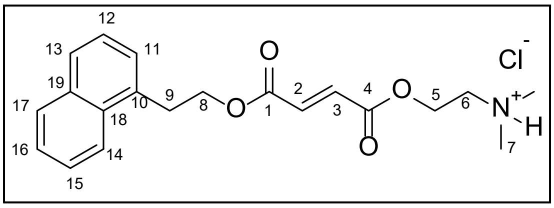

Compound was prepared according to the modified **General procedure C**. 2-(Naphthalen-1-yl)ethan-1-ol (5 mmol, 0.86 g) and dimethylethanolamine (5 mmol, 0.45 g) were used in this procedure. The product was obtained as clear thick oil (1.28 g, 75 %) and used immediately to obtain corresponding salt.

R*f*= 0.16 (diethyl ether : acetone = 9:1);

To the solution of amine (0.3 mmol, 102 mg) in diethyl ether (5 mL) a solution of hydrogen chloride in dioxane (4 M, 0.6 mmol, 0.15 mL) was added. The white precipitate was filtered off and washed with additional diethyl ether (10 mL) and then dried. The product was obtained as white powder in quantitative yield (113 mg).

^{1}H NMR (599 MHz, Chloroform-d) δ 13.05 (s, 1H), 8.07 (d, J = 8.4 Hz, 1H, 14), 7.86 (d, J = 8.1 Hz, 1H, 17), 7.76 (d, J = 8.1 Hz, 1H, 13), 7.54 (ddd, J = 8.4, 6.6, 1.4 Hz, 1H, 15), 7.49 (t, J = 7.4 Hz, 1H, 16), 7.41 (t, J = 7.6 Hz, 1H, 12), 7.37 (d, J = 6.9 Hz, 1H, 11), 6.92 (d, J = 15.7 Hz, 1H, 3), 6.82 (d, J = 15.8 Hz, 1H, 2), 4.72 (dd, J = 6.3, 3.9 Hz, 2H, 5), 4.54 (t, J = 7.3 Hz, 2H, 8), 3.46 (t, J = 7.3 Hz, 2H, 9), 3.39 (q, J = 5.0 Hz, 2H, 6), 2.88 (d, J = 4.6 Hz, 6H, 7). **^{13}C NMR** (151 MHz, Chloroform-d) δ 164.6 (1), 164.2 (4), 135.3 (3), 134.0 (19), 133.3 (10), 132.2 (2), 132.1 (18), 129.0 (17), 127.8 (13), 127.2 (11), 126.4 (15), 125.9 (16), 125.6 (12), 123.5 (14), 65.6 (8), 59.4 (5), 56.0 (6), 43.6 (7), 32.1 (9). **IR (ATR)** ῦ = 3425 (br), 3084 (w), 3019 (w), 2964 (w), 2902 (w), 2582 (br), 1713 (s), 1468 (m), 1293 (s), 1154 (s), 1062 (m), 971 (m), 801 (s), 678 (m).

HRMS-ESI (m/z): 342.16996 ([M+Na]$^{+}$, calcd. for $C_{20}H_{24}NO_4^{+}$: 342.16998);

Methyl (((2R,3R,4S,5R,6S)-3,4,5,6-tetramethoxytetrahydro-2H-pyran-2-yl)methyl) fumarate (E-24)

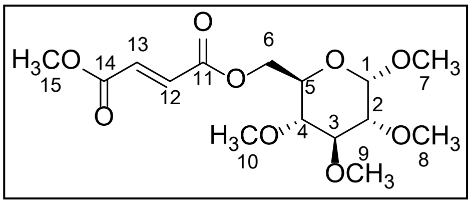

Compound was prepared according to the **General procedure E**. Permethyl glucose (1.5 mmol, 354 mg) was used. Product was obtained as a colourless thick oil (320 mg, 61 %).

R_f= 0.39 (pentane : diethyl ether = 6:4); **^{1}H NMR** (599 MHz, Chloroform-d) δ 6.89 (d, J = 15.9 Hz, 1H, 12 or 13), 6.85 (d, J = 15.8 Hz, 1H, 12 or 13), 4.78 (d, J = 3.6 Hz, 1H, 1), 4.43 (dd, J = 11.8, 2.2 Hz, 1H, 6), 4.34 (dd, J = 11.9, 5.3 Hz, 1H, 6'), 3.80 (s, 3H, 15), 3.72 (ddd, J = 10.1, 5.2, 2.2 Hz, 1H, 5), 3.61 (s, 3H, 9), 3.51 (s, 3H, 10), 3.50 (dd, *J* = 9.6, 8.8 Hz, 1H, 3), 3.50 (s, 3H, 8), 3.39 (s, 3H, 7), 3.18 (dd, J = 9.6, 3.6 Hz, 1H, 2), 3.06 (dd, J = 10.2, 8.8 Hz, 1H, 4). **^{13}C NMR** (151 MHz, Chloroform-d) δ 165.4 (14), 164.7 (11), 133.8 (12 or 13), 133.4 (12 or 13), 97.5 (1), 83.6 (3), 81.8 (2), 79.8 (4), 68.6 (5), 64.2 (6), 61.0 (9), 60.7 (10), 59.2 (8), 55.3 (7), 52.5 (15). **IR (ATR)** ῦ = 2936 (w), 2906 (w), 2838 (w), 1719 (s), 1436 (m), 1293 (s), 1154 (s), 968 (m), 900 (m), 736 (w).

HRMS-ESI (m/z): 371.1322 ([M+Na]$^+$, calcd. for $C_{15}H_{24}O_9Na^+$: 371.1313);

Cholesteryl methyl fumarate (E-25)

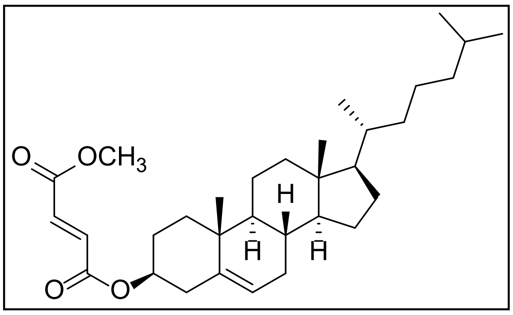

Compound was prepared according to the **General procedure D**. Cholesterol (1.5 mmol, 209 mg) was used. The product was obtained as a white waxy solid (305 mg, 40 %).

R_f = 0.22 (pentane : diethyl ether = 9:1); **^{1}H NMR** (500 MHz, Chloroform-d) δ 6.84 (s, 2H), 5.39 (d, J = 5.1 Hz, 1H), 4.72 (ddt, J = 16.3, 8.4, 4.3 Hz, 2H), 3.81 (s, 3H), 2.40 – 2.34 (m, 2H), 2.05 – 1.78 (m, 6H), 1.69 – 1.43 (m, 6H), 1.40 – 1.21 (m, 4H), 1.18 – 0.95 (m, 12H), 0.92 (d, J = 6.5 Hz, 3H), 0.87 (d, J = 2.3 Hz, 3H), 0.86 (d, J = 2.2 Hz, 3H), 0.68 (s, 3H). **^{13}C NMR** (126 MHz, Chloroform-d) δ 165.7, 164.5, 139.5, 134.6, 133.1, 123.2, 75.3, 56.8, 56.3, 52.4, 50.2, 42.5, 39.9, 39.7, 38.1, 37.1, 36.7, 36.3, 35.9, 32.06, 32.00, 28.4, 28.2, 27.8, 24.4, 24.0, 23.0, 22.7, 21.2, 19.5, 18.9, 12.0. **IR (ATR)** ῦ = 2944 (m), 2899 (m), 2868 (m), 1713 (s), 1641 (m), 1310 (s), 1290 (s), 994 (s), 815 (m), 774 (m).

HRMS-ESI (m/z): 521.3593 ([M+Na]$^+$, calcd. for $C_{32}H_{50}O_4Na^+$: 521.3601);

4-Bromophenyl (E)-4-(diethylamino)-4-oxobut-2-enoate (E-26)

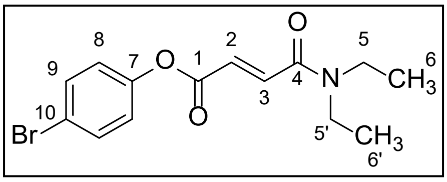

Compound was prepared according to the modified **General procedure C**. 4- Bromophenol (5 mmol, 0.87 g)

and diethylamine (5 mmol, 0.37 g) were used in this procedure. The product was obtained as thick oil (1.44 g, 88 %).

R*f*= 0.09 (pentane : diethyl ether = 6:4); **^{1}H NMR** (500 MHz, Chloroform-d) δ 7.54 – 7.48 (m, 3H, 3, 9), 7.06 – 7.02 (m, 2H, 8), 6.99 (d, J = 15.3 Hz, 1H, 2), 3.49 (q, J = 7.2 Hz, 2H, 5), 3.44 (q, J = 7.2 Hz, 2H, 5'), 1.24 (t, J = 7.2 Hz, 3H, 6'), 1.19 (t, J = 7.2 Hz, 3H, 6). **^{13}C NMR** (126 MHz, Chloroform-d) δ 164.1 (1), 163.5 (4), 149.6 (7), 136.5 (3), 132.7 (9), 129.9 (2), 123.3 (8), 119.3 (10), 42.7 (5'), 41.1 (5), 15.2 (6'), 13.1 (6). **IR (ATR)** ῦ = 2975 (w), 2933 (w), 2876 (w), 1730 (m), 1660 (m), 1434 (m), 1286 (s), 1135 (s), 1080 (m), 975 (m), 850 (s), 785 (m).

HRMS-ESI (m/z): 348.02058 ($[M+H]^+$, calcd. for $C_{14}H_{16}NO_3Na^+$: 348.02058);

Prop-2-yn-1-yl (E)-4-((2-hydroxyethyl)(methyl)amino)-4-oxobut-2-enoate (E-27)

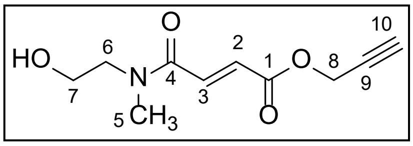

Compound was prepared according to the modified **General procedure C**. Propargyl alcohol (5 mmol, 0.28 g) and *N*-methylethanolamine (5 mmol, 0.38 g) were used in this procedure. After purification the product was obtained as white powder (0.56 g, 53 %).

NMR spectra contain two sets of signals due to the presence of two rotamers in ratio of 57:43.

R*f*= 0.11 (DCM : MeOH = 95:5); Major rotamer: **^{1}H NMR** (500 MHz, Chloroform-d) δ 7.44 (d, J = 15.3 Hz, 1H, 3), 6.81 (d, J = 15.3 Hz, 1H, 2), 4.79 (d, J = 2.5 Hz, 2H, 8), 3.81 (t, J = 5.2 Hz, 2H, 7), 3.62 (t, J = 5.2 Hz, 2H, 6), 3.19 (s, 3H, 5), 2.90 (br s, 1H, -OH), 2.51 – 2.50 (m, 1H, 10). **^{13}C NMR** (126 MHz, Chloroform-d) δ 165.9 (4), 164.9 (1), 134.7 (3), 130.7 (2), 77.2 (9), 75.5 (10), 61.1 (7), 52.7 (8), 51.6 (6), 37.1 (5). Minor rotamer: **^{1}H NMR** (500 MHz, Chloroform-d) δ 7.53 (d, J = 15.4 Hz, 1H), 6.77 (d, J = 15.3 Hz, 1H), 4.77 (d, J = 2.5 Hz, 2H), 3.78 (t, J = 5.4 Hz, 2H), 3.55 (t, J = 5.3 Hz, 2H), 3.04 (s, 3H), 2.90 (s, 1H), 2.51 – 2.49 (m, 1H). **^{13}C NMR** (126 MHz, Chloroform-d) δ 165.2, 165.0, 135.5, 129.8, 77.2, 75.3, 59.9, 52.5, 52.1, 34.3. **IR (ATR)** ῦ = 3400 (br), 3286 (w), 2873 (w), 2132 (w), 1717 (m), 1614 (m), 1289 (s), 1062 (m), 1020 (m), 763 (m).

HRMS-ESI (m/z): 234.07309 ($[M+Na]^+$, calcd. for $C_{10}H_{13}NO_4Na^+$: 234.07368);

4-Bromophenyl (E)-4-(benzyl(methyl)amino)-4-oxobut-2-enoate (E-28)

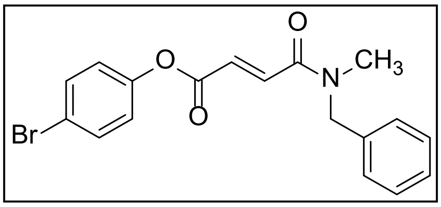

Compound was prepared according to the modified **General procedure C**. 4-Bromophenol (5 mmol, 0.87 g) and *N*-benzylmethylamine (5 mmol, 0.69 g) were used in this procedure. The product was obtained as a colourless thick oil (1.53 g, 82 %).

NMR spectra contain two sets of signals due to the presence of two rotamers in ratio of 54:46.

R*f*= 0.70 (pentane : diethyl ether = 7:3); **^{1}H NMR** (500 MHz, Chloroform-d) δ 7.60 (t, J = 15.4 Hz, 1H), 7.55 – 7.47 (m, 2H), 7.41 – 7.27 (m, 4H), 7.22 – 7.17 (m, 1H), 7.08 – 6.99 (m, 3H), 4.67 (d, J = 24.5 Hz, 2H), 3.06 (d, J = 7.2 Hz, 3H). **^{13}C NMR** (126 MHz, Chloroform-d) δ 164.9, 164.5, 163.95, 163.87, 149.55, 149.52, 136.5, 136.14, 136.11, 135.9, 132.71, 132.67, 130.55, 130.54, 129.3, 128.9, 128.3, 128.2, 127.9, 126.7, 123.33, 123.31, 119.40, 119.37, 53.8, 51.5, 35.2, 34.3. **IR (ATR)** ῦ = 3058 (w), 3102 (w), 2925 (w), 1737 (s), 1657 (s), 1396 (m), 1282 (s), 1096 (m), 980 (m), 807 (m), 691 (w).

HRMS-ESI (m/z): 396.0197 ([M+Na]$^+$, calcd. for $C_{18}H_{16}NO_3BrNa^+$: 396.0206);

Dimethyl 4,4'-(ethane-1,2-diylbis(methylazanediyl))(2E,2'E)-bis(4-oxobut-2-enoate) (E-29)

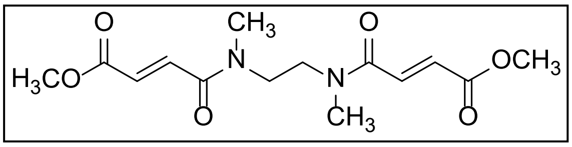

To the solution of *N,N'*-dimethylethane-1,2-diamine (2 mmol, 176 mg), monomethyl fumarate (4.5 mmol, 0.59 g) and 4-(dimethylamino)-pyridine (0.2 mmol, 24 mg) in dichloromethane (10 mL), EDCI (4.5 mmol, 0.86 g) was added. Reaction mixture was stirred at room temperature for 18 hours and then was diluted with additional dichloromethane (20 mL) and washed with brine (20 mL) then with hydrochloric acid (1 M, 5 mL). The organic phase was evaporated and purified via column chromatography. The product was obtained as white waxy solid (340 mg, 54 %).

NMR spectra contain three sets of signals due to the presence of three rotamers.

R*f*= 0.62 (DCM : MeOH = 9:1); **^{1}H NMR** (500 MHz, DMSO-d6) δ 7.40 – 7.25 (m, 2H), 6.54 – 6.42 (m, 2H), 3.79 – 3.68 (m, 6H), 3.65 – 3.47 (m, 4H), 3.10 – 2.84 (m, 6H). **^{13}C NMR** (126 MHz, DMSO-d_6) δ 165.46, 165.43, 165.41, 165.36, 164.18, 164.17, 163.9, 163.7, 135.2, 134.49, 134.40, 134.3, 129.7, 129.23, 129.11, 129.03, 52.01, 51.98, 51.92, 51.86, 47.96, 46.65,

46.28, 44.59, 36.31, 35.59, 33.96, 33.70. **IR (ATR)** ῡ = 2963 (w), 2926 (w), 2852 (w), 1717 (s), 1656 (m), 1440 (m), 1362 (w), 1078 (m), 995 (m), 813 (w) 705 (m).

HRMS-ESI (m/z): 335.1223 ($[M+Na]^+$, calcd. for $C_{14}H_{20}N_2O_6Na^+$: 335.1214);

4-Bromophenyl (E)-4-(diethylamino)-4-oxobut-2-enoate (E-30)

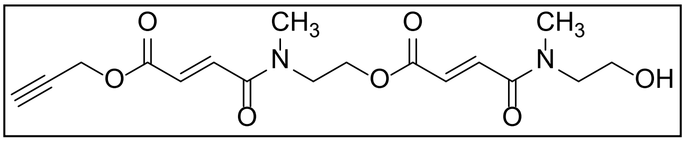

Compound was prepared according to the modified **General procedure C. E-27** (2 mmol, 0.42 g) and *N*-methylethanolamine (2 mmol, 0.15 g) were used in this procedure. The product was obtained as thick oil (0.27 g, 37 %).

NMR spectra are complicated due to the presence of four conformers in the solution.

R*f*= 0.21 (DCM : MeOH = 95:5; **^{1}H NMR** (599 MHz, Chloroform-d) δ 7.51 – 7.33 (m, 2H), 6.83 – 6.65 (m, 2H), 4.82 – 4.72 (m, 2H), 4.40 – 4.31 (m, 2H), 3.82 – 3.70 (m, 4H), 3.64 – 3.47 (m, 2H), 3.17 (dd, J = 9.6, 4.5 Hz, 3H), 3.08 – 2.99 (m, 3H), 2.54 – 2.48 (m, 1H), 2.02 (s, 1H). **^{13}C NMR** (151 MHz, Chloroform-d) δ 165.87, 165.64, 165.50, 165.41, 165.31, 165.21, 165.18, 165.17, 165.10, 164.85, 164.82, 164.78, 164.74, 164.71, 135.61, 135.52, 134.88, 134.83, 134.68, 134.66, 134.47, 134.42, 130.98, 130.73, 130.65, 130.57, 130.47, 130.34, 129.95, 129.58, 77.24, 77.20, 75.58, 75.56, 75.53, 75.49, 62.73, 62.10, 62.00, 61.47, 61.09, 61.03, 59.82, 59.73, 52.78, 52.74, 52.69, 52.39, 52.30, 51.62, 51.60, 48.69, 48.65, 47.49, 47.34, 37.18, 37.16, 37.13, 36.78, 34.47, 34.34, 34.25. **IR (ATR)** ῡ = 3405 (br), 3271 (br), 2951 (w), 2892 (w), 2130 (w), 1721 (s), 1647 (s), 1404 (m), 1267 (s), 1164 (s), 1071 (w), 864 (s), 763 (m).

HRMS-ESI (m/z): 389.13175 ($[M+Na]^+$, calcd. for $C_{17}H_{22}N_2O_7Na^+$: 389.13192);

N1,N1,N4,N4-tetrabenzylfumaramide (E-31)

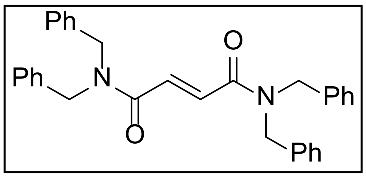

To a solution of fumaryl chloride (5 mmol, 0.76 g) in dichloromethane (25 mL), dibenzyl amine (10 mmol, 1.97 g) was added dropwise. The resulting mixture was cooled down in an ice bath and triethylamine (20 mmol, 2.02 g) was added dropwise. Afterwards, the reaction mixture was washed with hydrochloric acid solution (0.5 M, 50 mL). Organic phase was dried with magnesium sulfate and evaporated under reduced pressure. Crude

material was purified via column chromatography on silica. Product was obtained as a pale white crystalline solid (2.06 g, 87%).

R$_f$ = 0.27 (pentane : diethyl ether = 1:1); **^{1}H NMR** (599 MHz, Chloroform-d) δ 7.58 (s, 2H), 7.37 – 7.27 (m, 12H), 7.23 – 7.13 (m, 8H), 4.63 (s, 4H), 4.57 (s, 4H). **^{13}C NMR** (151 MHz, Chloroform-d) δ 165.9, 136.9, 136.1, 132.5, 129.1, 128.8, 128.5, 128.0, 127.8, 126.9, 50.2, 48.7.

IR (ATR) ῡ =3065 (w), 3027 (w), 2984 (w), 1633 (s), 1611 (s), 1440 (s), 1271 (m), 1079 (m), 946 (m), 821 (w), 748 (m).

Analytical data is in agreement with literature.[10]

4-Bromophenyl (E)-3-cyanoacrylate (E-32)

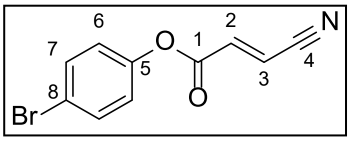

To the suspension of amide (**S-3**) (1 mmol, 286 mg) in acetonitrile (3 mL) phosphoryl chloride (2 mmol, 307 mg) was added dropwise. Then, reaction mixture was heated to 50 °C for one hour. Afterwards, mixture was cooled to room temperature and solvent was evaporated with the help of rotavap. Crude product was purified with column chromatography on silica. Product was obtained as white solid (160 mg, 63 %).

R$_f$ = 0.70 (pentane : diethyl ether = 7:3); **^{1}H NMR** (400 MHz, Chloroform-d) δ 7.57 – 7.49 (m, 2H, 7), 7.09 – 7.01 (m, 2H, 6), 6.90 (d, J = 16.3 Hz, 1H, 2), 6.67 (d, J = 16.3 Hz, 1H, 3). **^{13}C NMR** (101 MHz, Chloroform-d) δ 161.6 (1), 149.1 (5), 138.8 (2), 132.9 (7), 123.0 (6), 120.0 (8), 115.12 (4), 115.11 (3). **IR (ATR)** ῡ = 3079 (w), 3037 (w), 2226 (w), 1892 (w), 1733 (s), 1626 (m), 1482 (s), 1262 (s), 1112 (m), 1010 (m), 802 (s), 758 (m).

HRMS-ESI (m/z): 305.9736 ([M+MeOH+Na]$^+$, calcd. for $C_{11}H_{10}O_3BrNNa^+$: 305.9736);

Other intermediates

Ethyl 2-hydroxy-2-(2-oxotetrahydrofuran-3-yl)acetate (S-1)

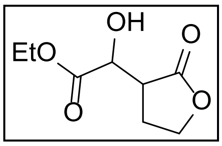

To the solution of diisopropylamine ((50 mmol, in THF (40 mL) at -78 °C, butyllithium (50 mmol, 1.6 M in hexanes, 31.25 mL) was added dropwise. The reaction mixture was stirred for 30 minutes. Afterwards, γ-butyrolactone (50 mmol, 4.31 g) dissolved in THF (10 mL) was added dropwise. The resulting solution was stirred for one hour at -78 °C before ethyl glyoxylate (50 mmol, 50% solution in

toluene, 10.1 mL) was added. The reaction mixture was allowed to warm to the room temperature and was quenched with saturated solution of aqueous ammonium chloride (50 mL) and diluted with dichloromethane (100 mL). The organic phase was separated, dried with magnesium sulphate and solvents were evaporated under reduced pressure. The crude product was filtered through short column of silica with pentane and diethyl ether (3 : 1) as eluents and was used as is without further purification. It was obtained 2.42 g (26 %) of crude material.

^{1}H NMR (599 MHz, Chloroform-d) δ 4.39 – 4.21 (m, 5H), 3.32 (dd, J = 4.4, 1.0 Hz, 1H), 3.18 (tdd, J = 9.7, 3.2, 1.0 Hz, 1H), 2.43 – 2.36 (m, 2H), 1.30 (td, J = 7.1, 1.1 Hz, 3H). **^{13}C NMR** (151 MHz, Chloroform-d) δ 176.0, 172.4, 69.4, 67.0, 62.6, 43.4, 24.9, 14.2.

Ketopantolactone (S-2)

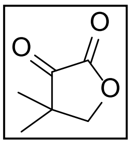

DL-pantolactone (30 mmol, 3.90 g) was dissolved in chloroform (30 mL) and water (5.5 mL). To this mixture, bromine (33 mmol, 5.28 g) was added slowly in one portion. The reaction mixture was stirred at mild reflux for 3 hours. Afterwards, mixture was allowed to cool to room temperature and transferred to a separatory funnel and washed with water (30 mL). The organic layer was dried with magnesium sulphate and evaporated to approx. 5 mL. To this residual solution diethyl ether (50 mL) was added and mixture was placed to a refrigerator. The product crystallised out of the solution over night and was collected via filtration (2.87 g, 75 %).

^{1}H NMR (400 MHz, Chloroform-d) δ 4.44 (s, 2H), 1.30 (s, 6H). **^{13}C NMR** (101 MHz, Chloroform-d) δ 198.2, 160.5, 77.2, 42.0, 22.3.

Analytical data is in agreement with literature.[15]

4-Bromophenyl (E)-4-amino-4-oxobut-2-enoate (S-3)

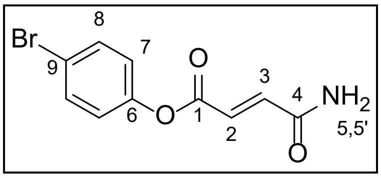

Compound was prepared according to the modified **General procedure C**. The procedure was carried out on double scale. 4-Bromophenol (10 mmol, 1.75 g) and solution of ammonia in dioxane (0.5 M, 10 mmol, 20 mL) were used in this procedure. The crude product was purified via crystallization from hot isopropyl alcohol and toluene mixture. The product was obtained as a white solid (1.24g, 43 %).

R$_f$ = 0.80 (DCM : MeOH = 9:1); **^{1}H NMR** (599 MHz, DMSO-d6) δ 7.98 (s, 1H, 5), 7.65 – 7.62 (m, 2H, 8), 7.61 (s, 1H, 5'), 7.23 – 7.19 (m, 2H, 7), 7.16 (d, J = 15.6 Hz, 1H, 2), 6.77 (d, J = 15.6 Hz, 1H, 3). **^{13}C NMR** (151 MHz, DMSO-d6) δ 164.2 (4 or 1), 163.6 (4 or 1), 149.4 (6), 139.7 (2), 132.4 (8), 127.7 (3), 124.1 (7), 118.5 (9). **IR (ATR)** ῦ = 3316 (br), 3149 (br), 1732 (s), 1674 (m), 1480 (s), 1222 (s), 1009 (s), 947 (m), 769 (w).

HRMS-ESI (m/z): 291.9561 ($[M+Na]^+$, calcd. for $C_{10}H_8NO_3BrNa^+$: 291.9580);

Crystal structures

X-Ray diffraction: Data sets for compounds ***Z*-14**, ***Z*-23,** ***Z*-28** and ***Z*-32** were collected with a Bruker D8 Venture PHOTON III diffractometer. Programs used: data collection: APEX3 V2019.1-0[1] (Bruker AXS Inc., **2019**); cell refinement: SAINT V8.40A (Bruker AXS Inc., **2019**); data reduction: SAINT V8.40A (Bruker AXS Inc., **2019**); absorption correction, SADABS V2016/2 (Bruker AXS Inc., **2019**); structure solution *SHELXT-2015*[2] (Sheldrick, G. M. *Acta Cryst.,* **2015**, *A71*, 3-8); structure refinement *SHELXL-2015*[3] (Sheldrick, G. M. *Acta Cryst.,* **2015**, *C71* (1), 3-8) and graphics, *XP*[4] (Version 5.1, Bruker AXS Inc., Madison, Wisconsin, USA, **1998**). *R*-values are given for observed reflections, and wR^2 values are given for all reflections.

Exceptions and special features: For compound ***Z*-23** the ammonium unit was found disordered over two positions in the asymmetric unit. Several restraints (SADI, SAME, ISOR and SIMU) were used in order to improve refinement stability.

X-ray crystal structure analysis of *Z*-14 (gil9697): A colorless plate-like specimen of $C_{11}H_9BrO_4$, approximate dimensions 0.051 mm x 0.130 mm x 0.147 mm, was used for the X-ray crystallographic analysis. The X-ray intensity data were measured. A total of 454 frames were collected. The total exposure time was 5.04 hours. The frames were integrated with the Bruker SAINT software package using a narrow-frame algorithm. The integration of the data using a monoclinic unit cell yielded a total of 9737 reflections to a maximum θ angle of 26.72° (0.79 Å resolution), of which 2399 were independent (average redundancy 4.059, completeness = 99.5%, R_{int} = 2.75%, R_{sig} = 3.73%) and 2287 (95.33%) were greater than 2σ(F^2). The final cell constants of a = 5.72170(10) Å, b = 10.8588(2) Å, c = 9.3899(2) Å, β = 100.3770(10)°, volume = 573.860(19) $Å^3$, are based upon the refinement of the XYZ-centroids of 6226 reflections above 20 σ(I) with 5.790° < 2θ < 53.37°. Data were corrected for absorption effects using the multi-scan method (SADABS). The ratio of minimum to maximum apparent transmission was 0.874. The calculated minimum and maximum transmission coefficients (based on crystal size) are 0.6210 and 0.8390. The structure was solved and refined using the Bruker SHELXTL Software Package, using the space group $P2_1$, with Z = 2 for the formula unit, $C_{11}H_9BrO_4$. The final anisotropic full-matrix least-squares refinement on F^2 with 146 variables converged at R1 = 1.77%, for the observed data and wR2 = 3.89% for all data. The goodness-of-fit was 0.970. The largest peak in the final difference electron density synthesis was 0.201 $e^-/Å^3$ and the largest hole was -0.206 $e^-/Å^3$ with an RMS deviation

of 0.041 e$^-$/Å^3. On the basis of the final model, the calculated density was 1.650 g/cm^3 and F(000), 284 e$^-$. Flack parameter was refined to: 0.014(5). CCDC Nr.: 2105407.

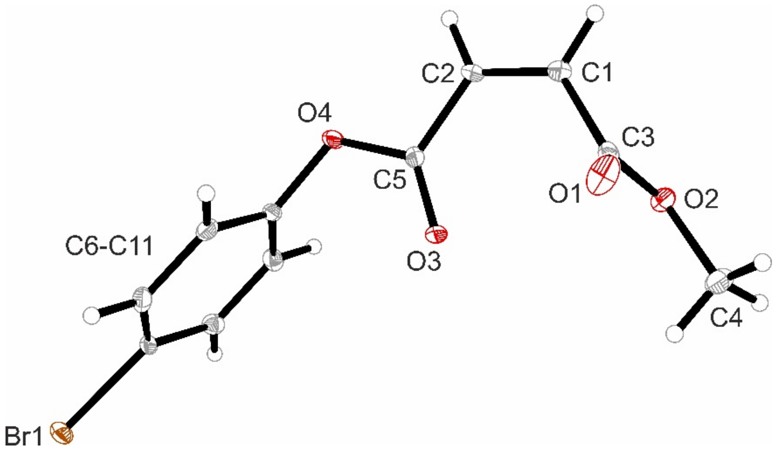

C5-C2-C1-C3 = -2.4°
C5-O3-C3-O1 = 119.7°

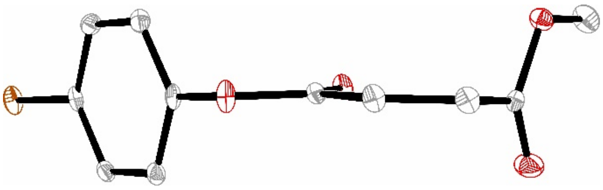

Figure S1: Crystal structure of compound ***Z*-14**.

Thermal ellipsoids are shown at 30% probability.

X-ray crystal structure analysis of *Z*-23 (glo9896): A colorless plate-like specimen of $C_{20}H_{24}ClNO_4$, approximate dimensions 0.035 mm x 0.134 mm x 0.232 mm, was used for the X-ray crystallographic analysis. The X-ray intensity data were measured on a 'Bruker D8 Venture PHOTON III Diffractometer' system equipped with a 'micro focus tube' 'Cu Ims' (CuK_α, λ = 1.54178 Å) and a 'MX mirror' monochromator. A total of 2647 frames were collected. The total exposure time was 42.26 hours. The frames were integrated with the Bruker SAINT software package using a narrow-frame algorithm. The integration of the data using an orthorhombic unit cell yielded a total of 16315 reflections to a maximum θ angle of 66.57° (0.84 Å resolution), of which 3246 were independent (average redundancy 5.026, completeness = 99.1%, R_{int} = 6.50%, R_{sig} = 4.62%) and 2870 (88.42%) were greater than

$2\sigma(F^2)$. The final cell constants of a = 60.0503(13) Å, b = 6.91000(10) Å, c = 9.3339(2) Å, volume = 3873.08(13) $Å^3$, are based upon the refinement of the XYZ-centroids of 5925 reflections above 20 $\sigma(I)$ with 11.78° < 2θ < 132.9°. Data were corrected for absorption effects using the Multi-Scan method (SADABS). The ratio of minimum to maximum apparent transmission was 0.846. The calculated minimum and maximum transmission coefficients (based on crystal size) are 0.6600 and 0.9350. The structure was solved and refined using the Bruker SHELXTL Software Package, using the space group *Iba*2, with Z = 8 for the formula unit, $C_{20}H_{24}ClNO_4$. The final anisotropic full-matrix least-squares refinement on F^2 with 282 variables converged at R1 = 3.75%, for the observed data and wR2 = 7.94% for all data. The goodness-of-fit was 1.046. The largest peak in the final difference electron density synthesis was 0.203 $e^-/Å^3$ and the largest hole was -0.187 $e^-/Å^3$ with an RMS deviation of 0.041 $e^-/Å^3$. On the basis of the final model, the calculated density was 1.296 g/cm^3 and F(000), 1600 e^-. The hydrogen atoms at N1 and N1A were refined freely, but with DFIX and U-fixed value) CCDC Nr.: 2105408.

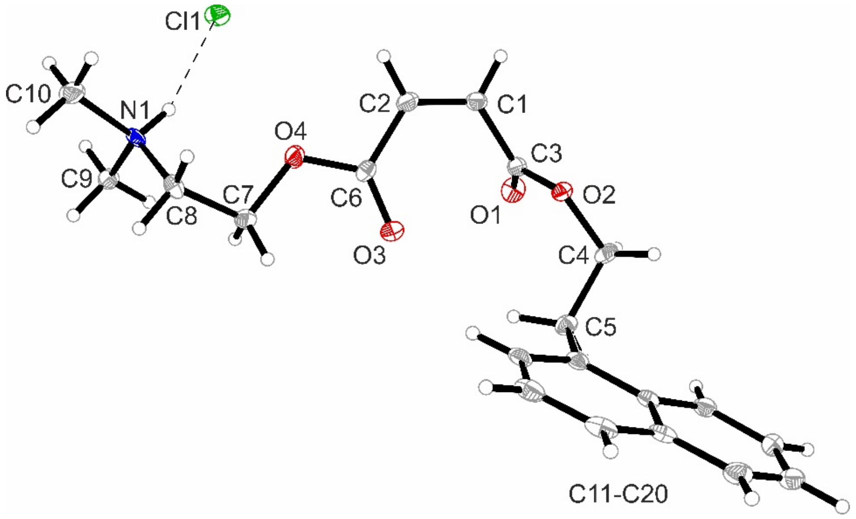

C6-C2-C1-C3 = 4.1°
C6-O3-C3-O1 = -109.9°

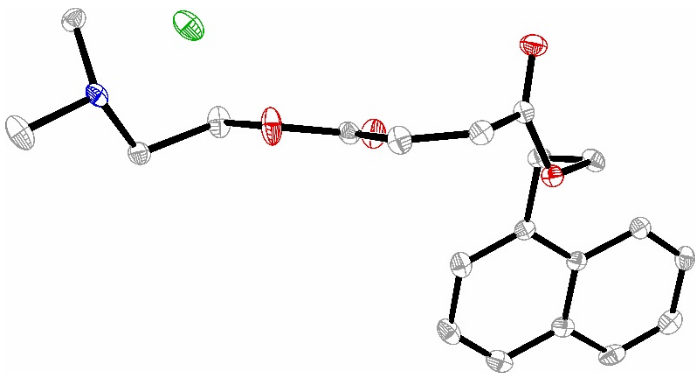

Figure S2: Crystal structure of compound ***Z*-19**.

Thermal ellipsoids are shown at 30% probability.

X-ray crystal structure analysis of *Z*-28 (gil9791): A colorless plate-like specimen of $C_{18}H_{16}BrNO_3$, approximate dimensions 0.039 mm x 0.177 mm x 0.210 mm, was used for the X-ray crystallographic analysis. The X-ray intensity data were measured (λ = 0.71073 Å). A total of 774 frames were collected. The total exposure time was 6.45 hours. The frames were integrated with the Bruker SAINT software package using a narrow-frame algorithm. The integration of the data using a monoclinic unit cell yielded a total of 18694 reflections to a maximum θ angle of 26.02° (0.81 Å resolution), of which 3110 were independent (average redundancy 6.011, completeness = 99.7%, R_{int} = 4.53%, R_{sig} = 3.03%) and 2764 (88.87%) were greater than $2\sigma(F^2)$. The final cell constants of a = 5.8586(2) Å, b = 7.5465(3) Å, c = 35.7576(14) Å, β = 90.087(2)°, volume = 1580.91(10) Å^3, are based upon the refinement of the XYZ-centroids of 8787 reflections above 20 σ(I) with 5.859° < 2θ < 53.45°. Data were corrected for absorption effects using the Multi-Scan method (SADABS). The ratio of minimum to maximum apparent transmission was 0.719. The calculated minimum and maximum transmission coefficients (based on crystal size) are 0.6100 and 0.9050. The structure was solved and refined using the Bruker SHELXTL Software Package, using the space group $P2_1/n$, with Z = 4 for the formula unit, $C_{18}H_{16}BrNO_3$. The final anisotropic full-matrix least-squares refinement on F^2 with 209 variables converged at R1 = 4.37%, for the observed data and wR2 = 8.74% for all data. The goodness-of-fit was 1.166. The largest peak in the final difference electron density synthesis was 0.478 $e^-/Å^3$ and the largest hole was -0.508 $e^-/Å^3$ with an RMS deviation of 0.082 $e^-/Å^3$. On the basis of the final model, the calculated density was 1.572 g/cm^3 and F(000), 760 e^-. CCDC Nr.: 2105409.

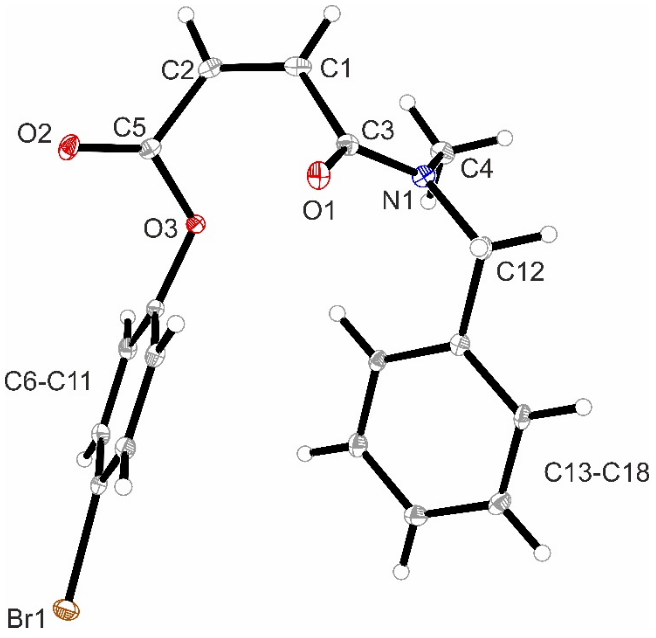

C5-C2-C1-C3 = -6.1°
C5-O2-C3-O1 = -156.4°

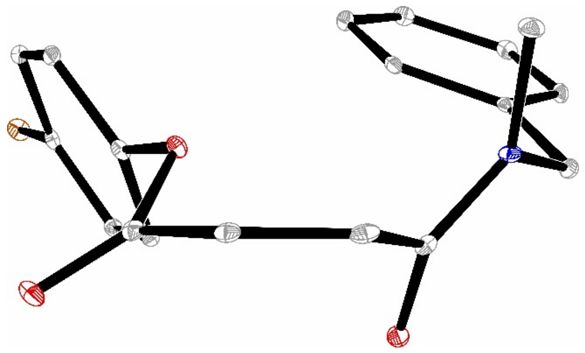

Figure S3: Crystal structure of compound ***Z*-24**.

Thermal ellipsoids are shown at 30% probability.

X-ray crystal structure analysis of *Z*-32 (gil9837): A colorless plate-like specimen of $C_{10}H_6BrNO_2$, approximate dimensions 0.032 mm x 0.084 mm x 0.212 mm, was used for the X-ray crystallographic analysis. The X-ray intensity data were measured (λ = 1.54178 Å). A total of 1319 frames were collected. The total exposure time was 20.30 hours. The frames were integrated with the Bruker SAINT software package using a wide-frame algorithm. The integration of the data using a monoclinic unit cell yielded a total of 12900 reflections to a maximum θ angle of 66.37° (0.84 Å resolution), of which 1676 were independent (average redundancy 7.697, completeness = 99.7%, R_{int} = 17.10%, R_{sig} = 10.20%) and 1168 (69.69%) were greater than $2\sigma(F^2)$. The final cell constants

of a = 21.9062(13) Å, b = 5.5200(3) Å, c = 7.9171(5) Å, β = 96.445(5)°, volume = 951.30(10) $Å^3$, are based upon the refinement of the XYZ-centroids of 2733 reflections above 20 σ(I) with 8.123° < 2θ < 131.4°. Data were corrected for absorption effects using the Multi-Scan method (SADABS). The ratio of minimum to maximum apparent transmission was 0.659. The calculated minimum and maximum transmission coefficients (based on crystal size) are 0.3790 and 0.8390. The structure was solved and refined using the Bruker SHELXTL Software Package, using the space group $P2_1/c$, with Z = 4 for the formula unit, $C_{10}H_6BrNO_2$. The final anisotropic full-matrix least-squares refinement on F^2 with 127 variables converged at R1 = 8.34%, for the observed data and wR2 = 23.60% for all data. The goodness-of-fit was 1.081. The largest peak in the final difference electron density synthesis was 2.136 $e^-/Å^3$ and the largest hole was -1.165 $e^-/Å^3$ with an RMS deviation of 0.191 $e^-/Å^3$. On the basis of the final model, the calculated density was 1.760 g/cm^3 and F(000), 496 e^-. CDC Nr.: 2105410.

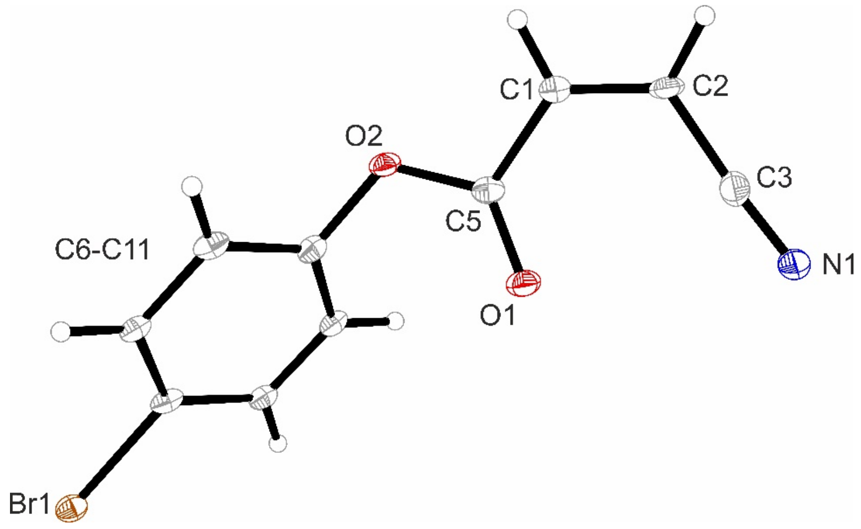

C5-C1-C2-C3 = 0.6°
C5-O1-C3-N1 = -172.7°

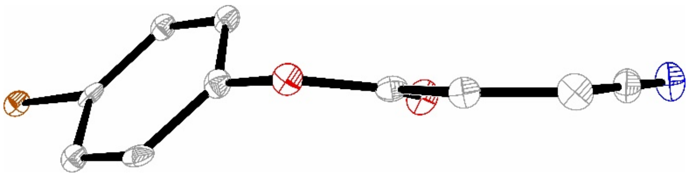

Figure S4: Crystal structure of compound ***Z*-32**.

Thermal ellipsoids are shown at 30% probability.

References X-Ray Part:

1. Bruker AXS (**2019**) *APEX3 Version 2019.1-0*, *SAINT Version 8.40A* and *SADABS Bruker AXS area detector scaling and absorption correction Version 2016/2*, Bruker AXS Inc., Madison, Wisconsin, USA.
2. Sheldrick, G. M., *SHELXT – Integrated space-group and crystal-structure determination*, *Acta Cryst.,* **2015**, *A71*, 3-8.
3. Sheldrick, G.M., *Crystal structure refinement with SHELXL*, *Acta Cryst.*, **2015**, *C71 (1)*, 3-8.
4. Bruker AXS (**1998**) *XP – Interactive molecular graphics, Version 5.1,* Bruker AXS Inc., Madison, Wisconsin, USA.

Computational data

Method

All calculation performed in this study were done in Gaussian 16, Revision B.01.[16] Optimization of all structure was done without geometrical constraints using PBE0[17] hybrid density functional with Grimme's dispersion with Becke-Johnson damping (GD3BJ).[18] For calculations a triple zeta basis set def2-TZVP[19] by Ahlrichs and co-workers or def2-TZVPD[20] variant including diffusion functions were used. Basis set was obtained st https://www.basissetexchange.org/[21]. Acetonitrile solvation was implemented using implicit solvent model. Stationary points were investigated *via* frequency analysis – all minima were without presence of imaginary frequencies. Gibbs free energies were calculated at 298.15K and 1.00 atm. Triplet excitation energies were calculated using time-dependent DFT (TD-DFT) with functional LC-BLYP[22,23] and def2-TZVP basis set. For visualization and rendering images of structures CYLview20[24] software was used.

Non-covalent interaction analysis

To obtain further insight into non-covalent interaction taking place between carbonyls of *Z*-configured alkenes, NBO[25] analysis was carried out To visualize orbitals, Multiwfn[26] software was used. Resulting pictures of desired orbitals were created with the isovalue = 0.035.

Results

Six different molecules were investigated, (E-14, Z-14, E-28, Z-28, E-32, Z-32) and in the case of Z-12, E-24 and Z-24 several low-lying conformers were identified. Summary of electronic energies, Gibbs free energies and triplet excitation energies can be found in the Table 1.

Table 1 Summary basic calculated properties of selected molecules

#	E (PBE0-D3BJ) [Eh]	G (PBE0-D3BJ) [Eh]	ΔG rel. [kJ/mol]	ET_1 [kJ/mol]
E-14	-3298.838005	-3298.702247	0	281
Z-14 (1)	-3298.831946	-3298.699173	8.1	303
Z-14 (2)	-3298.832652	-3298.698117	10.8	303
E-28 (1)	-3549.127606	-3548.88246	0	286
E-28 (2)	-3549.127192	-3548.882038	1.1	285
Z-28 (1)	-3549.126606	-3548.878774	9.7	303
Z-28 (2)	-3549.125162	-3548.879839	6.9	303
Z-28 (3)	-3549.125874	-3548.879648	7.4	303
Z-28 (4)	-3549.121928	-3548.872153	27.1	302
E-32	-3163.279212	-3163.186316	0	262
Z-32	-3163.277713	-3163.184656	4.4	263

Cartesian coordinates of optimized structures

E-14

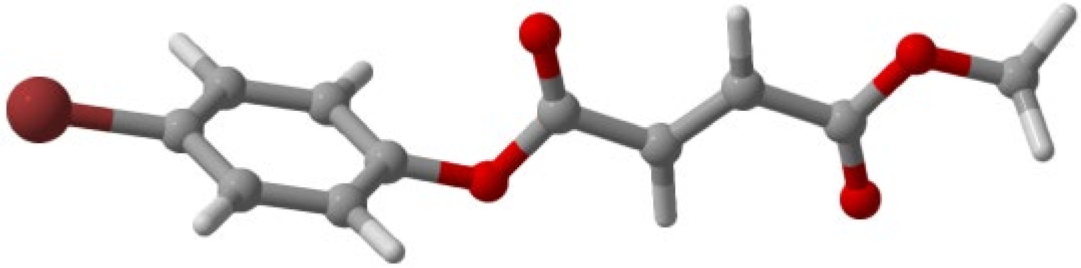

C	2.89250100	0.34792100	0.22352300
H	3.02831700	1.25050000	0.80691200

C	3.93198500	-0.34577700	-0.22454600
H	3.79433600	-1.24804200	-0.80848900
C	1.51400800	-0.09349600	-0.06303600
C	5.31618000	0.09343600	0.06071000
O	5.61627500	1.07433100	0.69687200
O	1.19921100	-1.06931800	-0.68881000
O	6.19434100	-0.74444800	-0.48208700
O	0.63089200	0.76799000	0.48489700
C	7.57206900	-0.42721400	-0.27461600
H	7.80193100	-0.42242600	0.79104300
H	8.13444300	-1.20613100	-0.78190700
H	7.80297500	0.54951700	-0.70011200
C	-0.72056800	0.49643500	0.31372200
C	-1.36701000	-0.32259900	1.22051100
C	-1.39963000	1.10621100	-0.72418800
C	-2.72914900	-0.54120400	1.08532800
H	-0.81130000	-0.78414200	2.02735000
C	-2.76227400	0.89081200	-0.86248200
H	-0.86774600	1.74610200	-1.41732400
C	-3.41126800	0.06806400	0.04409000
H	-3.25063300	-1.17898900	1.78691600
H	-3.30908500	1.36162900	-1.66886800
Br	-5.27057300	-0.22662800	-0.14047400

E-28 (1)

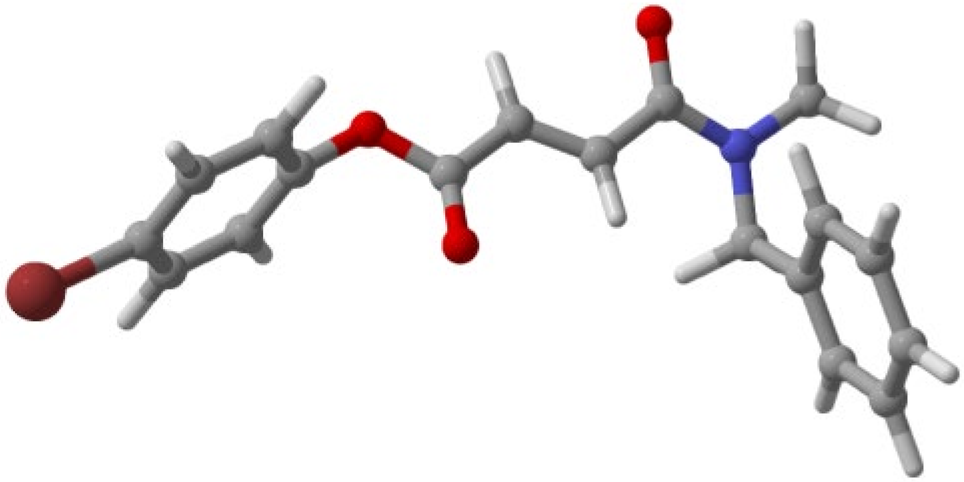

C	2.13937300	1.17447700	-0.02363300
H	2.22613500	0.11343000	-0.22581100
C	0.93119900	1.70352200	0.13404000
H	0.80238000	2.75691300	0.34925900
C	3.35837600	2.01774300	0.13096900
C	-0.27311800	0.86296300	0.02344400
O	-0.29928800	-0.31838700	-0.19812200
O	3.30174800	3.09819600	0.71107700
O	-1.38116900	1.61453000	0.21237600
C	5.75090600	2.21621500	-0.12360000
H	6.03046100	2.82160100	-0.99110000
H	5.64044100	2.86602300	0.74039100
H	6.54176400	1.48814500	0.06463000
C	-2.61123000	0.97703100	0.13242200
C	-3.23996700	0.59181800	1.30143700
C	-3.20883000	0.80137900	-1.10212700
C	-4.49855000	0.01340500	1.23731100
H	-2.74973700	0.74523700	2.25483500
C	-4.46632500	0.22254900	-1.17110400
H	-2.69643700	1.11675800	-2.00258500
C	-5.09677200	-0.16573600	0.00055600
H	-5.00447900	-0.29243500	2.14365200
H	-4.94800500	0.07860900	-2.12937000
Br	-6.81410500	-0.95373500	-0.09114500
N	4.50948600	1.51296100	-0.35997200
C	4.59237100	0.33937100	-1.20111500
H	3.64607500	0.19491500	-1.72510500
H	5.32521700	0.54874900	-1.98546600
C	4.98394500	-0.92653700	-0.47894500
C	5.41084600	-2.02330200	-1.22385400
C	4.90386900	-1.04125700	0.90262900
C	5.74276000	-3.21437100	-0.59917500
H	5.48371000	-1.94140800	-2.30386900
C	5.24044100	-2.23371000	1.53151600
H	4.58398400	-0.19319600	1.49730400
C	5.65827800	-3.32347500	0.78376600

H	6.07313900	-4.05971700	-1.19198300
H	5.17554500	-2.30729800	2.61110300
H	5.92131500	-4.25354200	1.27413000

E-28 (2)

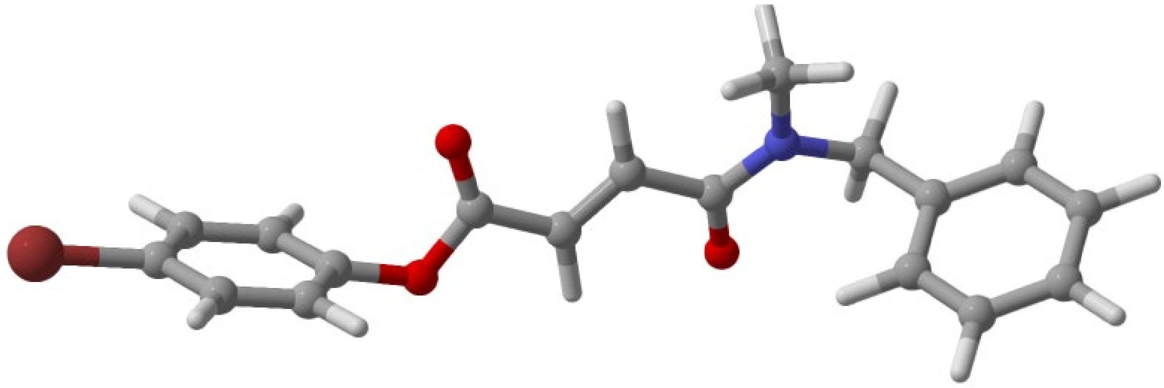

C	-1.64464800	-0.95337100	0.15577000
H	-1.47099900	-1.17977100	1.20027600
C	-0.61631400	-0.60703800	-0.61053400
H	-0.74984900	-0.38076800	-1.66103800
C	-3.01109100	-1.06580500	-0.42617300
C	0.74247100	-0.52422500	-0.04796000
O	1.05785300	-0.75136000	1.08972500
O	-3.16502700	-1.13751300	-1.64333400
O	1.61862700	-0.14824600	-1.00668800
C	-3.92589900	-0.91421900	1.87720700
H	-4.83751000	-0.43169200	2.23152700
H	-3.81299200	-1.86850300	2.39957700
H	-3.09640700	-0.25929700	2.13036300
C	2.95215600	-0.02330200	-0.64242300
C	3.79140100	-1.11443500	-0.76992700
C	3.42343500	1.20671600	-0.22297800
C	5.13722800	-0.97474100	-0.46811600
H	3.39613800	-2.06539400	-1.10508000
C	4.76861100	1.35107300	0.07982700
H	2.74427100	2.04579400	-0.13618300
C	5.61019700	0.25734700	-0.04547600
H	5.80754700	-1.81884700	-0.56368300
H	5.15371600	2.30747200	0.40841400

Br	7.44655300	0.45180500	0.36511600
N	-4.04101000	-1.10161800	0.44437900
C	-5.38080500	-1.33811300	-0.05482100
H	-5.28424900	-1.70397500	-1.07735500
H	-5.83814600	-2.12919600	0.54630100
C	-6.25262700	-0.10993200	-0.02269500
C	-7.57058800	-0.19690600	0.41030700
C	-5.76240700	1.12159000	-0.44794100
C	-8.39099800	0.92336400	0.41059900
H	-7.95787800	-1.15058600	0.75433600
C	-6.57772000	2.24298000	-0.44417400
H	-4.73387700	1.20197300	-0.78266900
C	-7.89600500	2.14682100	-0.01650100
H	-9.41610800	0.84060000	0.75346200
H	-6.18336200	3.19636100	-0.77725400
H	-8.53260100	3.02408500	-0.01225900

E-32

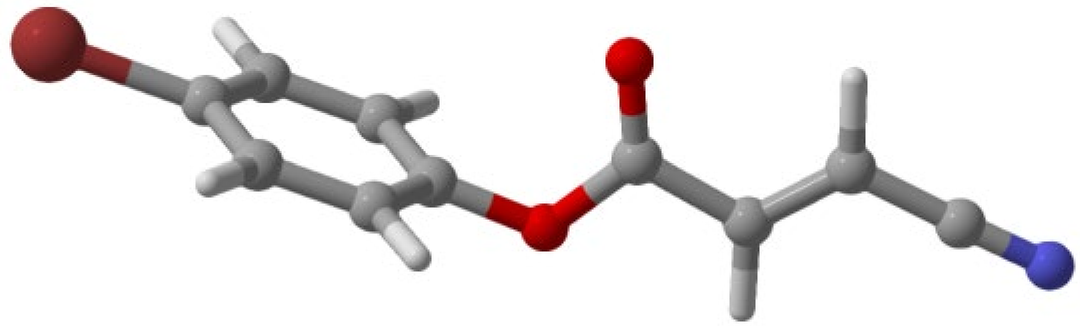

C	4.86632800	-0.02749900	-0.55435400
H	4.66976300	-0.08047700	-1.62050400
C	3.86229300	0.01661300	0.31922000
H	4.02193300	0.06995900	1.38837800
C	6.22611500	-0.00811300	-0.14982700
C	2.47034600	-0.00750900	-0.17766700
O	2.14186600	-0.06443500	-1.33097400
O	1.61217600	0.04438600	0.85768200
C	0.25254800	0.02895100	0.56412500
C	-0.40689000	-1.18334700	0.49302000
C	-0.41491400	1.22954000	0.41448200

C	-1.77373200	-1.19728000	0.26191400
H	0.14184700	-2.10834100	0.61937700
C	-1.78182300	1.21943700	0.18337600
H	0.12793800	2.16425400	0.48008700
C	-2.44538200	0.00514600	0.10854100
H	-2.30646300	-2.13733000	0.20387400
H	-2.32080800	2.15013600	0.06435100
Br	-4.31017100	-0.01136400	-0.20577800
N	7.33715900	0.00633000	0.15372000

Z-14 (1)

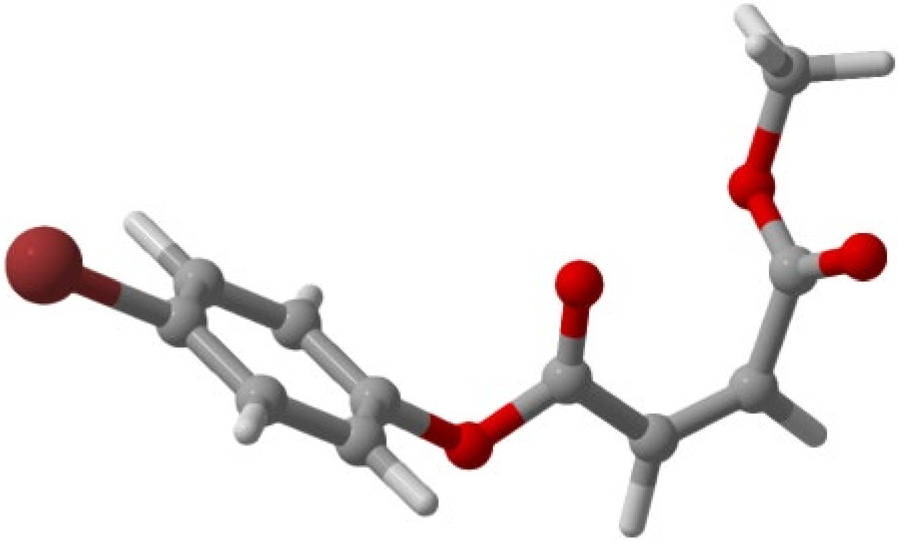

C	3.13530000	-1.66536500	-0.40340700
H	3.03388500	-2.67899100	-0.76924200
C	4.33365500	-1.13320400	-0.19494300
H	5.22749300	-1.72017200	-0.38168800
C	1.89688900	-0.91447700	-0.14063400
C	4.58533600	0.23036500	0.35248600
O	4.85132700	0.42818600	1.51097000
O	1.82738700	0.18655200	0.33828300
O	4.56623500	1.15660200	-0.59182100
O	0.82053400	-1.64243300	-0.50838300
C	4.82021600	2.49700400	-0.15811900
H	5.80159700	2.56470300	0.31083400

H	4.78353600	3.10802200	-1.05560400
H	4.05323100	2.81173500	0.54952800
C	-0.43158200	-1.06876500	-0.33278300
C	-0.93779400	-0.22712500	-1.30600800
C	-1.16891400	-1.40761400	0.78590200
C	-2.21393900	0.29253900	-1.15629600
H	-0.34043900	0.01859100	-2.17524100
C	-2.44653500	-0.89094300	0.93913100
H	-0.74799200	-2.07190100	1.53043800
C	-2.95385600	-0.04529600	-0.03403200
H	-2.62524000	0.95214200	-1.90909200
H	-3.03724400	-1.14749500	1.80871000
Br	-4.69672400	0.66084000	0.17117700

Z-14 (2)

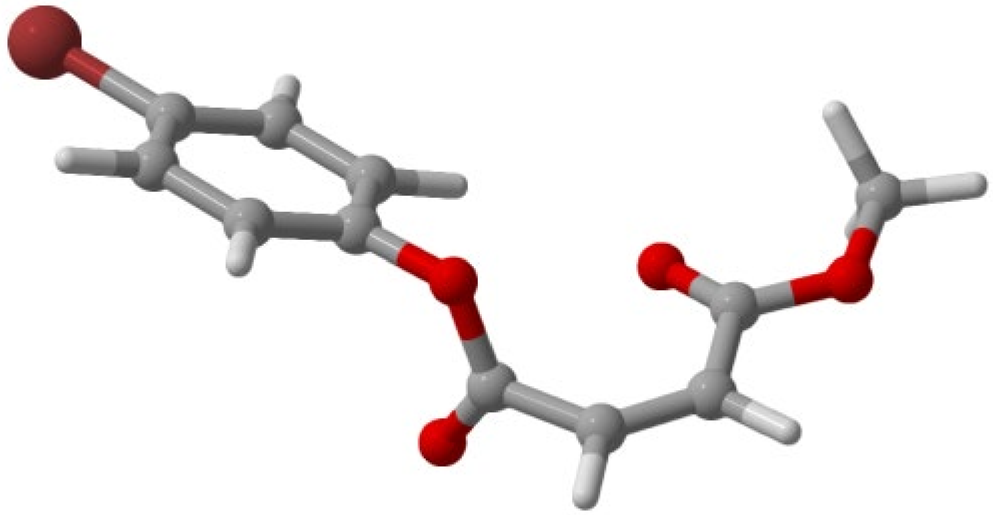

C	-3.25893400	-1.85925300	0.06766700
H	-3.45751100	-2.92371400	-0.00526400
C	-4.23316100	-0.97951900	-0.12705600
H	-5.23421200	-1.30918500	-0.37446700
C	-1.86550000	-1.51023400	0.45522100
C	-4.00601500	0.47371600	-0.00618200
O	-2.97292700	0.98193000	0.36439100
O	-1.44316900	-1.60189300	1.57364800
O	-5.08976500	1.15940300	-0.35358800
O	-1.12738200	-1.18079300	-0.61769200

C	-4.98141600	2.58211100	-0.27481000
H	-4.19372800	2.93870100	-0.93868700
H	-5.94683700	2.96968400	-0.58808300
H	-4.76089900	2.88839200	0.74788300
C	0.18386900	-0.77336100	-0.39993000
C	1.20851900	-1.60586100	-0.80551900
C	0.43081500	0.47359100	0.14538000
C	2.52196100	-1.18304300	-0.66197600
H	0.98228200	-2.57506300	-1.23232200
C	1.74172800	0.89724400	0.29297600
H	-0.39821300	1.10159800	0.44654000
C	2.77275100	0.06333000	-0.11234800
H	3.33731500	-1.82146900	-0.97586500
H	1.95538800	1.86947600	0.71750600
Br	4.56270400	0.64343100	0.08651900

Z-28 (1)

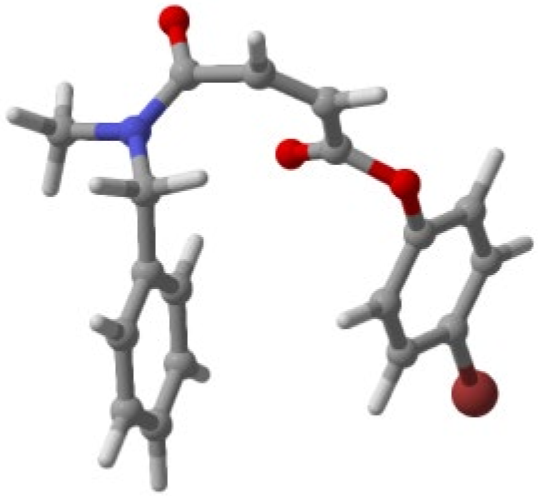

C	-3.22694100	-2.31083400	-0.76769500
H	-4.05019000	-2.70660400	-1.35655800
C	-1.99384000	-2.39384000	-1.25943700
H	-1.82094200	-2.82858900	-2.23588300
C	-3.61987100	-1.82341900	0.59280500
C	-0.81738100	-1.88024900	-0.54580900
O	-0.77281900	-1.53592200	0.60573100
O	-3.67332800	-2.64402300	1.50168500

O	0.25029900	-1.83961400	-1.37751600
C	-4.45566000	-0.04524000	1.99339100
H	-5.53694600	0.11706600	1.95293600
H	-4.23111300	-0.77850000	2.76296900
H	-3.96850300	0.90026700	2.23623100
C	1.41935900	-1.27518900	-0.89051600
C	2.48758900	-2.10330700	-0.60408200
C	1.51532600	0.09927700	-0.76331400
C	3.68412900	-1.54601500	-0.17730800
H	2.38495700	-3.17566900	-0.71470800
C	2.70672900	0.65978200	-0.33316500
H	0.66441300	0.72745100	-0.99632500
C	3.77893700	-0.17051700	-0.04453500
H	4.53041600	-2.18103200	0.04964900
H	2.79625100	1.73308300	-0.22691400
Br	5.40834100	0.59297400	0.53983500
N	-3.97096400	-0.53171600	0.72097100
C	-4.06230300	0.38067600	-0.39701100
H	-3.82082500	-0.16811800	-1.30994900
H	-5.10157700	0.71229000	-0.49730500
C	-3.16527800	1.58853500	-0.29300100
C	-3.54955900	2.77457300	-0.91050500
C	-1.94523700	1.53893100	0.37129300
C	-2.72346300	3.88846300	-0.87856600
H	-4.50646600	2.82695300	-1.42026600
C	-1.12118900	2.65522800	0.41186500
H	-1.63727300	0.62092300	0.85772100
C	-1.50472400	3.83202000	-0.21593700
H	-3.03613100	4.80507300	-1.36569300
H	-0.17391300	2.60359800	0.93725100
H	-0.86030500	4.70296500	-0.18424100

Z-28 (2)

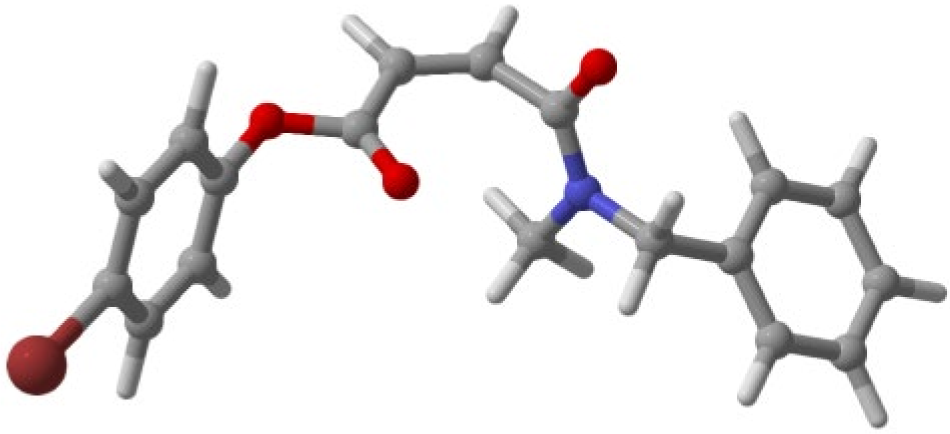

C	-1.94303500	2.53755500	0.24771000
H	-2.64102700	3.32624000	0.51406700
C	-0.65495000	2.69209100	0.54021600
H	-0.30690100	3.58595700	1.04214700
C	-2.54601700	1.43496300	-0.56389100
C	0.35660100	1.67951700	0.20817500
O	0.16646700	0.63915000	-0.36483400
O	-2.72088500	1.65452000	-1.75946200
O	1.57503000	2.07990700	0.64246100
C	-2.70225200	0.05286900	1.46304300
H	-1.90051400	-0.68085500	1.58852200
H	-2.42832900	0.96699800	1.98387400
H	-3.61423200	-0.34249700	1.91479900
C	2.64568500	1.22697800	0.41901000
C	3.47962900	1.47161700	-0.65578400
C	2.89397500	0.19537500	1.30600400
C	4.59049600	0.66525800	-0.85216300
H	3.26253700	2.28837400	-1.33298600
C	4.00223400	-0.61424100	1.11295800
H	2.22710300	0.02711100	2.14263200
C	4.83812400	-0.36990400	0.03468800
H	5.25359900	0.84518000	-1.68820100
H	4.21061300	-1.42522400	1.79845100
Br	6.35302200	-1.47200900	-0.22885200
N	-2.93603300	0.31578700	0.06264400
C	-3.51168900	-0.76960100	-0.70478400
H	-3.42319400	-0.49854500	-1.75750400

H	-2.91445200	-1.67125600	-0.53831800
C	-4.95066500	-1.03768100	-0.35254100
C	-5.39262300	-2.33667700	-0.13276200
C	-5.86400900	0.00946400	-0.26474300
C	-6.72610700	-2.59043700	0.16088900
H	-4.68537900	-3.15796800	-0.18879500
C	-7.19466900	-0.23986500	0.03304300
H	-5.52772400	1.02800500	-0.42777600
C	-7.63041600	-1.54212300	0.24517300
H	-7.05600900	-3.60889300	0.33171700
H	-7.89570300	0.58452300	0.09885000
H	-8.67072700	-1.73690100	0.47905900

Z-28 (3)

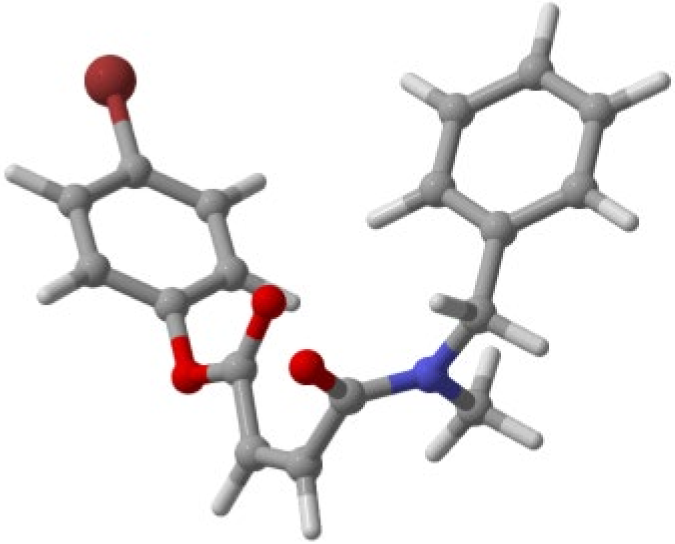

C	-2.62579700	-3.07030100	0.09547000
H	-3.28489300	-3.91024000	0.29850800
C	-1.32155900	-3.21765000	0.30906300
H	-0.92804600	-4.15535700	0.68038600
C	-3.31538800	-1.90233600	-0.53870700
C	-0.35349800	-2.13984700	0.07072900
O	-0.59753000	-1.04114700	-0.35381000
O	-3.46072900	-1.92895800	-1.75710900
O	0.89389600	-2.55099500	0.39801900
C	-3.64119400	-0.92196100	1.69370600
H	-4.61185900	-0.93104800	2.19641300

H	-3.06798400	-1.78522500	2.02234000
H	-3.10528400	-0.01551500	1.98590600
C	1.92567200	-1.63363100	0.26810500
C	2.07875800	-0.62989200	1.20778700
C	2.81945000	-1.78889500	-0.77440200
C	3.14942000	0.24292900	1.10038800
H	1.36730900	-0.53108800	2.01821400
C	3.89448500	-0.91942500	-0.88410600
H	2.67598800	-2.58502700	-1.49438400
C	4.04573100	0.08822900	0.05415200
H	3.28273600	1.03346300	1.82720000
H	4.60350800	-1.02930600	-1.69413500
Br	5.50969200	1.27750300	-0.09241600
N	-3.82750600	-0.95453400	0.26194700
C	-4.63059400	0.10845900	-0.30677000
H	-4.79872100	-0.14659600	-1.35403800
H	-5.60229400	0.11634600	0.19656300
C	-3.99641600	1.47056100	-0.19922000
C	-4.77876200	2.58050400	0.09896200
C	-2.63434100	1.64424000	-0.42657700
C	-4.21532500	3.84801700	0.15672500
H	-5.83944700	2.45189900	0.29014900
C	-2.06879600	2.90924200	-0.36176300
H	-2.01303600	0.78184700	-0.63950900
C	-2.85728300	4.01580900	-0.07354200
H	-4.83775900	4.70440300	0.39038100
H	-1.00563200	3.03146900	-0.53693900
H	-2.41373300	5.00360600	-0.02290400

Z-28 (4)

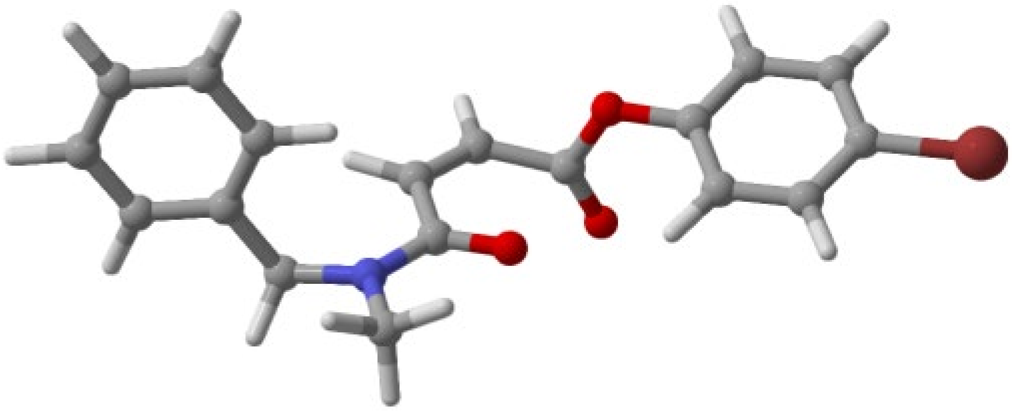

C	-2.17605000	1.30661400	-0.66811300
H	-3.15573900	1.73381000	-0.50245500
C	-1.11242800	2.09472700	-0.57712900
H	-1.22892900	3.14995400	-0.35060800
C	-2.02174500	-0.14576000	-0.93865800
C	0.29020700	1.65882300	-0.81208600
O	0.84093000	1.69327900	-1.87711800
O	-0.90236400	-0.65744900	-0.93029900
O	0.90188800	1.36360100	0.35168600
C	-3.03901000	-2.30389200	-1.27891000
H	-2.98990200	-2.60388700	-2.32999900
H	-2.14293200	-2.65186200	-0.77149100
H	-3.91768100	-2.76248600	-0.82340000
C	2.19744000	0.87174500	0.29742400
C	3.20981100	1.62542300	0.85939200
C	2.43821100	-0.37962600	-0.24293800
C	4.50109600	1.11857800	0.88353600
H	2.98993400	2.59990800	1.27759200
C	3.72726000	-0.88663900	-0.22409300
H	1.61861800	-0.94484400	-0.66870600
C	4.74482900	-0.13125400	0.33908600
H	5.30490200	1.69548000	1.32182600
H	3.93448000	-1.86324700	-0.64194000
Br	6.50506200	-0.82616900	0.36768600
N	-3.14091800	-0.86594100	-1.15169300
C	-4.45949700	-0.30112500	-1.33621300
H	-4.37521800	0.70647800	-1.74576800

H	-4.95918400	-0.88589700	-2.11328500
C	-5.31855200	-0.28637100	-0.09531600
C	-6.68883800	-0.07407400	-0.22668400
C	-4.78164400	-0.45025800	1.17474000
C	-7.50480200	-0.01839400	0.89152100
H	-7.11930500	0.04774300	-1.21580600
C	-5.59888900	-0.39890700	2.29737500
H	-3.71857500	-0.62611500	1.29286500
C	-6.96091300	-0.18092800	2.16010500
H	-8.56959500	0.14757000	0.77370200
H	-5.16567500	-0.53120000	3.28226800
H	-7.59849700	-0.14143100	3.03567900

Z-32

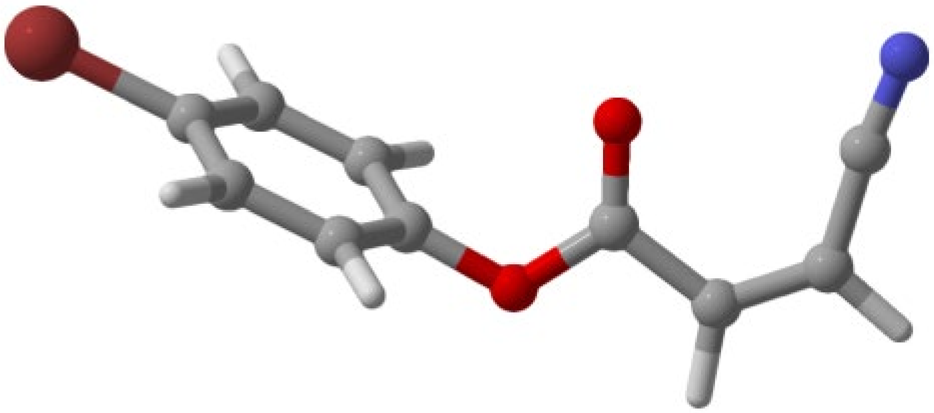

C	-5.10382300	-0.38428900	0.08676700
H	-6.03682600	-0.92243200	0.21520400
C	-3.94489600	-1.02596900	0.23329100
H	-3.94703300	-2.07959400	0.47922900
C	-5.25228700	0.99254500	-0.23431400
C	-2.63127500	-0.37258200	0.07632700
O	-2.44627200	0.78212900	-0.19394200
O	-1.65033200	-1.27403700	0.27950800
C	-0.33669800	-0.82996600	0.18439300
C	0.24014400	-0.16323200	1.24940900
C	0.37975500	-1.12980300	-0.95816300
C	1.56997500	0.21804300	1.16836400
H	-0.34253500	0.05442300	2.13576600

C	1.71120700	-0.75140400	-1.04207800
H	-0.09864000	-1.65649300	-1.77444700
C	2.29057000	-0.08020000	0.02234500
H	2.03765200	0.74023900	1.99264600
H	2.28719100	-0.97946800	-1.92922900
Br	4.10627500	0.43709400	-0.08826400
N	-5.48323200	2.09163500	-0.49035600

References

[1] G. Özüduru, T. Schubach, M. M. K. Boysen, *Org. Lett.* **2012**, *14*, 4990–4993.

[2] E. Brenna, F. G. Gatti, A. Manfredi, D. Monti, F. Parmeggiani, *Adv. Synth. Catal.* **2012**, *354*, 2859–2864.

[3] M. Miesch, F. Wendling, *European J. Org. Chem.* **2000**, *2000*, 3381–3391.

[4] G. Falsone, B. Spur, M. Erdmann, W. Peters, *Arch. Pharm. (Weinheim).* **1983**, *316*, 530–536.

[5] H.-J. Tsai, S.-C. Wu, *Phosphorus. Sulfur. Silicon Relat. Elem.* **2000**, *161*, 191–204.

[6] N. G. Turrini, R. C. Cioc, D. J. H. van der Niet, E. Ruijter, R. V. A. Orru, M. Hall, K. Faber, *Green Chem.* **2017**, *19*, 511–518.

[7] J. K. Crandall, W. W. Conover, *J. Org. Chem.* **1978**, *43*, 1323–1327.

[8] D. Barillier, R. Benhida, M. Vazeux, *Phosphorus. Sulfur. Silicon Relat. Elem.* **1993**, *78*, 83–95.

[9] T. Classen, M. Korpak, M. Schölzel, J. Pietruszka, *ACS Catal.* **2014**, *4*, 1321–1331.

[10] A. Martinez-Cuezva, C. Lopez-Leonardo, D. Bautista, M. Alajarin, J. Berna, *J. Am. Chem. Soc.* **2016**, *138*, 8726–8729.

[11] T. Kato, S. Matsuoka, M. Suzuki, *Chem. Commun.* **2015**, *51*, 13906–13909.

[12] F. Cateni, P. Nitti, S. Drioli, G. Procida, R. Menegazzi, M. Romano, *Med. Chem. Res.* **2021**, *30*, 913–924.

[13] S. Koul, D. H. G. Crout, W. Errington, J. Tax, *J. Chem. Soc. Perkin Trans. 1* **1995**, 2969–2988.

[14] S. Mawaziny, A. M. Lawny, *Phosphorus. Sulfur. Silicon Relat. Elem.* **2000**, *163*, 99–120.

[15] H. Yamaoka, N. Moriya, M. Ikunaka, *Org. Process Res. Dev.* **2004**, *8*, 931–938.

[16] Gaussian 16, Revision B.01, Frisch, M.J., Trucks, G.W., Schlegel, H.B., Scuseria, G.E., Robb, M.A., Cheeseman, J.R.; Scalmani, G.; Barone, V.; Petersson, G.A.; Nakatsuji, H.; Li, X.; Caricato, M.; Marenich, A.V.; Bloino, J., Janesko, B.G., Gomperts, R., Mennucci, B., Hratchian, H.P., Ortiz, J.V., Izmaylov, A.F., Sonnenberg, J.L., Williams-Young, D.,

Ding, F., Lipparini, F., Egidi, F., Goings, J., Peng, B., Petrone, A., Henderson, T., Ranasinghe, D., Zakrzewski, V.G., Gao, J., Rega, N., Zheng, G., Liang, W., Hada, M., Ehara, M., Toyota, K., Fukuda, R., Hasegawa, J., Ishida, M., Nakajima, T., Honda, Y., Kitao, O., Nakai, H., Vreven, T., Throssell, K., Montgomery Jr., J.A., Peralta, J.E., Ogliaro, F., Bearpark, M.J., Heyd, J.J., Brothers, E.N., Kudin, K.N., Staroverov, V.N., Keith, T.A., Kobayashi, R., Normand, J., Raghavachari, K., Rendell, A.P., Burant, J.C., Iyengar, S.S., Tomasi, J., Cossi, M., Millam, J.M., Klene, M., Adamo, C., Cammi, R., Ochterski, J.W., Martin, R.L., Morokuma, K., Farkas, O., Foresman, J.B., Fox, D.J. Gaussian, Inc., Wallingford CT (2016) GaussView 5.0. Wallingford, E.U.A.

[17] C. Adamo, V. Barone, *J. Chem. Phys.* **1999**, *110*, 6158–6170.

[18] S. Grimme, S. Ehrlich, L. Goerigk, *J. Comput. Chem.* **2011**, *32*, 1456–1465.

[19] F. Weigend, R. Ahlrichs, *Phys. Chem. Chem. Phys.* **2005**, *7*, 3297–3305.

[20] D. Rappoport, F. Furche, *J. Chem. Phys.* **2010**, *133*, 134105.

[21] B. P. Pritchard, D. Altarawy, B. Didier, T. D. Gibson, T. L. Windus, *J. Chem. Inf. Model.* **2019**, *59*, 4814–4820.

[22] A. D. Becke, *Phys. Rev. A* **1988**, *38*, 3098–3100.

[23] H. Iikura, T. Tsuneda, T. Yanai, K. Hirao, *J. Chem. Phys.* **2001**, *115*, 3540–3544.

[24] C. Y. Legault, **2020**.

[25] and F. W. E. D. Glendening, A. E. Reed, J. E. Carpenter, **n.d.**

[26] T. Lu, F. Chen, *J. Comput. Chem.* **2012**, *33*, 580–592.

NMR-Spectra of Key Compounds

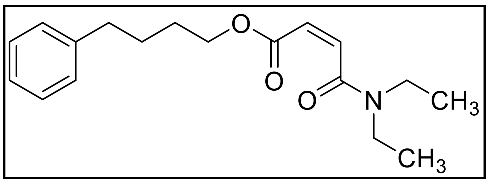

Z-1

^{1}H NMR (500 MHz, $CDCl_3$)

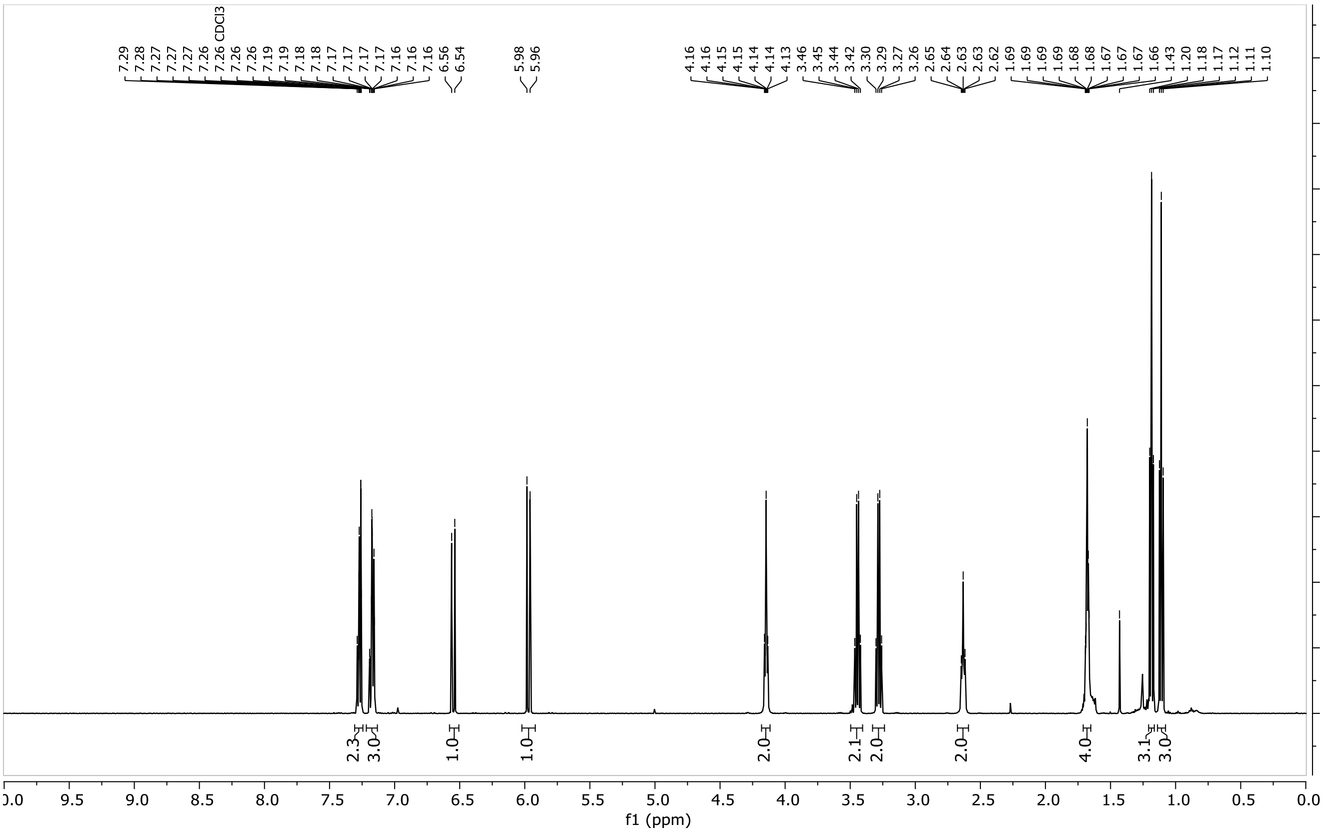

^{13}C NMR (126 MHz, $CDCl_3$)

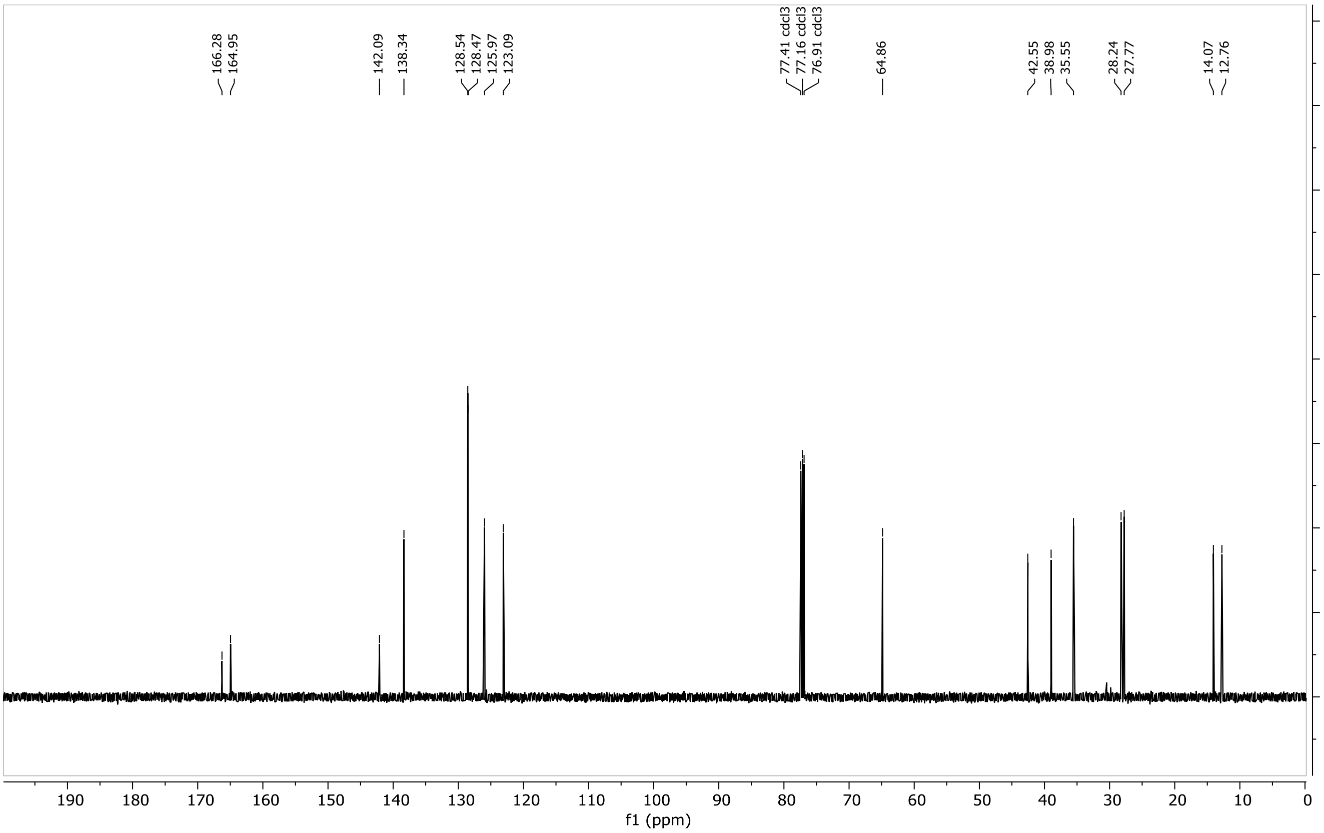

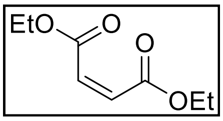

Z-2

^{1}H NMR (400 MHz, $CDCl_3$)

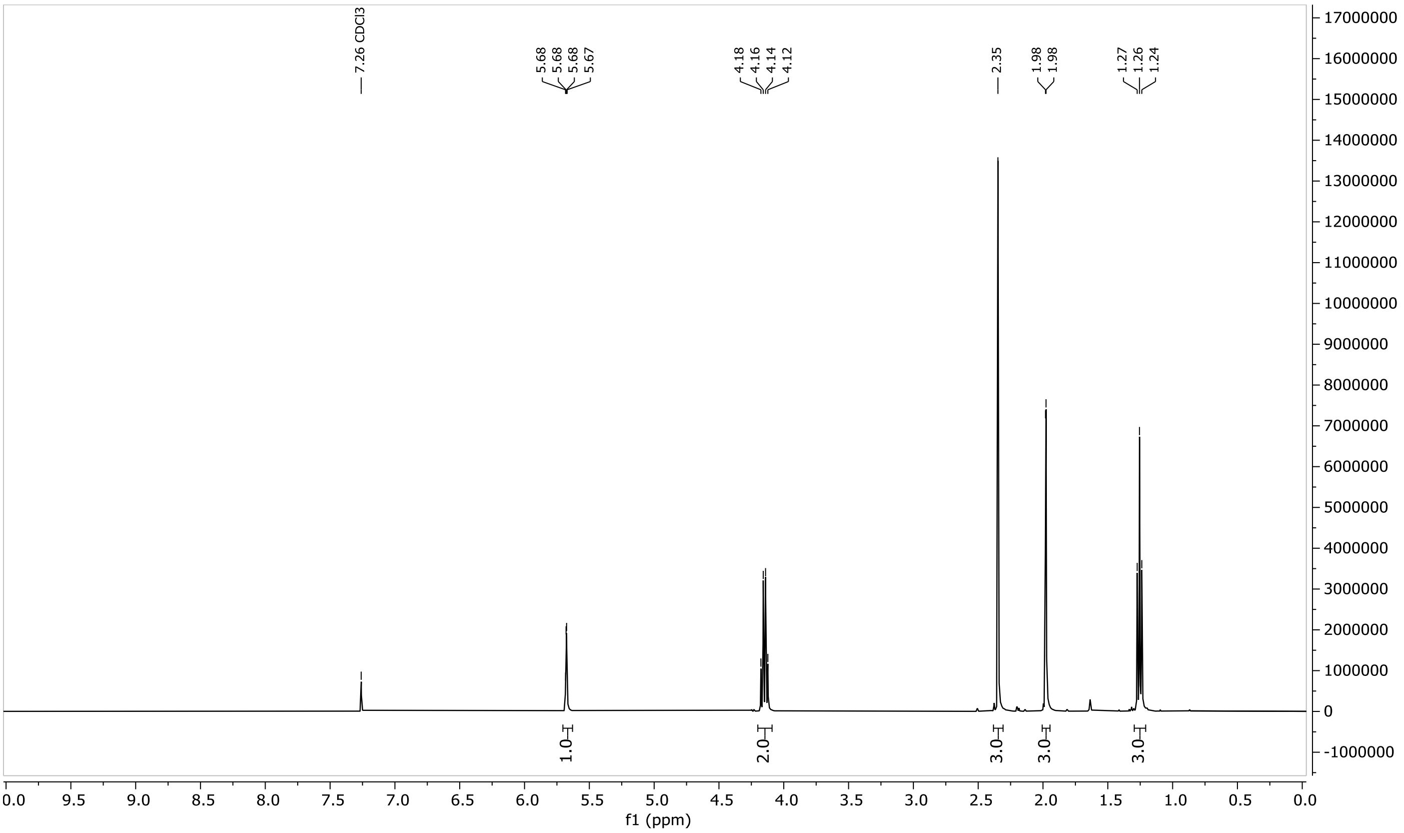

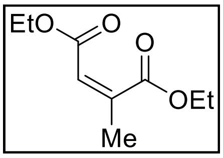

Z-5

^{1}H NMR (300 MHz, $CDCl_3$)

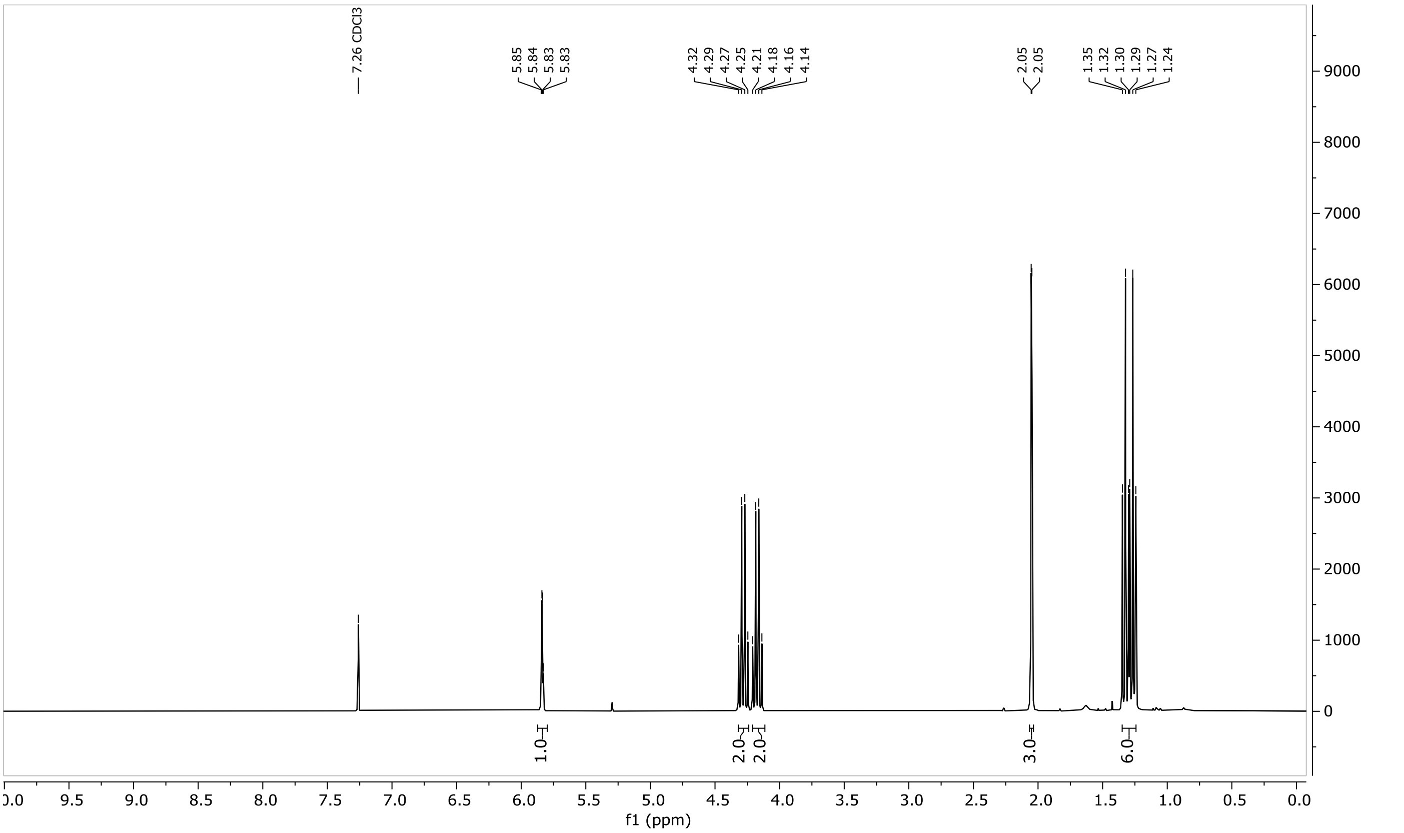

Z-3

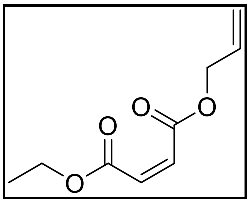

^{1}H NMR (500 MHz, $CDCl_3$)

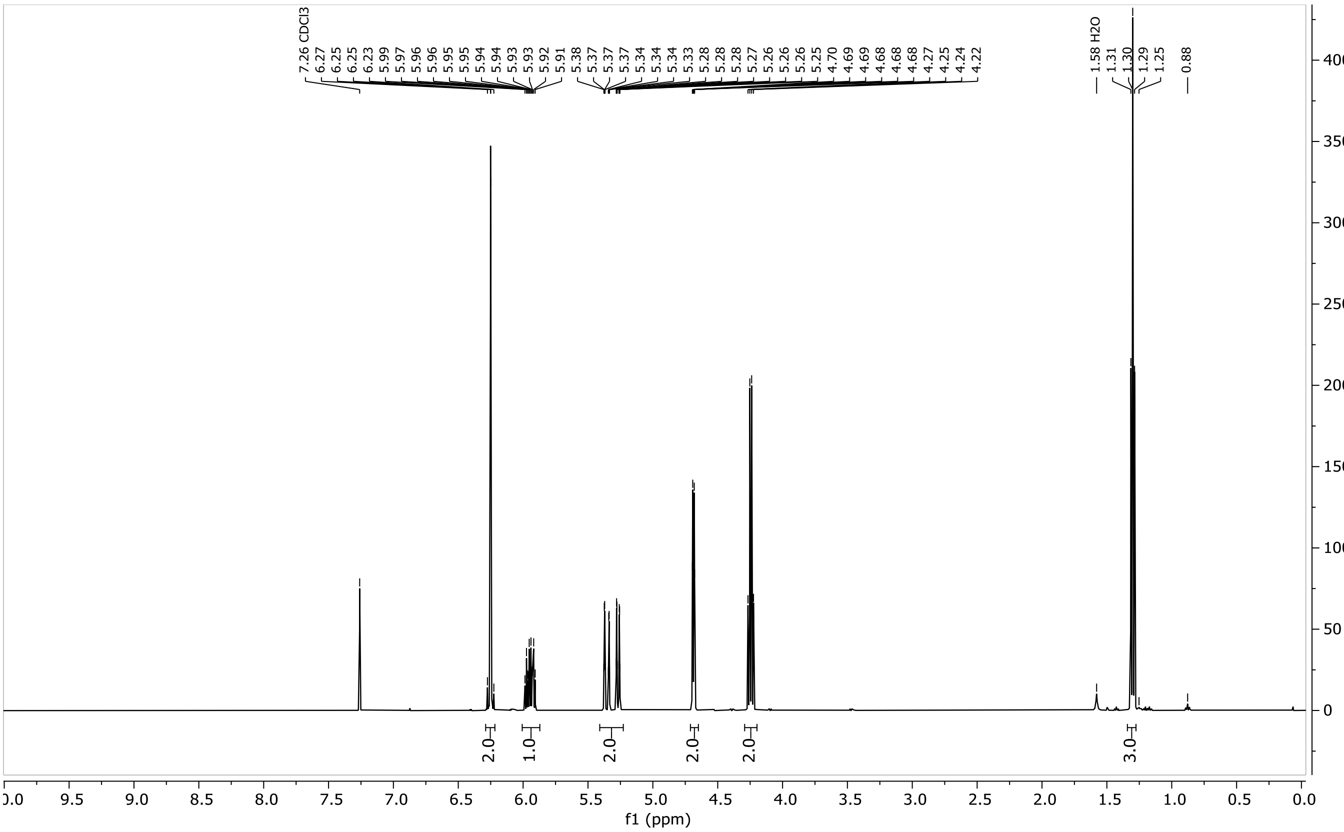

^{13}C NMR (126 MHz, $CDCl_3$)

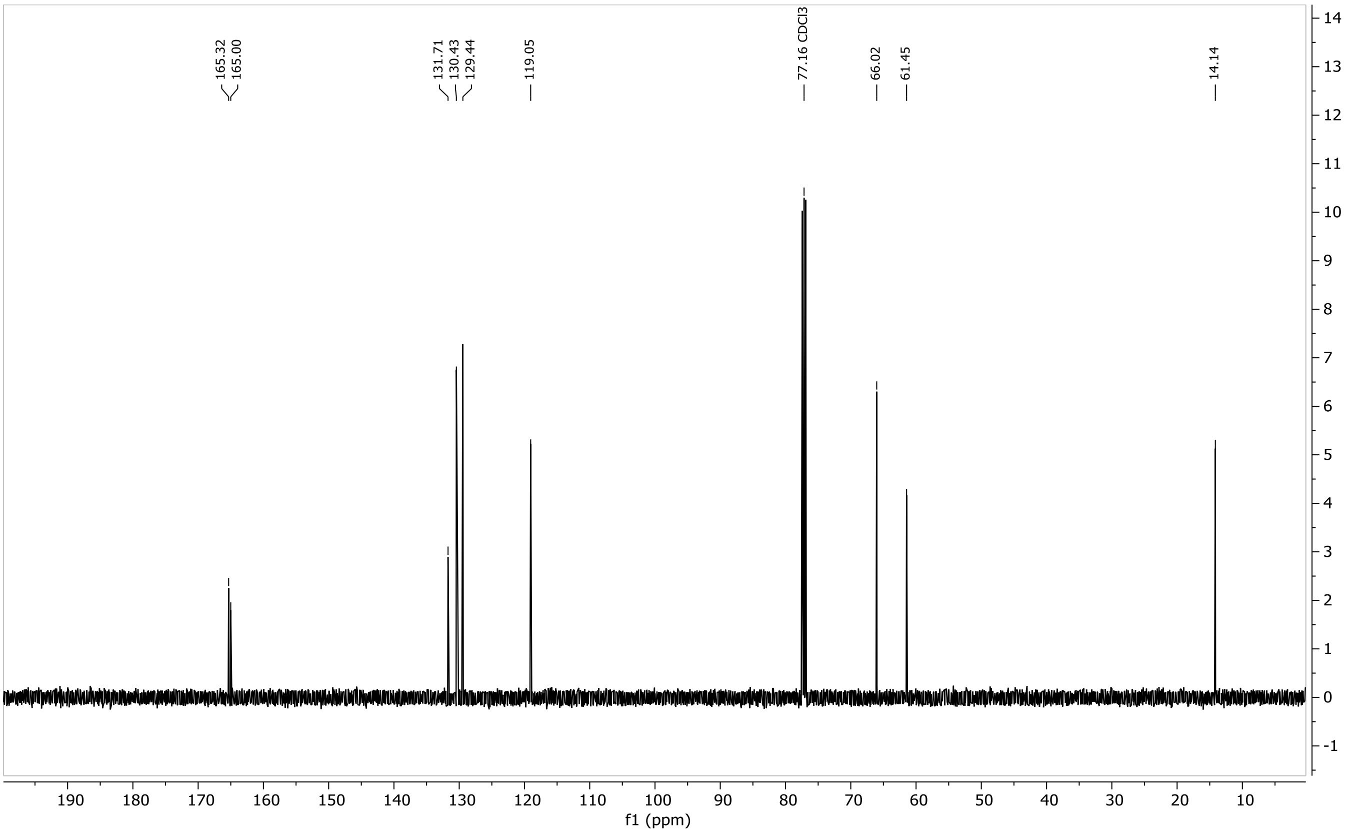

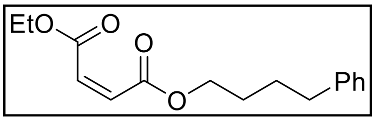

Z-4

^{1}H NMR (400 MHz, $CDCl_3$)

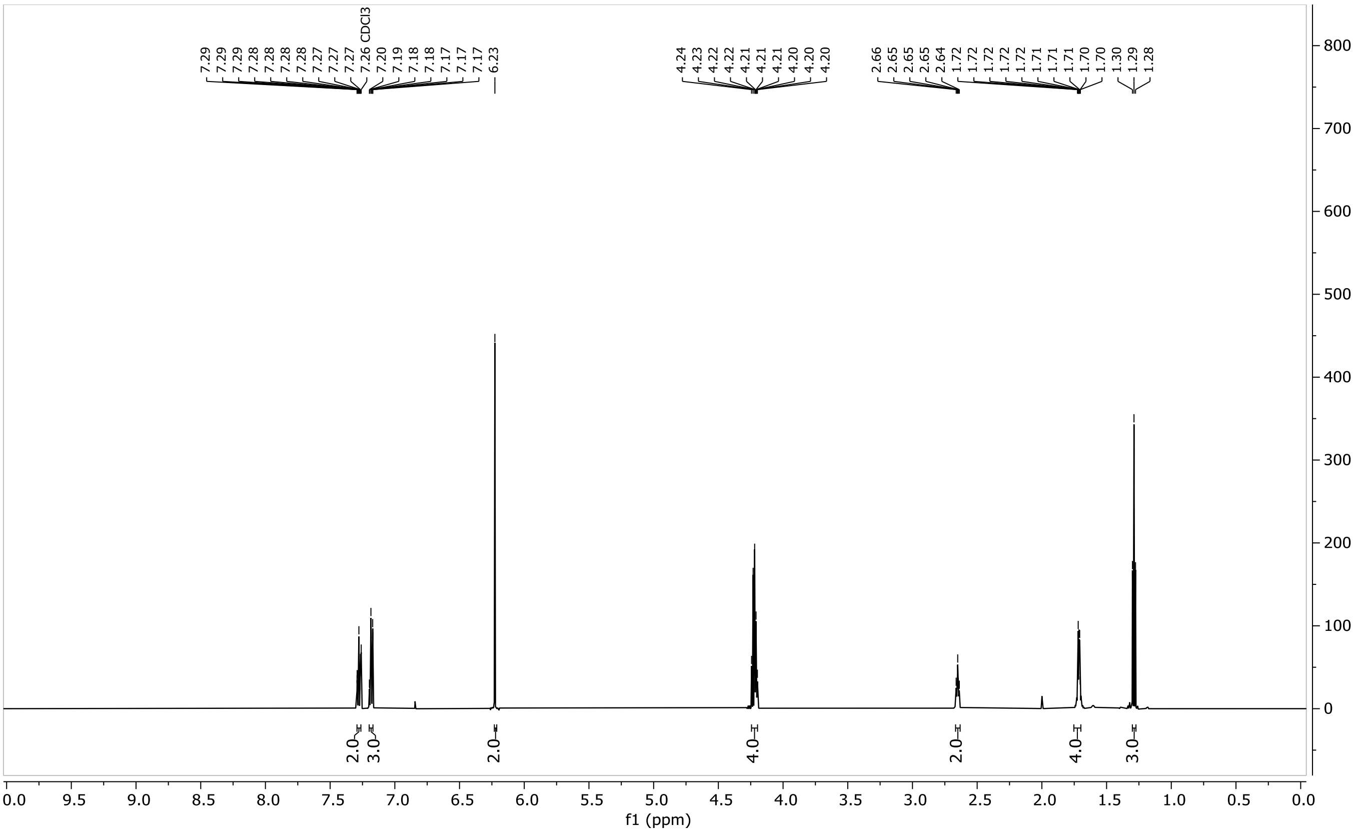

^{13}C NMR (100 MHz, $CDCl_3$)

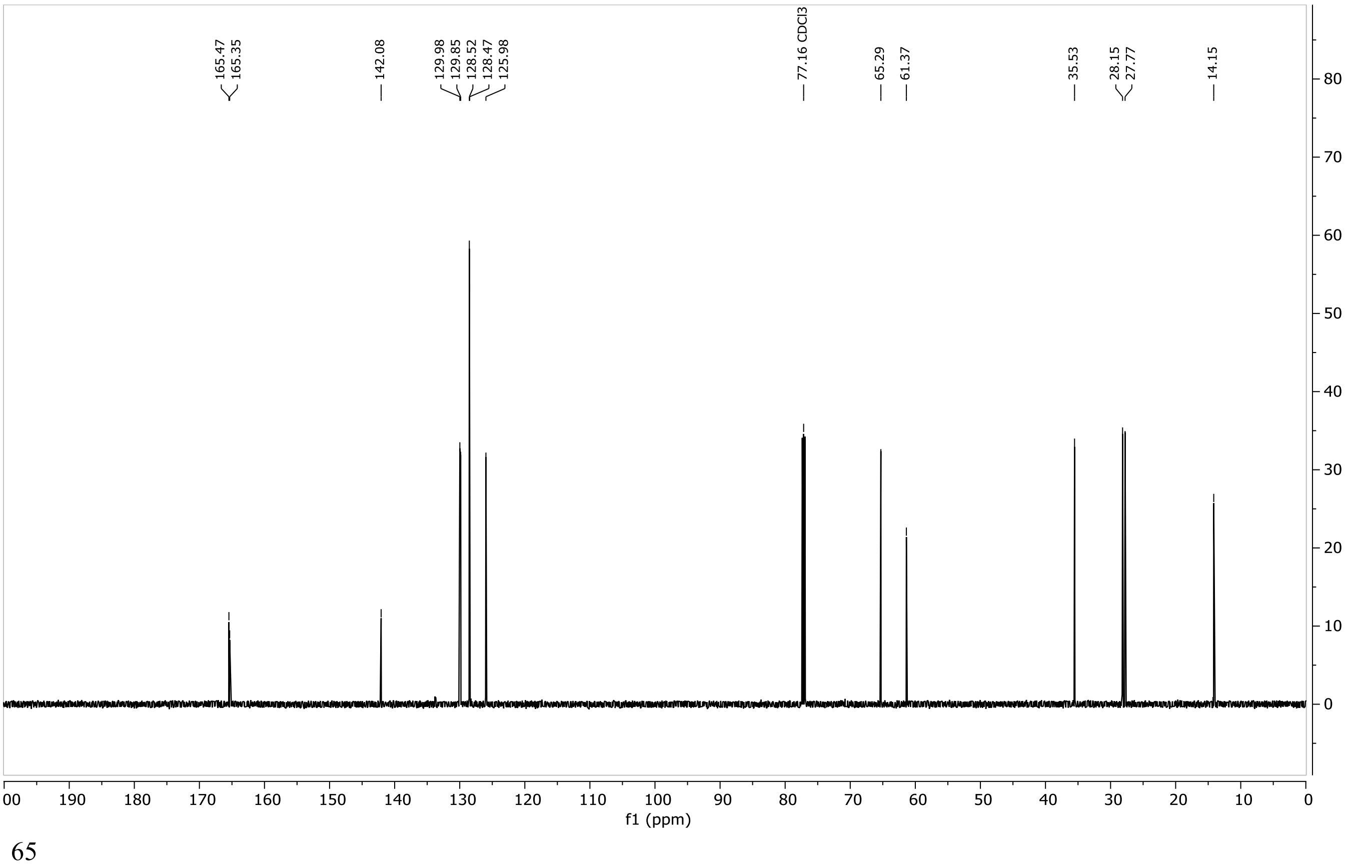

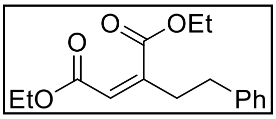

Z-6

^{1}H NMR (600 MHz, $CDCl_3$)

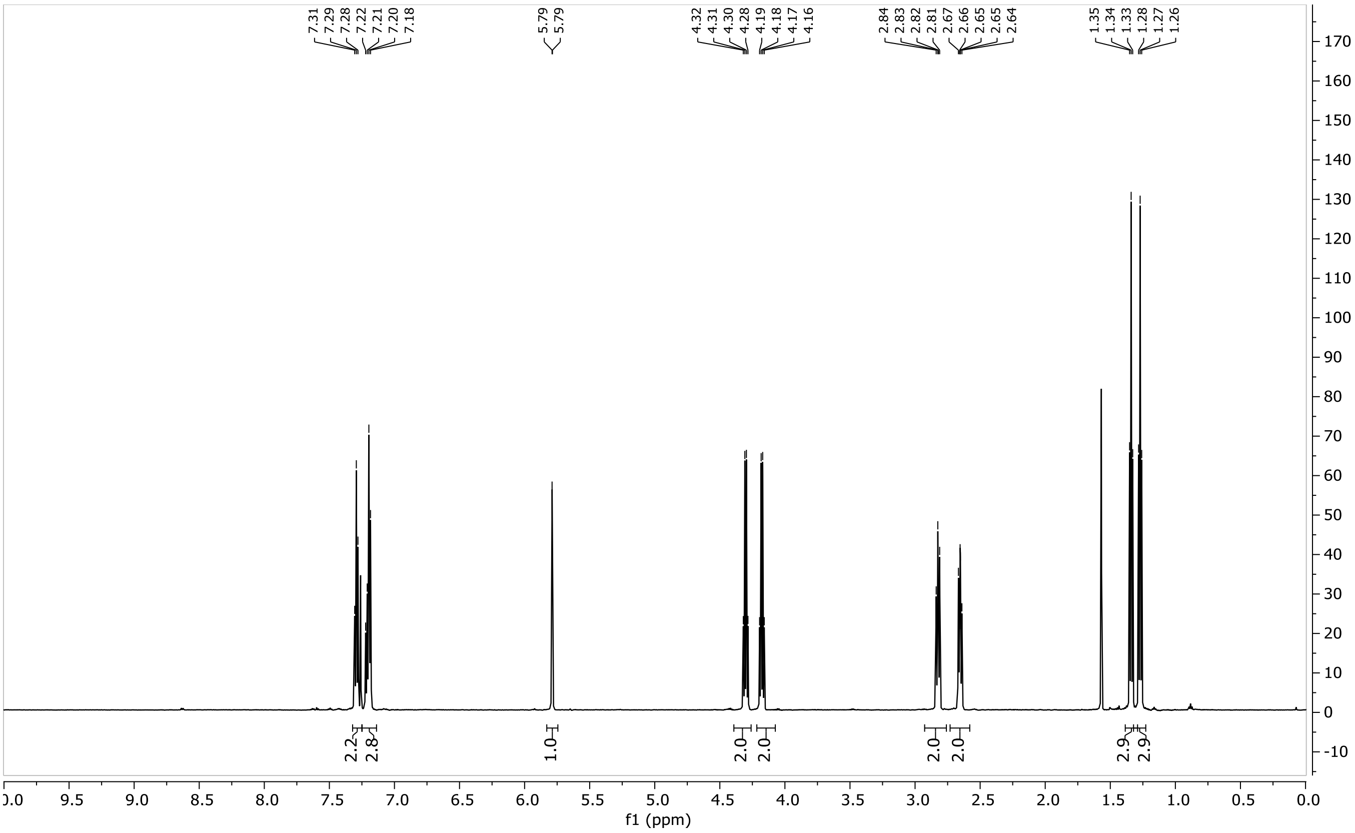

^{13}C NMR (151 MHz, $CDCl_3$)

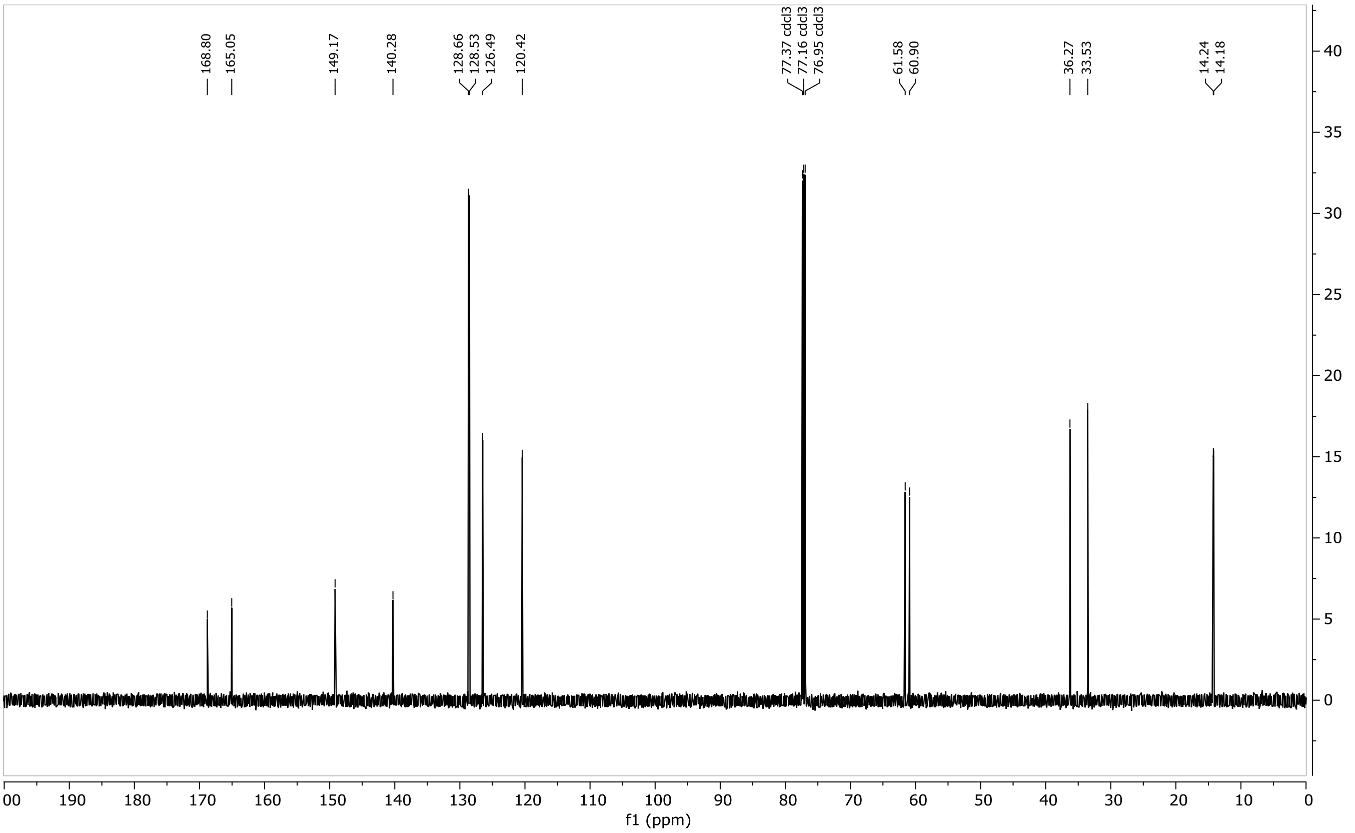

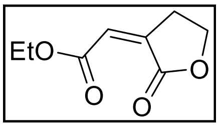

Z-7

^{1}H NMR (600 MHz, $CDCl_3$)

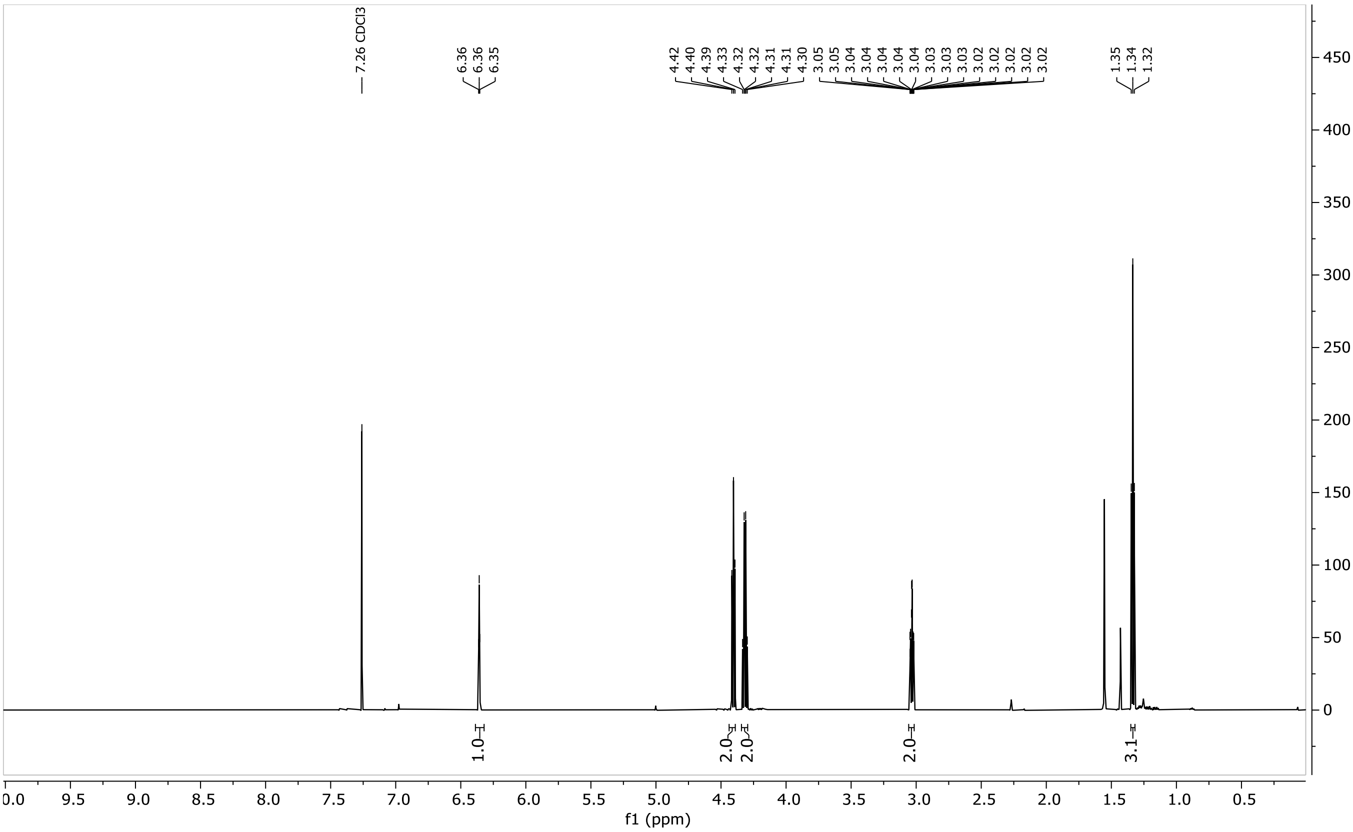

^{13}C NMR (151 MHz, $CDCl_3$)

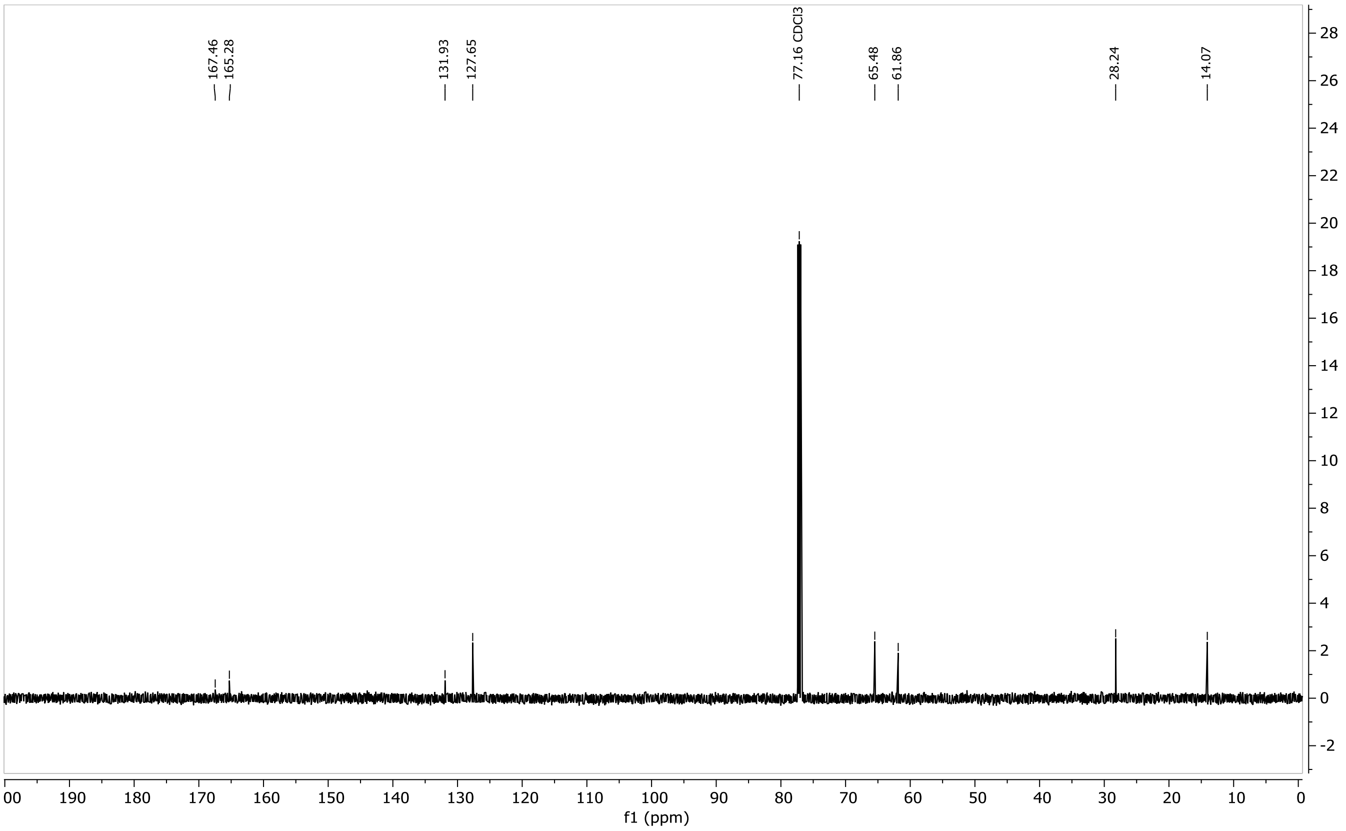

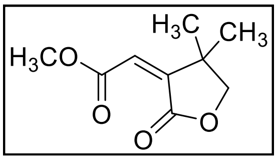

Z-8

^{1}H NMR (600 MHz, $CDCl_3$)

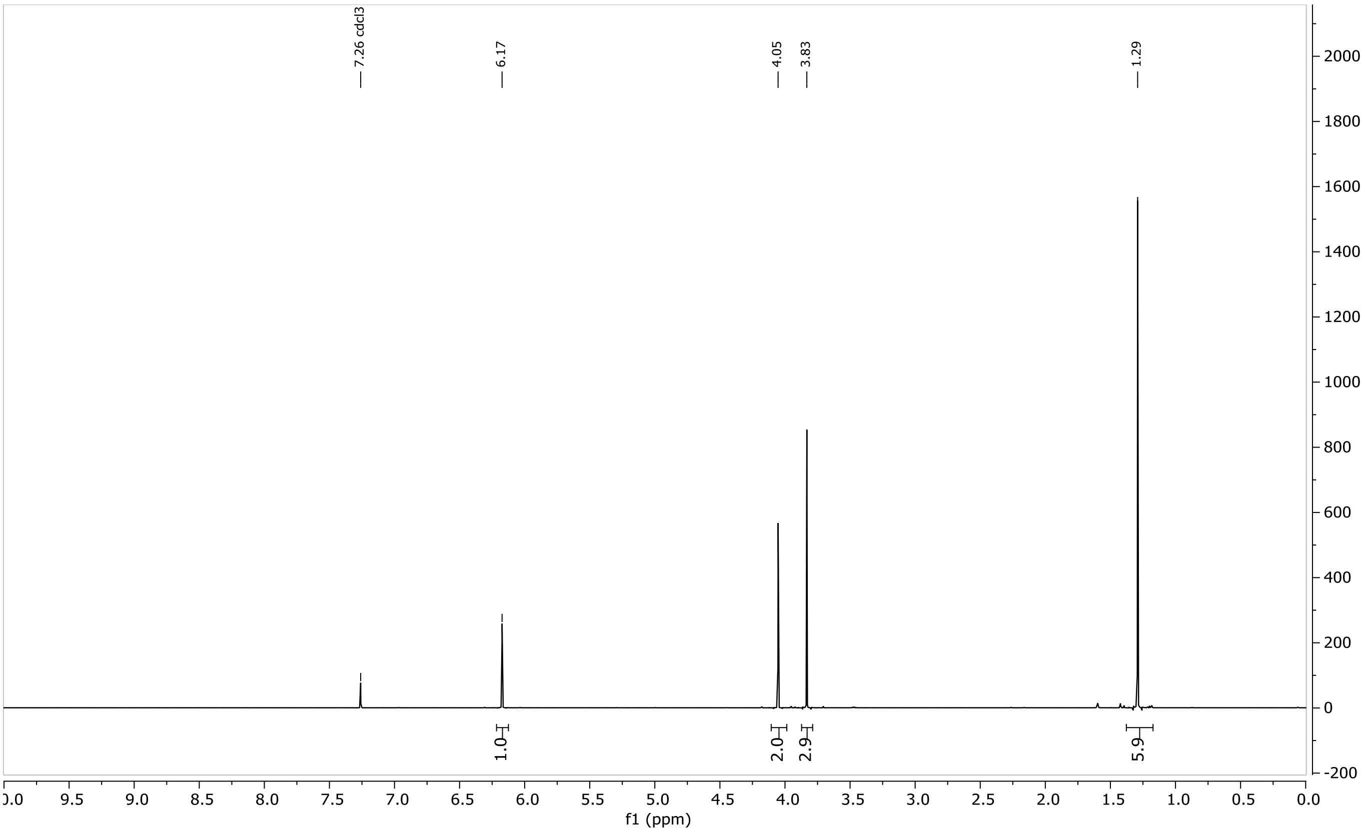

^{13}C NMR (151 MHz, $CDCl_3$)

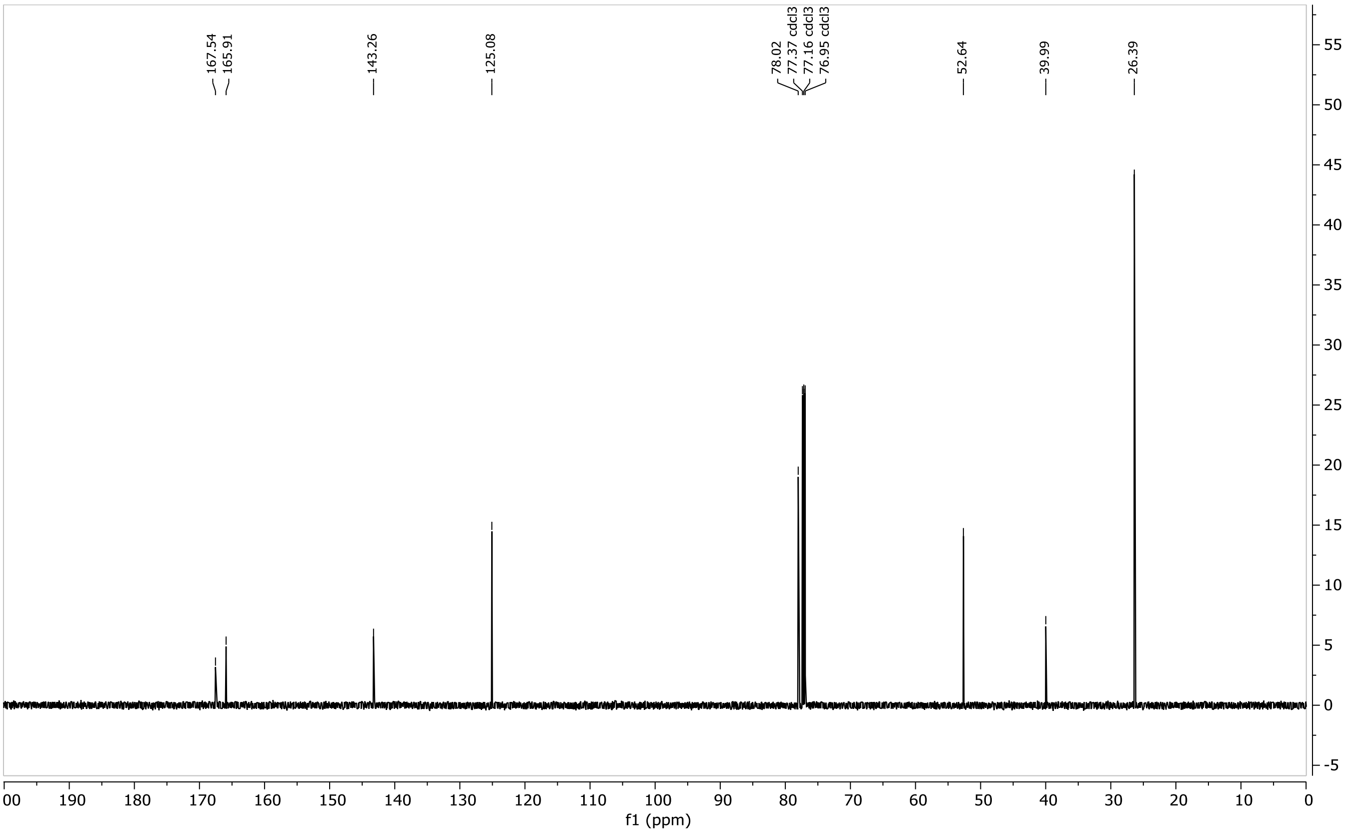

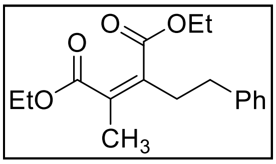

Z-9

^{1}H NMR (400 MHz, $CDCl_3$)

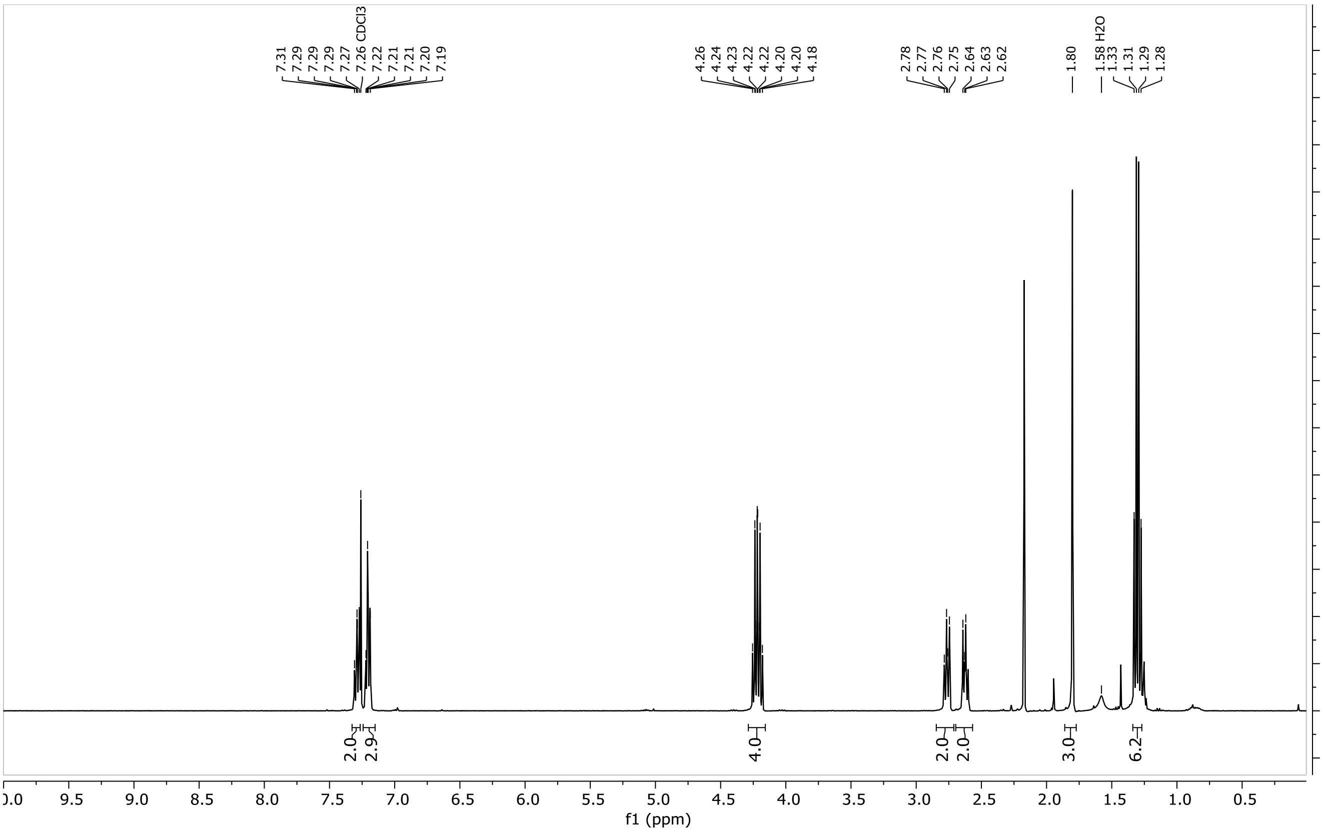

^{13}C NMR (100 MHz, $CDCl_3$)

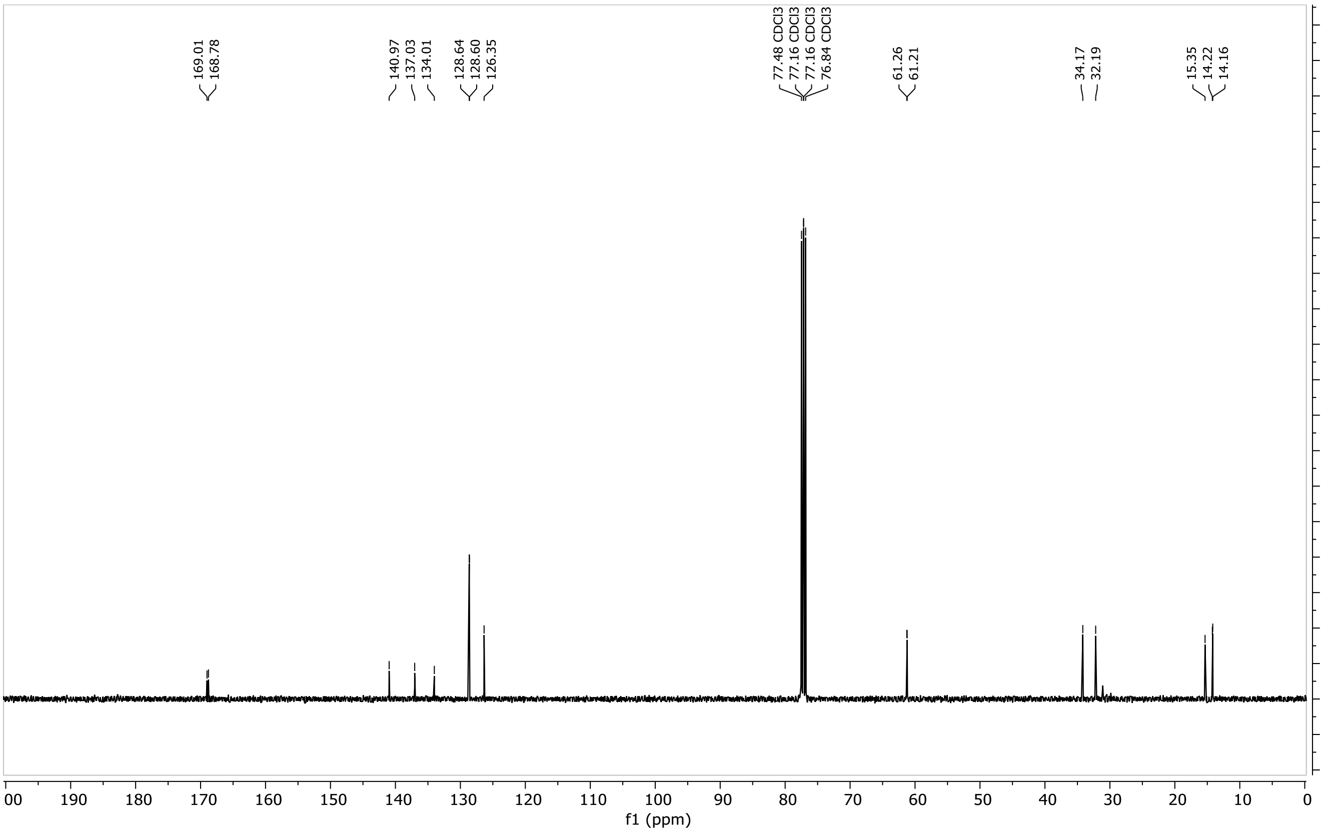

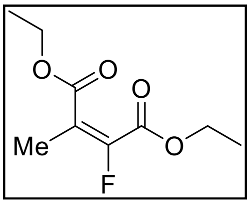

E-10

^{1}H NMR (400 MHz, $CDCl_3$)

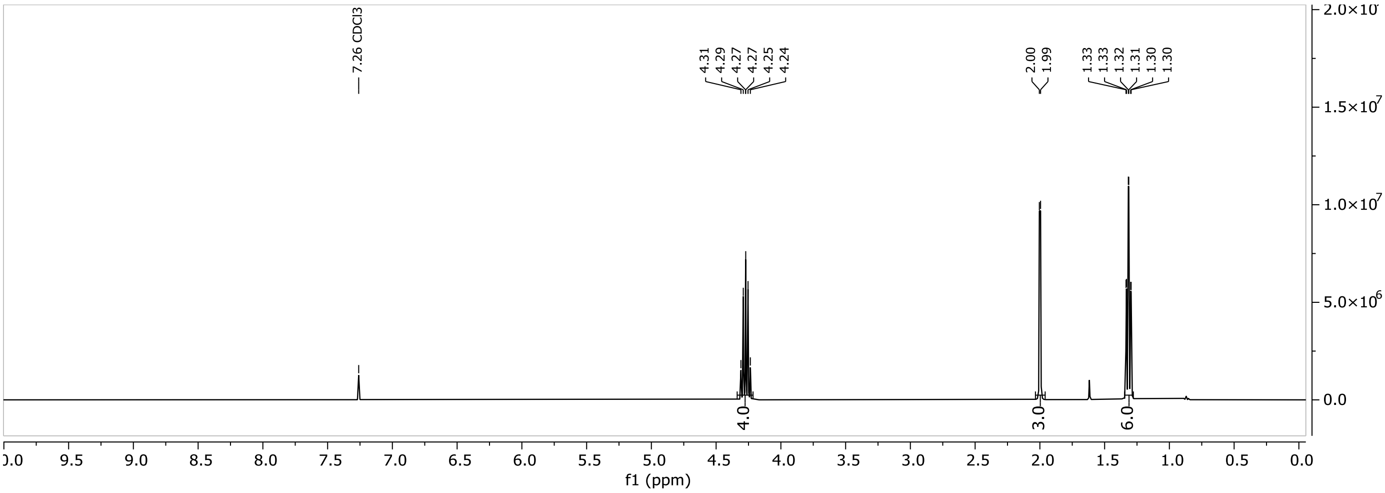

^{13}C-{^{19}F} NMR (151 MHz, $CDCl_3$)

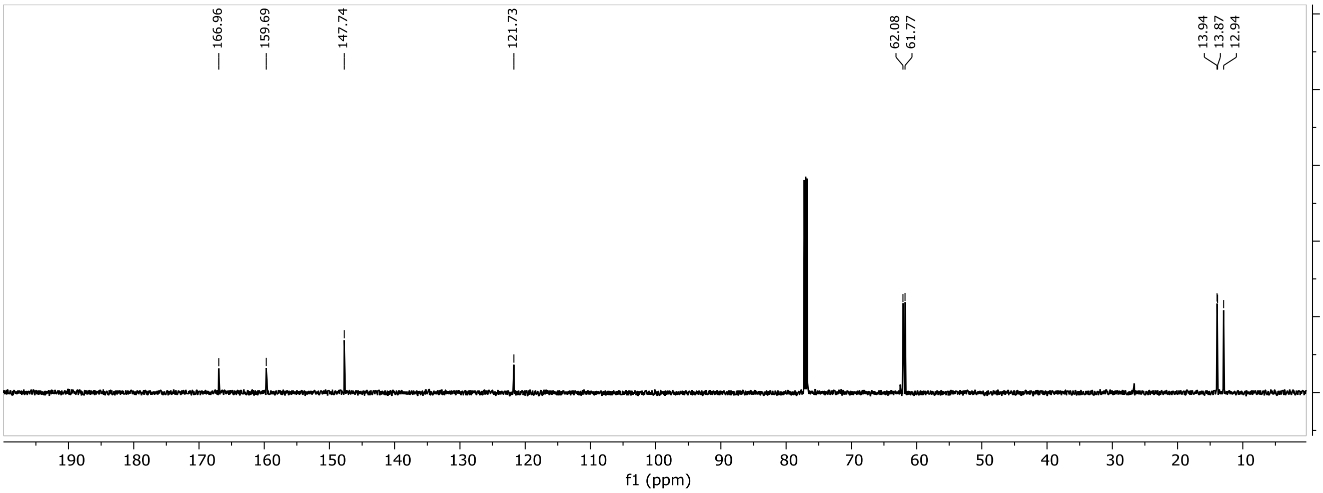

^{19}F NMR (376 MHz, $CDCl_3$)

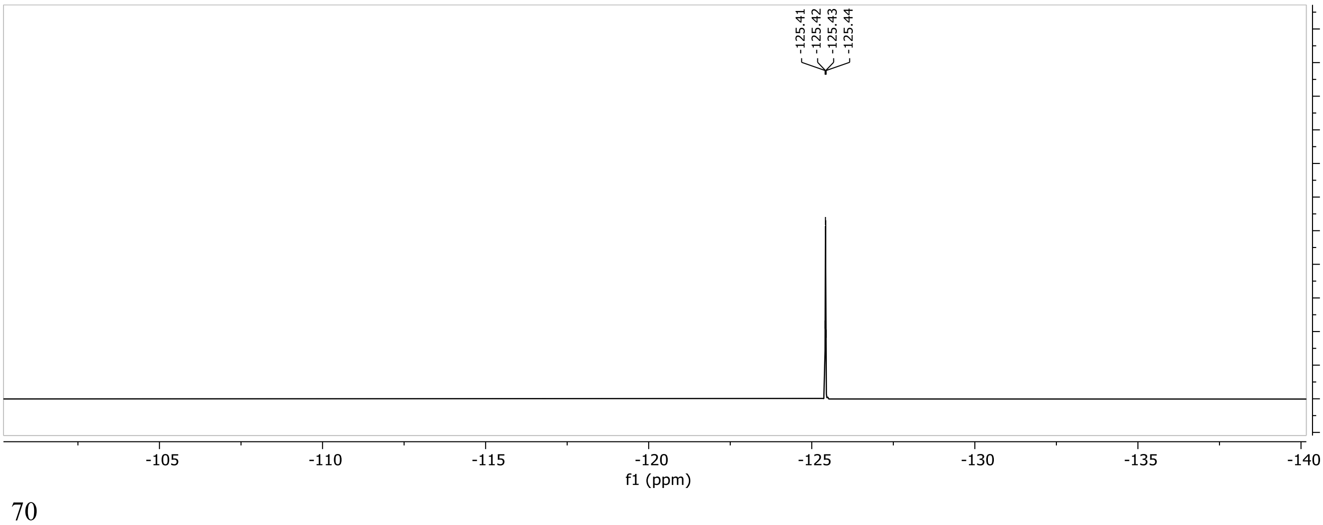

Z-11

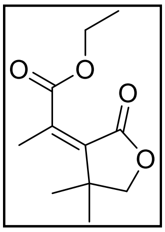

^{1}H NMR (600 MHz, $CDCl_3$)

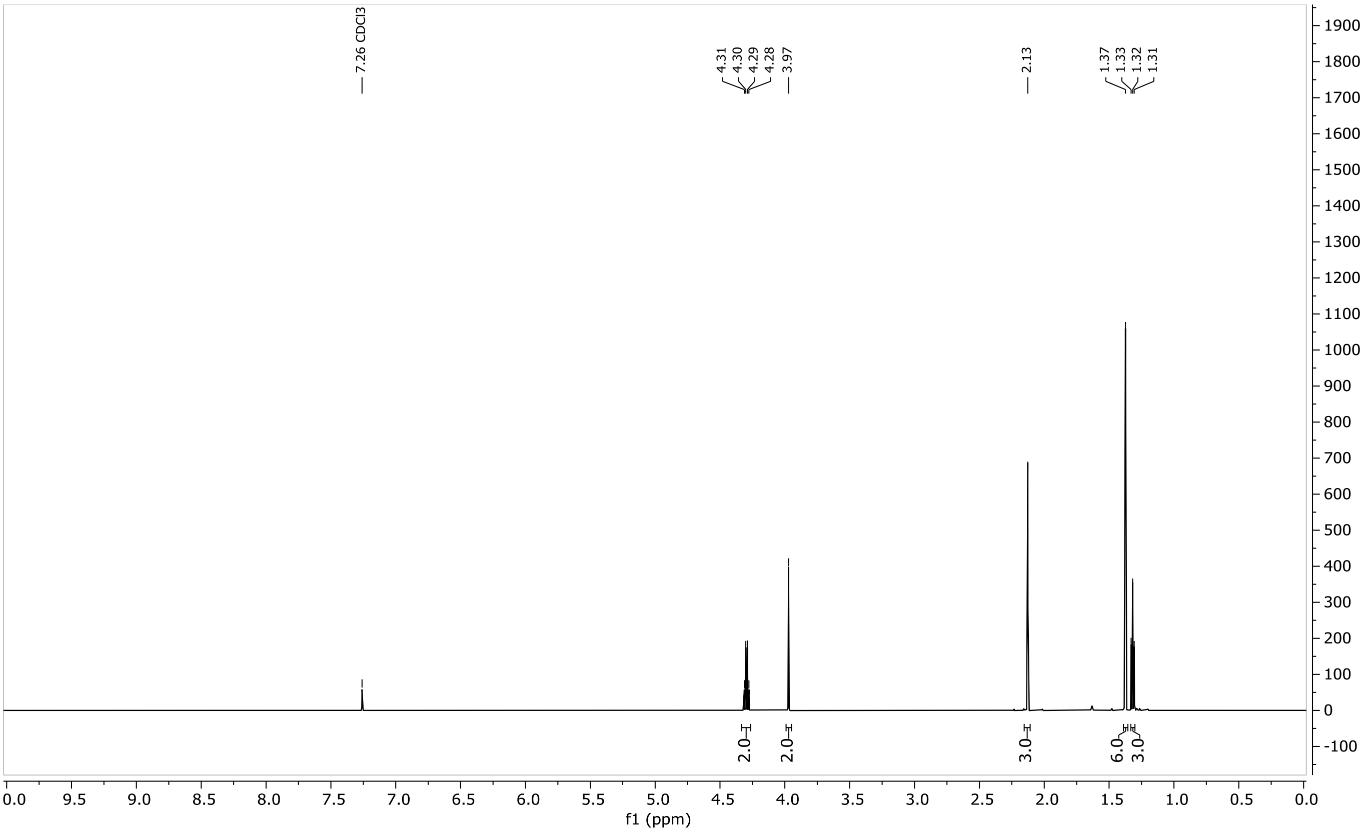

^{13}C NMR (151 MHz, $CDCl_3$)

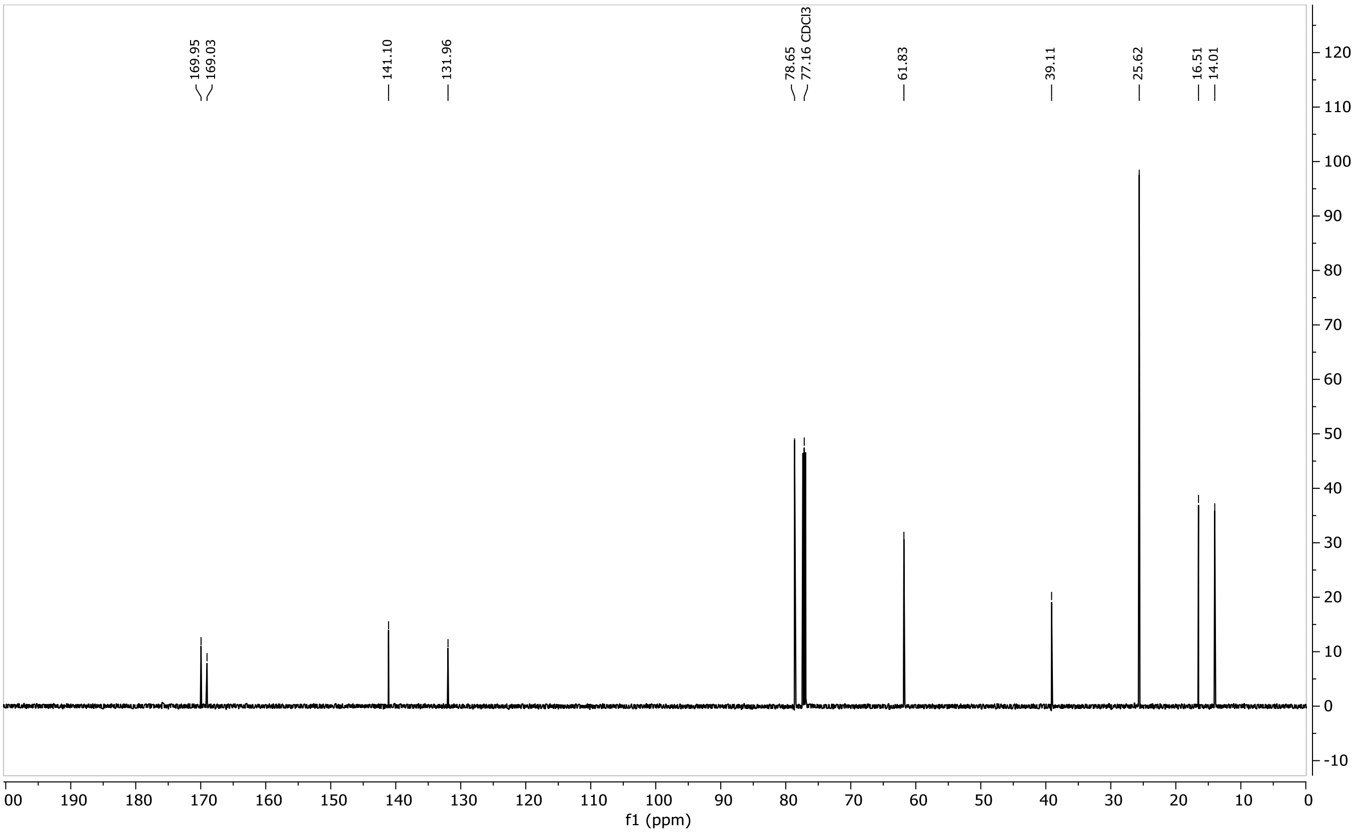

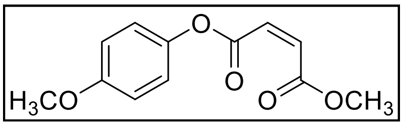

Z-12

^{1}H NMR (500 MHz, $CDCl_3$)

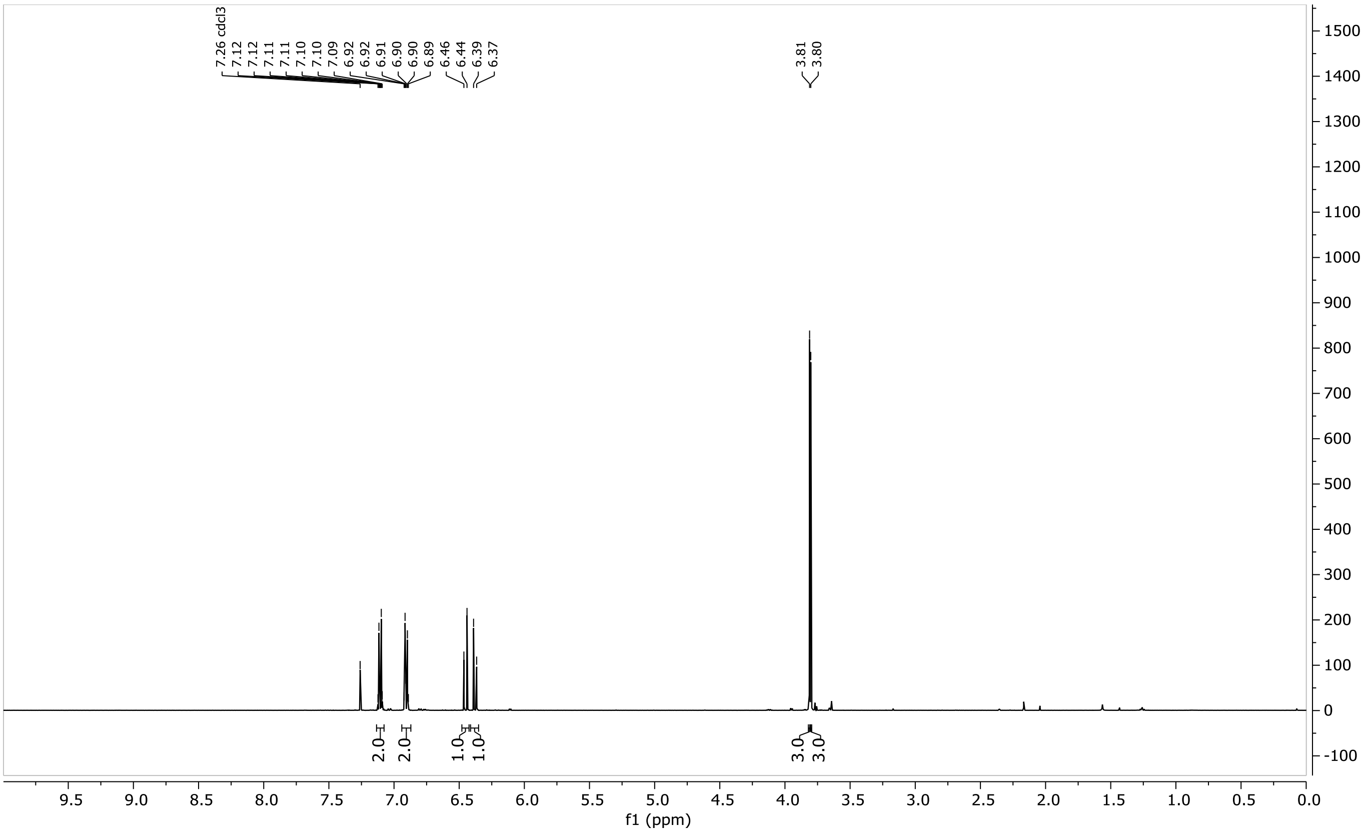

^{13}C NMR (126 MHz, $CDCl_3$)

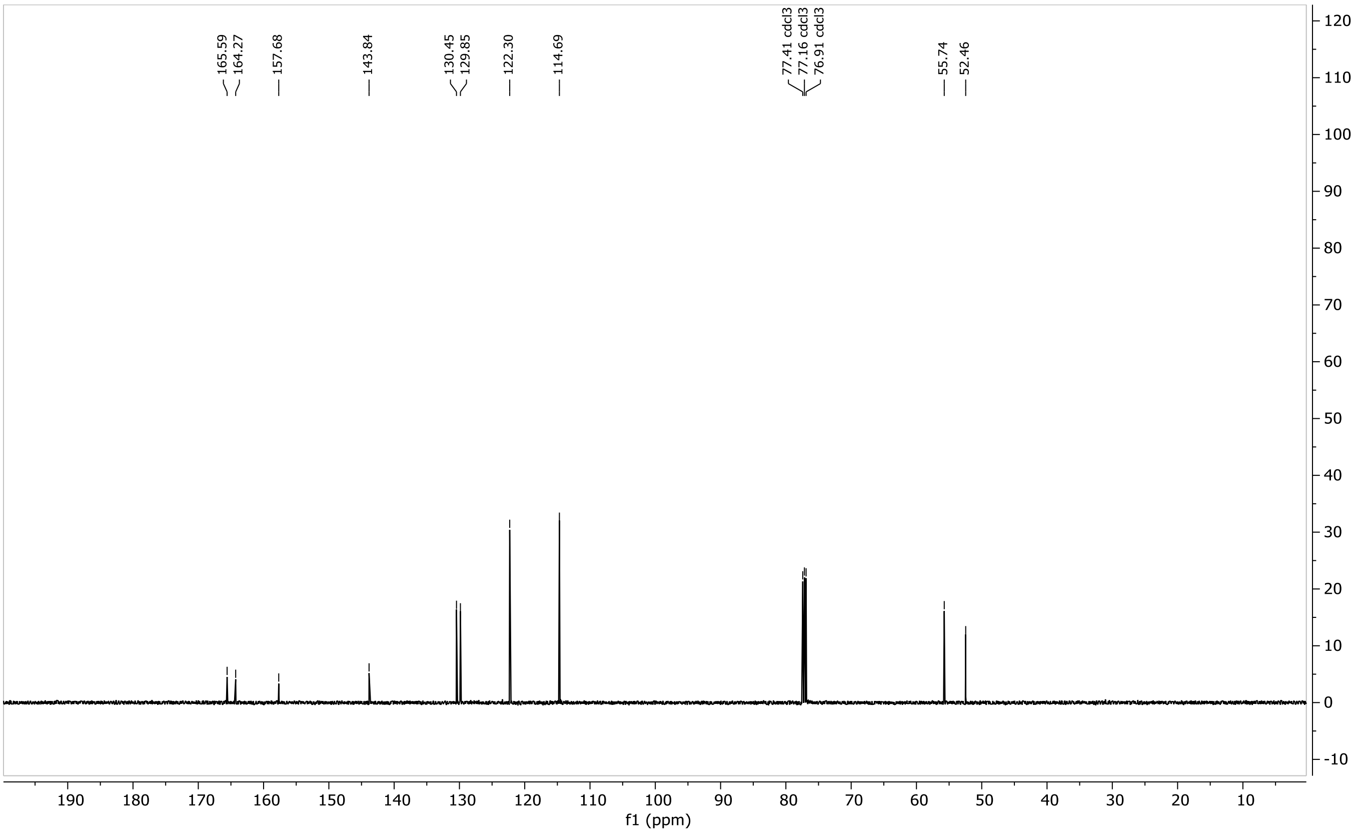

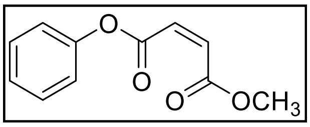

Z-13

^{1}H NMR (500 MHz, $CDCl_3$)

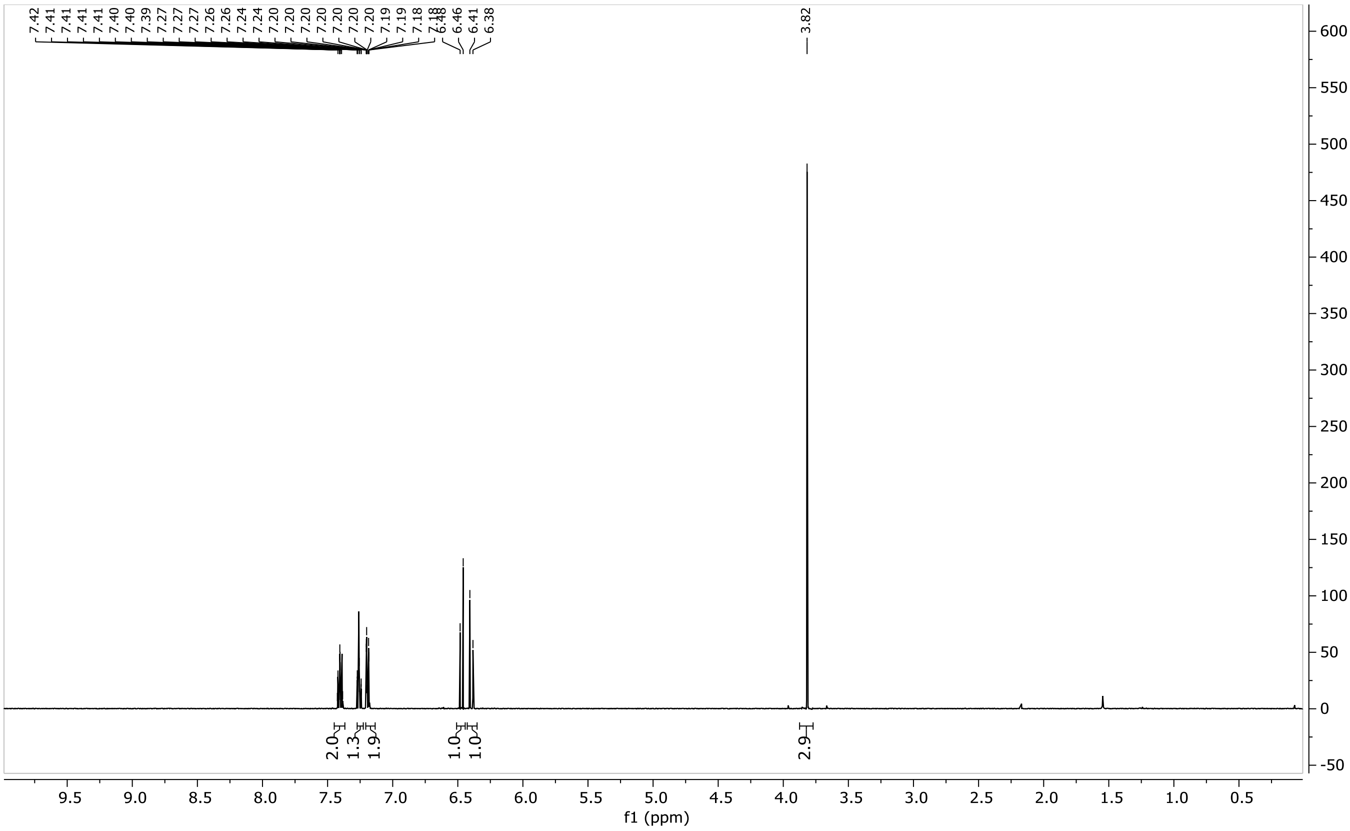

^{13}C NMR (126 MHz, $CDCl_3$)

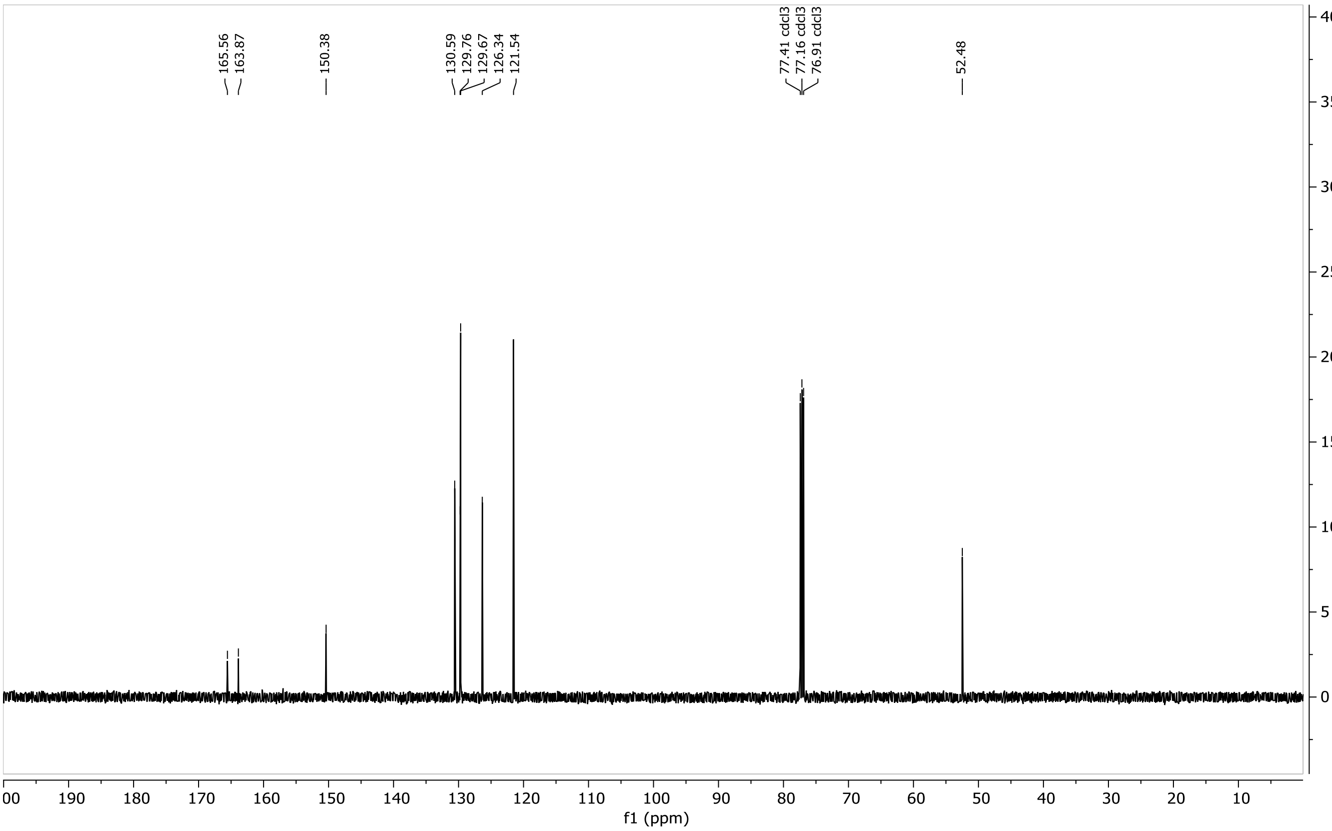

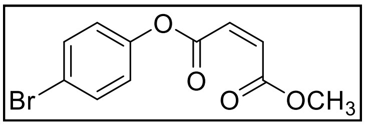

Z-14

^{1}H NMR (500 MHz, $CDCl_3$)

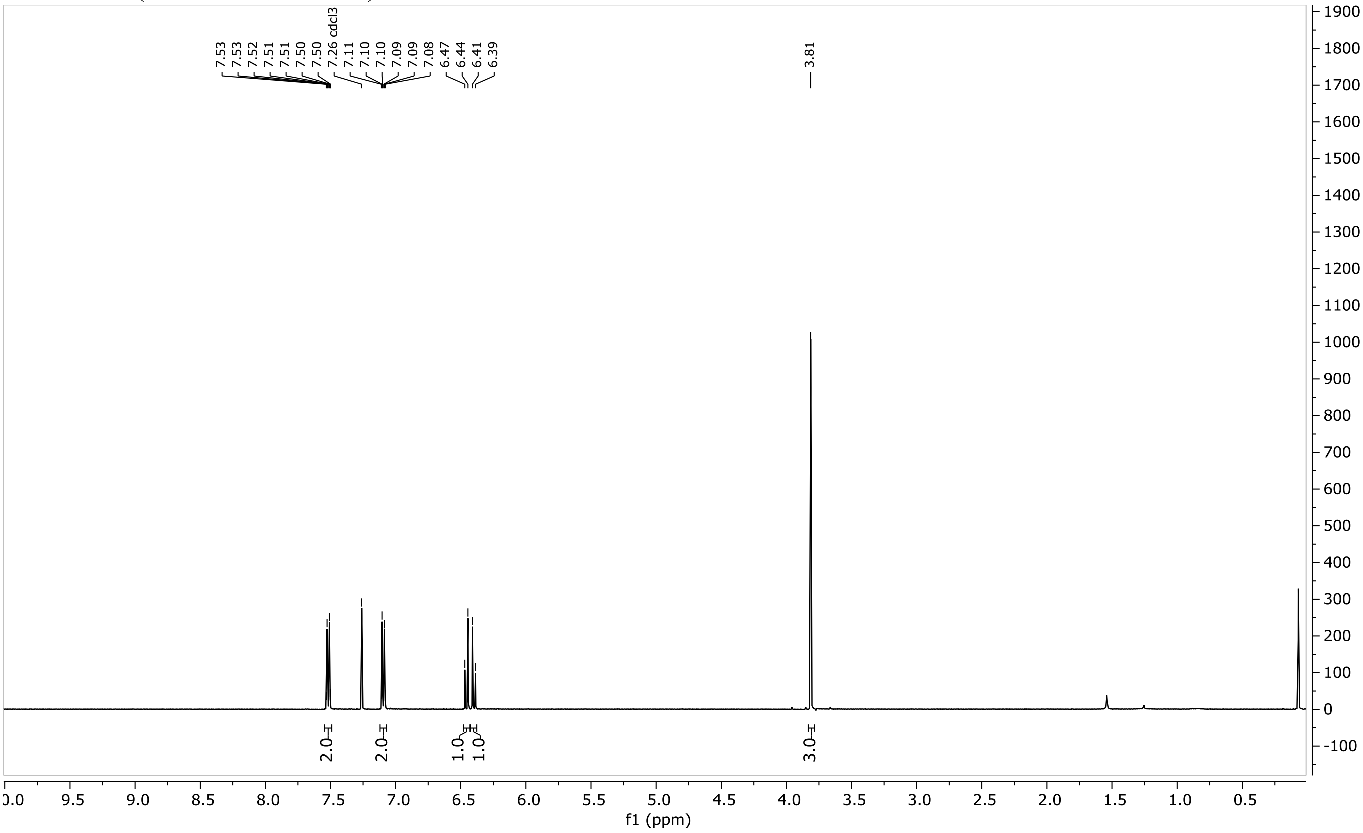

^{13}C NMR (126 MHz, $CDCl_3$)

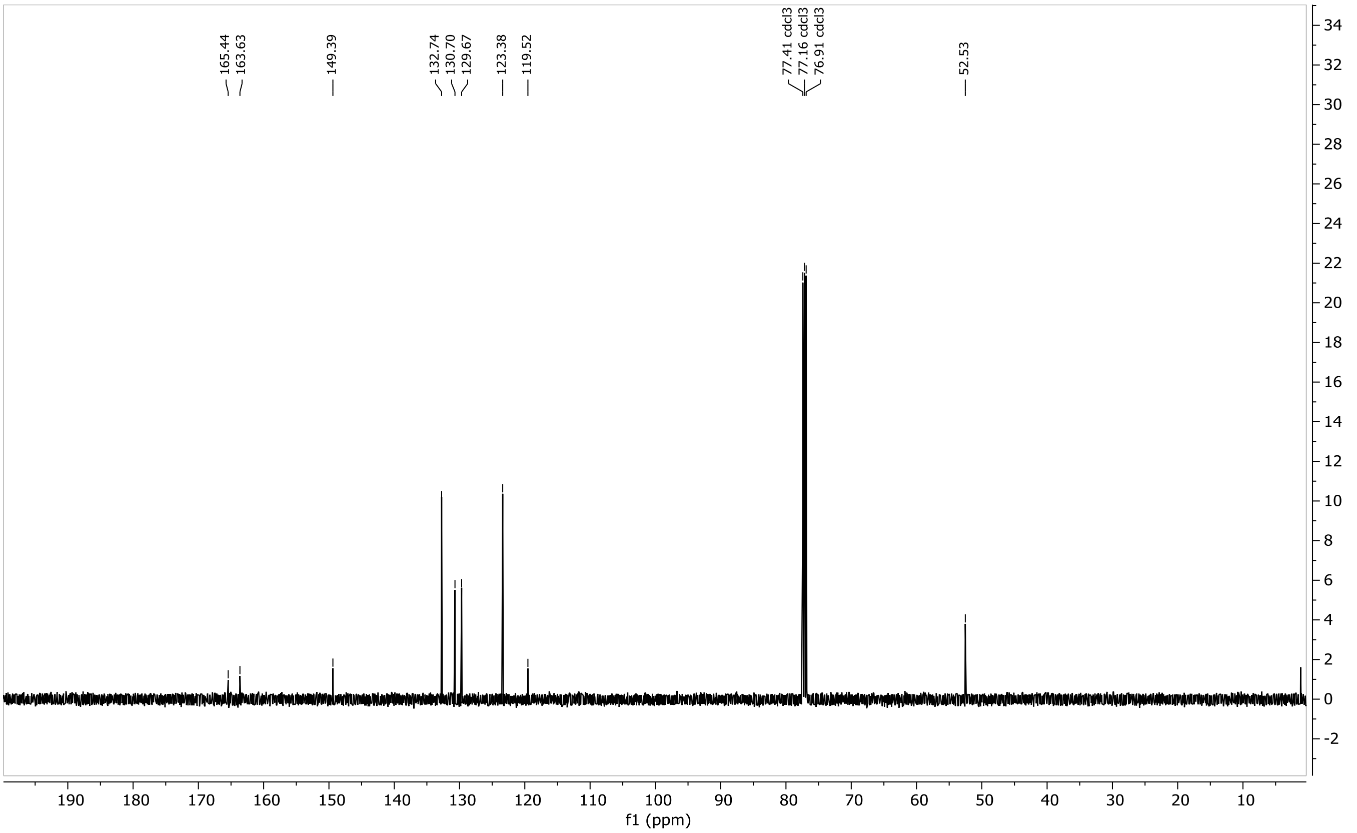

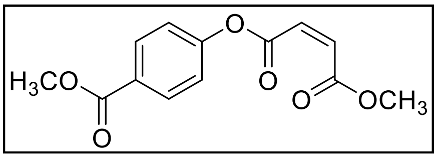

Z-15

^{1}H NMR (600 MHz, $CDCl_3$)

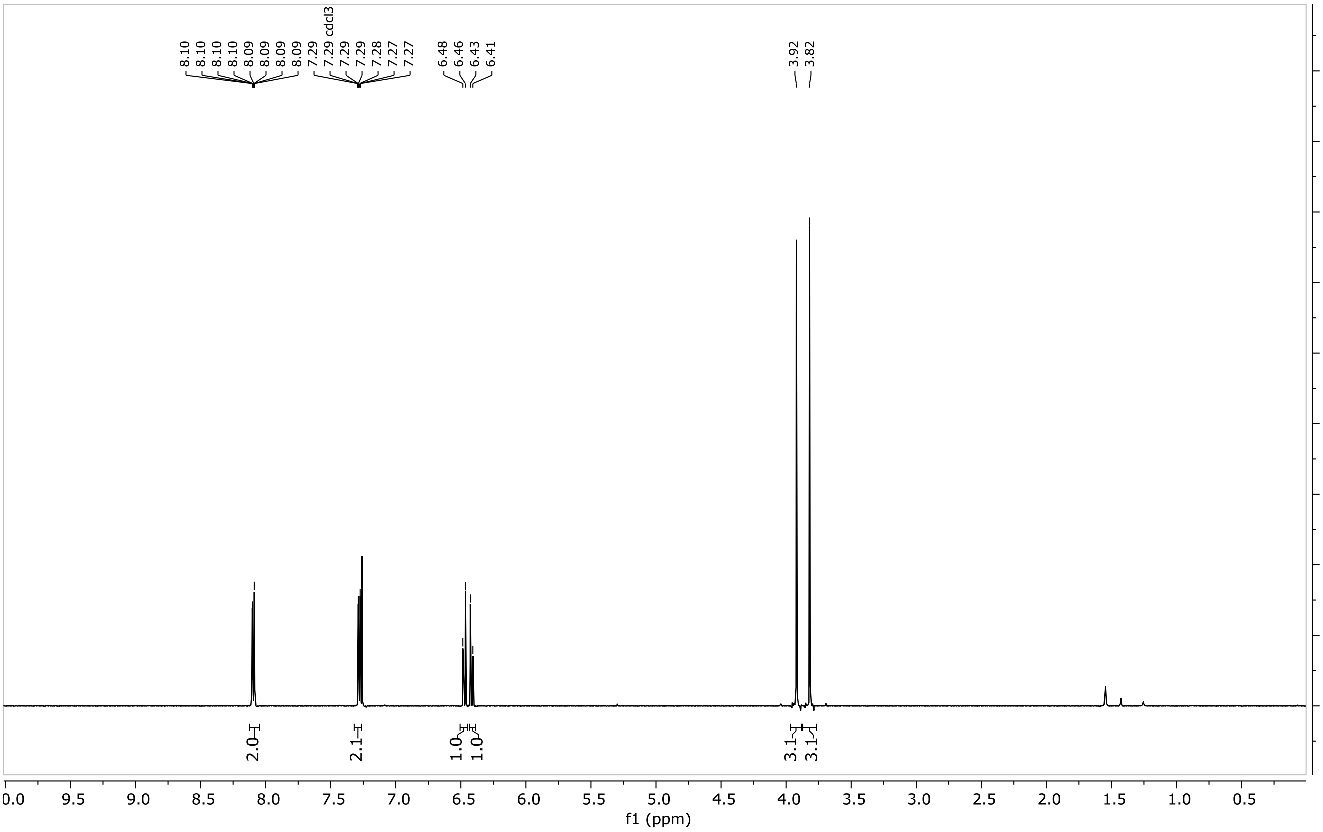

^{13}C NMR (151 MHz, $CDCl_3$)

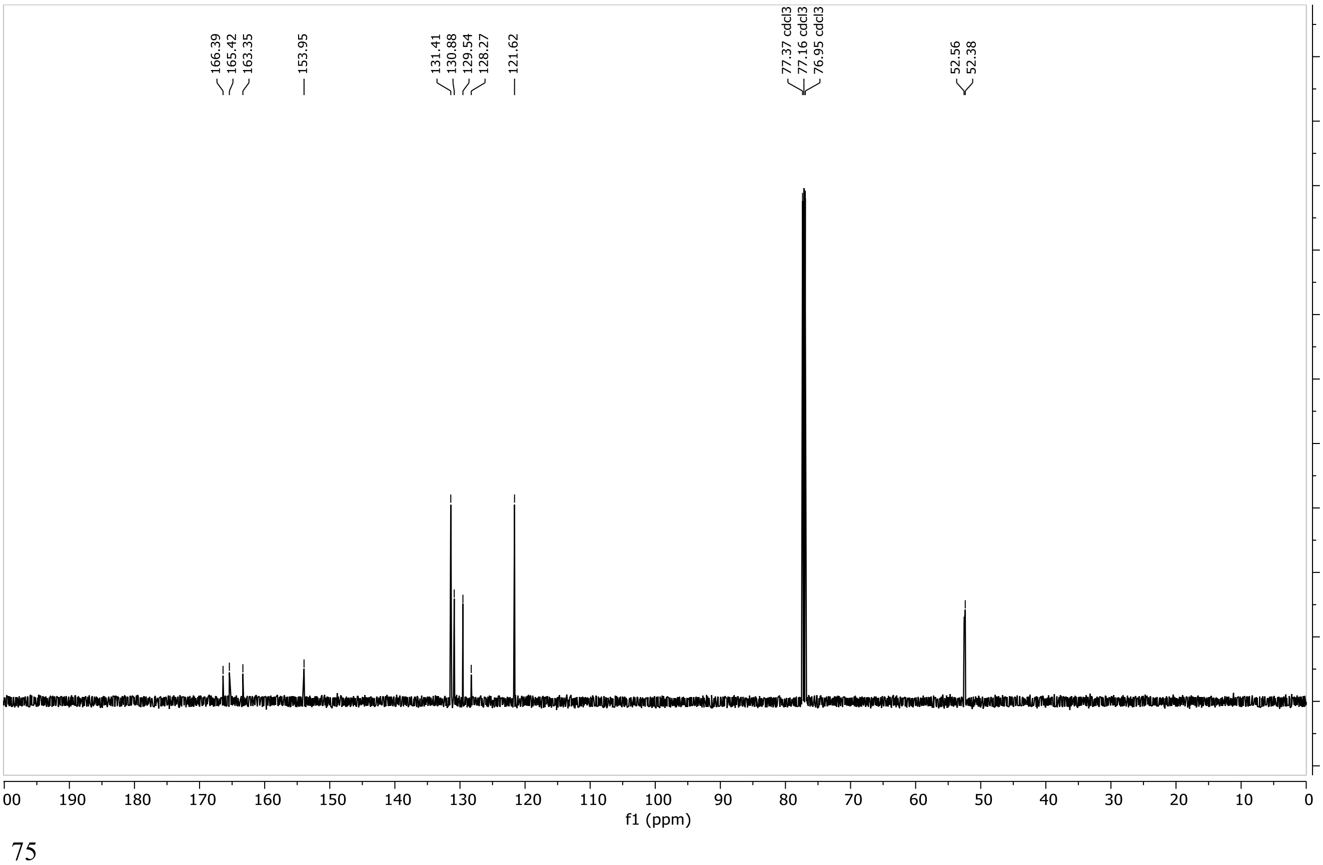

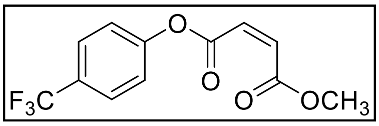

Z-16

^{1}H NMR (500 MHz, $CDCl_3$)

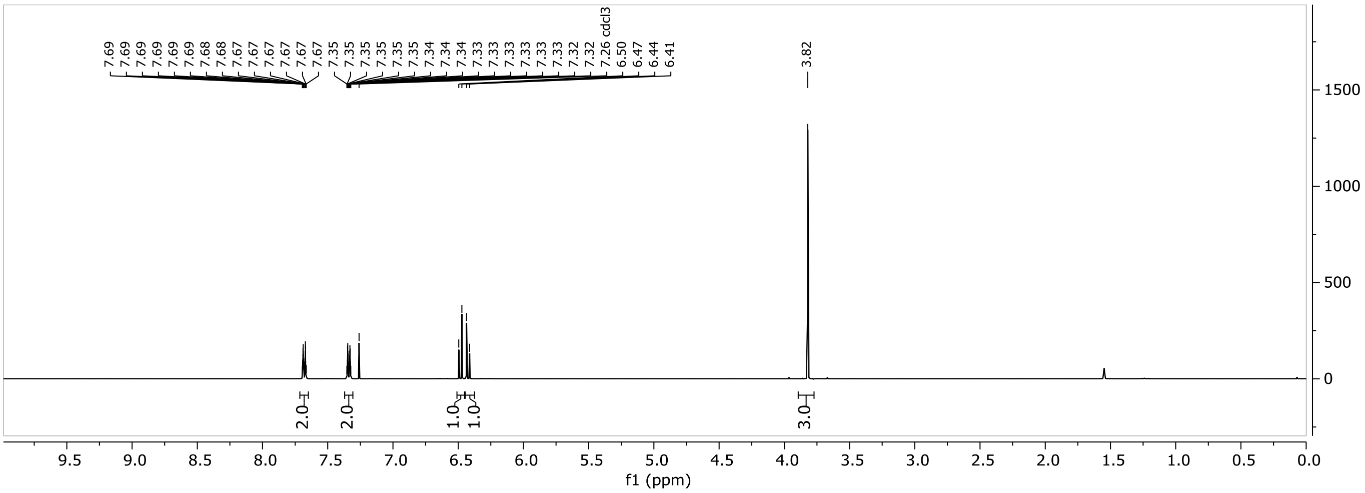

^{13}C-{^{19}F} NMR (126 MHz, $CDCl_3$)

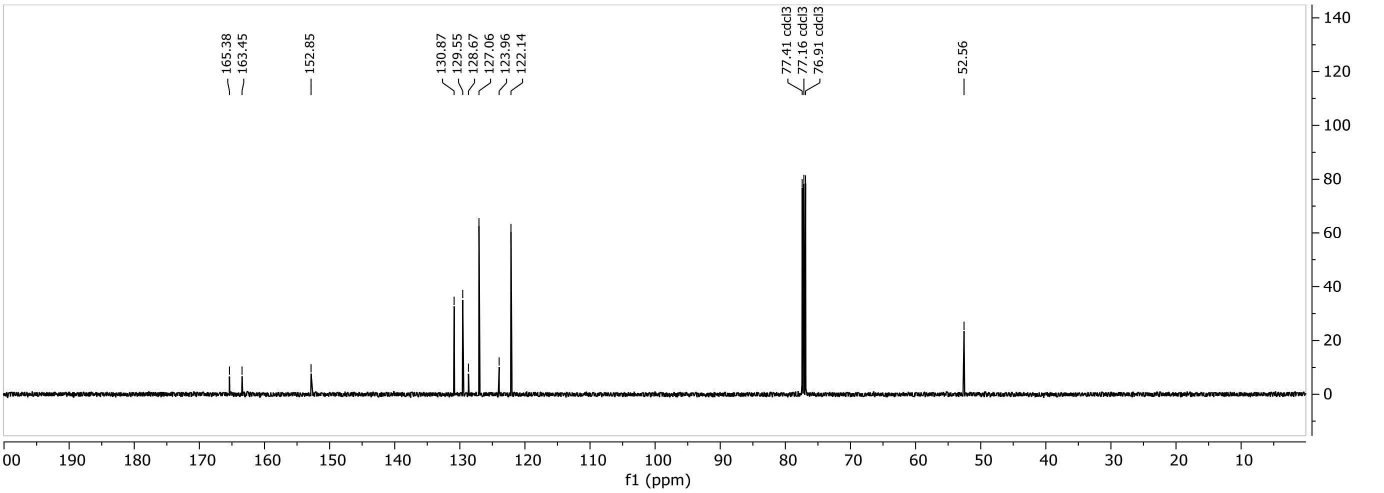

^{19}F NMR (376 MHz, $CDCl_3$)

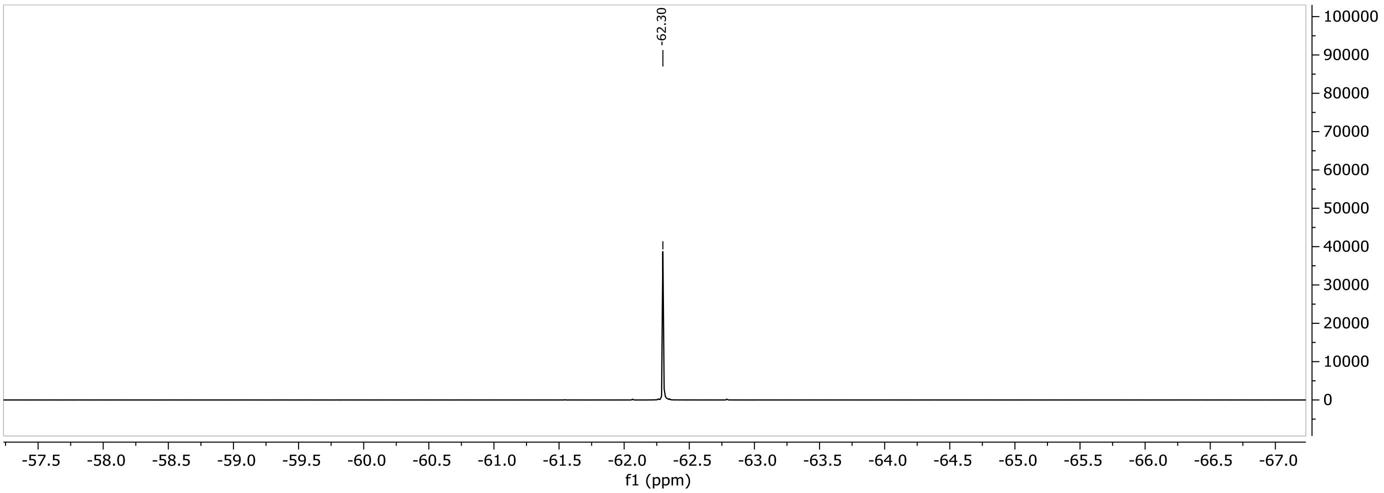

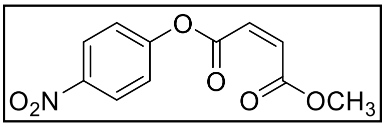

Z-17

^{1}H NMR (500 MHz, $CDCl_3$)

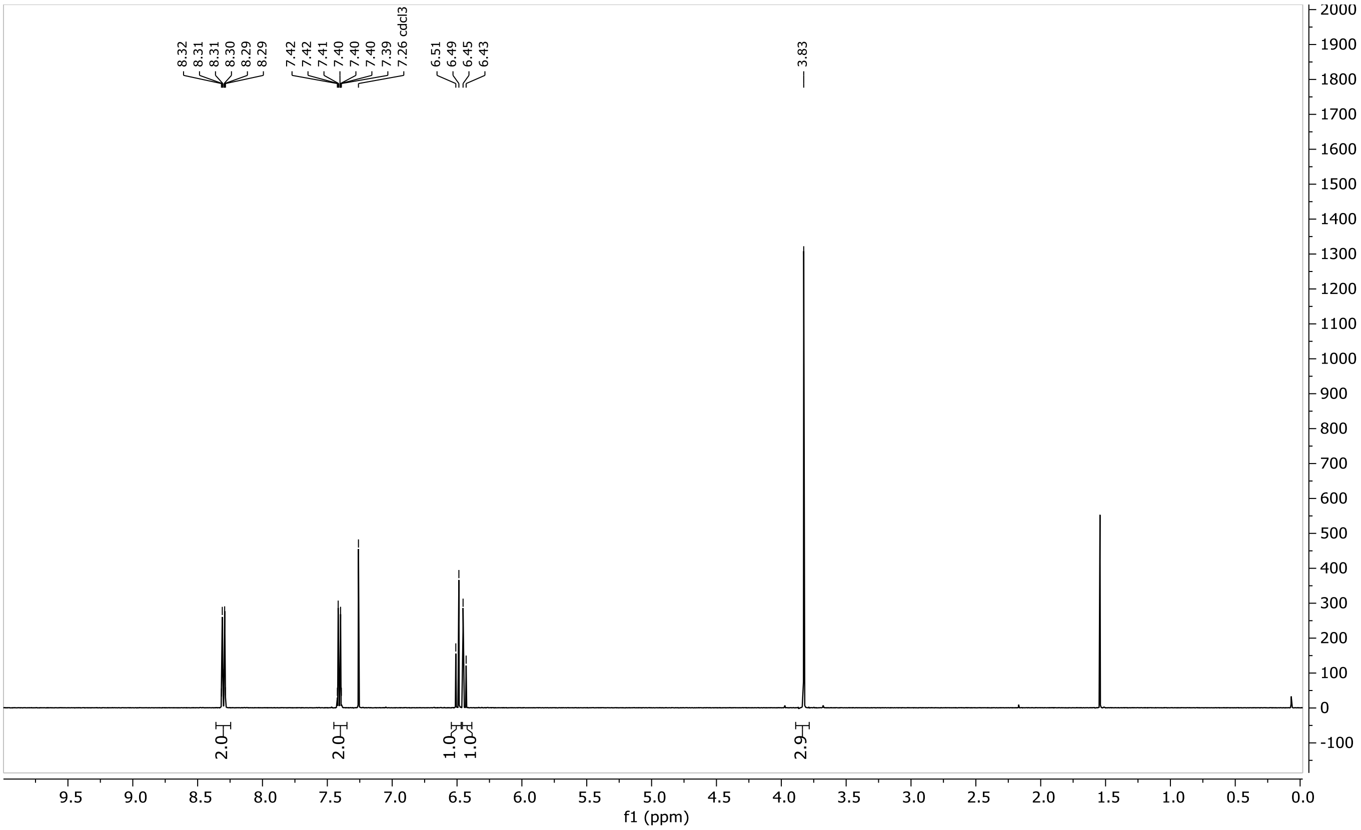

^{13}C NMR (126 MHz, $CDCl_3$)

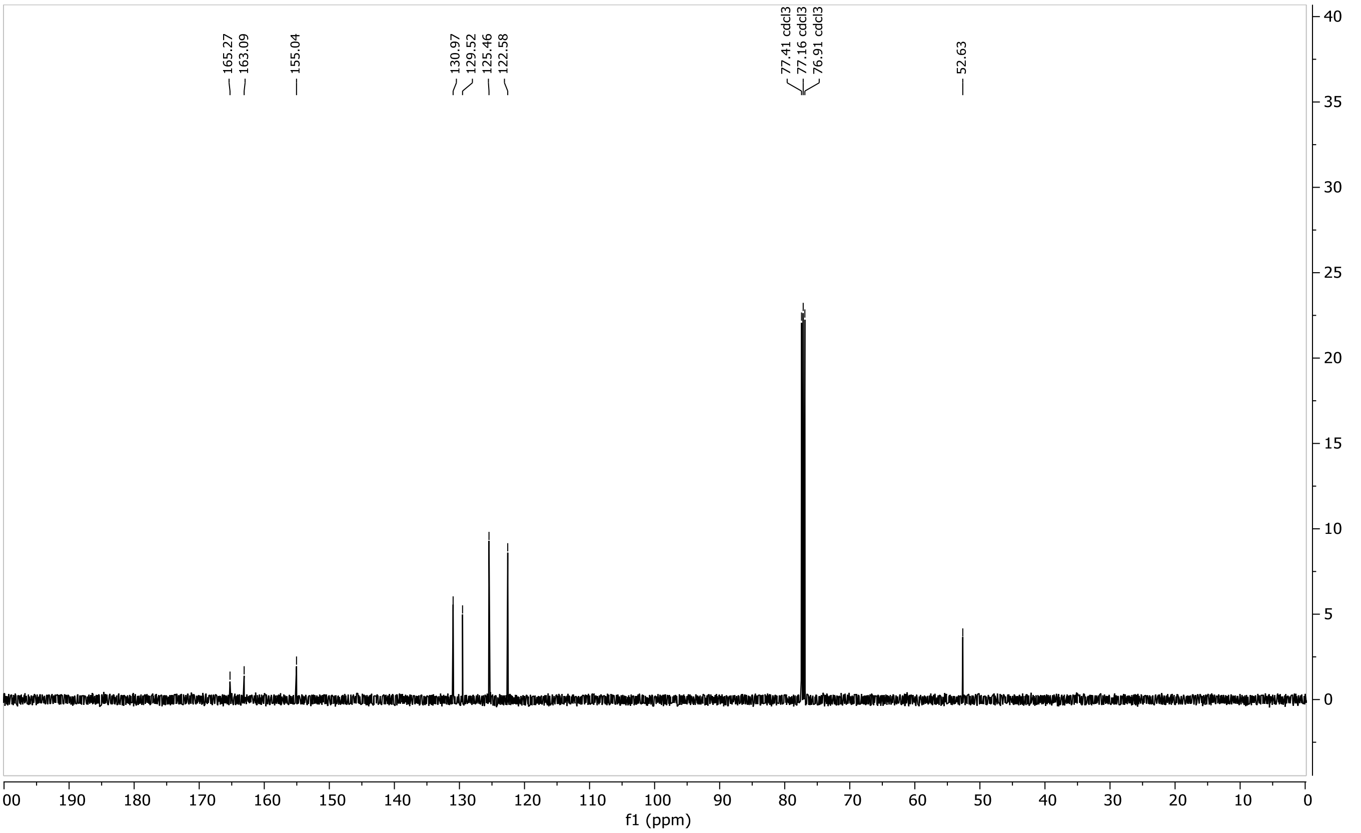

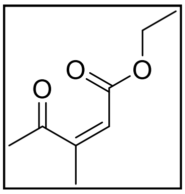

Z-18

^{1}H NMR (400 MHz, $CDCl_3$)

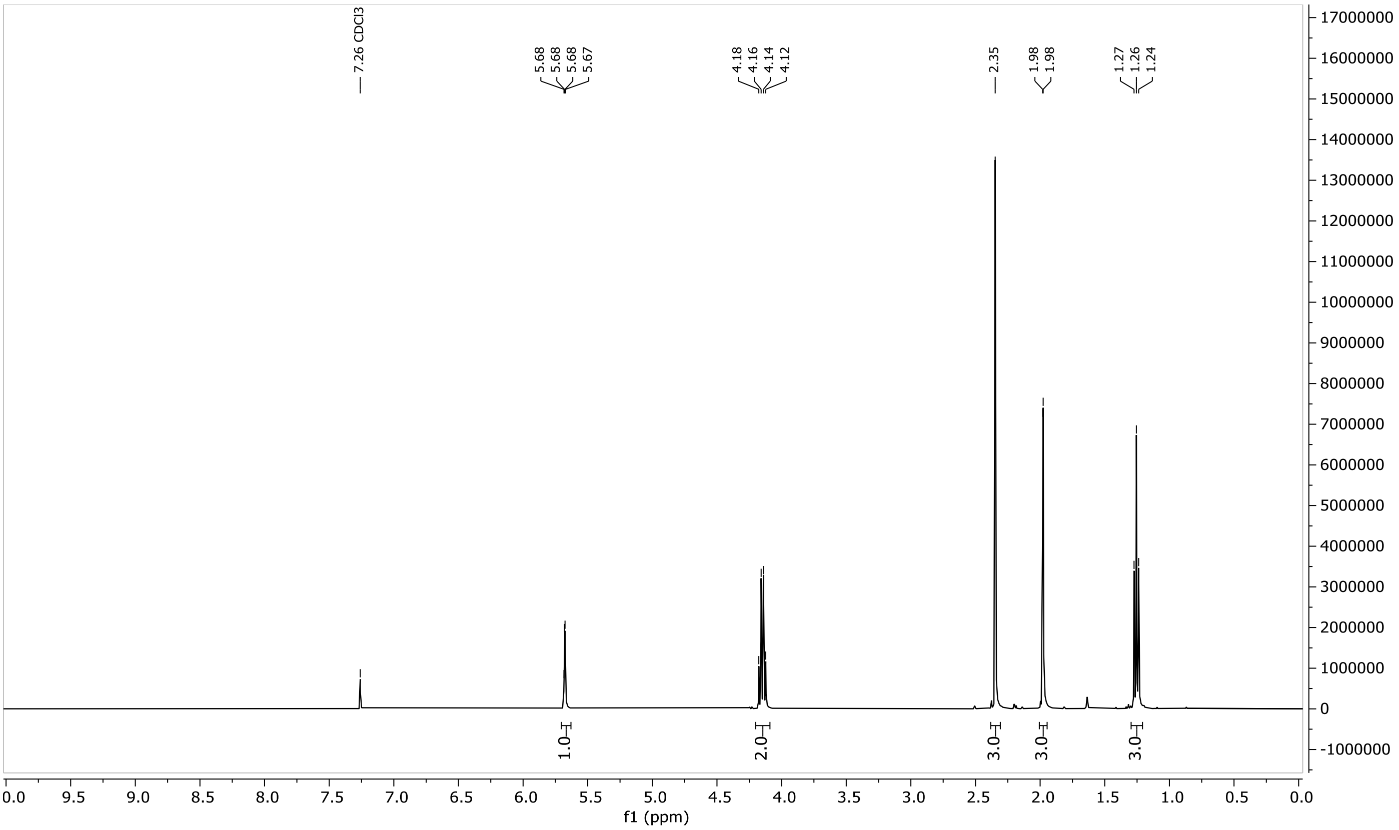

^{13}C NMR (100 MHz, $CDCl_3$)

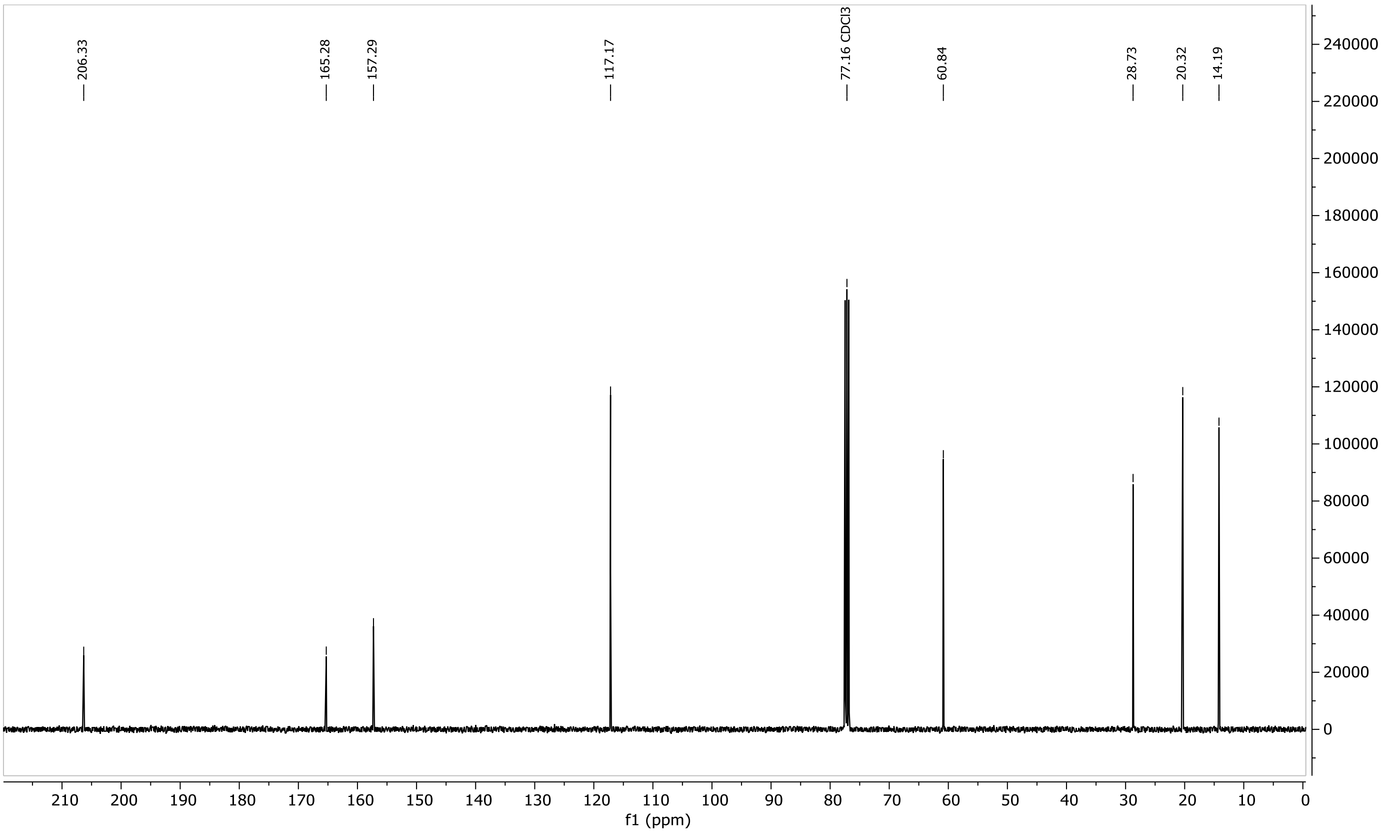

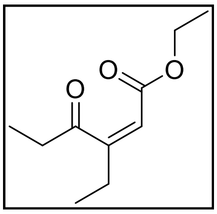

Z-19

^{1}H NMR (500 MHz, $CDCl_3$)

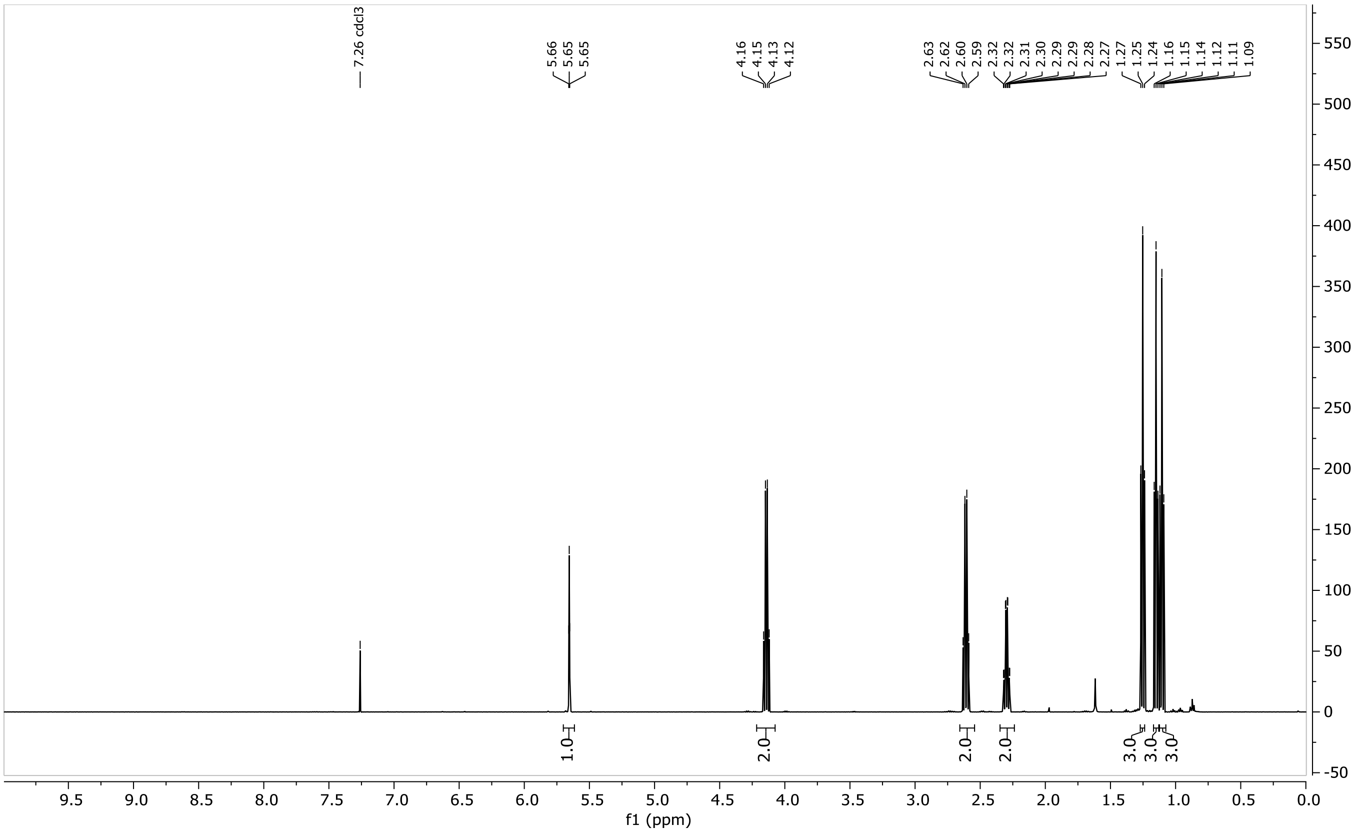

^{13}C NMR (126 MHz, $CDCl_3$)

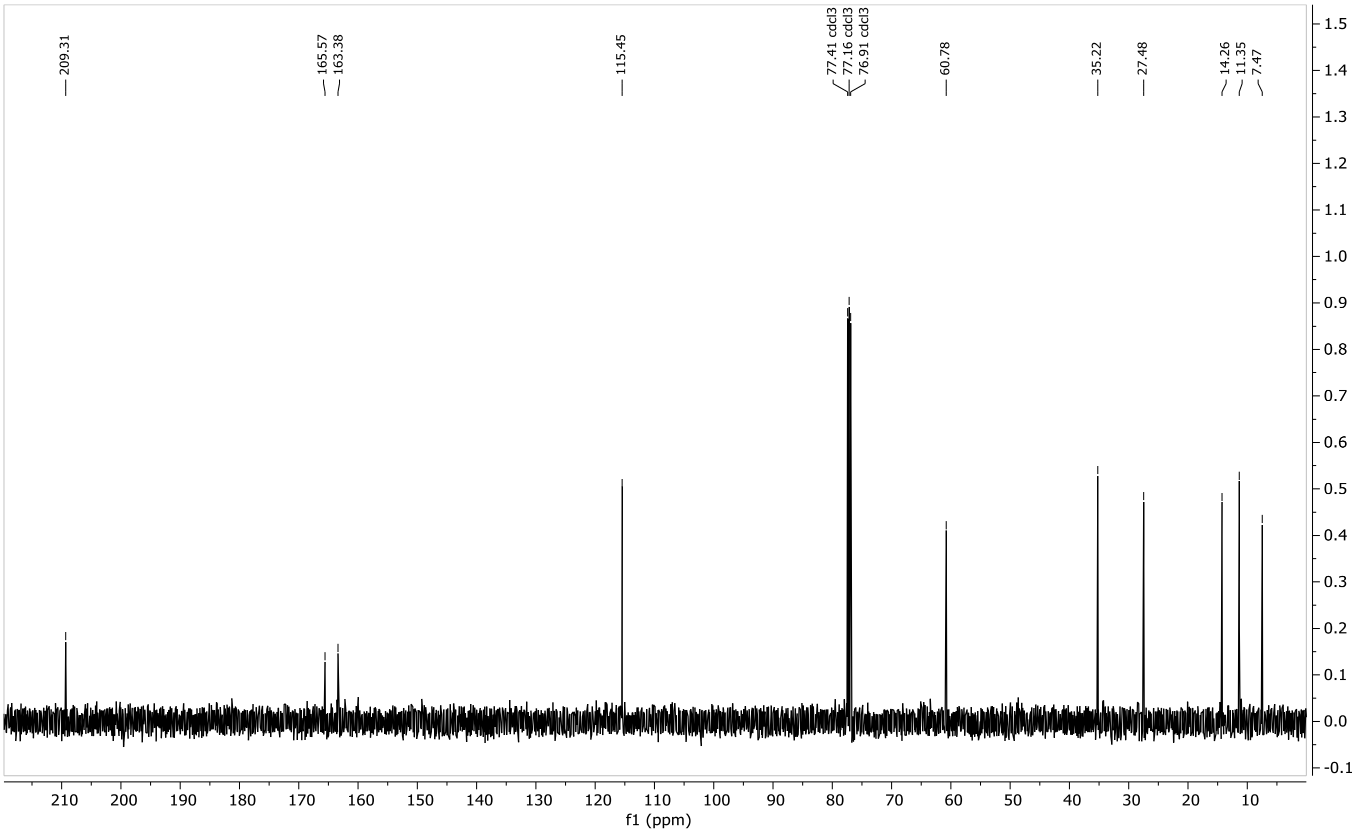

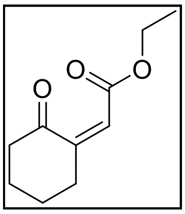

Z-20

^{1}H NMR (500 MHz, CD_3CN)

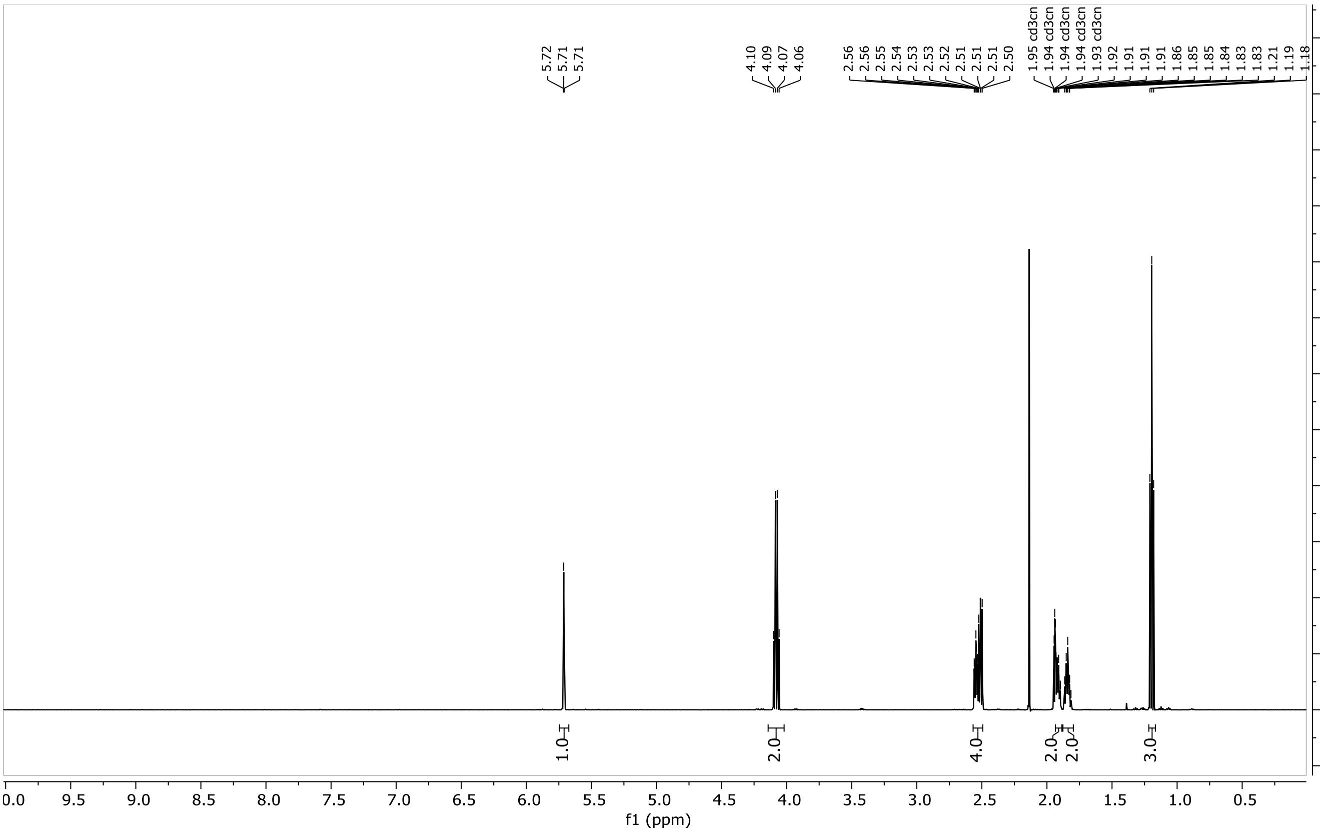

^{13}C NMR (126 MHz, CD_3CN)

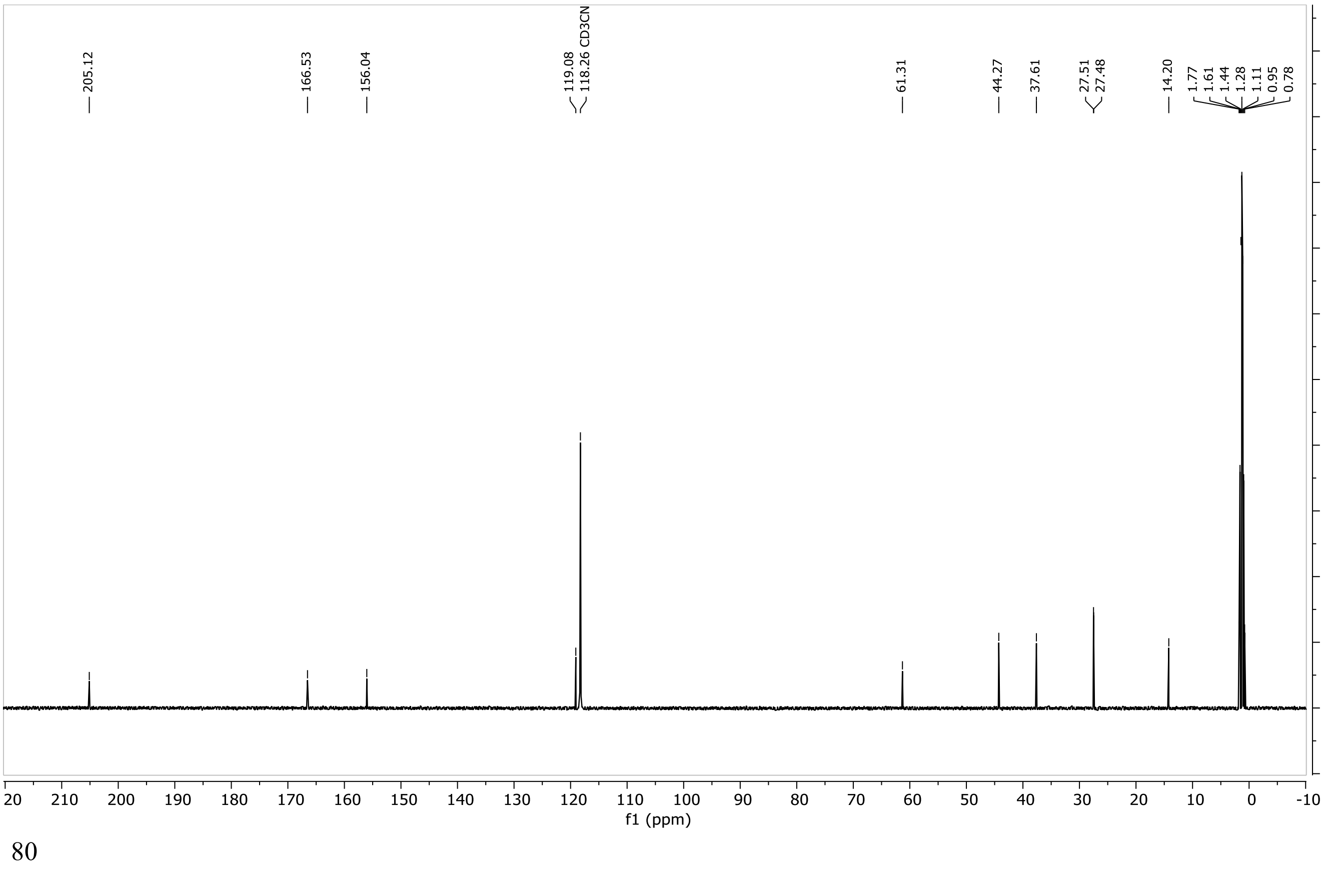

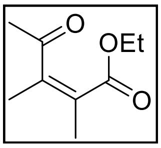

Z-21

^{1}H NMR (400 MHz, $CDCl_3$)

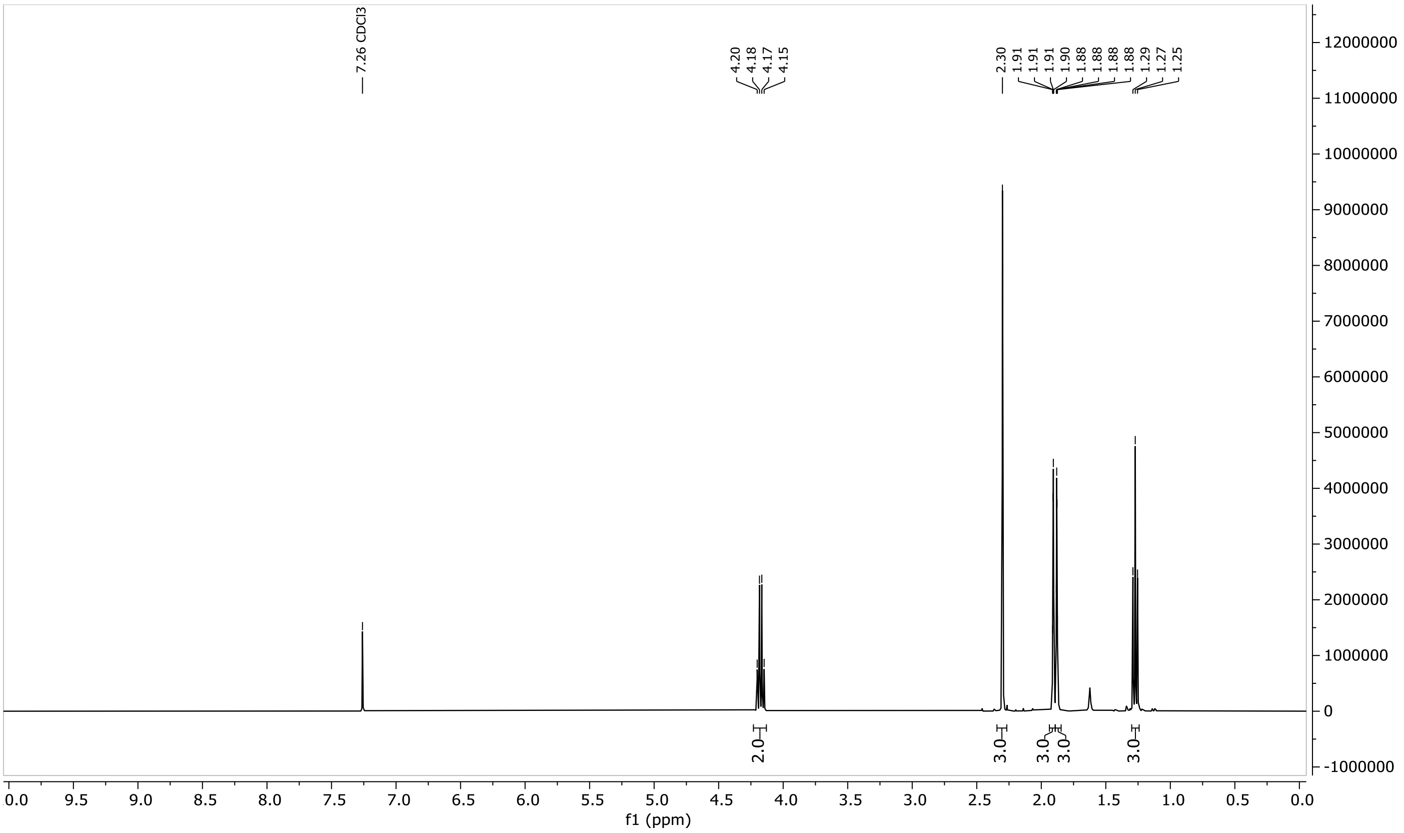

^{13}C NMR (100 MHz, $CDCl_3$)

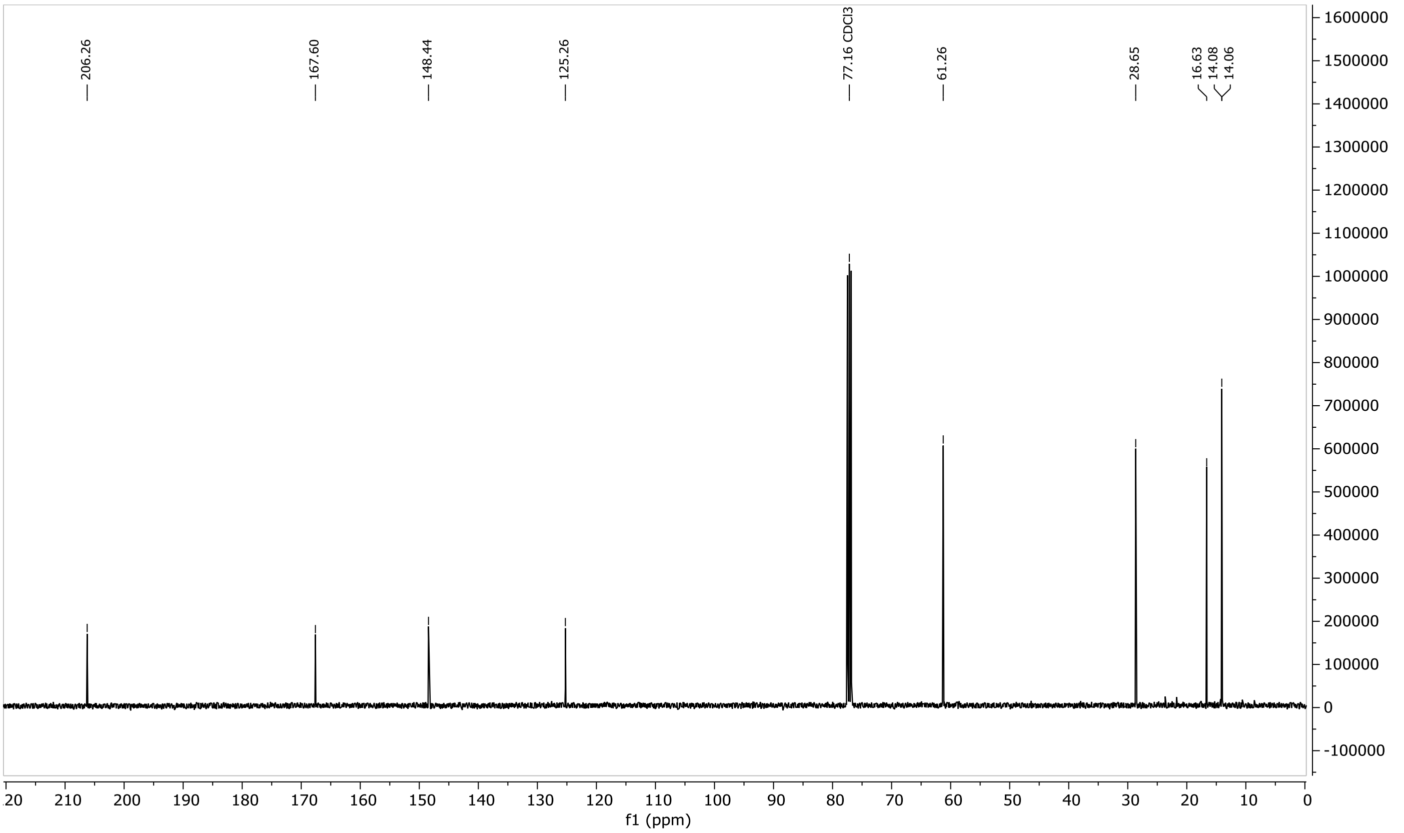

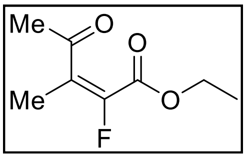

E-22

^{1}H NMR (600 MHz, CDCl$_3$)

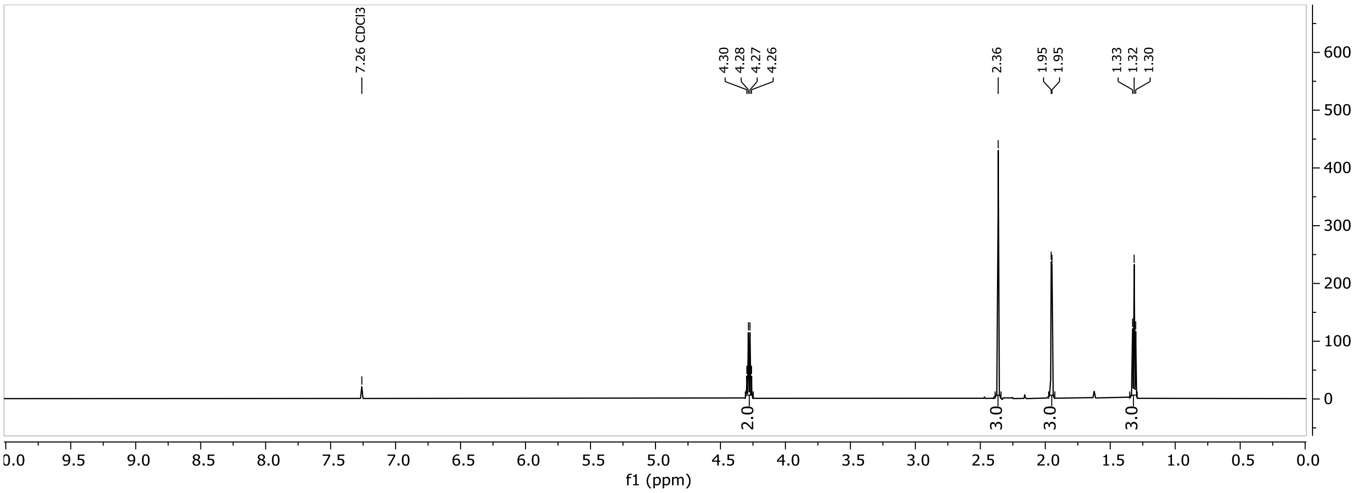

^{13}C-{^{19}F} NMR (151 MHz, CDCl$_3$)

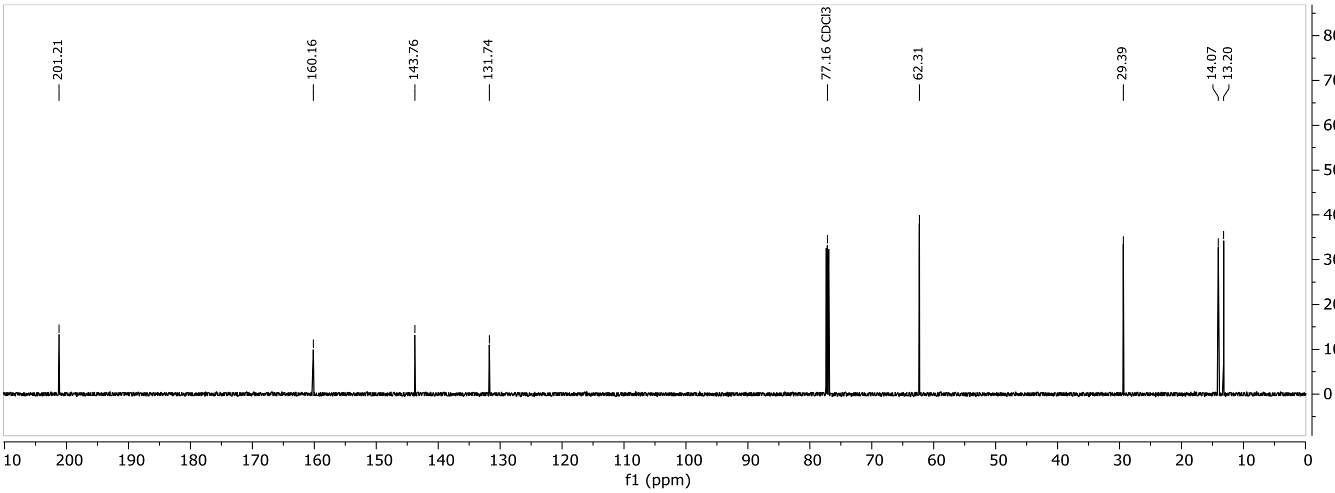

^{19}F NMR (564 MHz, CDCl$_3$)

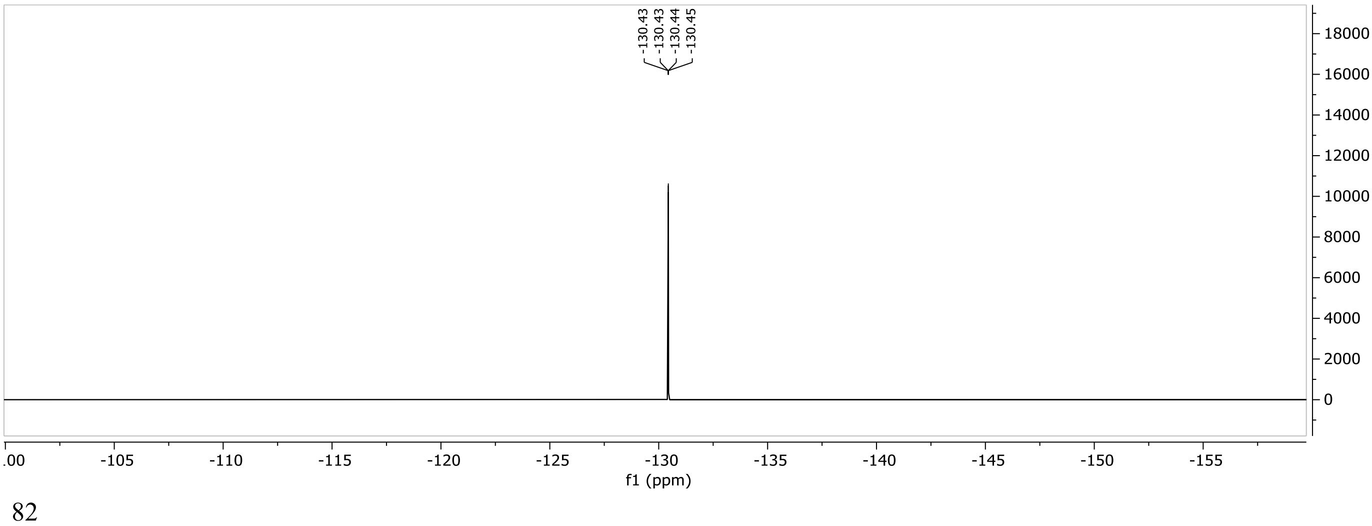

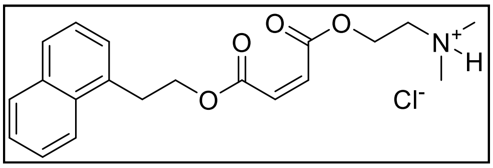

Z-23

^{1}H NMR (600 MHz, $CDCl_3$)

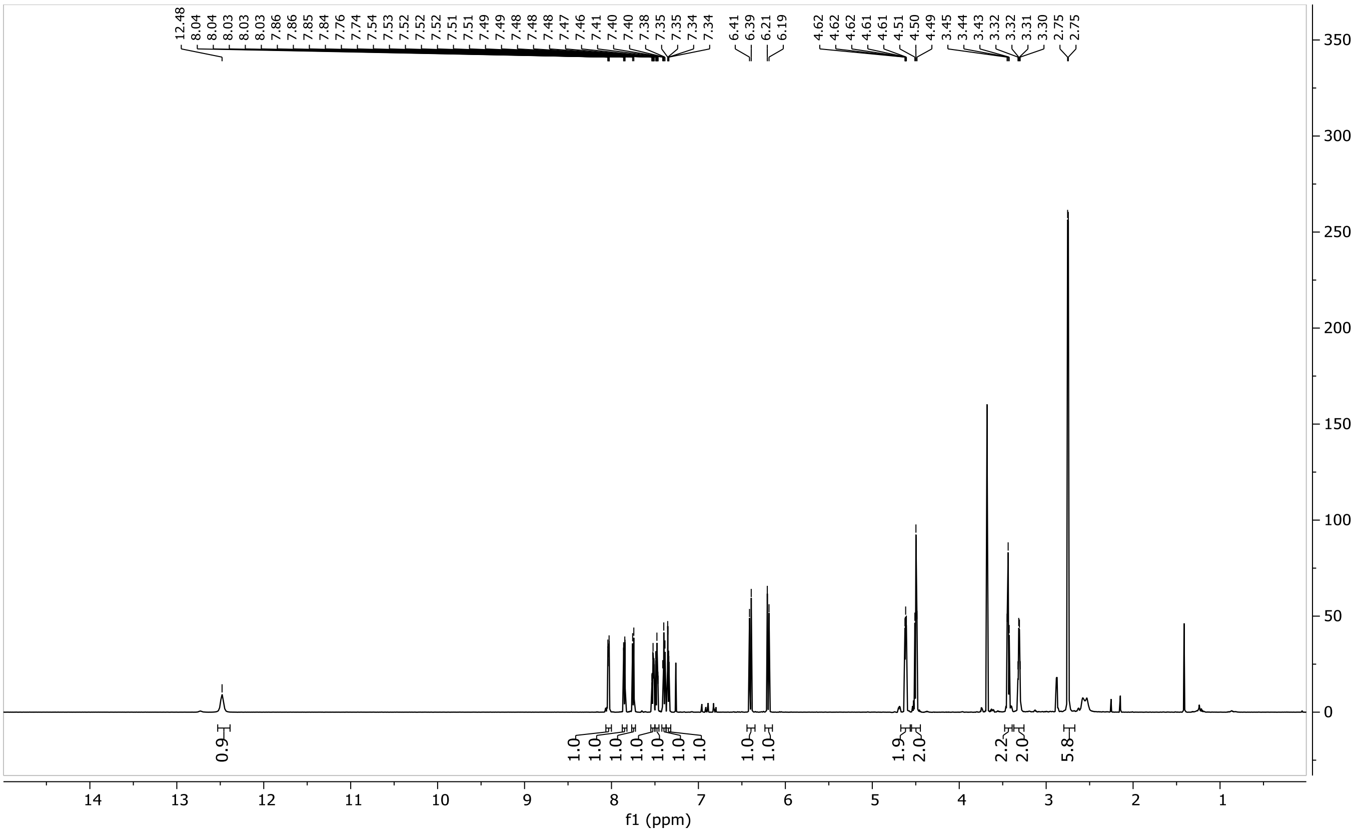

^{13}C NMR (151 MHz, $CDCl_3$)

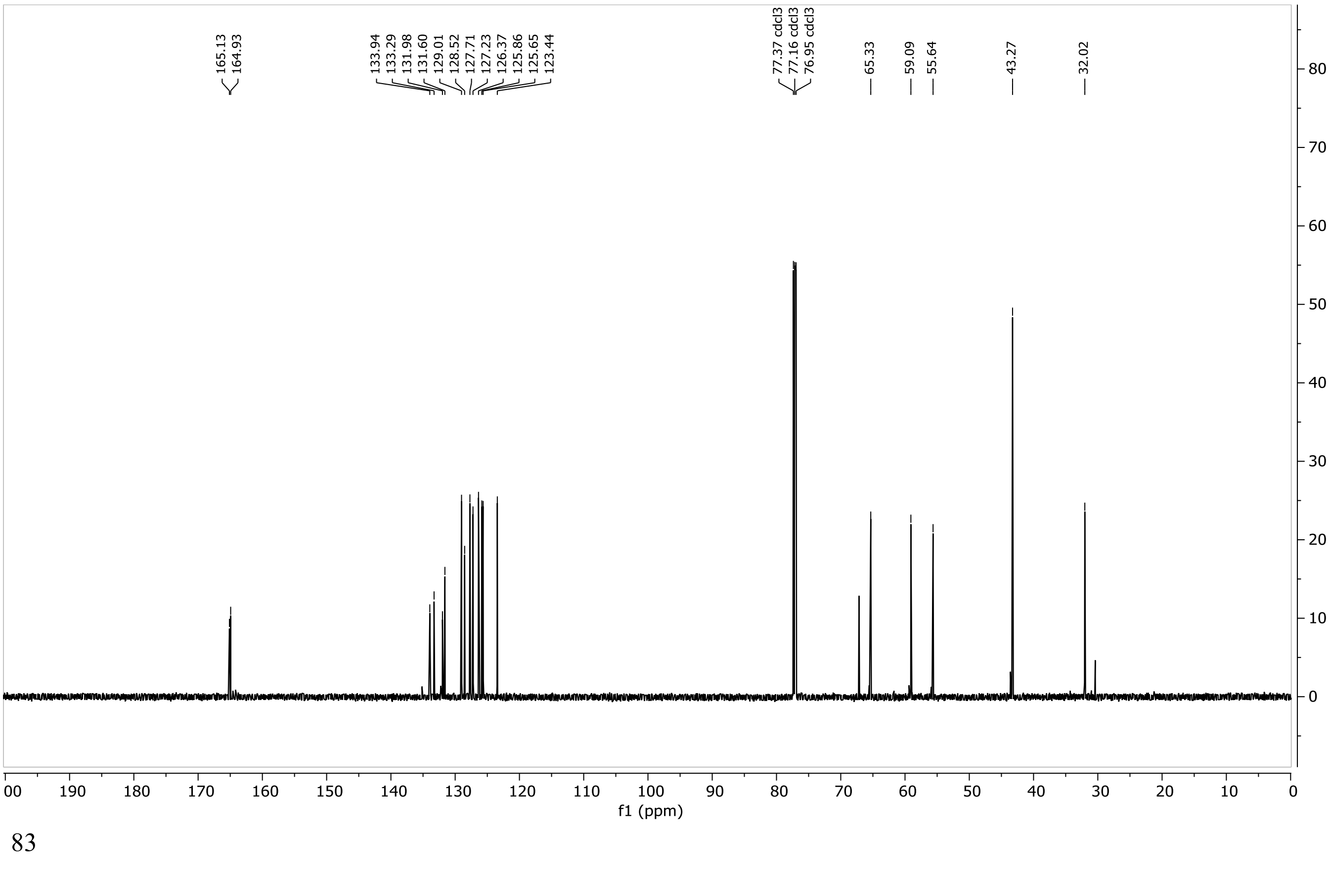

Z-24

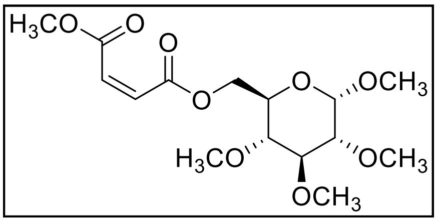

^{1}H NMR (600 MHz, $CDCl_3$)

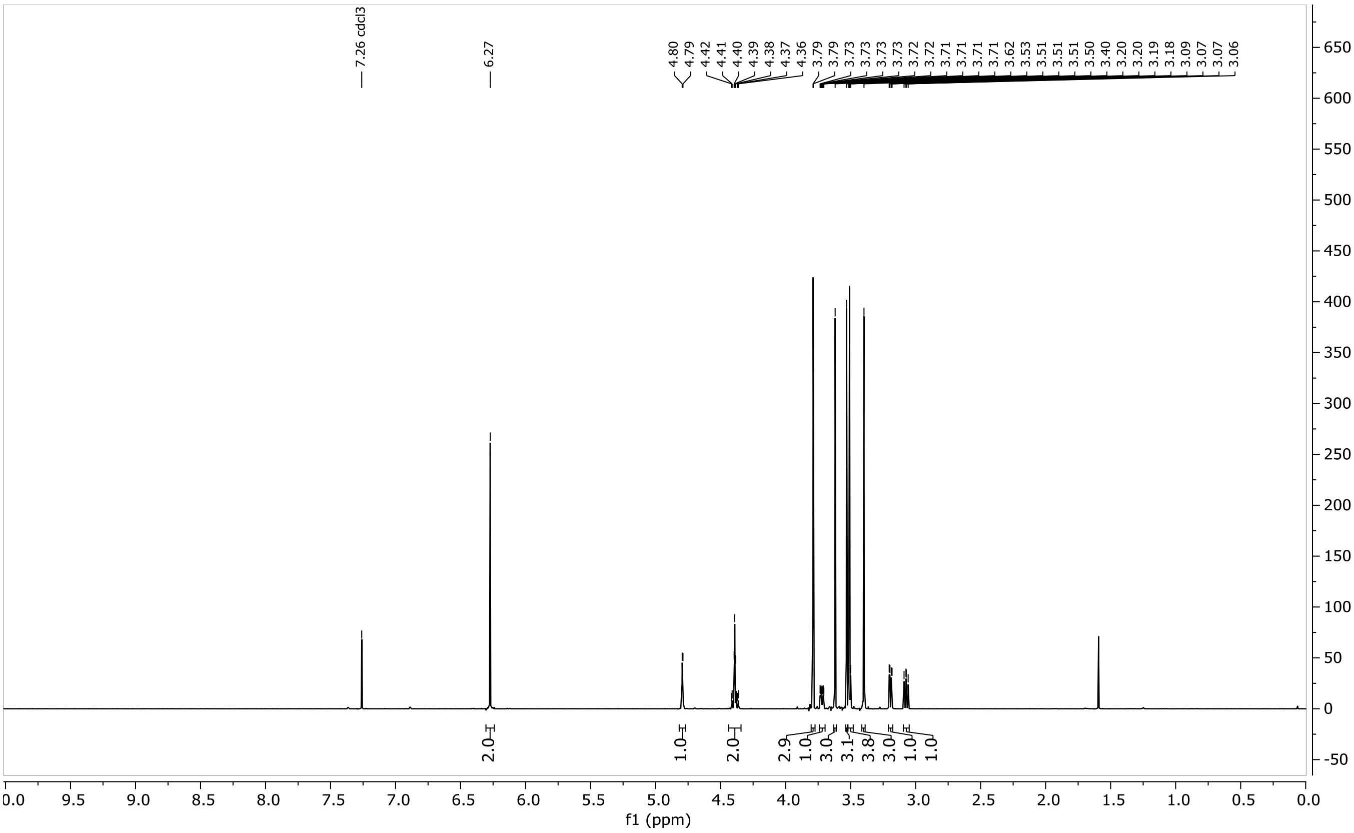

^{13}C NMR (151 MHz, $CDCl_3$)

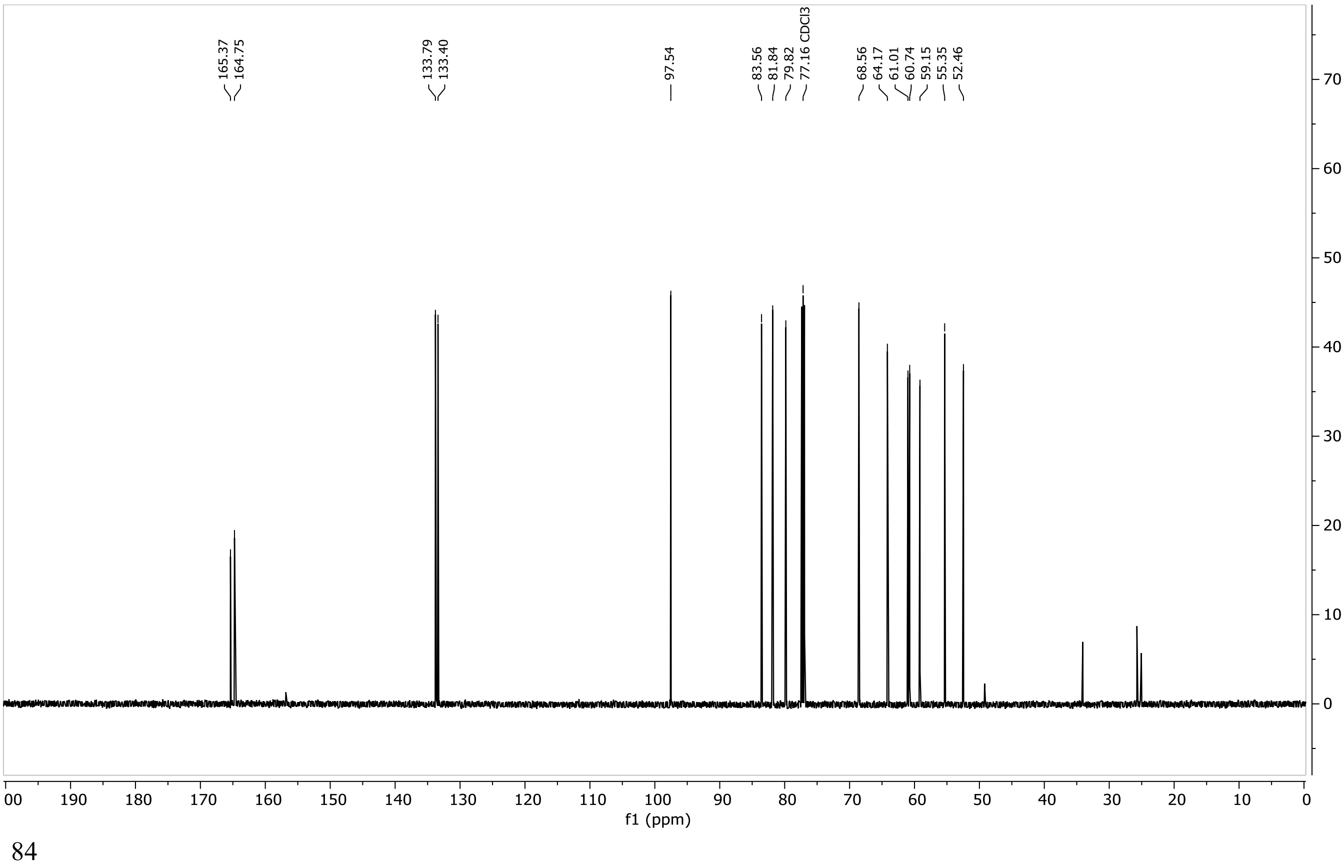

Z-25

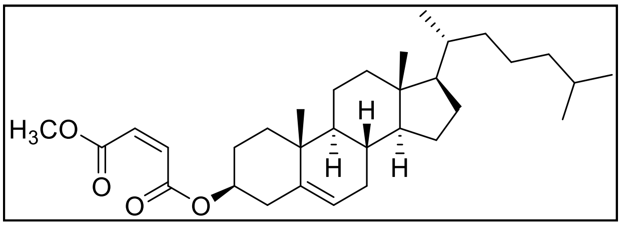

^{1}H NMR (500 MHz, $CDCl_3$)

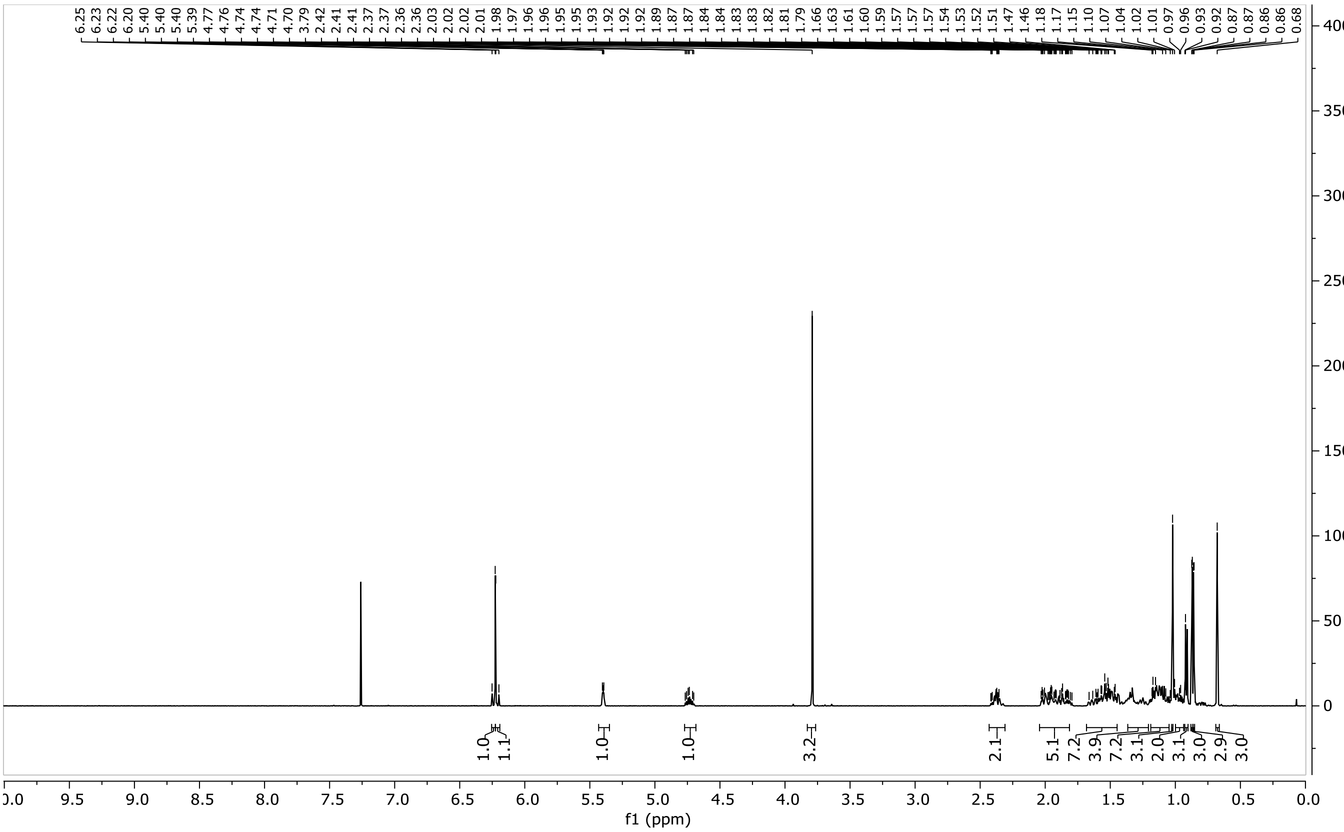

^{13}C NMR (126 MHz, $CDCl_3$)

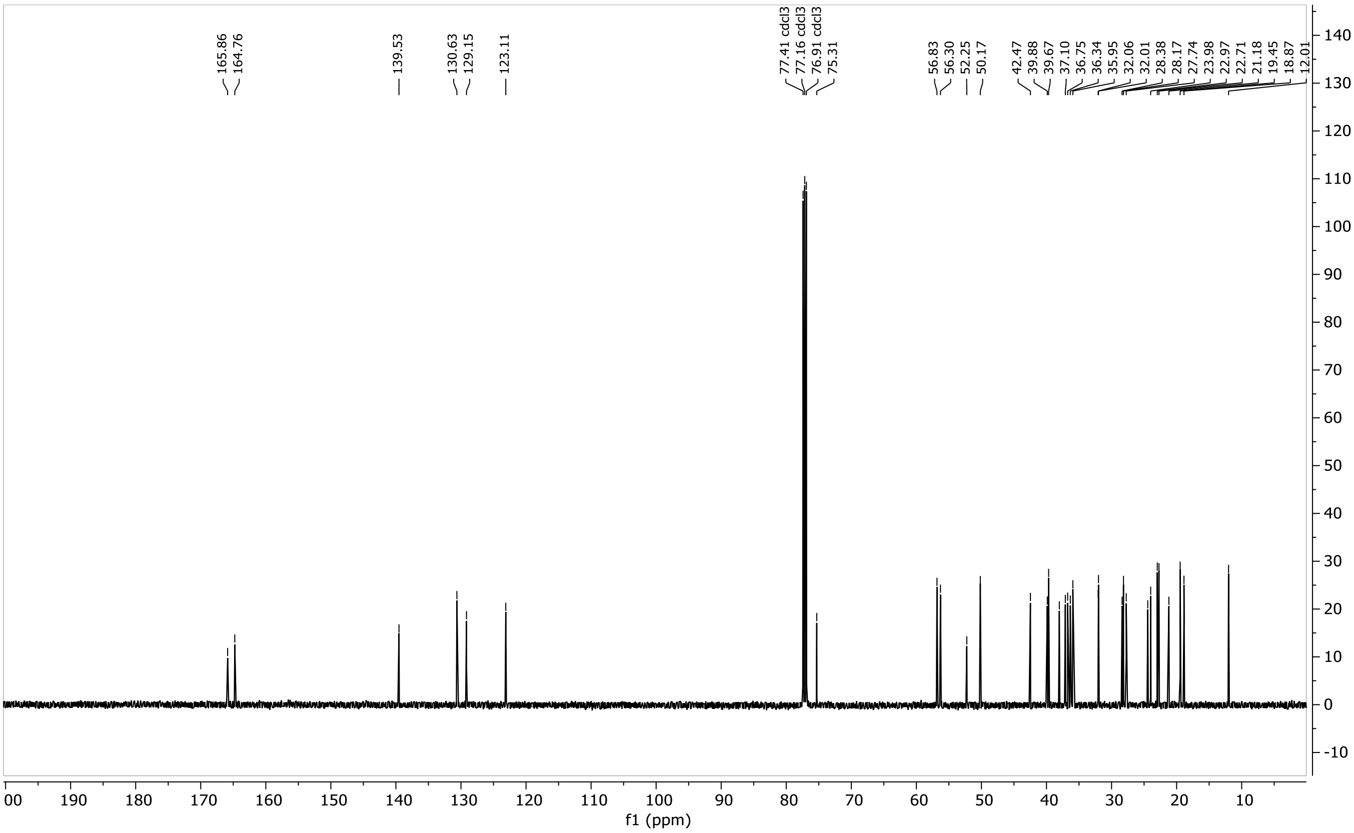

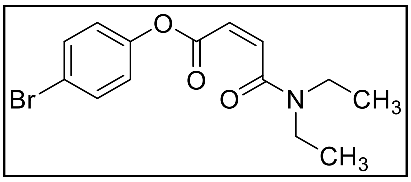

Z-26

^{1}H NMR (600 MHz, $CDCl_3$)

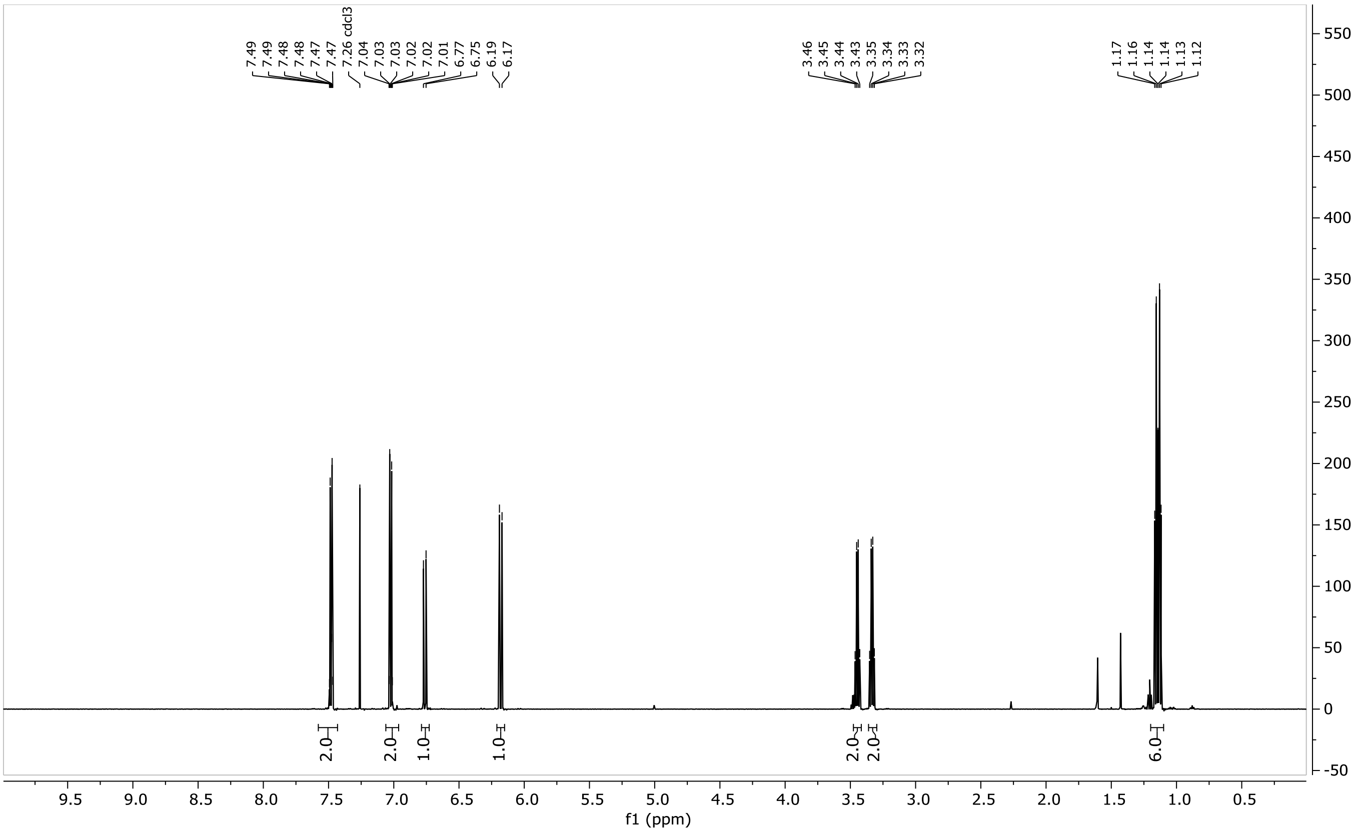

^{13}C NMR (151 MHz, $CDCl_3$)

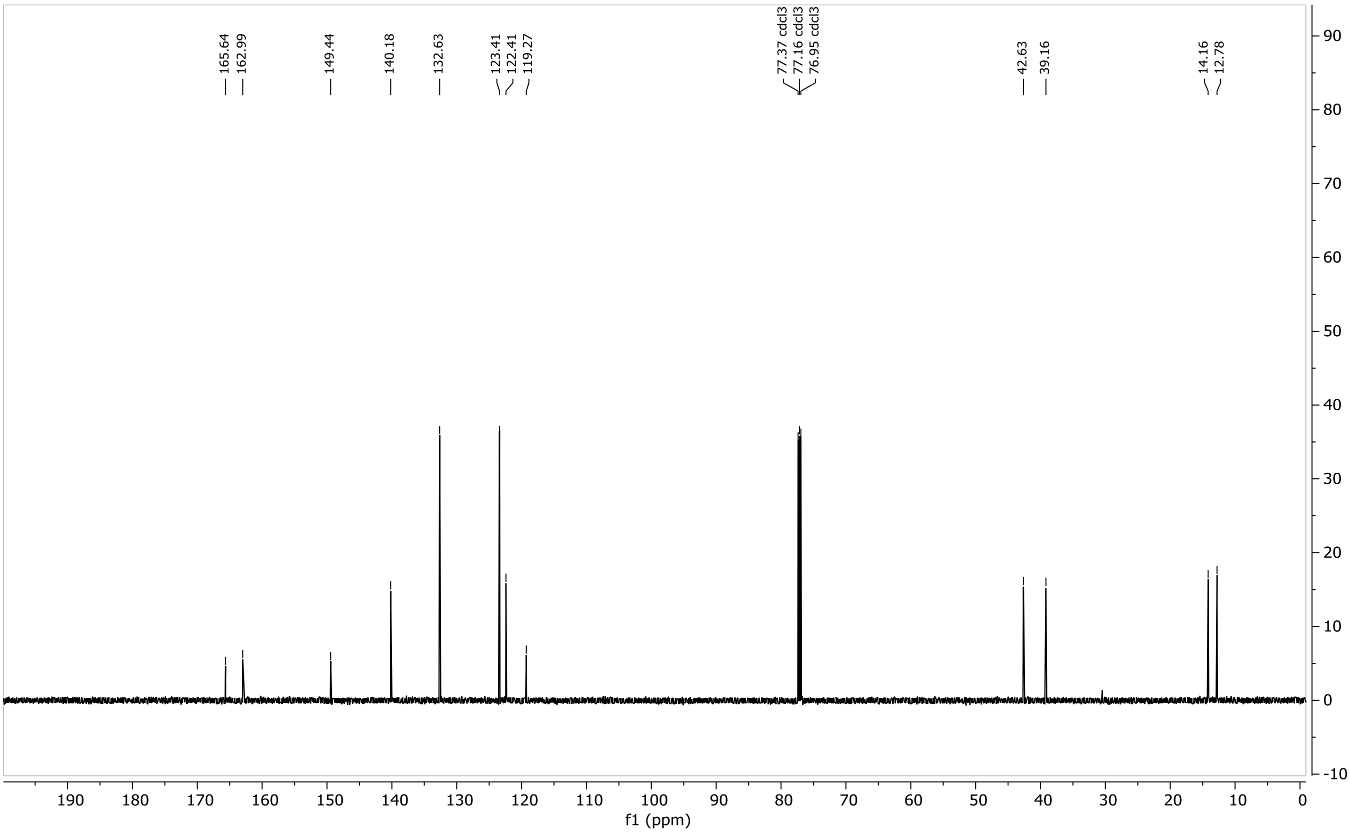

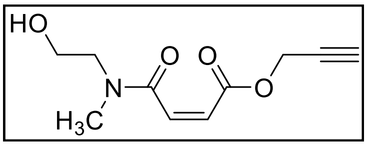

Z-27

^{1}H NMR (500 MHz, $CDCl_3$)

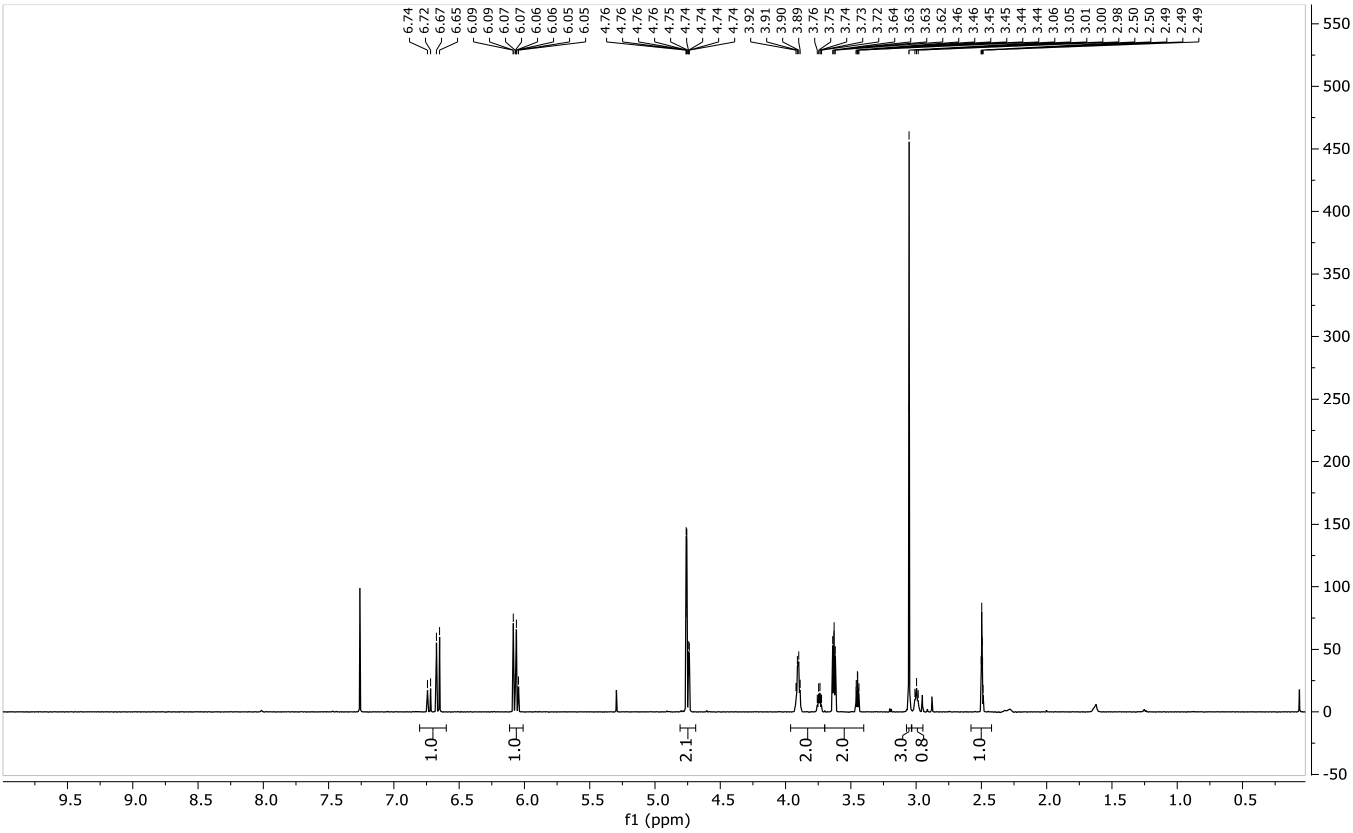

^{13}C NMR (126 MHz, $CDCl_3$)

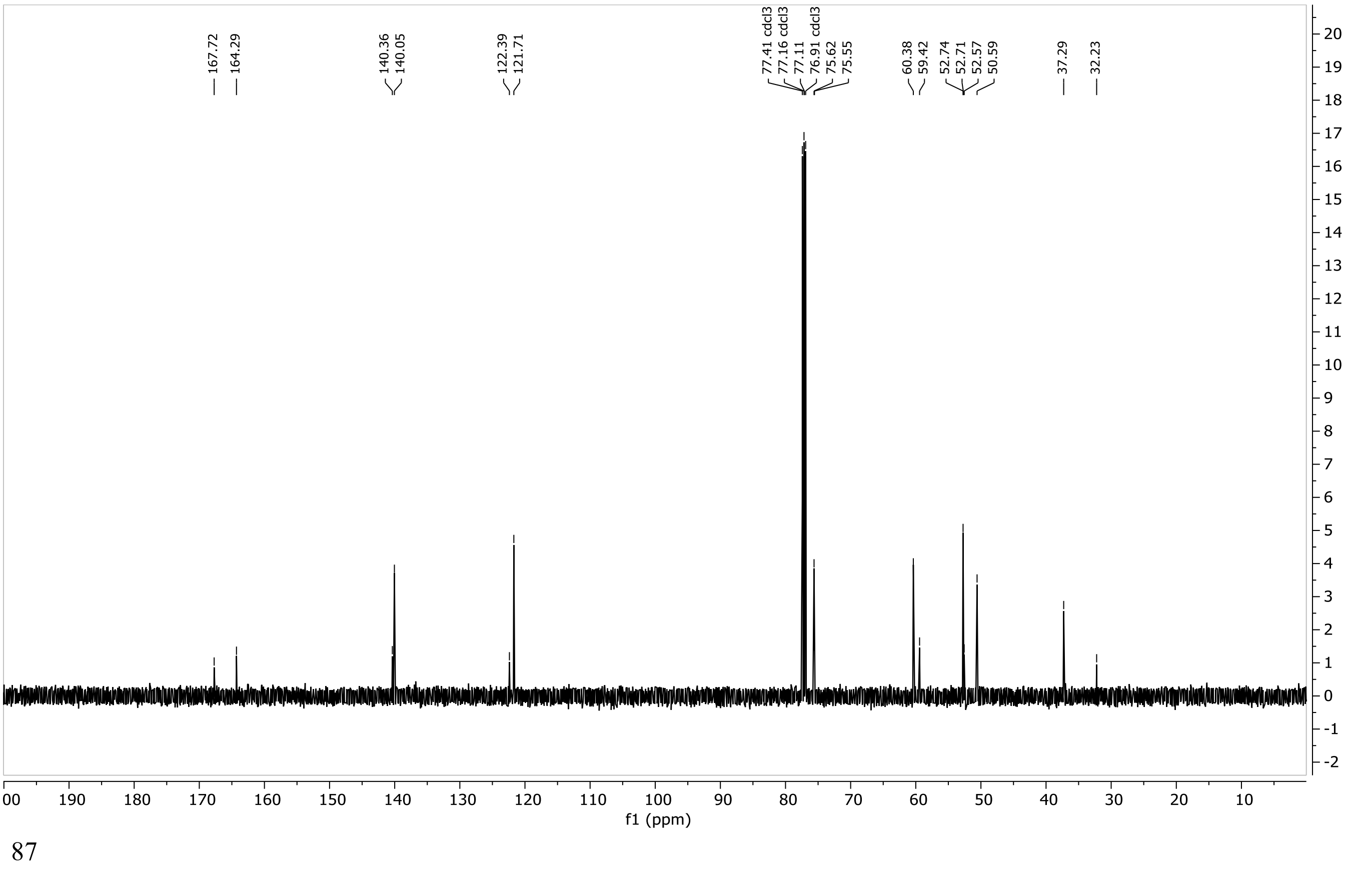

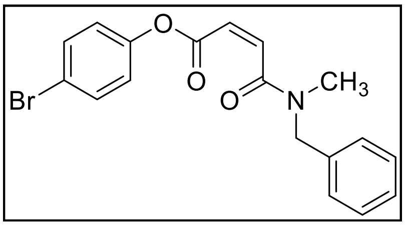

Z-28

^{1}H NMR (500 MHz, $CDCl_3$)

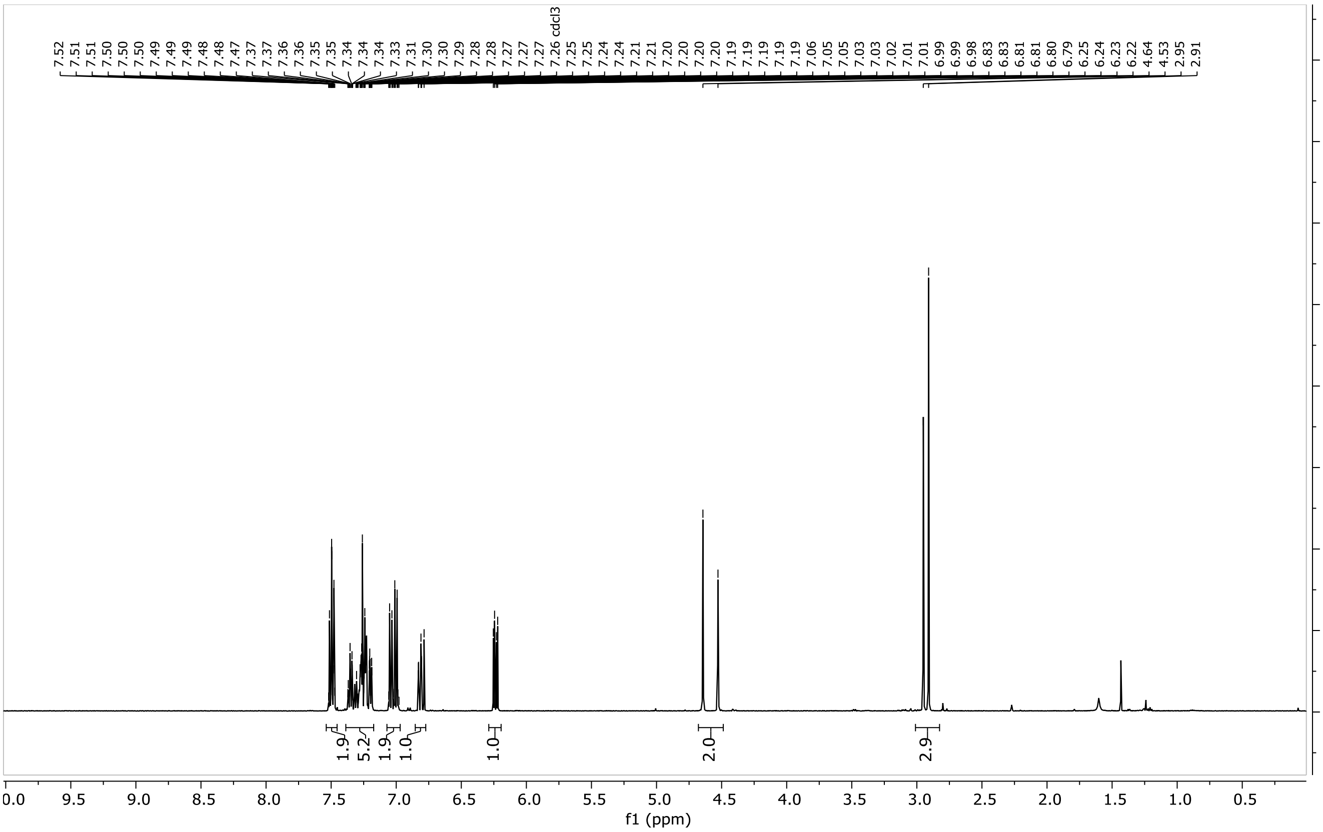

^{13}C NMR (126 MHz, $CDCl_3$)

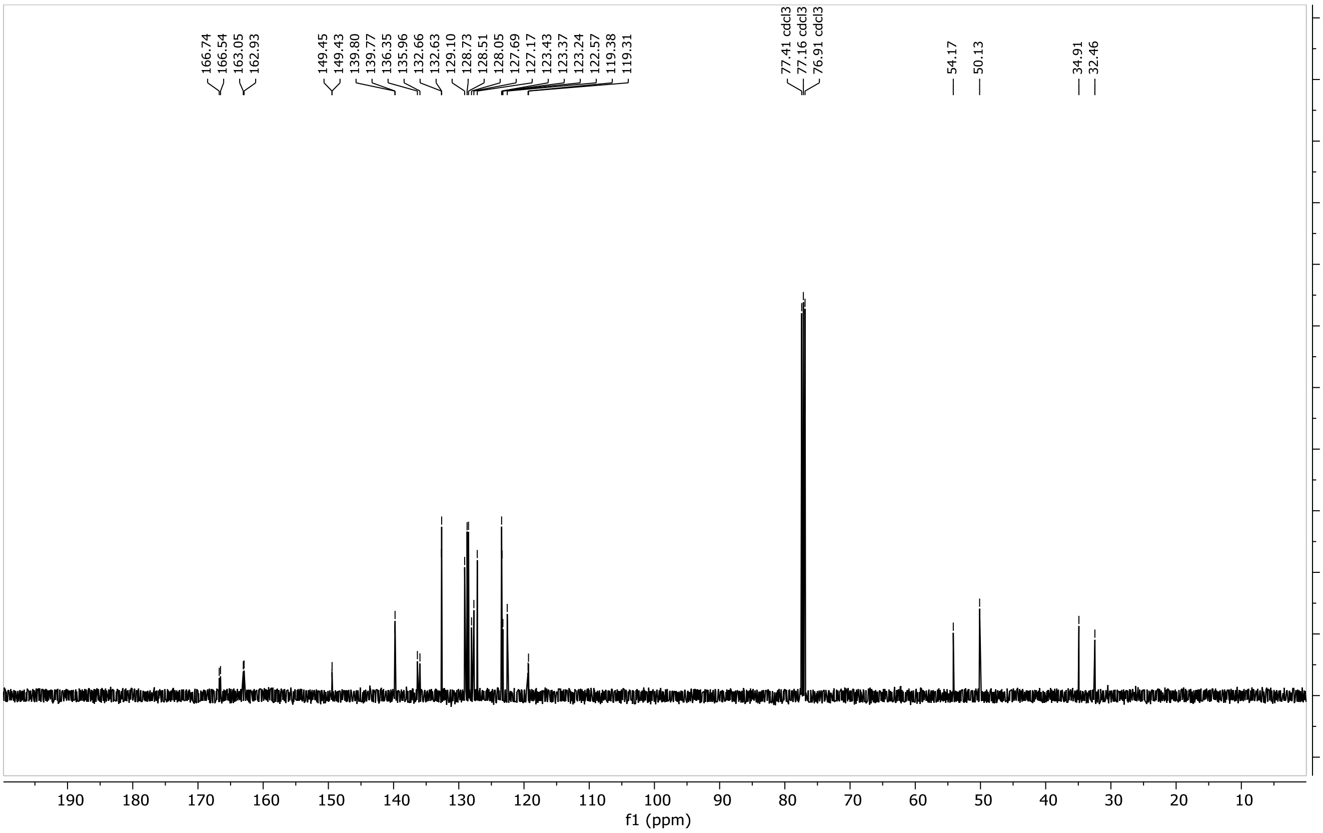

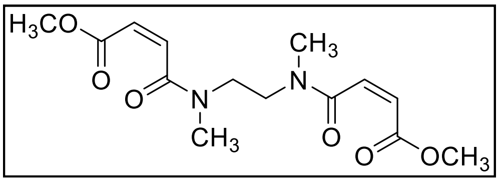

Z-29

^{1}H NMR (500 MHz, $CDCl_3$)

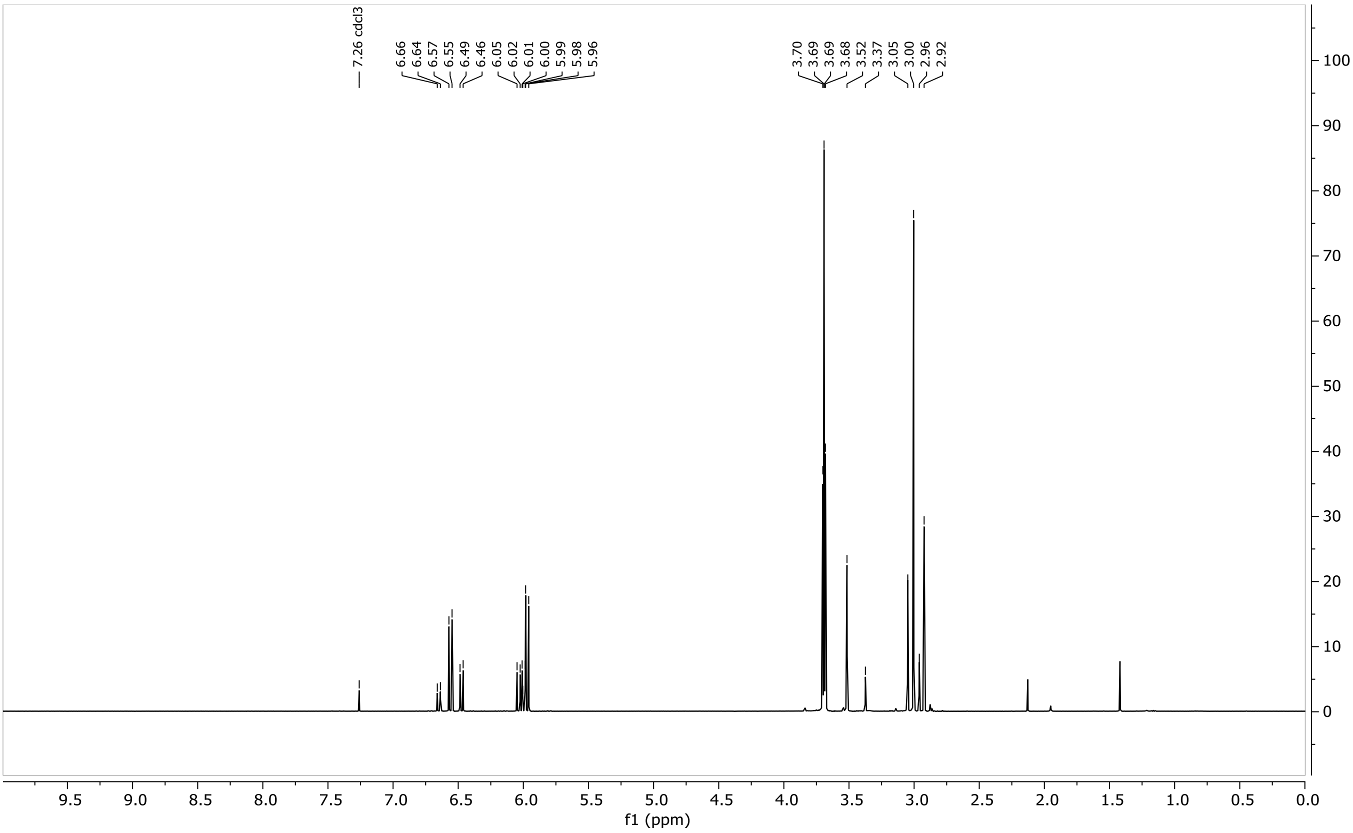

^{13}C NMR (126 MHz, $CDCl_3$)

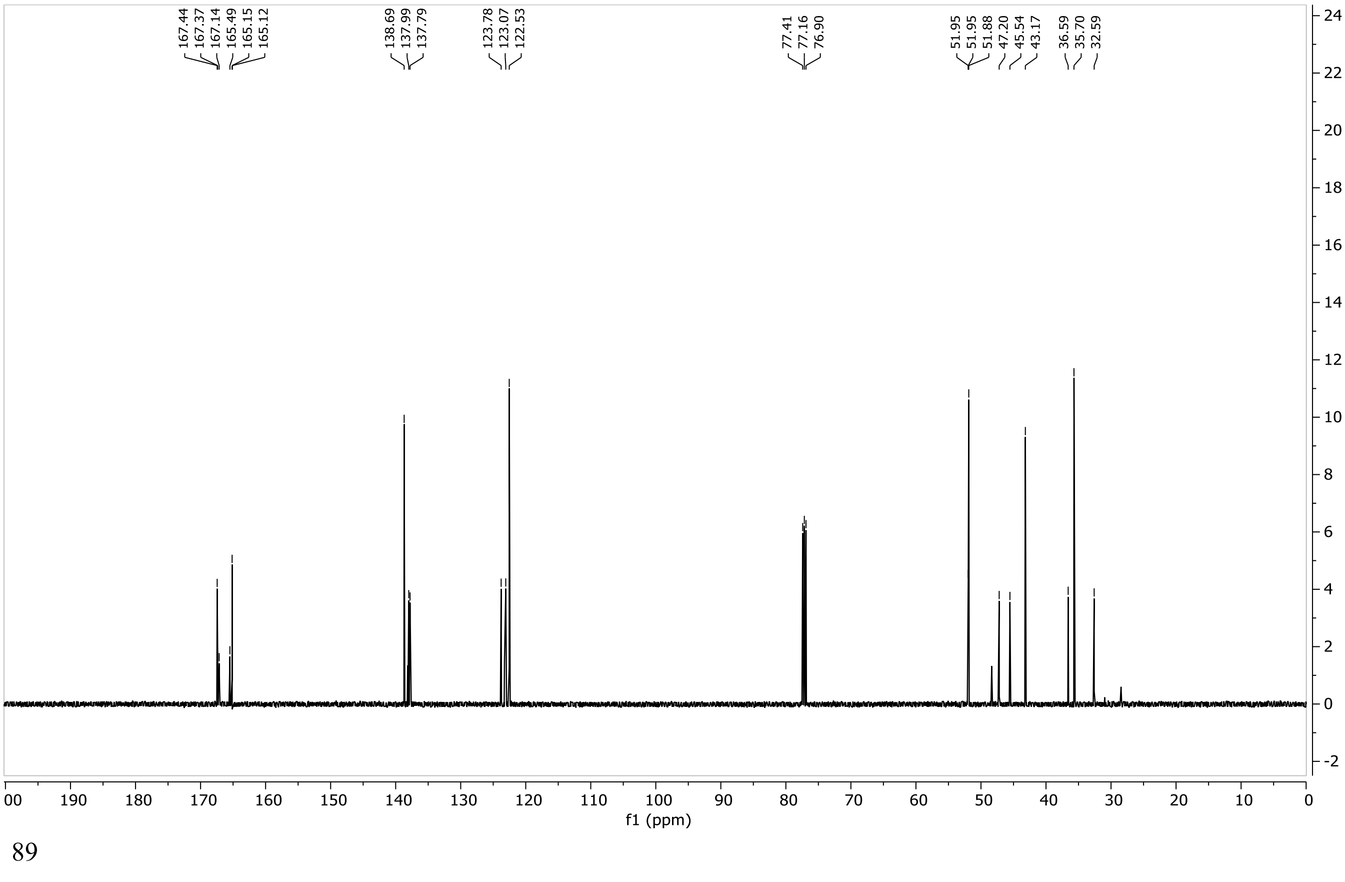

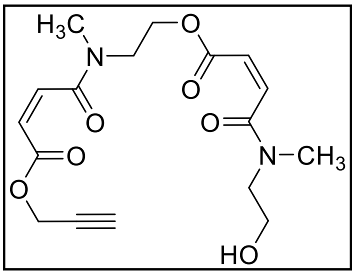

Z-30

^{1}H NMR (500 MHz, $CDCl_3$)

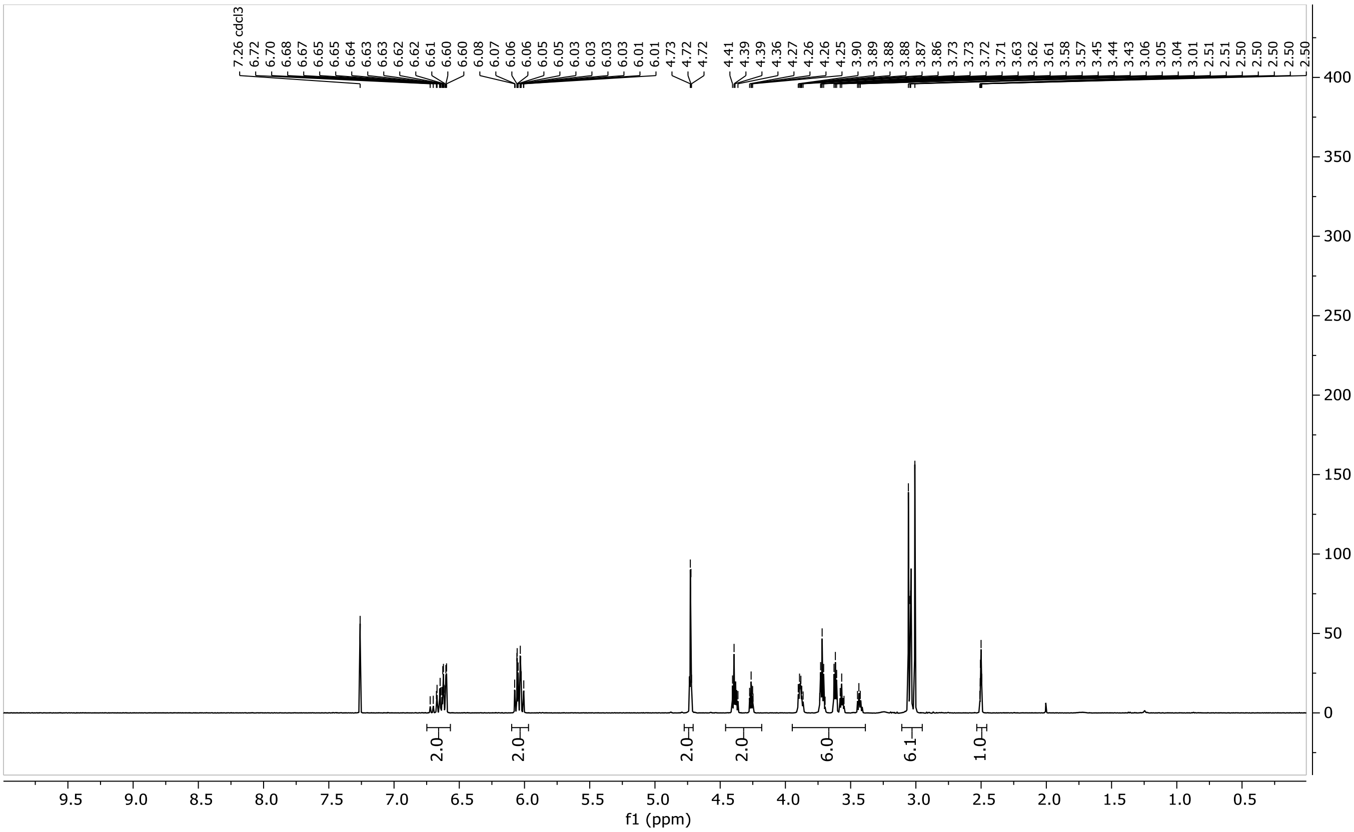

^{13}C NMR (126 MHz, $CDCl_3$)

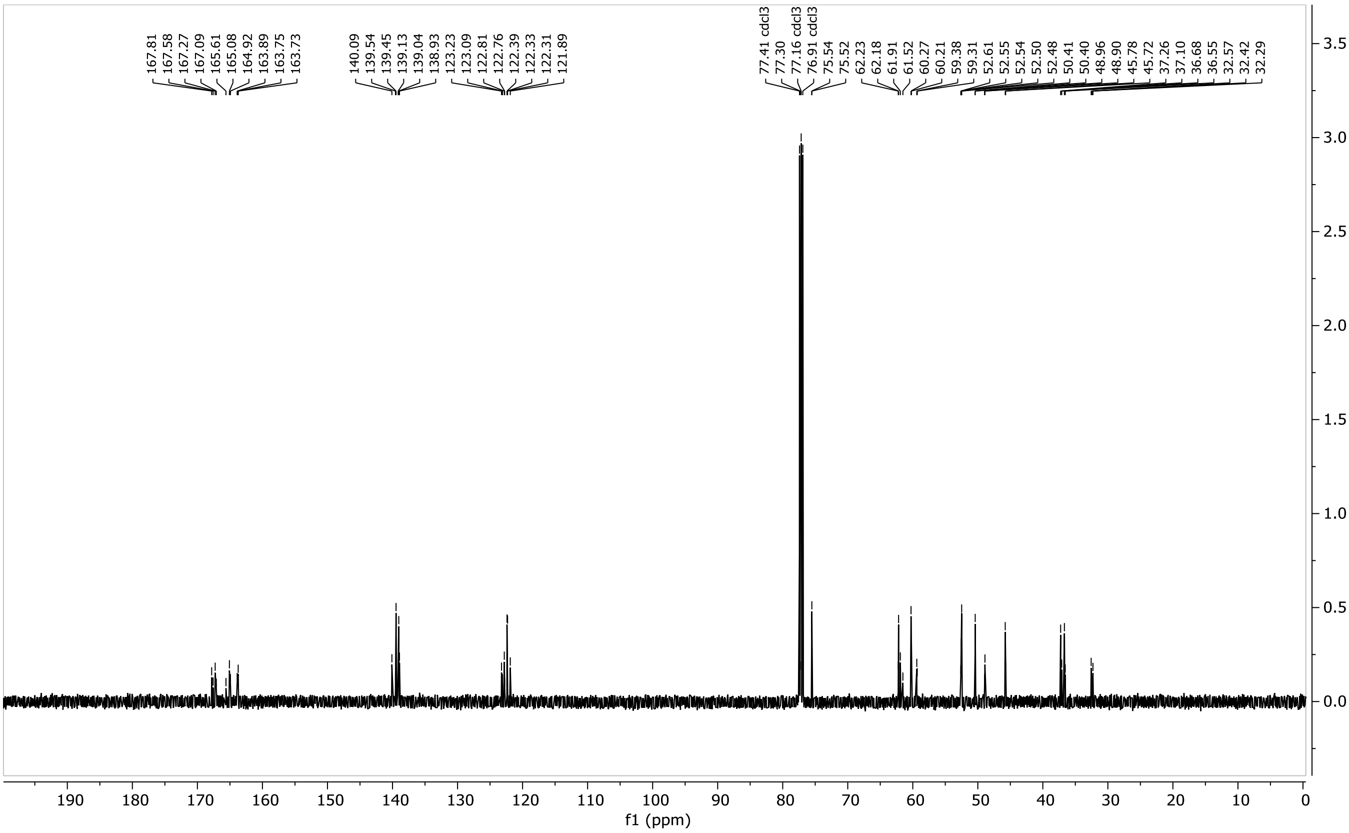

Z-31

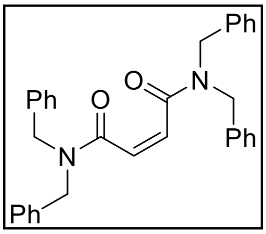

^{1}H NMR (600 MHz, $CDCl_3$)

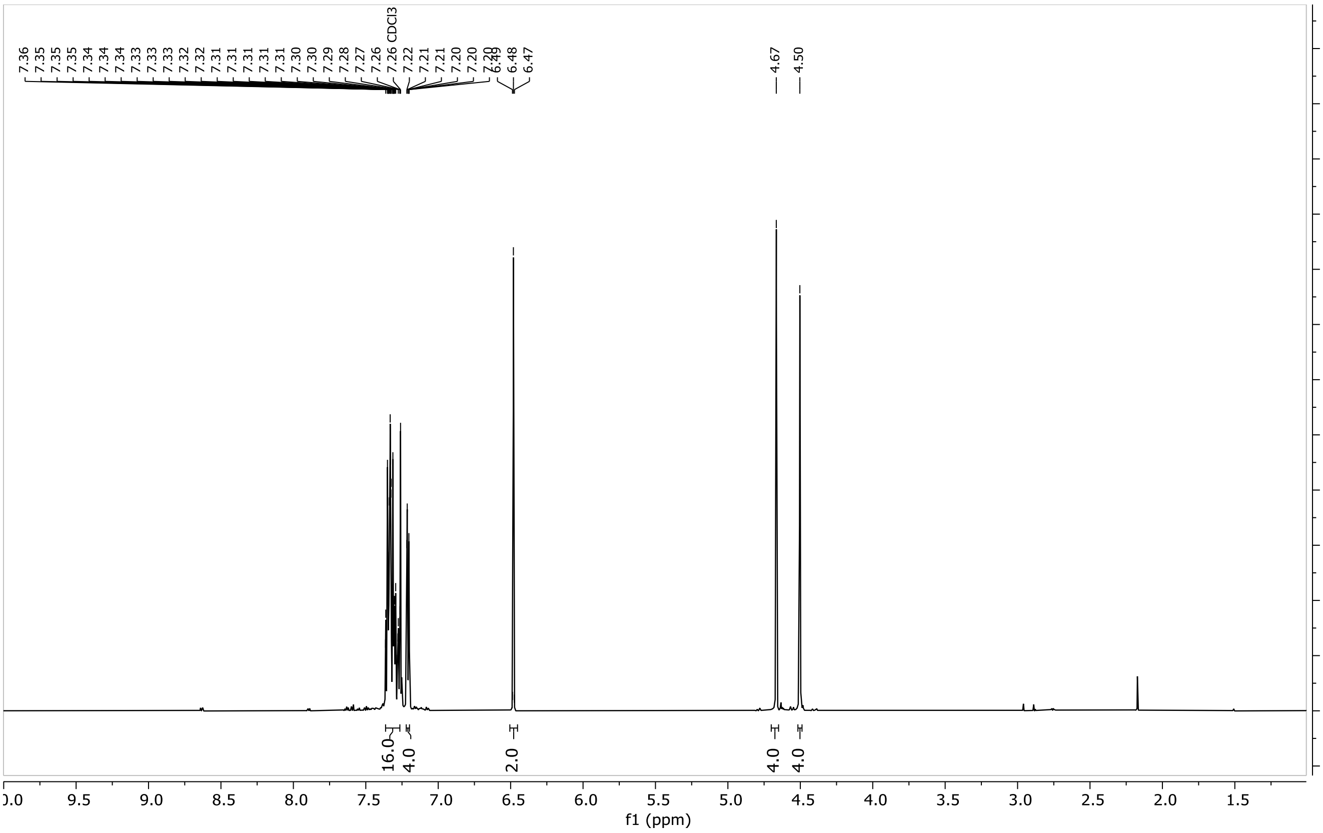

^{13}C NMR (151 MHz, $CDCl_3$)

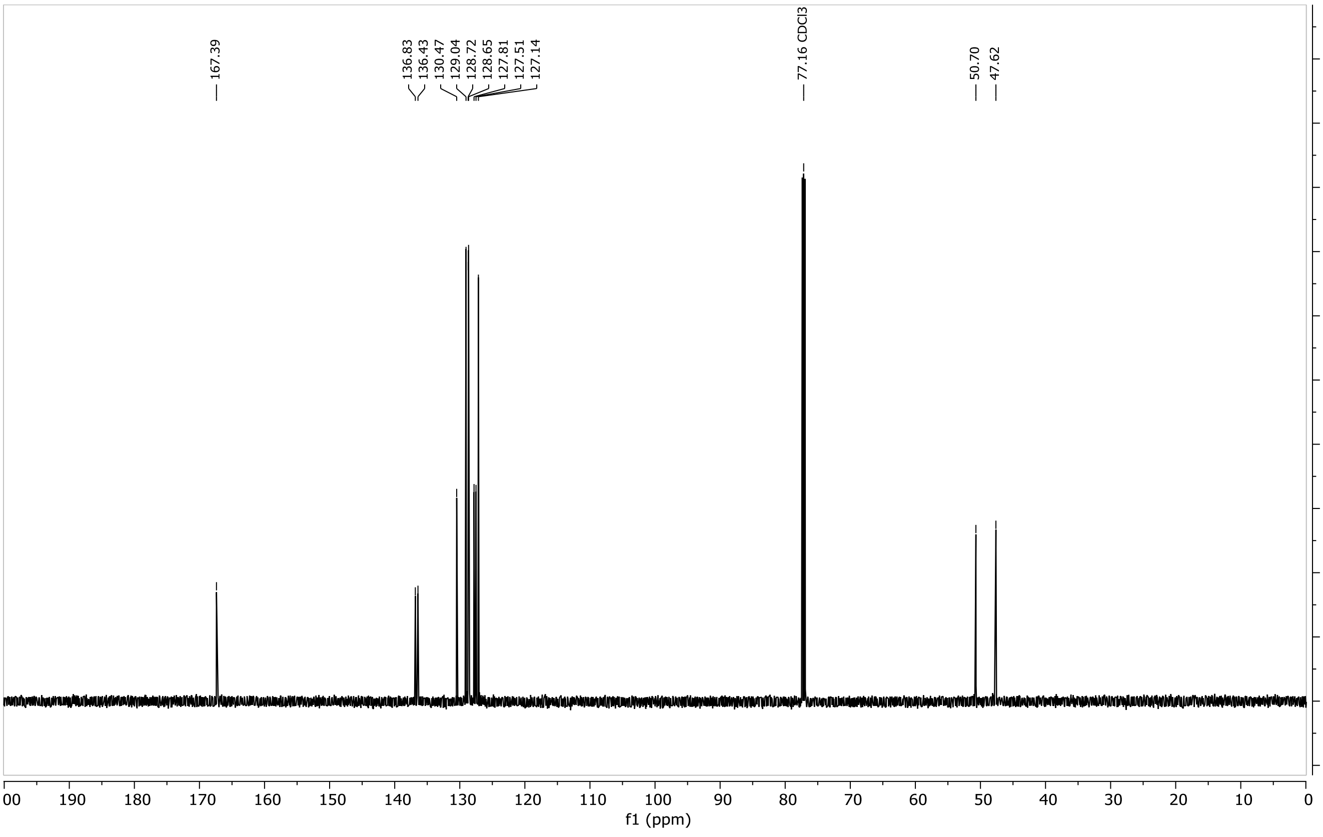

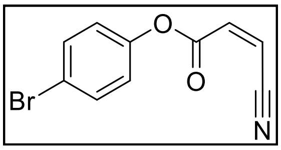

Z-32

^{1}H NMR (500 MHz, $CDCl_3$)

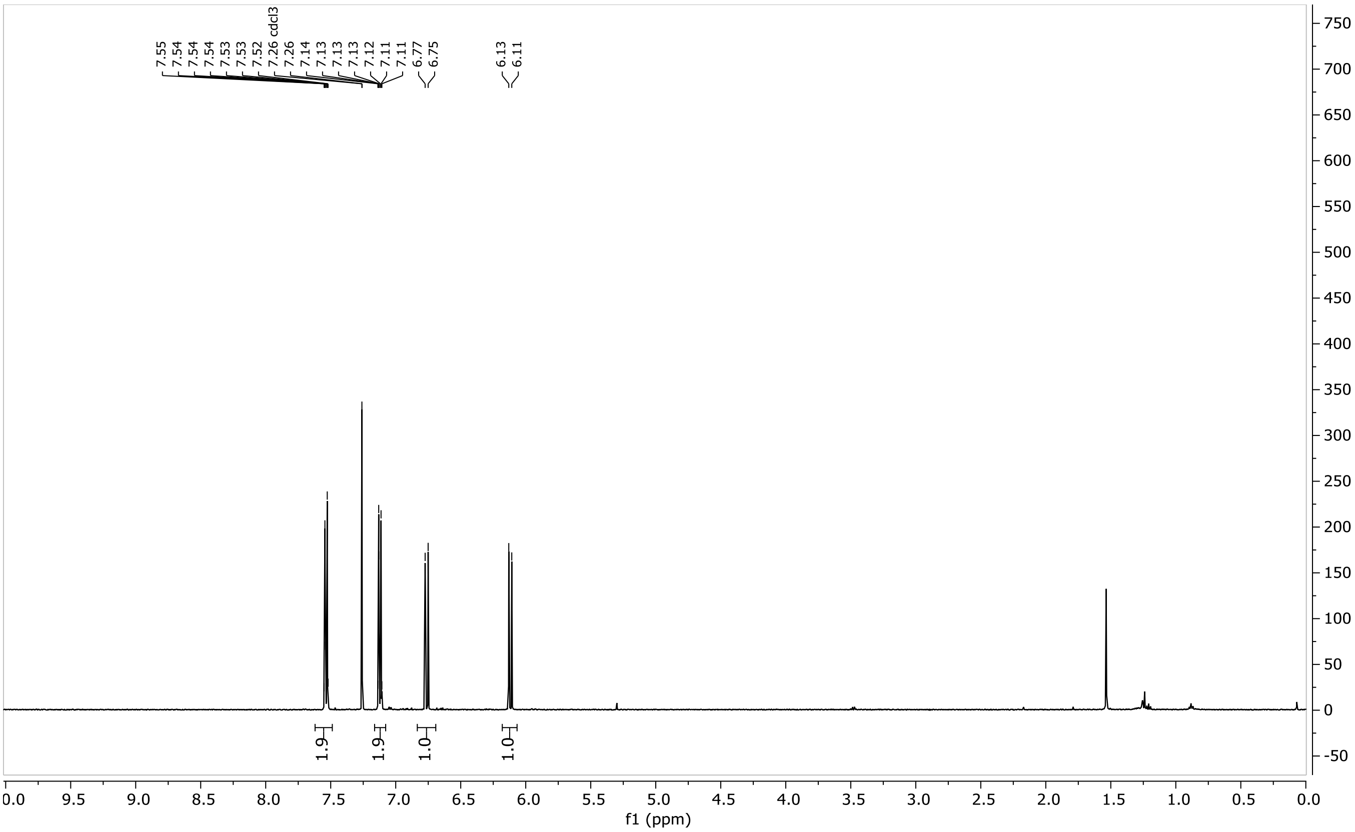

^{13}C NMR (126 MHz, $CDCl_3$)

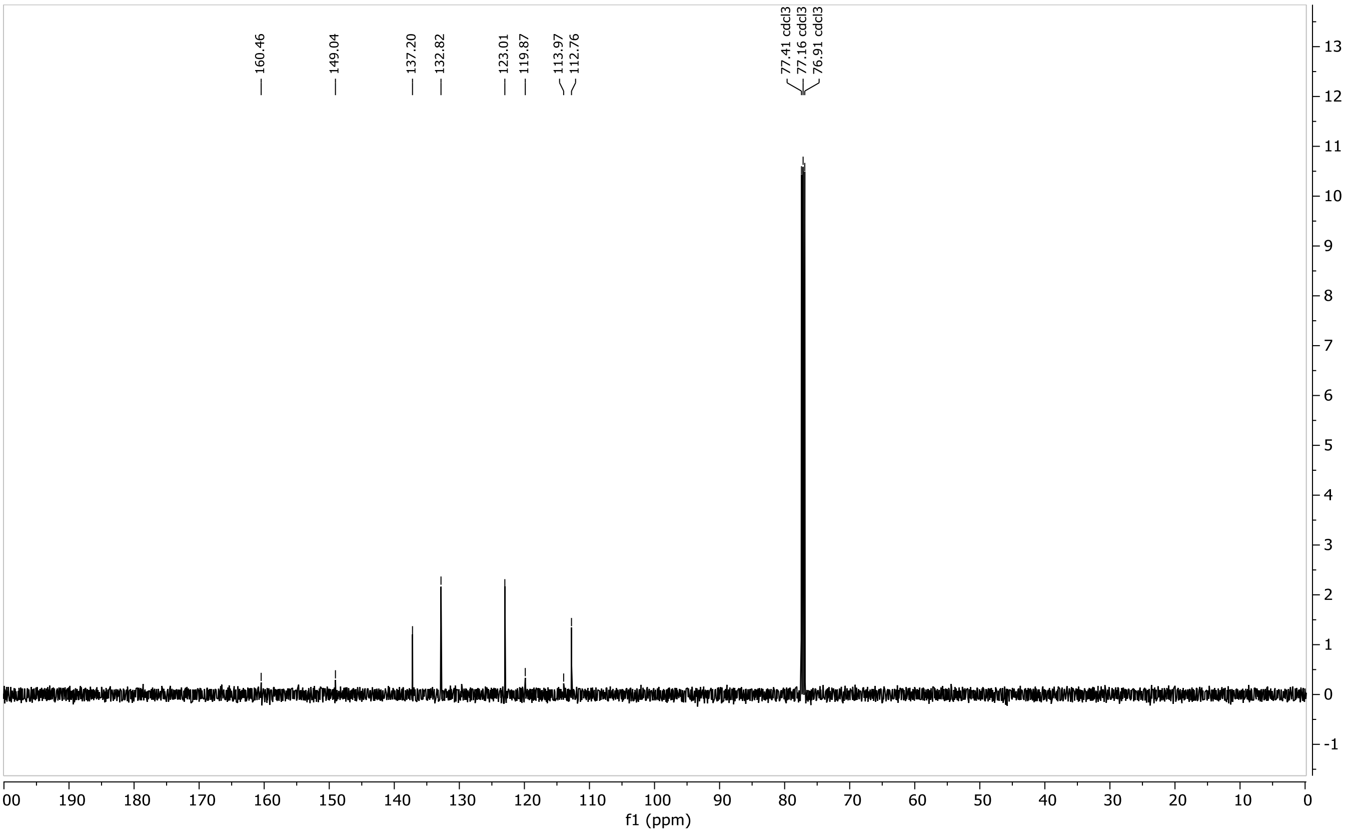

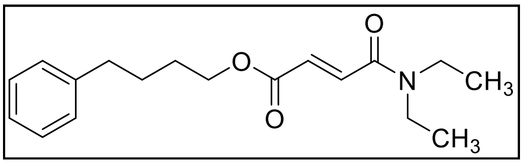

E-1

^{1}H NMR (500 MHz, $CDCl_3$)

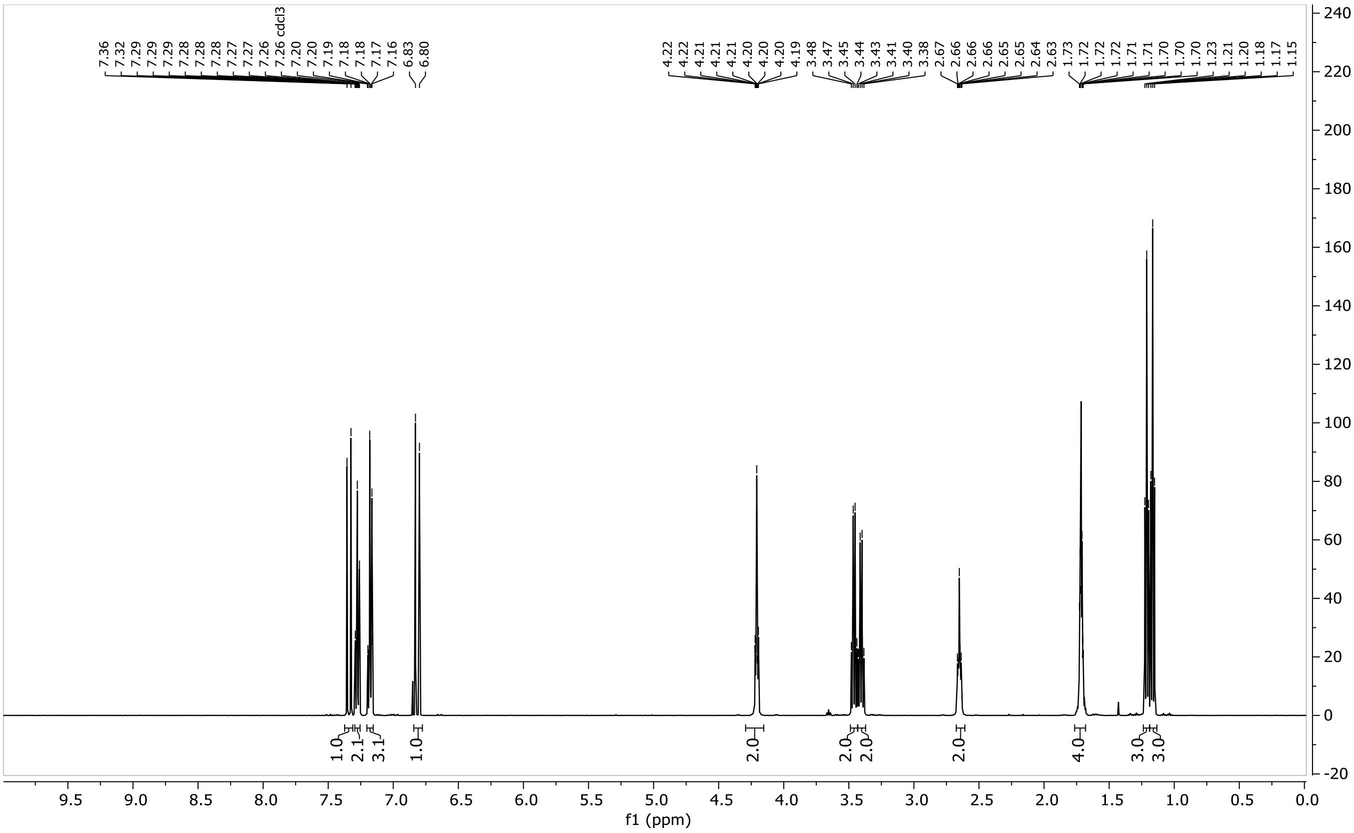

^{13}C NMR (126 MHz, $CDCl_3$)

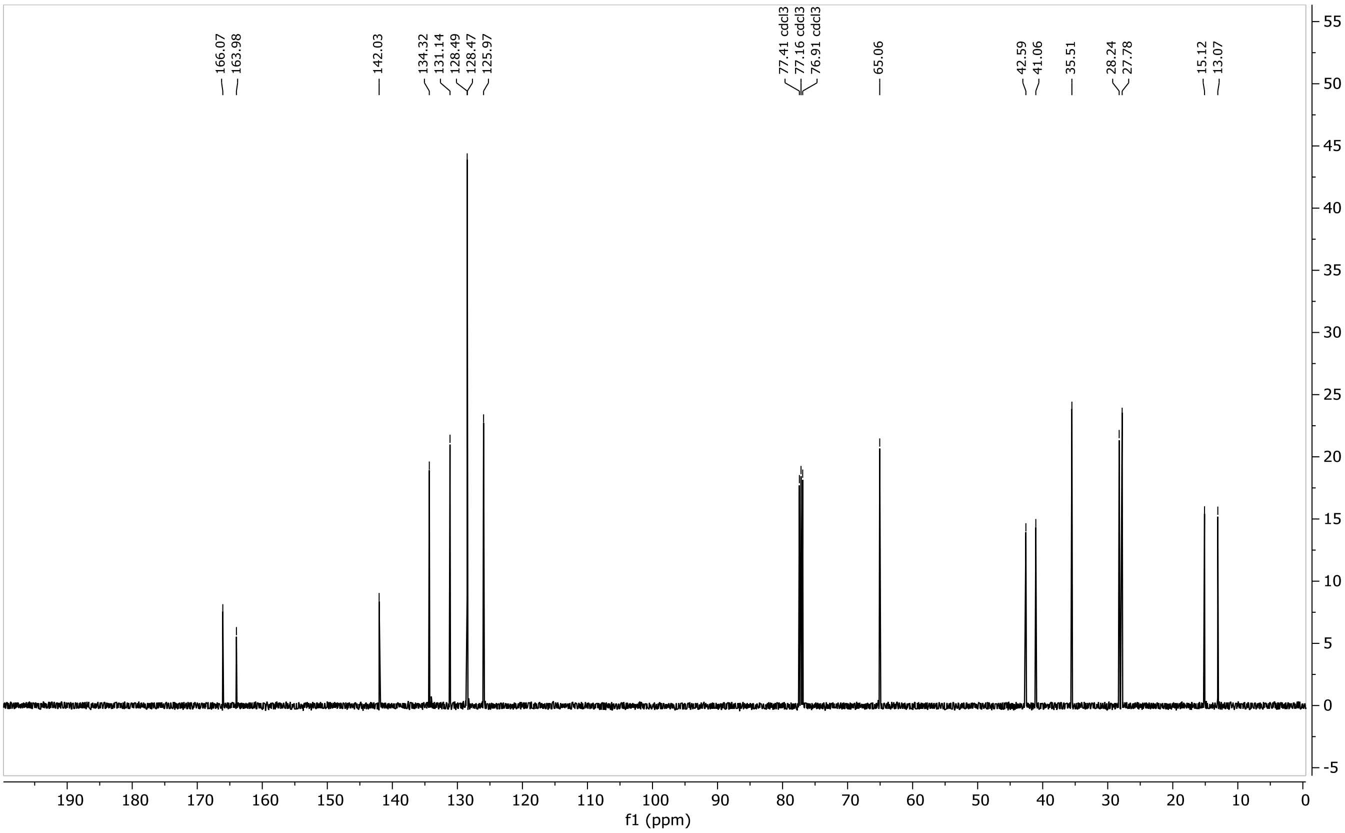

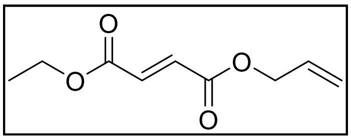

E-3

^{1}H NMR (500 MHz, $CDCl_3$)

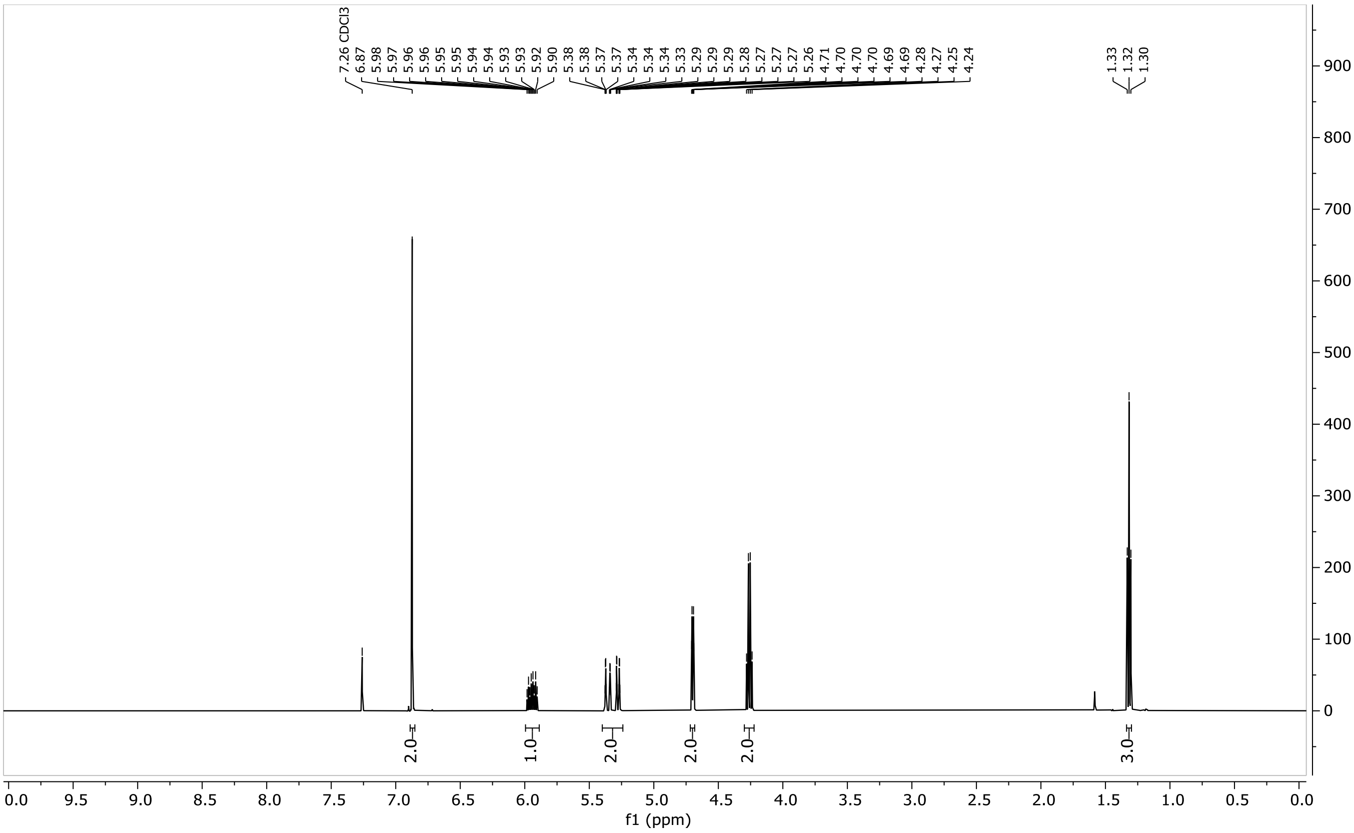

^{13}C NMR (126 MHz, $CDCl_3$)

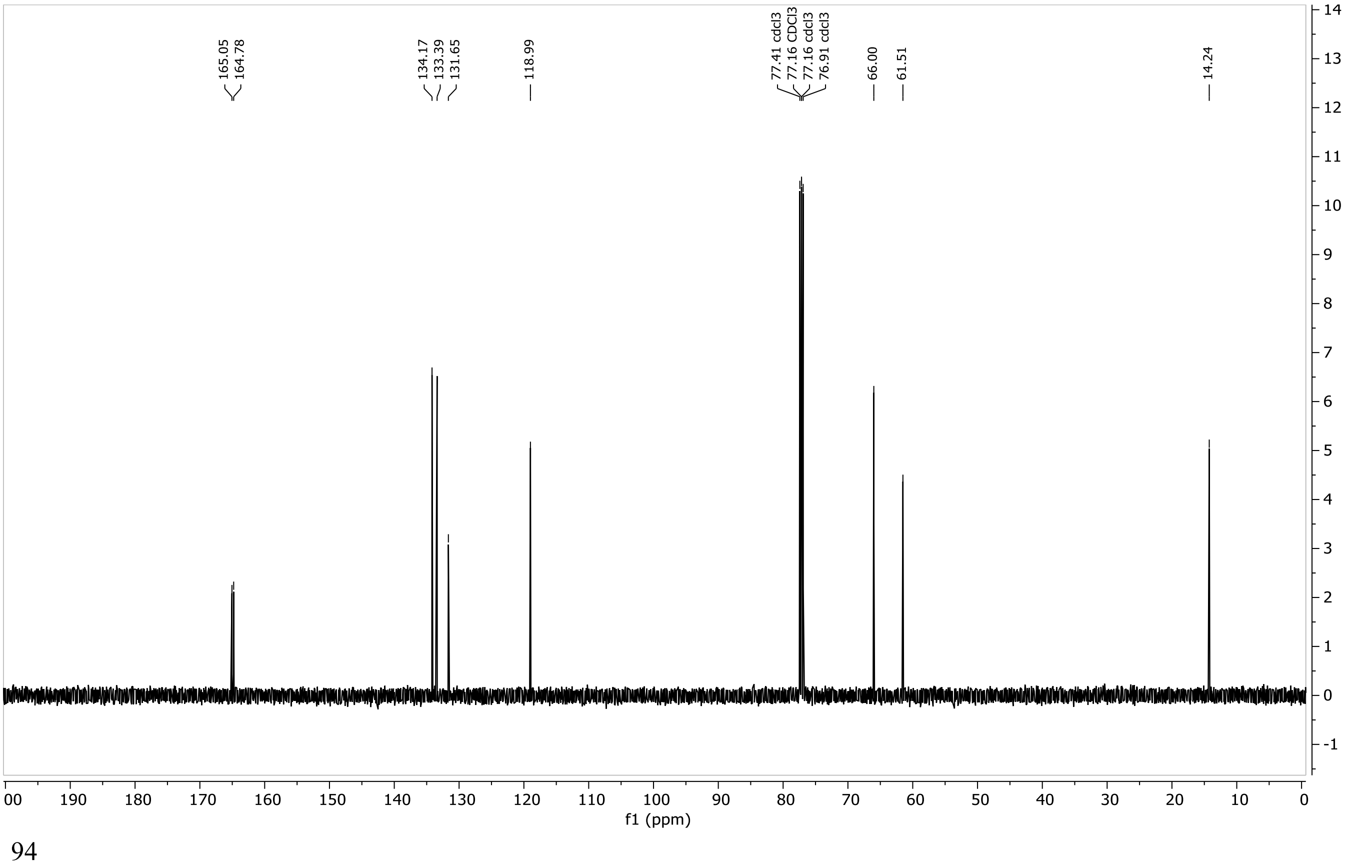

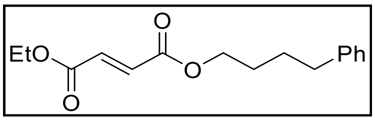

E-4

^{1}H NMR (500 MHz, $CDCl_3$)

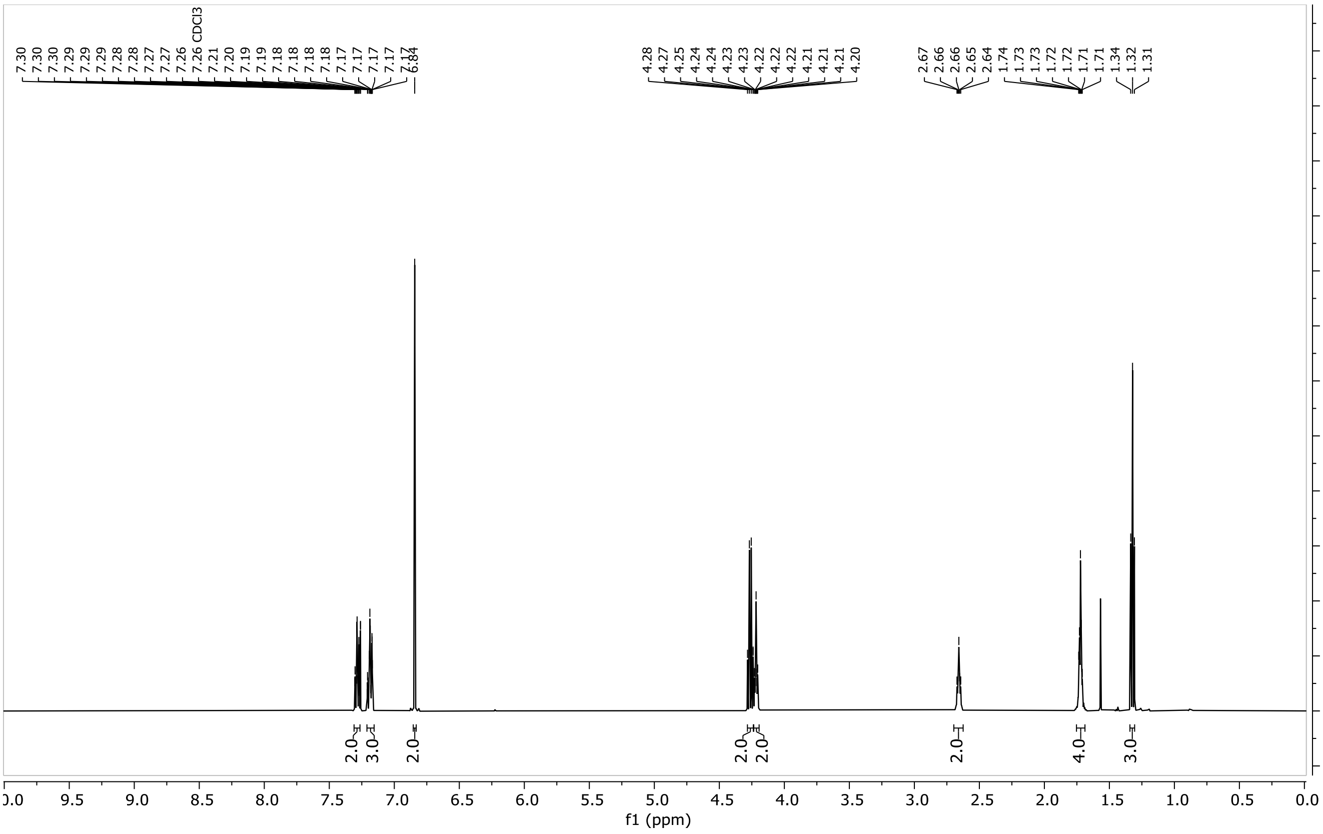

^{13}C NMR (126 MHz, $CDCl_3$)

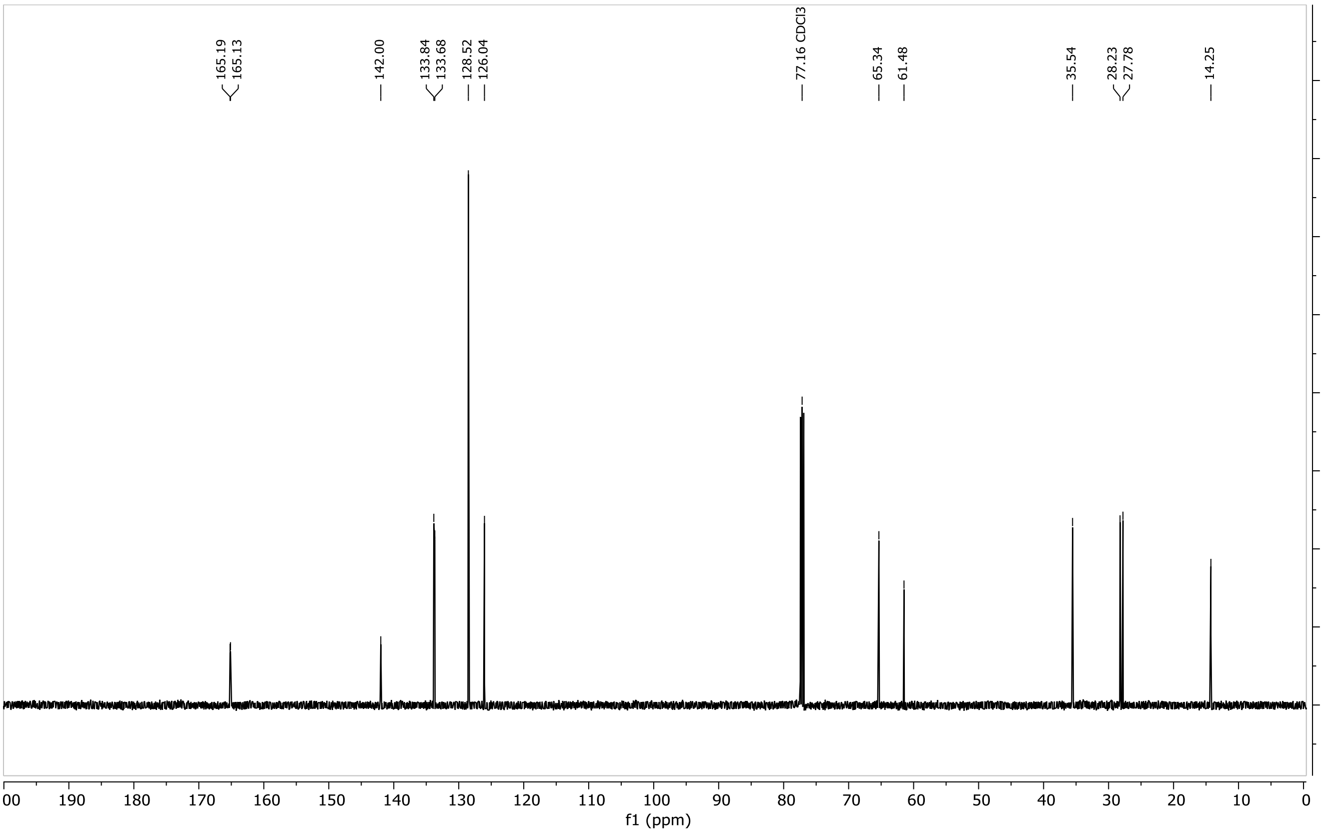

E-6

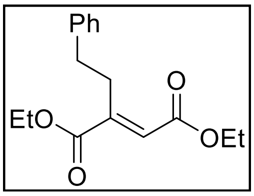

^{1}H NMR (600 MHz, $CDCl_3$)

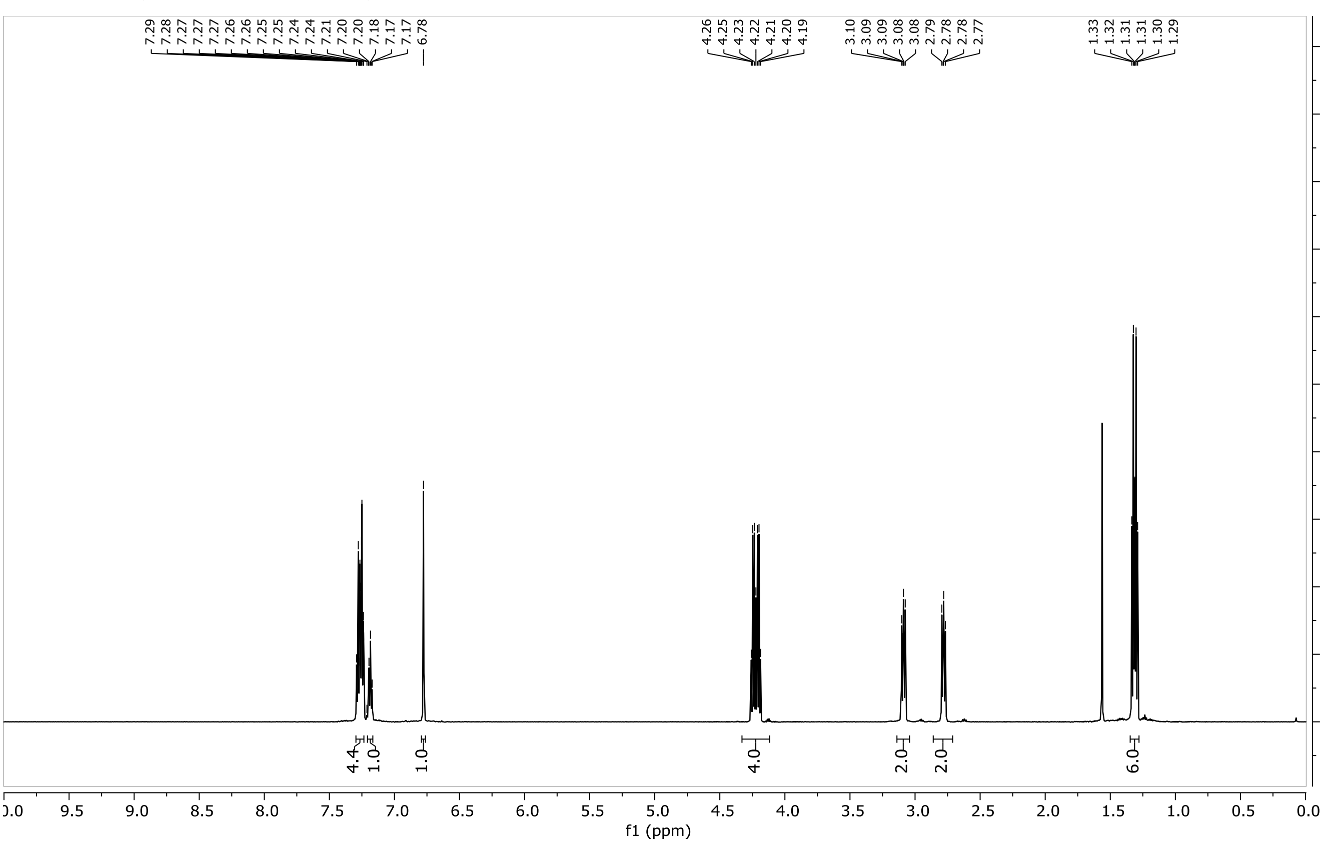

^{13}C NMR (151 MHz, $CDCl_3$)

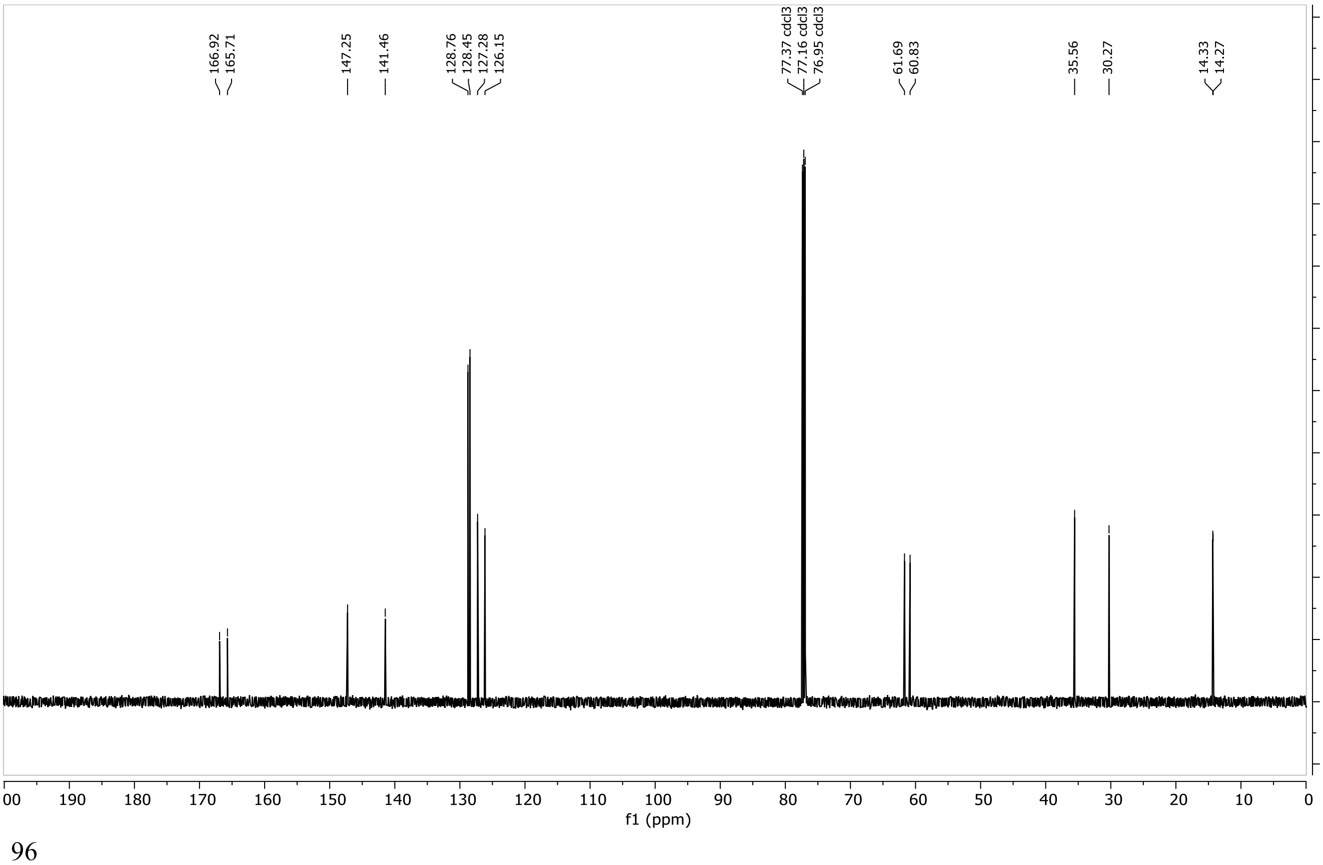

E-7

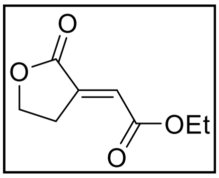

^{1}H NMR (600 MHz, $CDCl_3$)

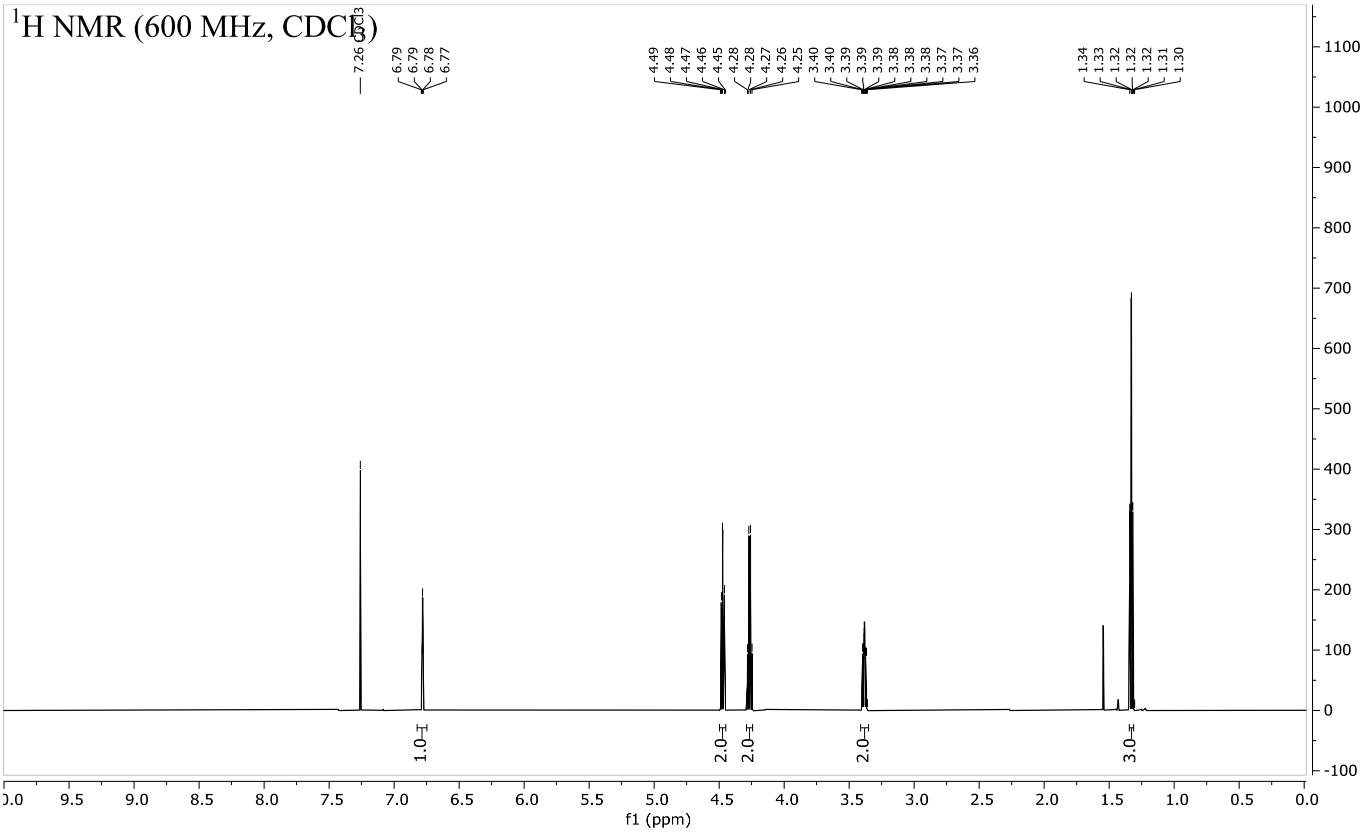

^{13}C NMR (151 MHz, $CDCl_3$)

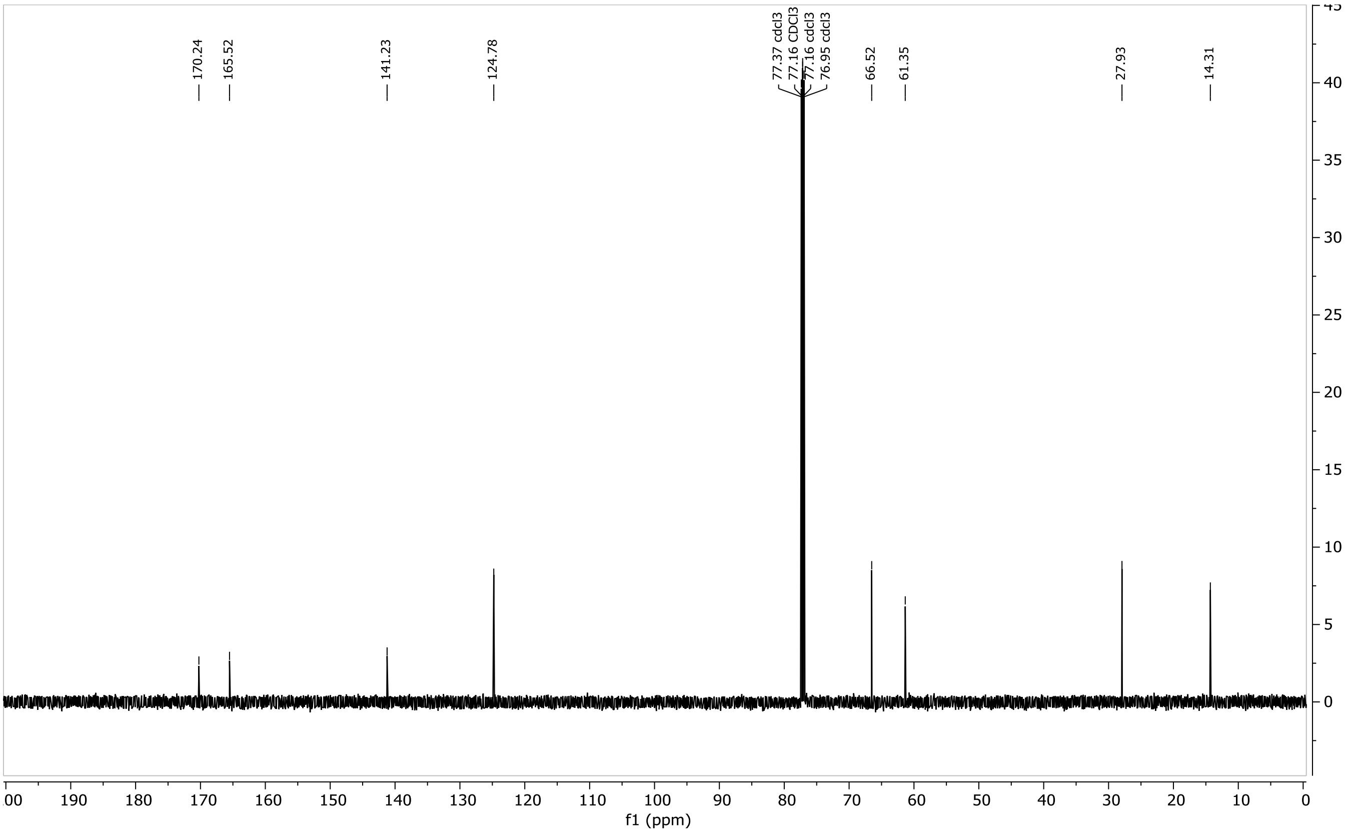

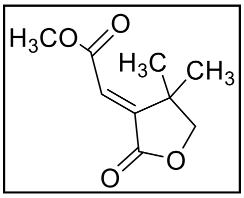

E-8

^{1}H NMR (600 MHz, $CDCl_3$)

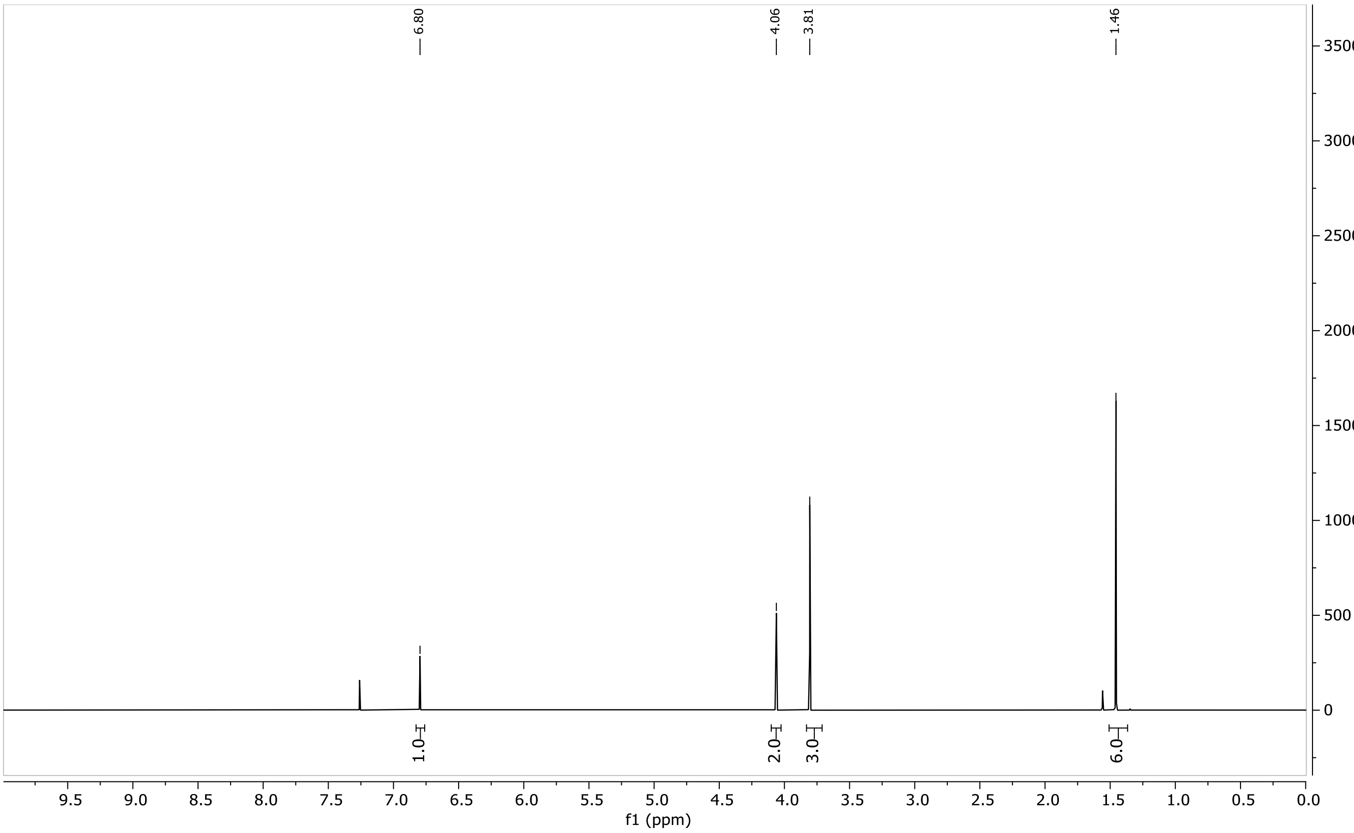

^{13}C NMR (151 MHz, $CDCl_3$)

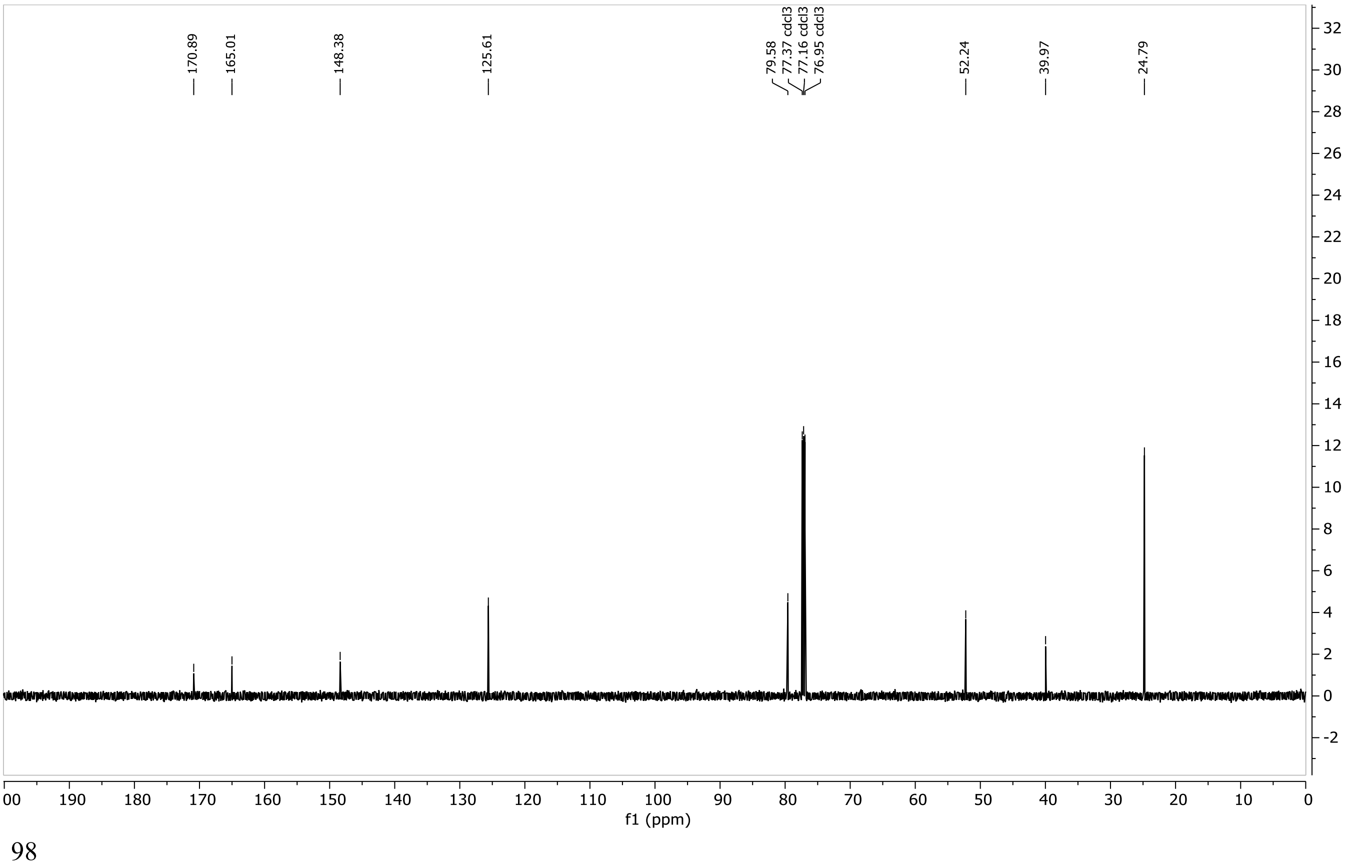

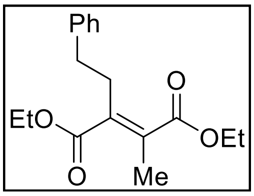

E-9

^{1}H NMR (600 MHz, $CDCl_3$)

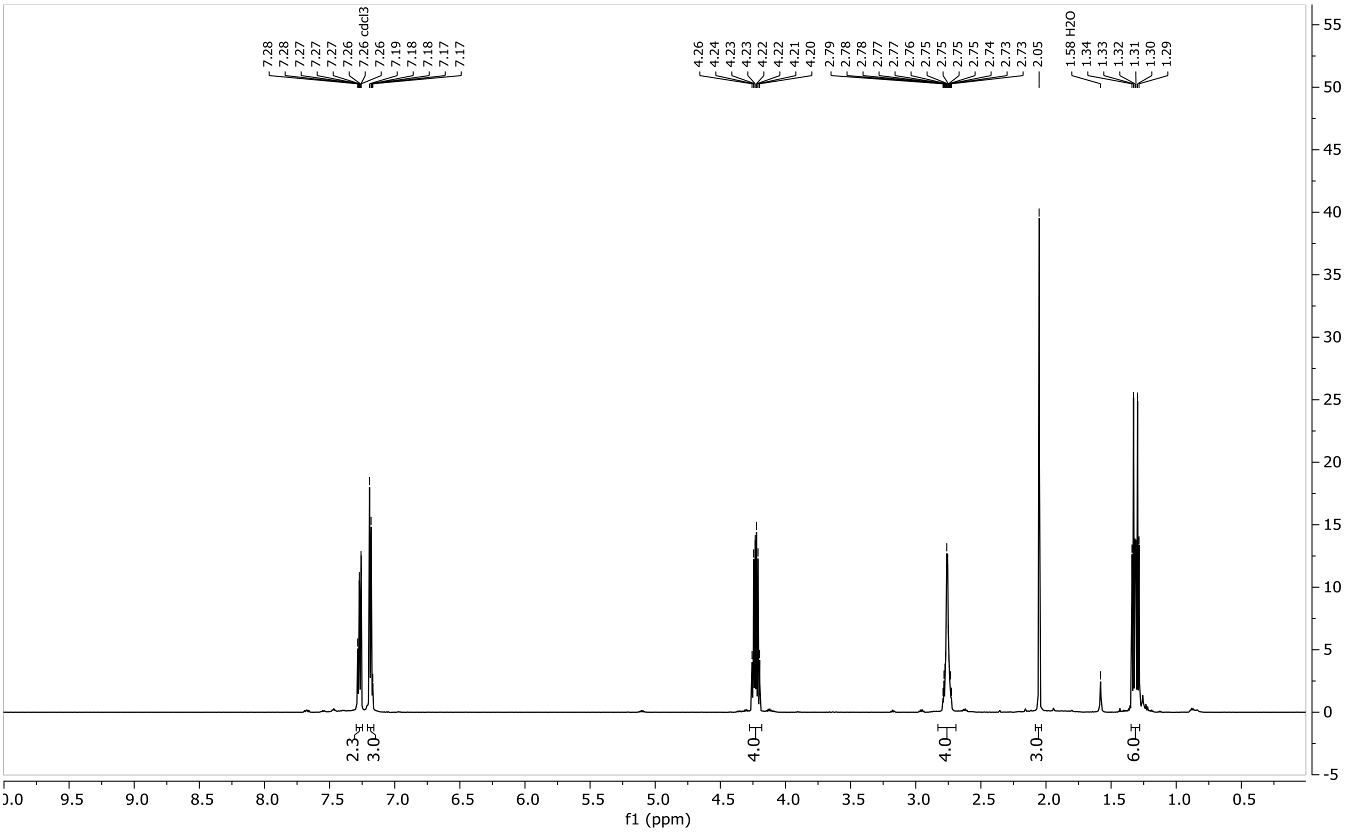

^{13}C NMR (151 MHz, $CDCl_3$)

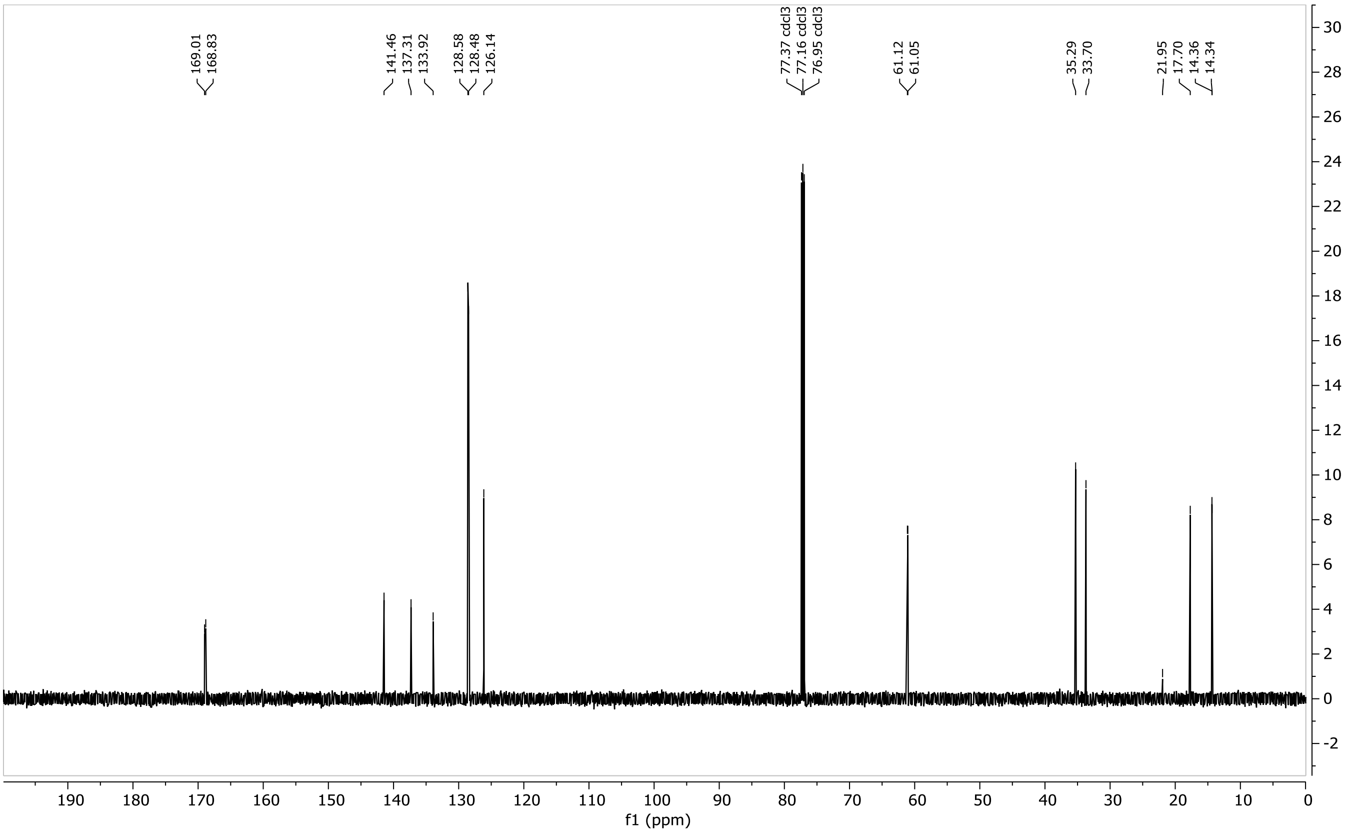

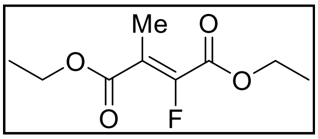

Z-10

^{1}H NMR (600 MHz, $CDCl_3$)

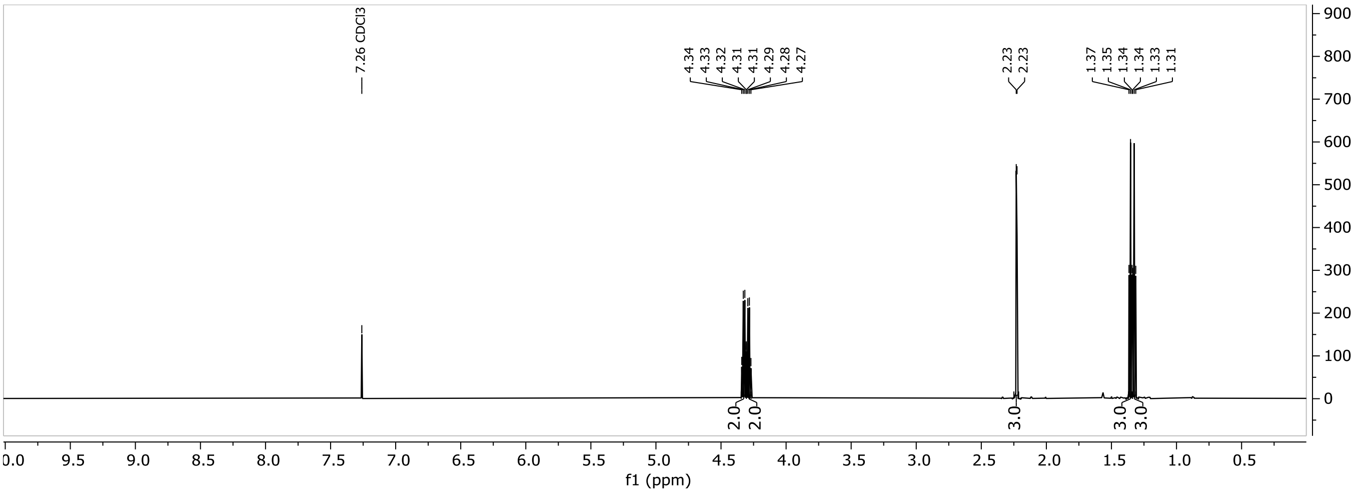

^{13}C-{^{19}F} NMR (151 MHz, $CDCl_3$)

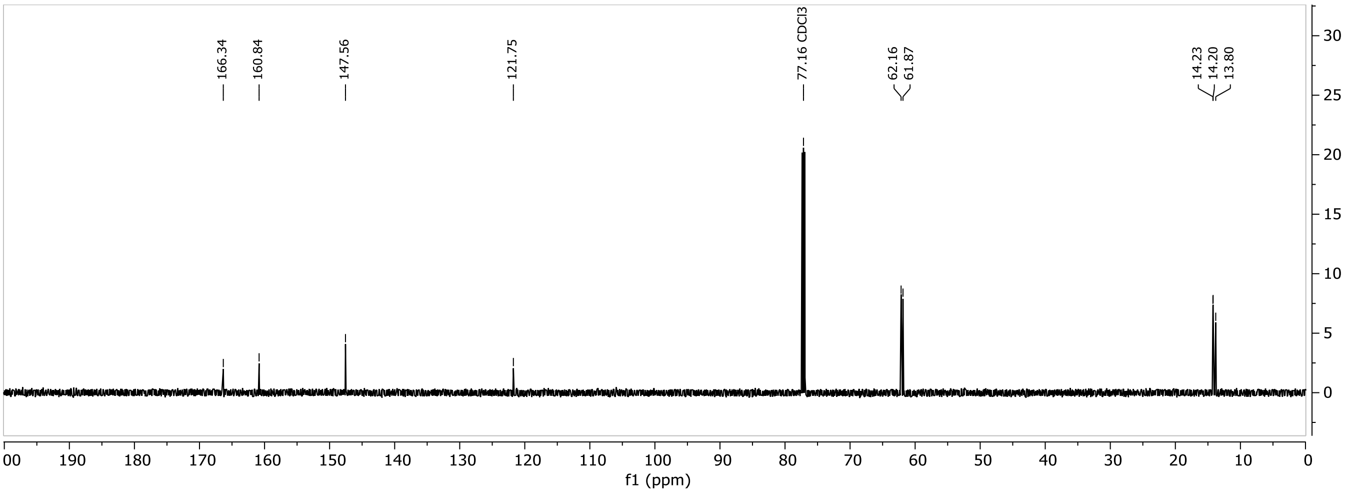

^{19}F NMR (376 MHz, $CDCl_3$)

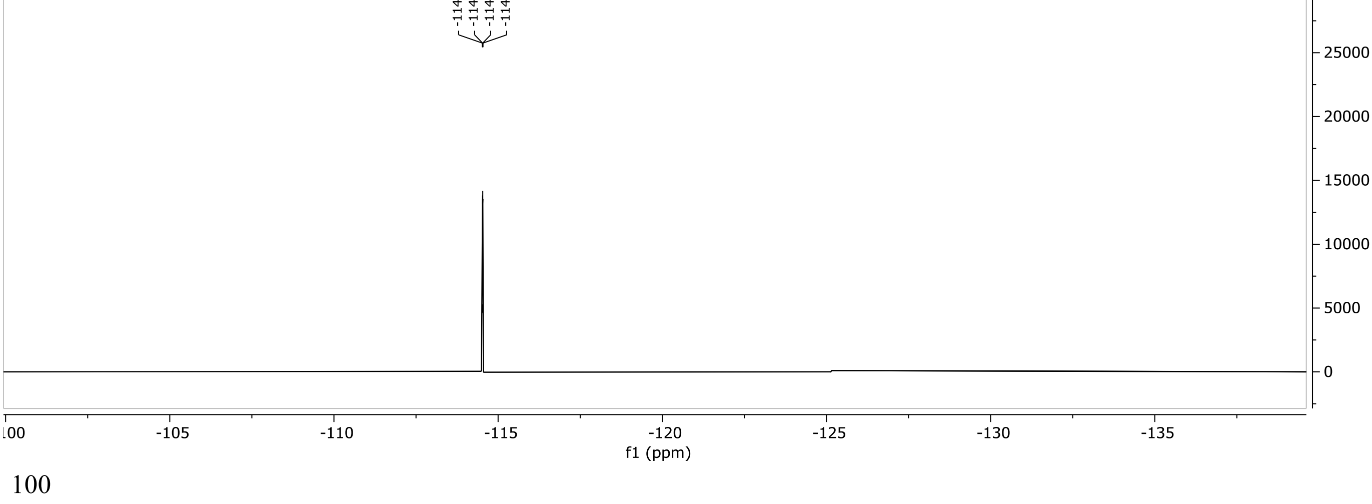

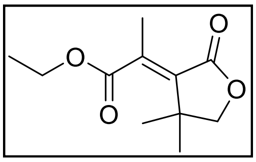

E-11

^{1}H NMR (600 MHz, $CDCl_3$)

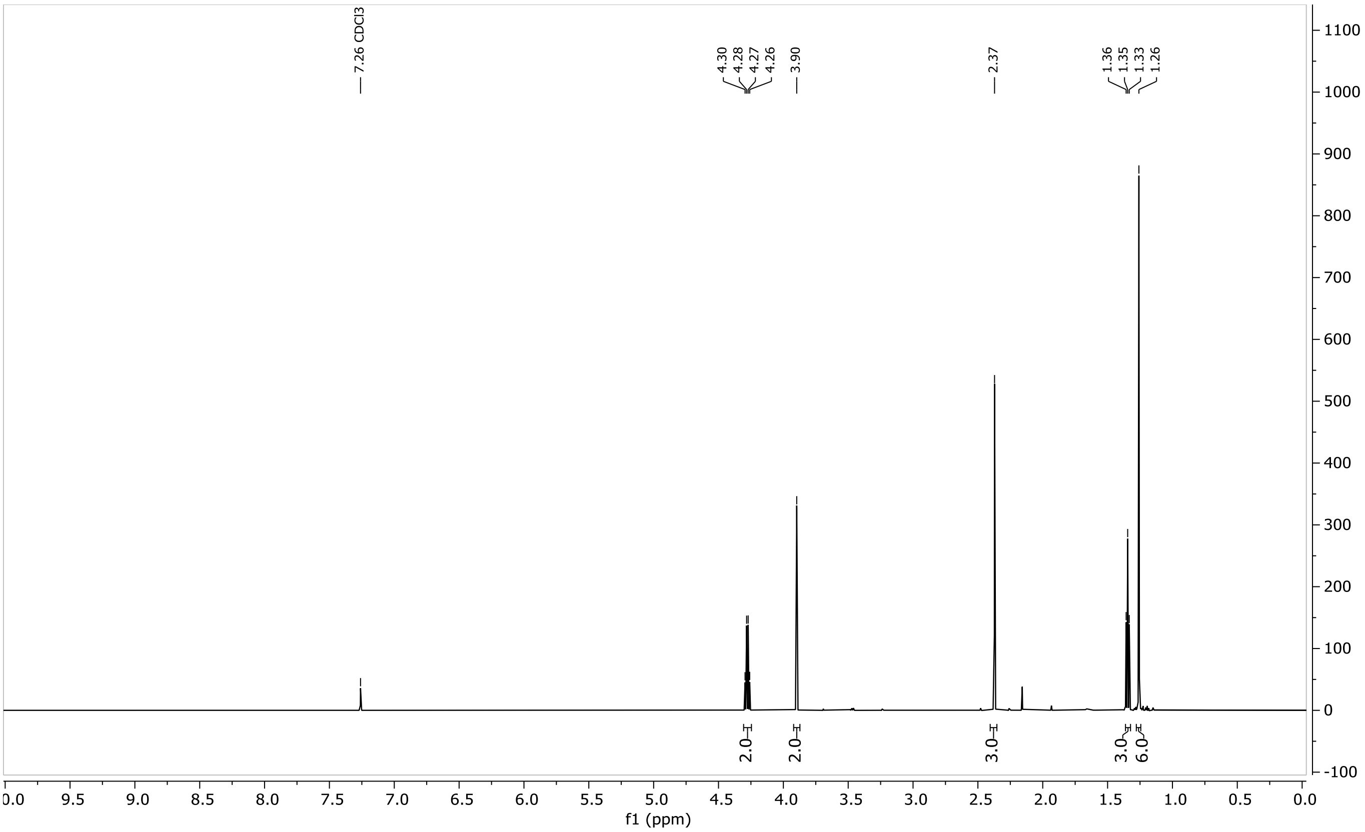

^{13}C NMR (151 MHz, $CDCl_3$)

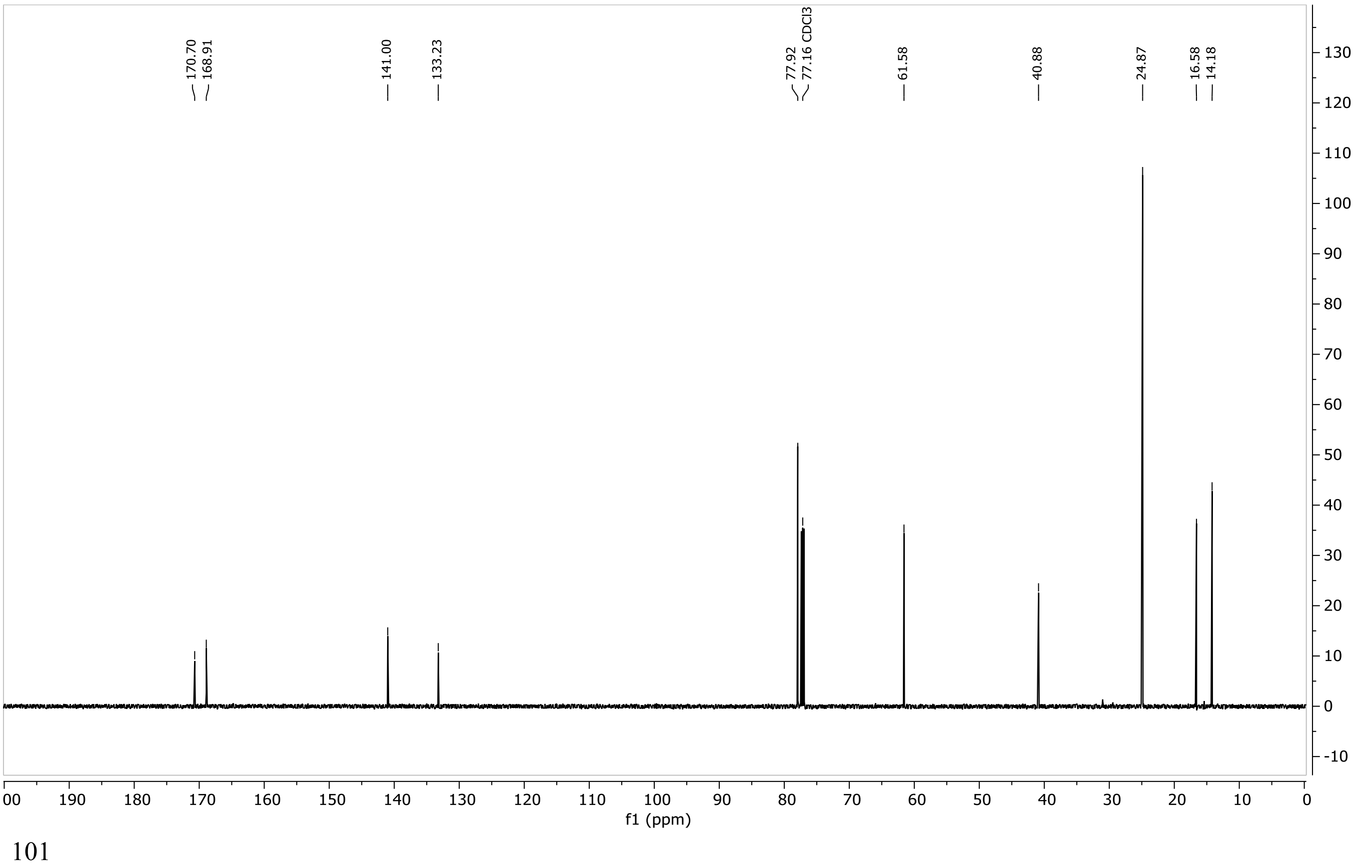

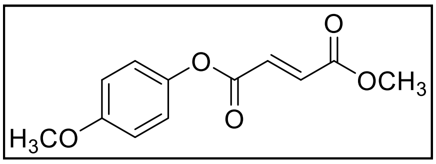

E-12

^{1}H NMR (500 MHz, $CDCl_3$)

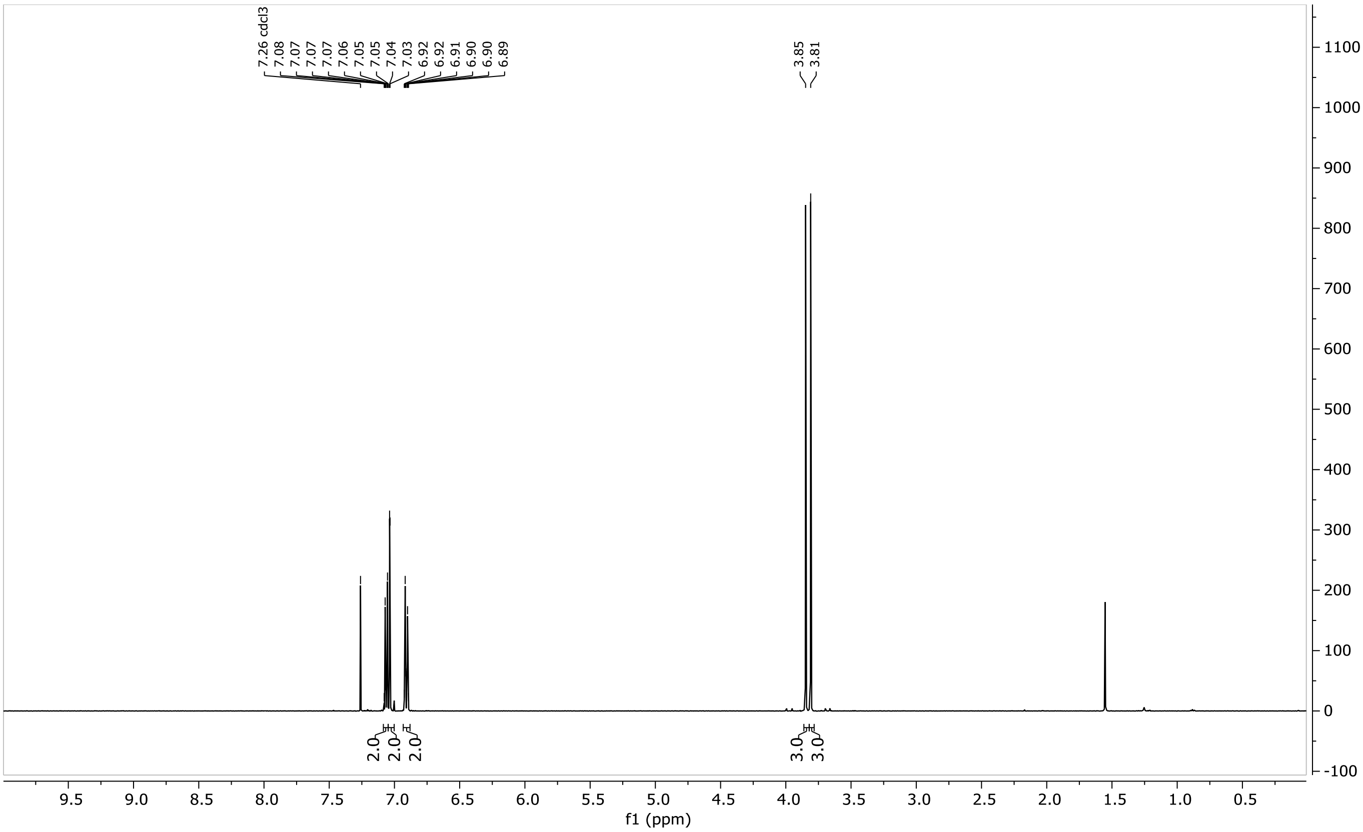

^{13}C NMR (126 MHz, $CDCl_3$)

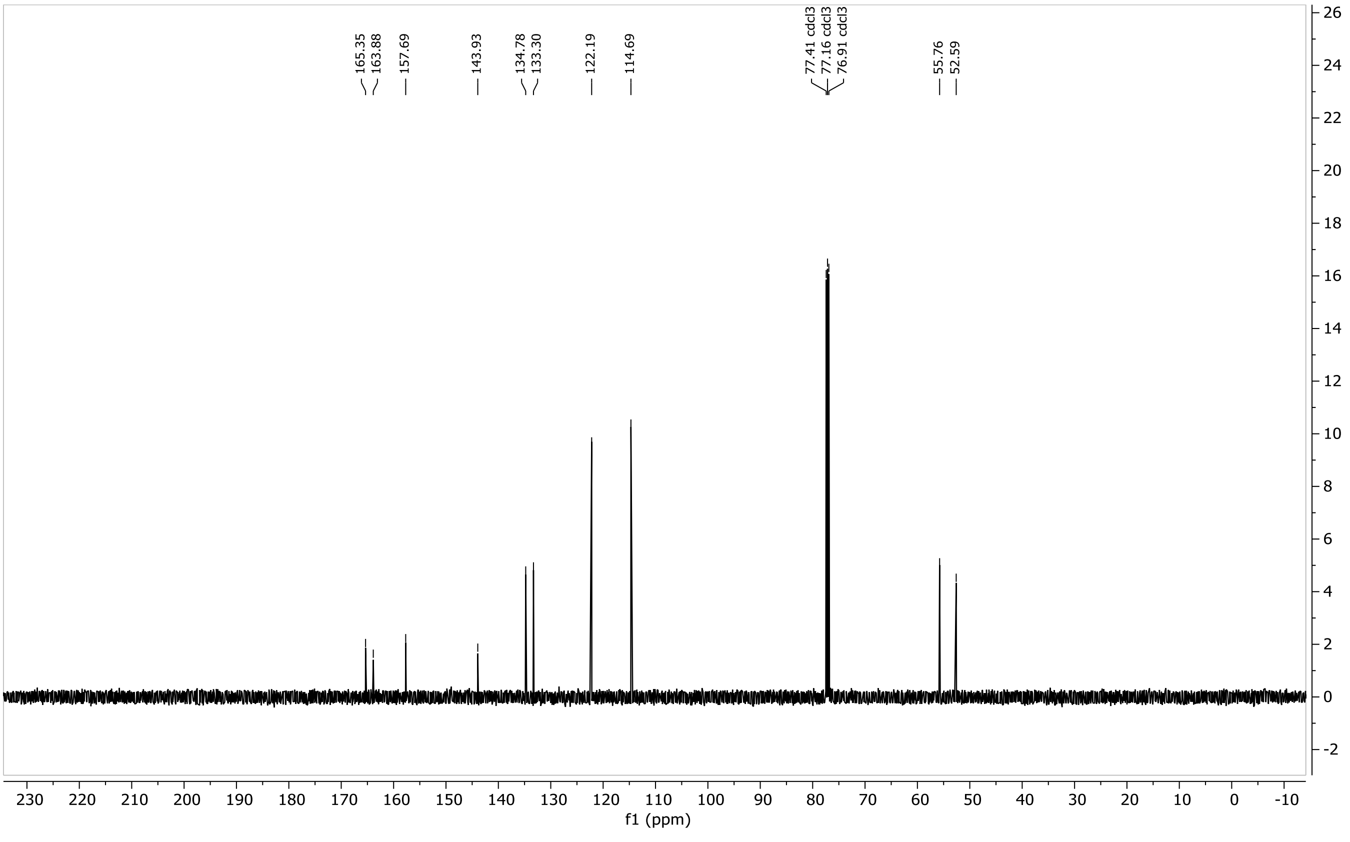

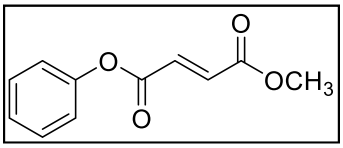

E-13

^{1}H NMR (600 MHz, $CDCl_3$)

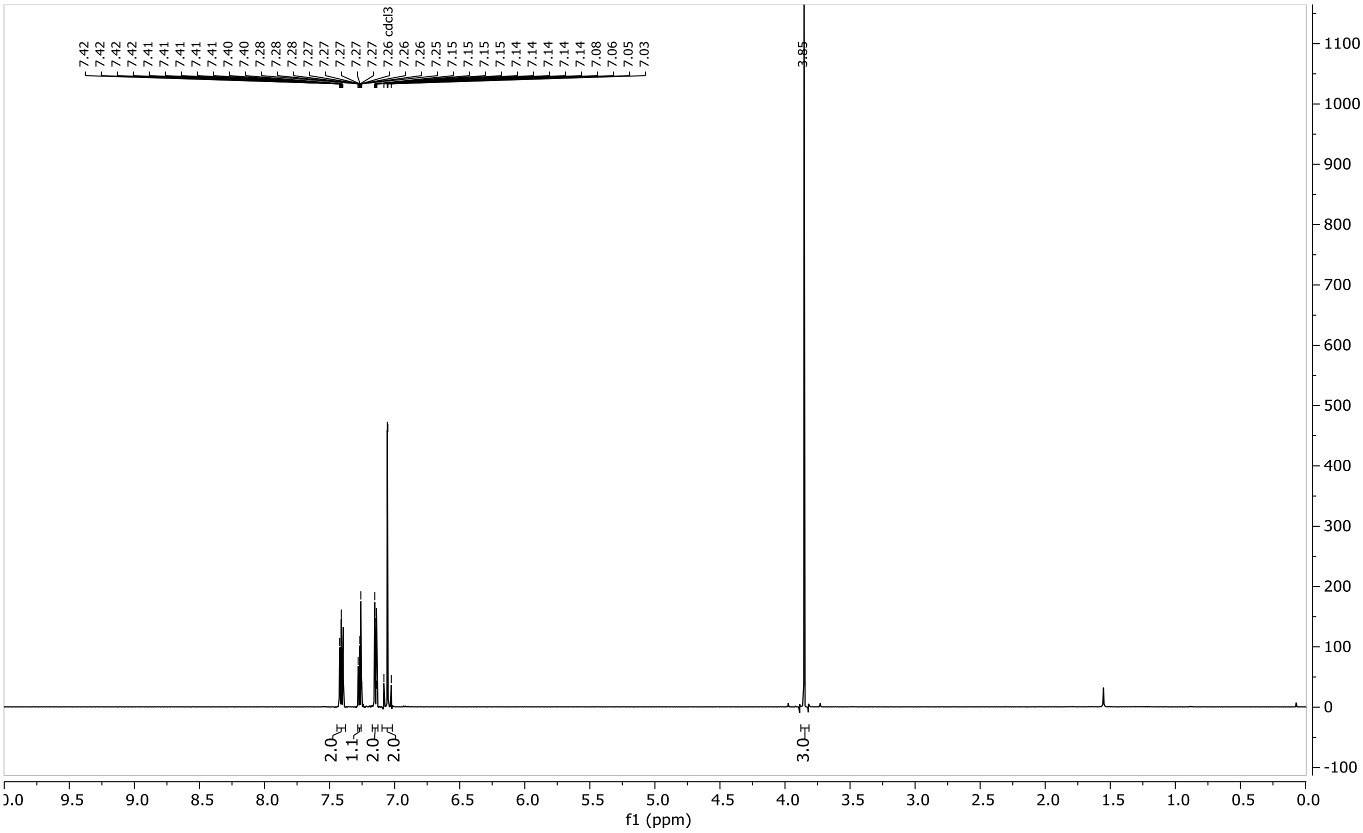

^{13}C NMR (151 MHz, $CDCl_3$)

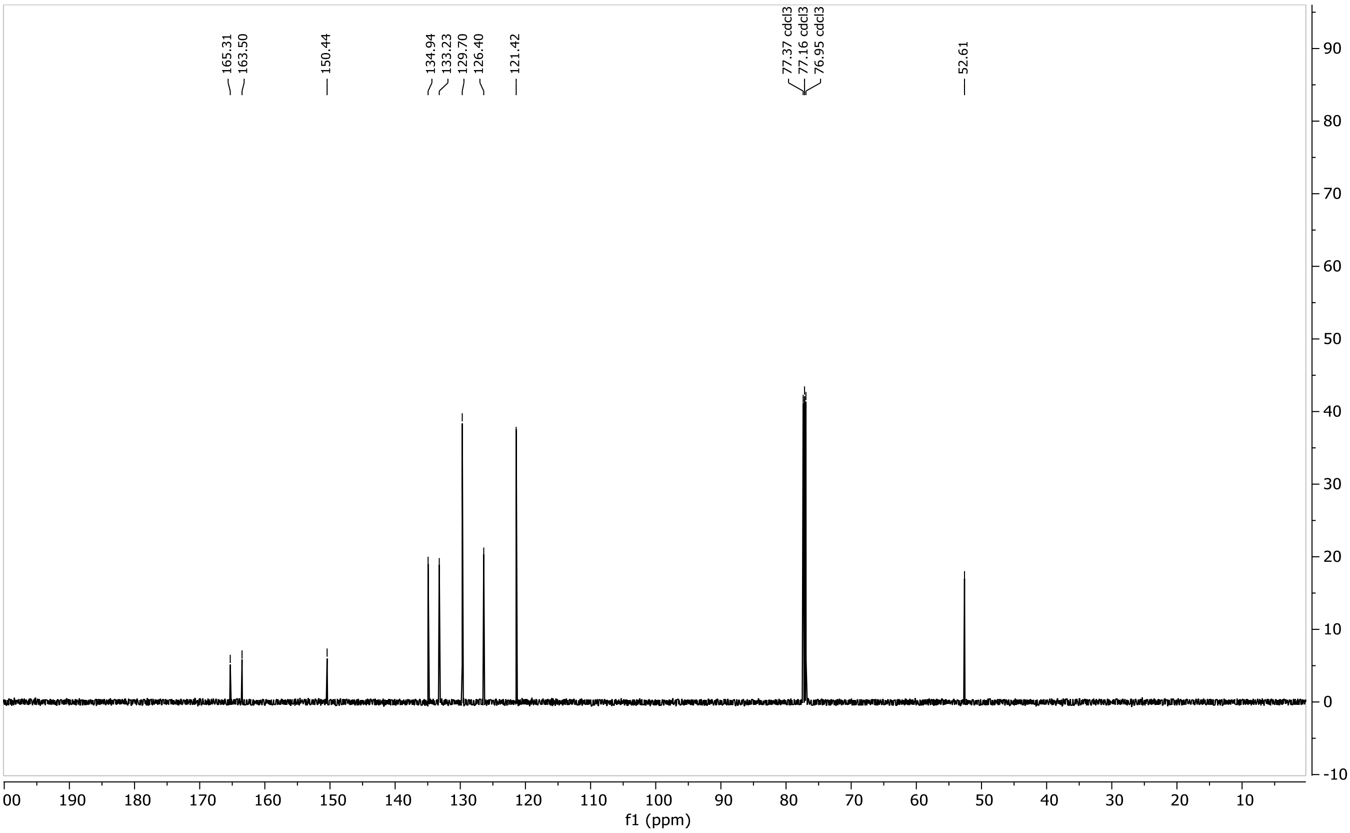

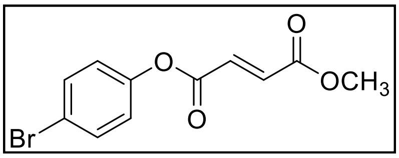

E-14

^{1}H NMR (500 MHz, $CDCl_3$)

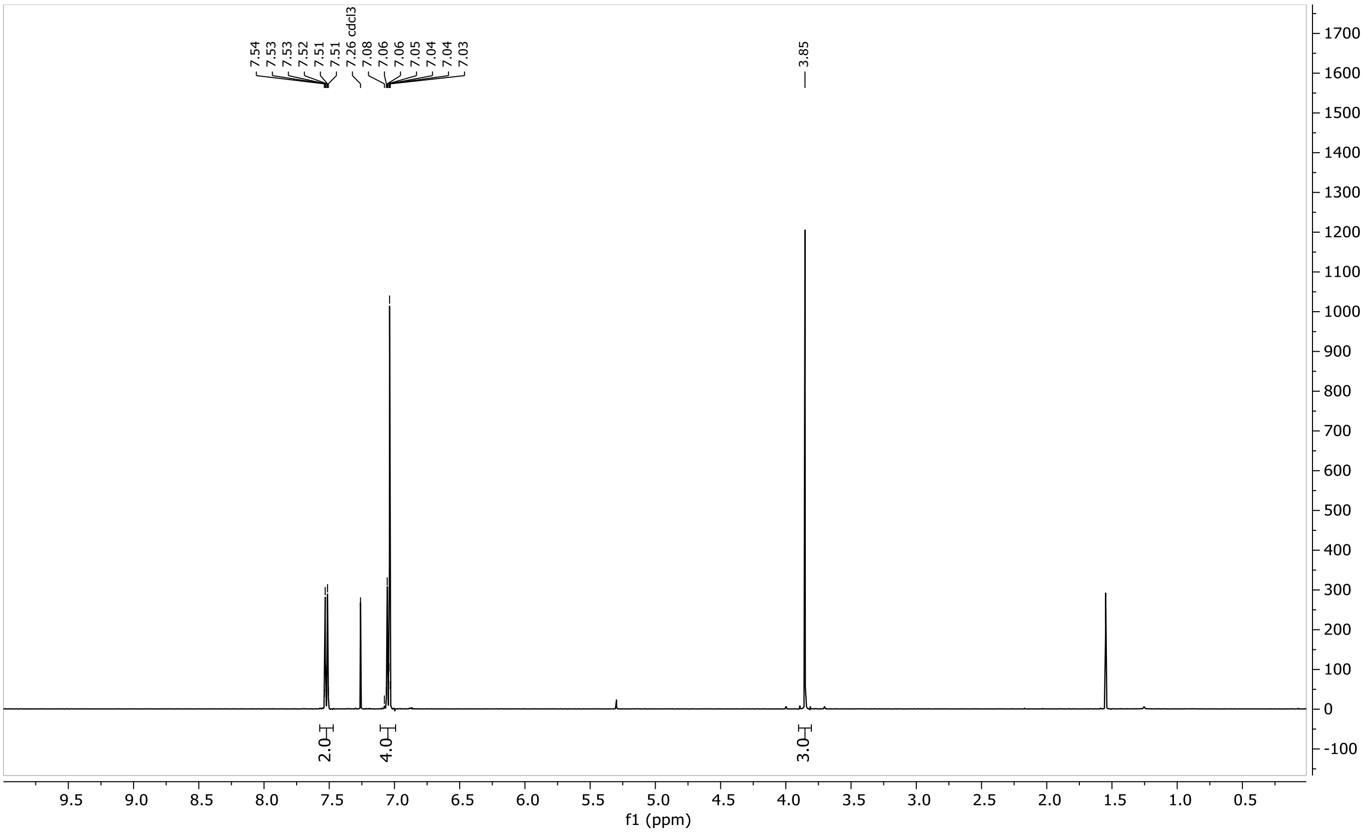

^{13}C NMR (126 MHz, $CDCl_3$)

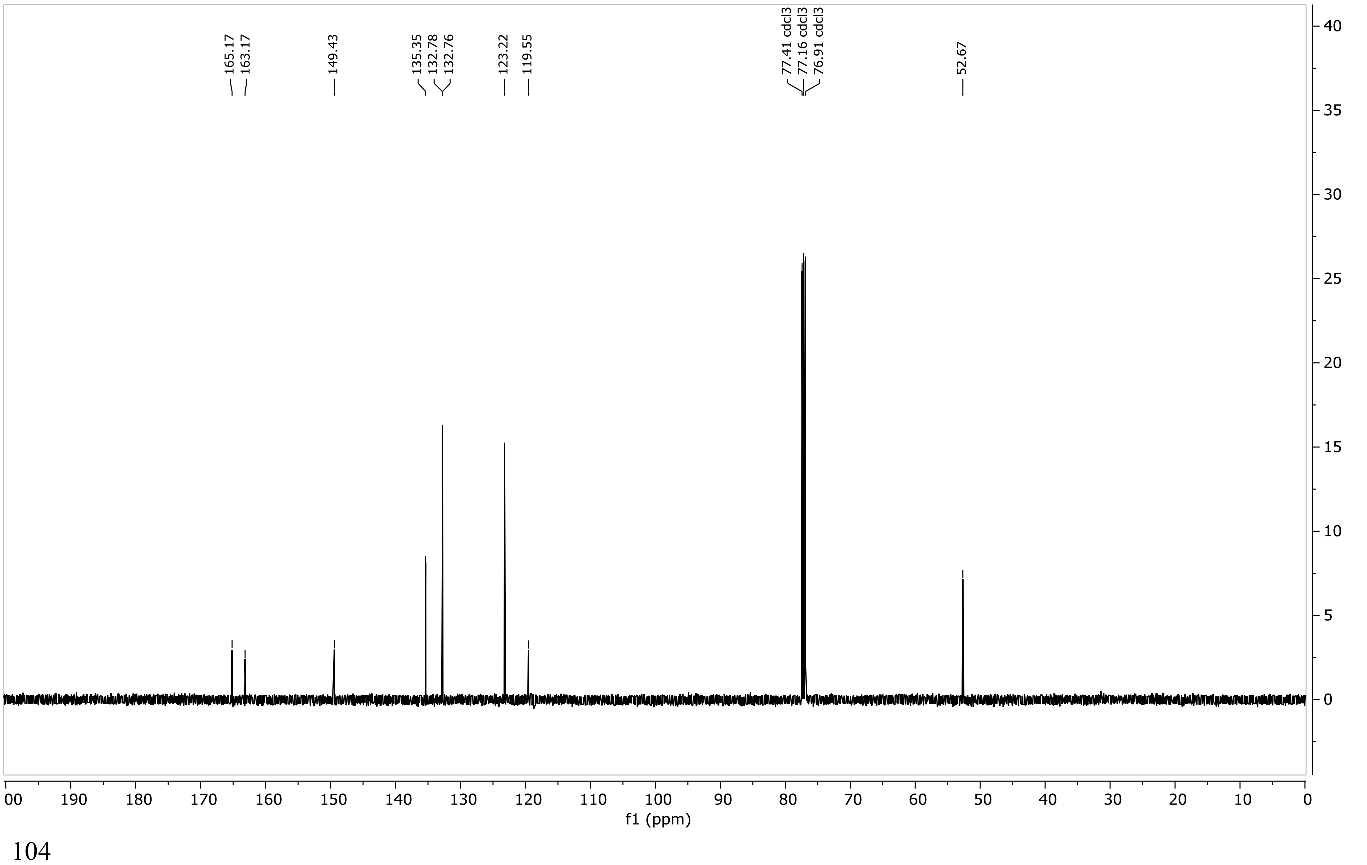

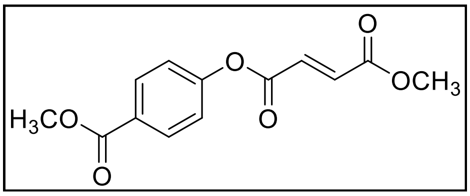

E-15

^{1}H NMR (600 MHz, $CDCl_3$)

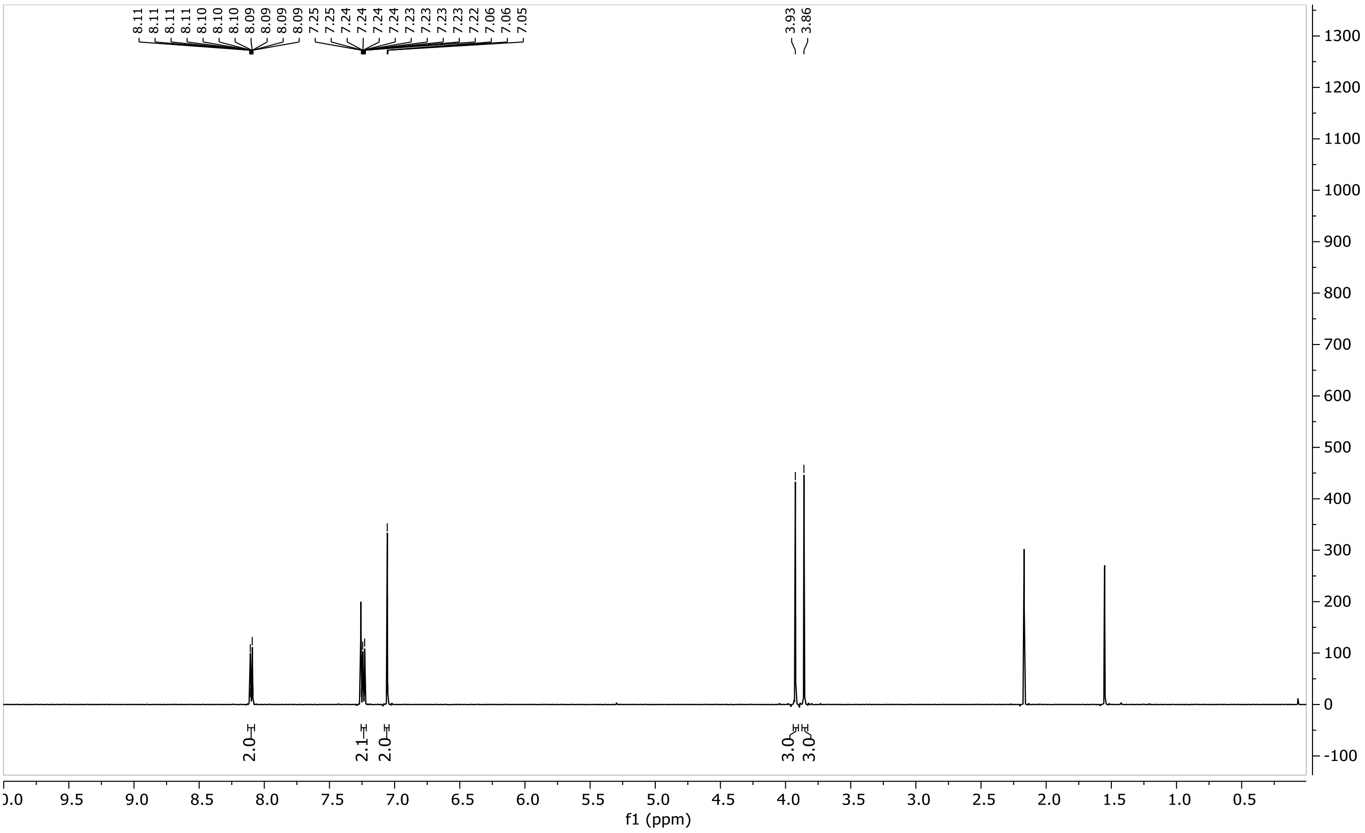

^{13}C NMR (151 MHz, $CDCl_3$)

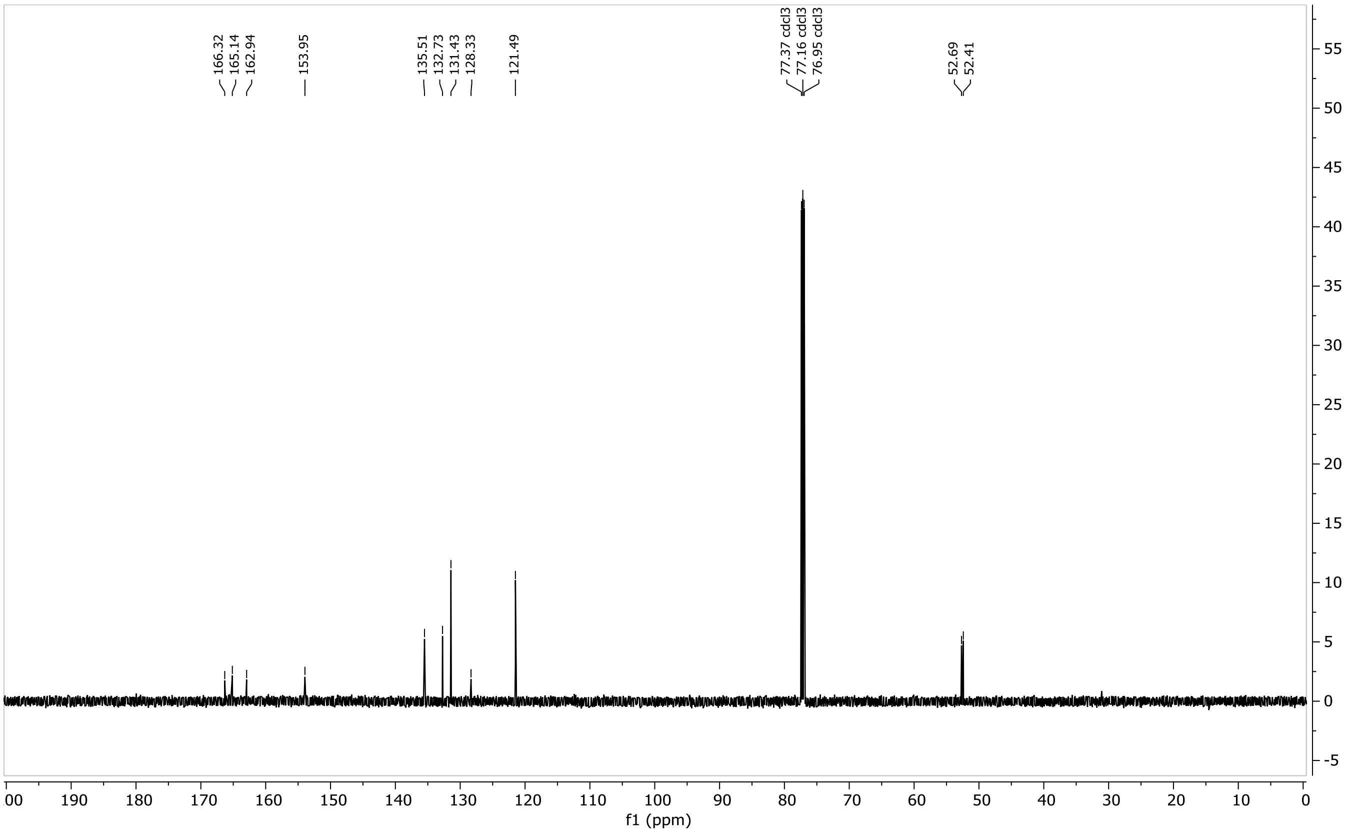

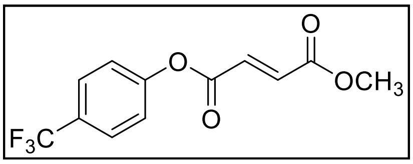

E-16

^{1}H NMR (500 MHz, $CDCl_3$)

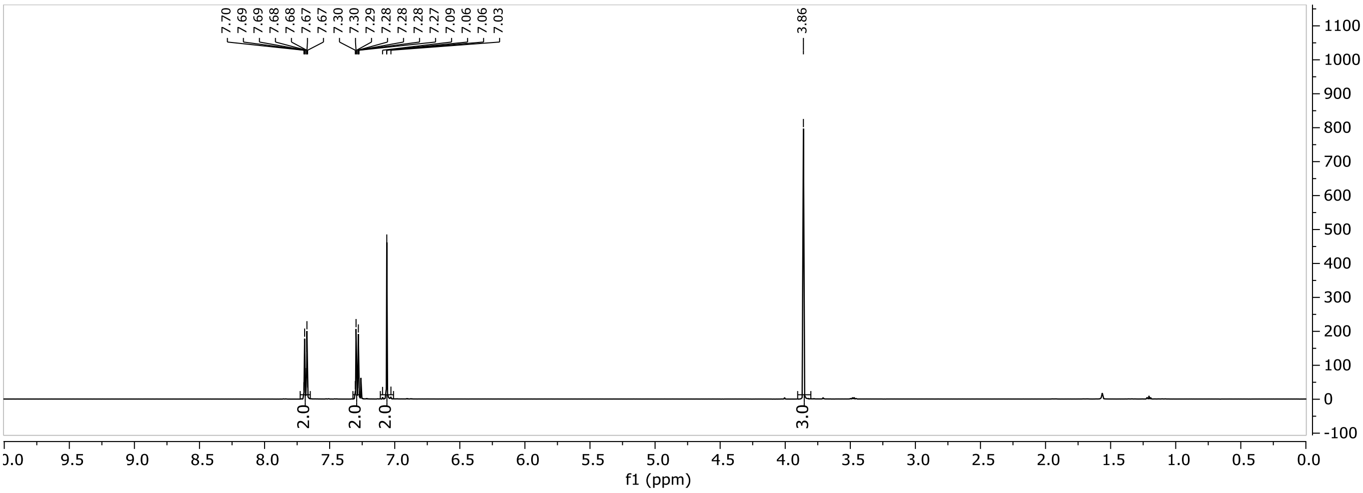

^{13}C-{^{19}F} NMR (126 MHz, $CDCl_3$)

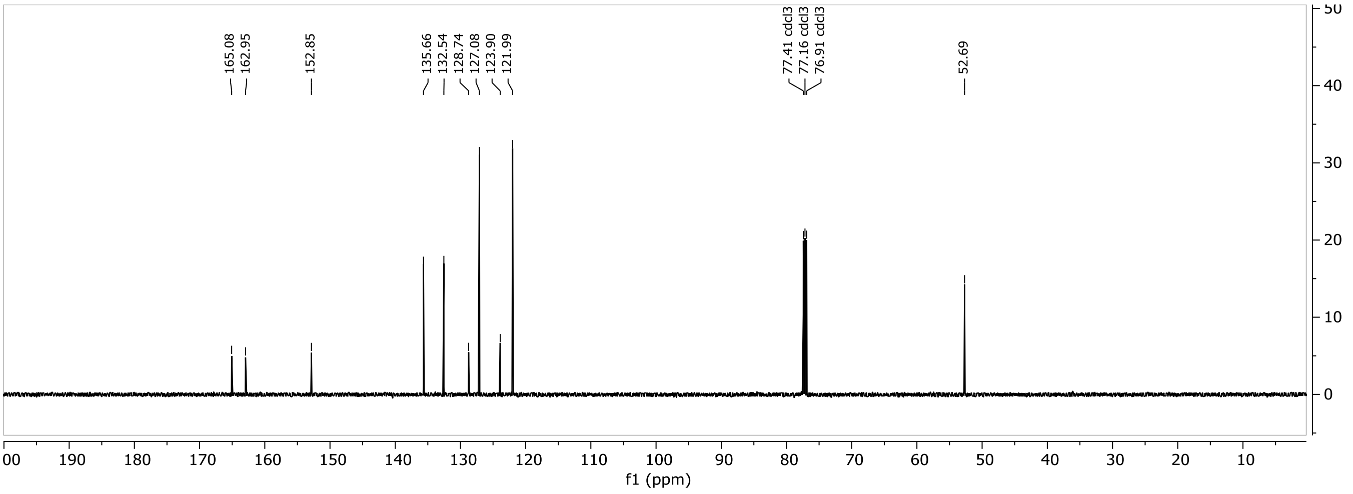

^{19}F NMR (470 MHz, $CDCl_3$)

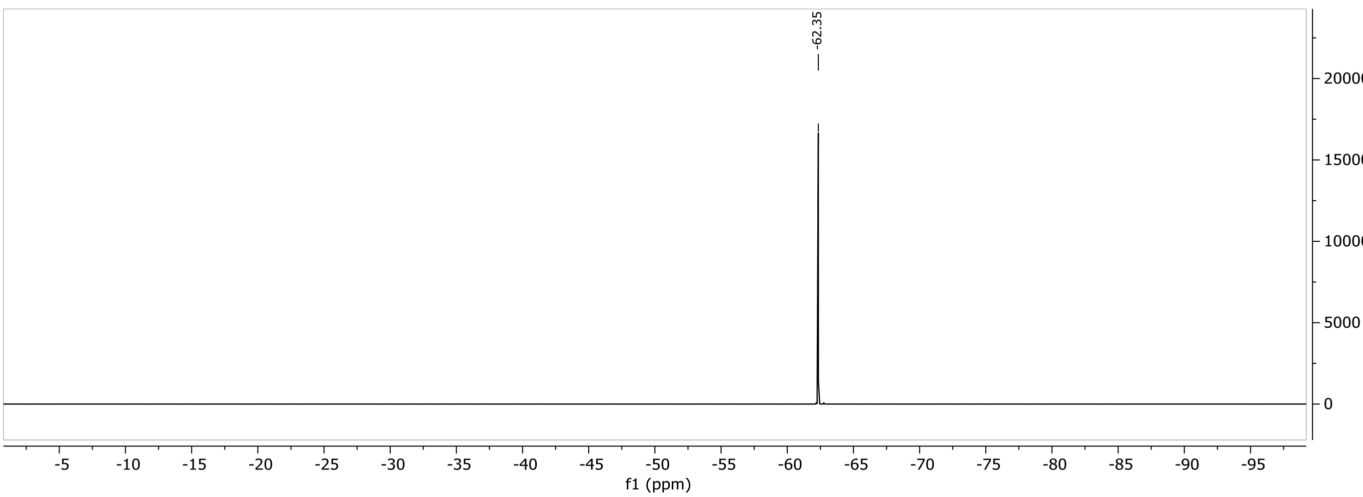

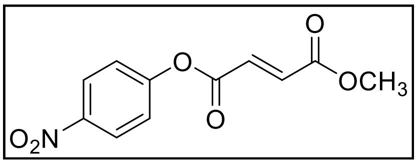

E-17

^{1}H NMR (600 MHz, $CDCl_3$)

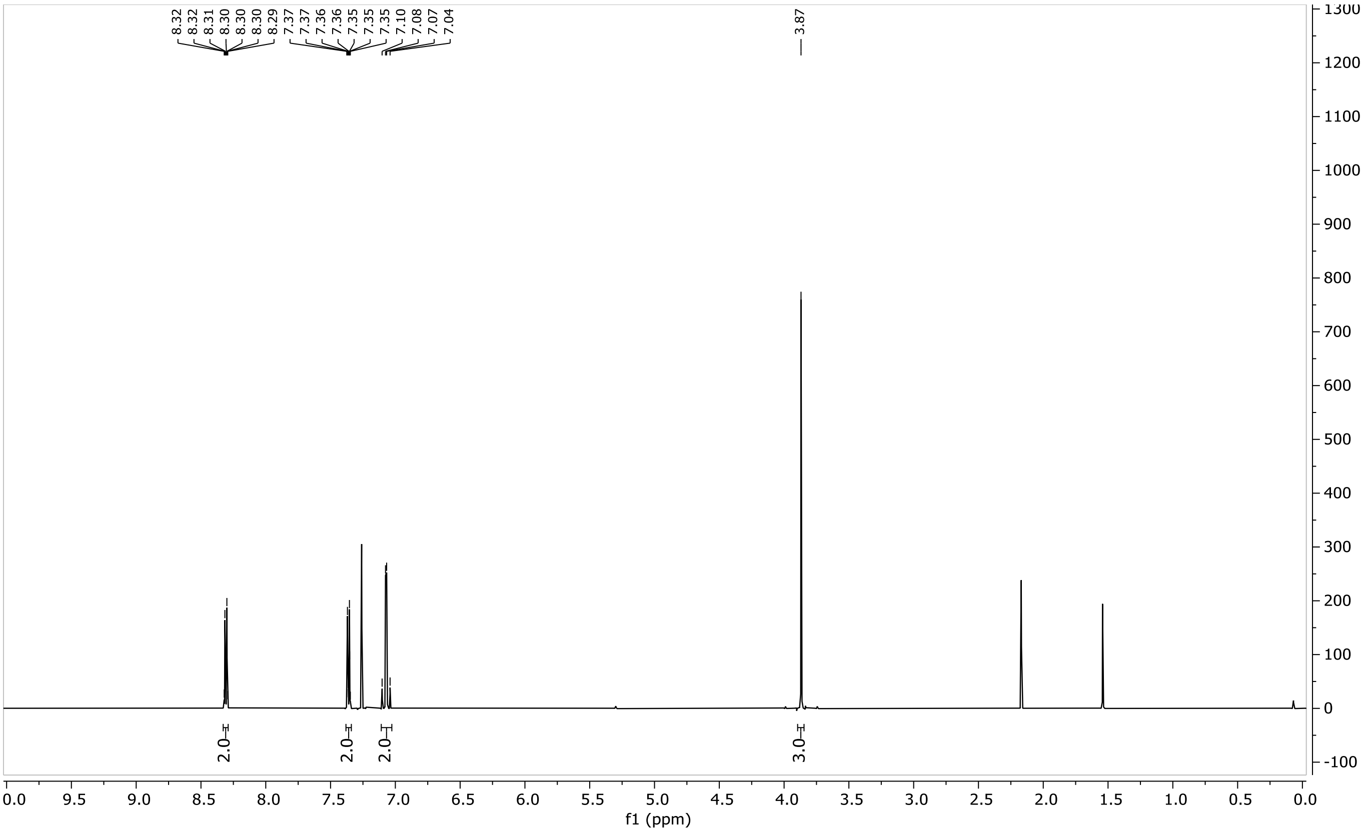

^{13}C NMR (151 MHz, $CDCl_3$)

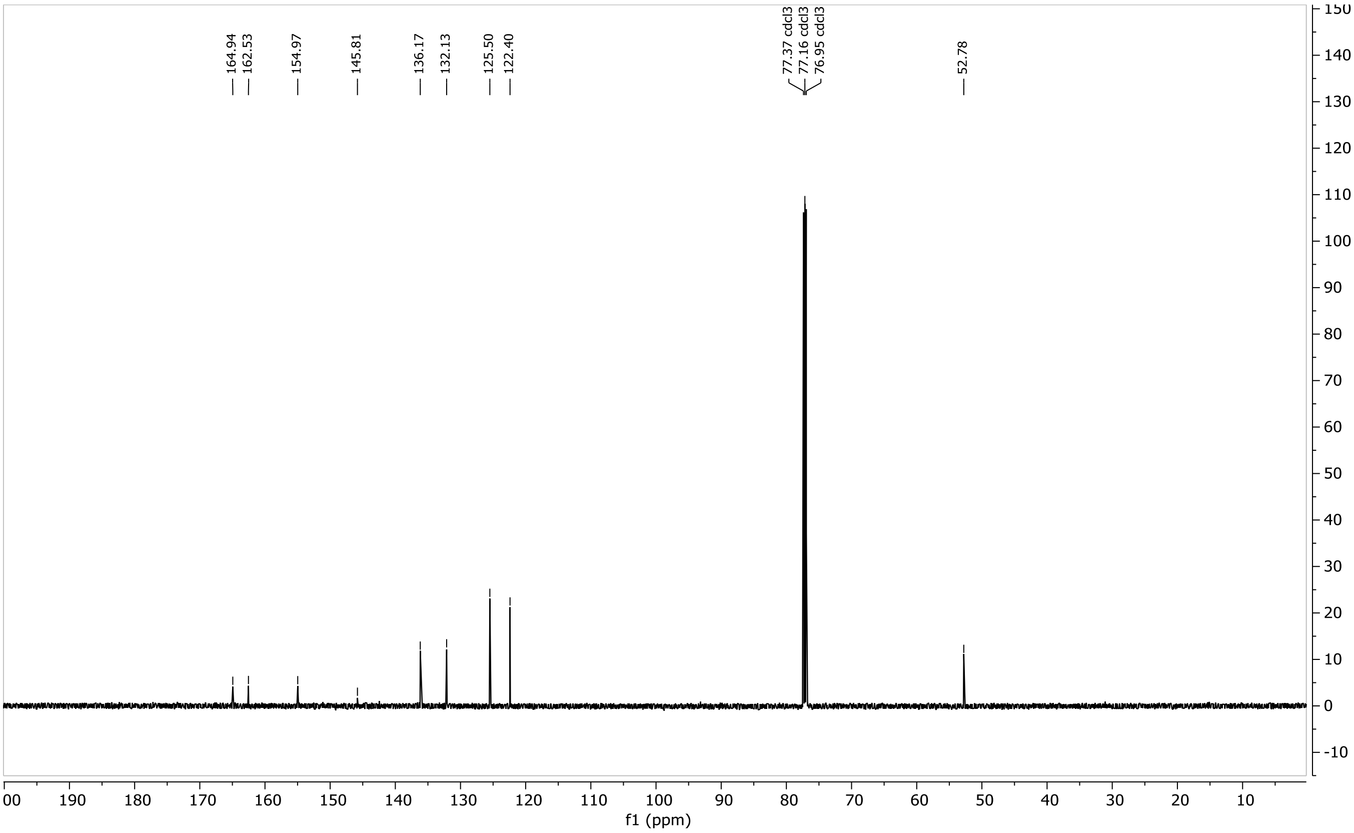

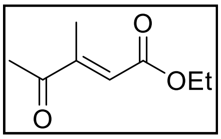

E-18

^{1}H NMR (500 MHz, $CDCl_3$)

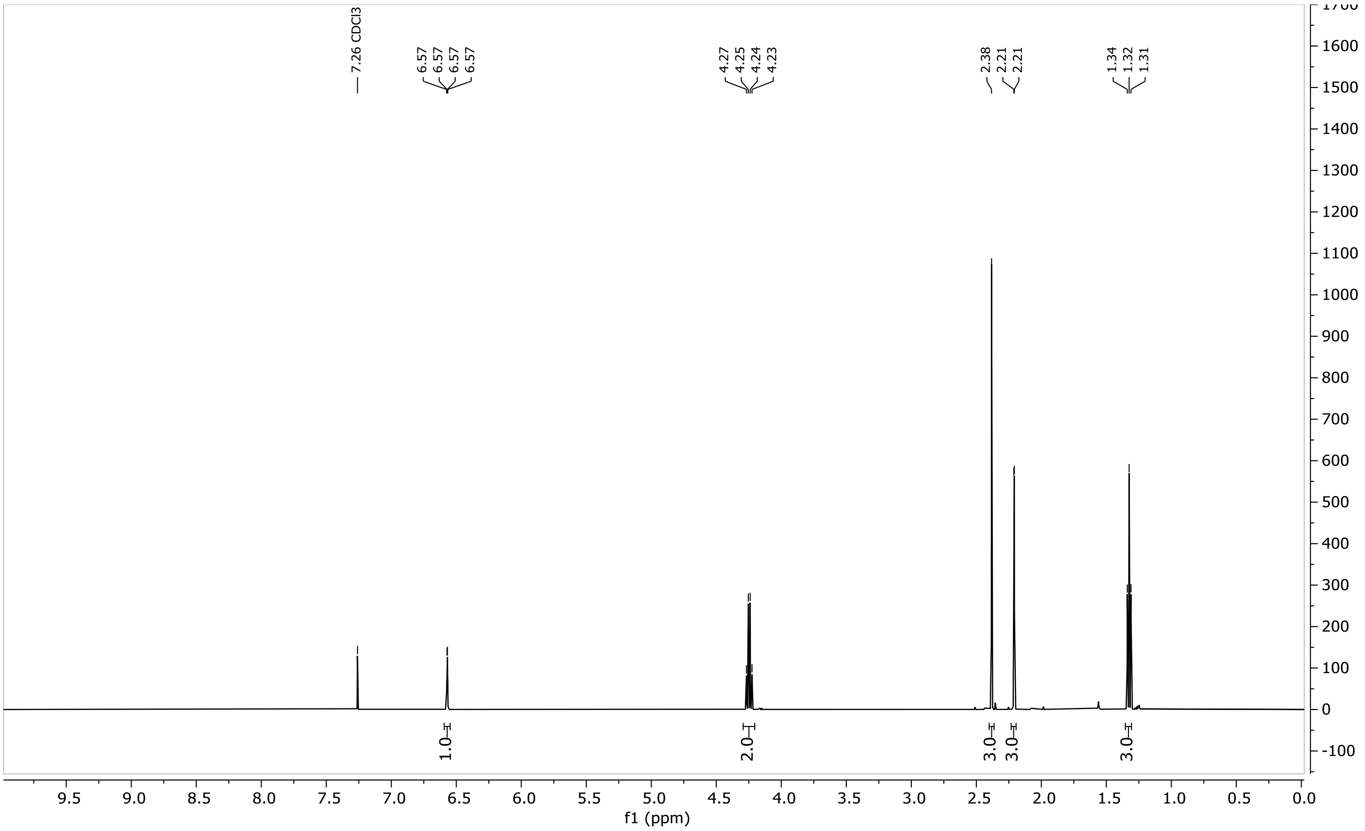

^{13}C NMR (126 MHz, $CDCl_3$)

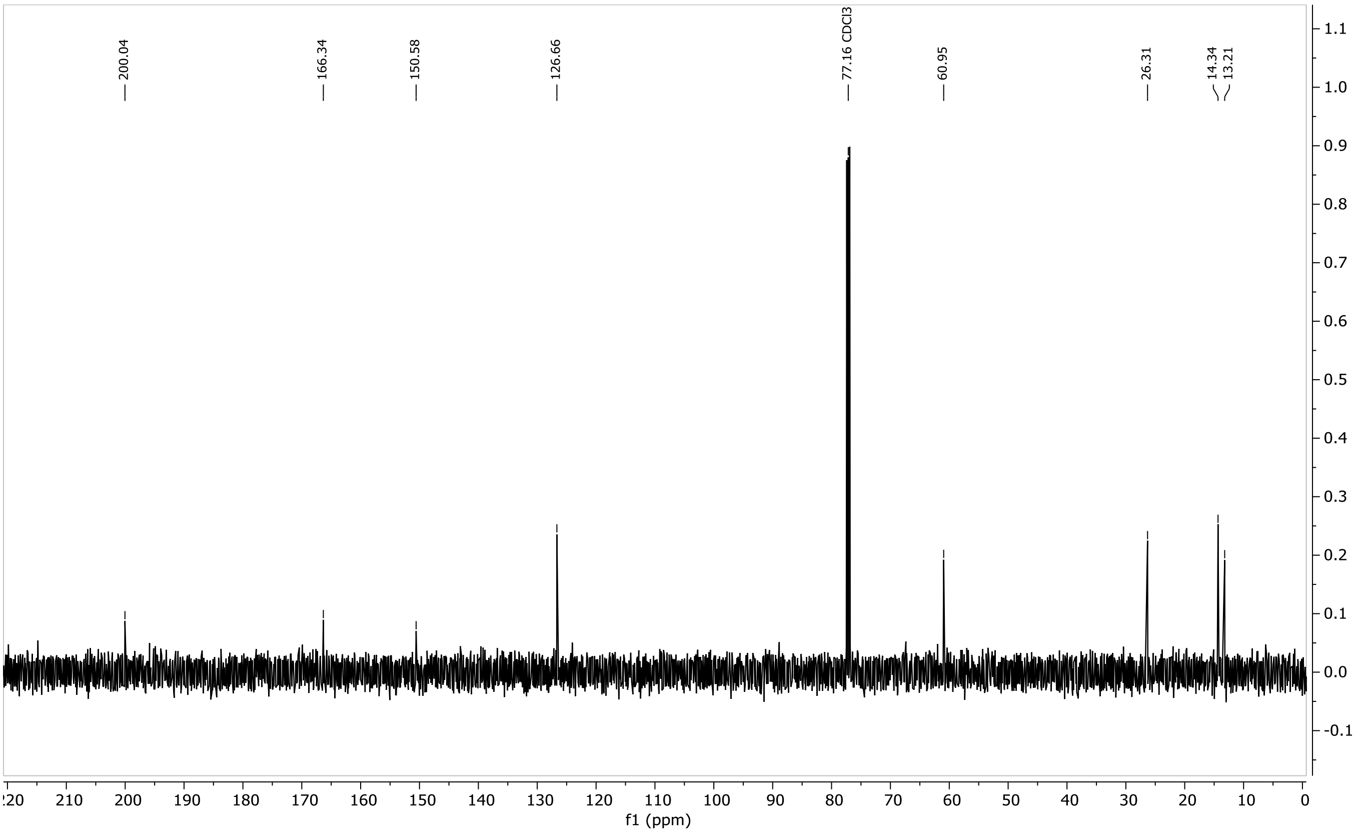

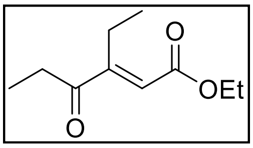

E-19

^{1}H NMR (500 MHz, $CDCl_3$)

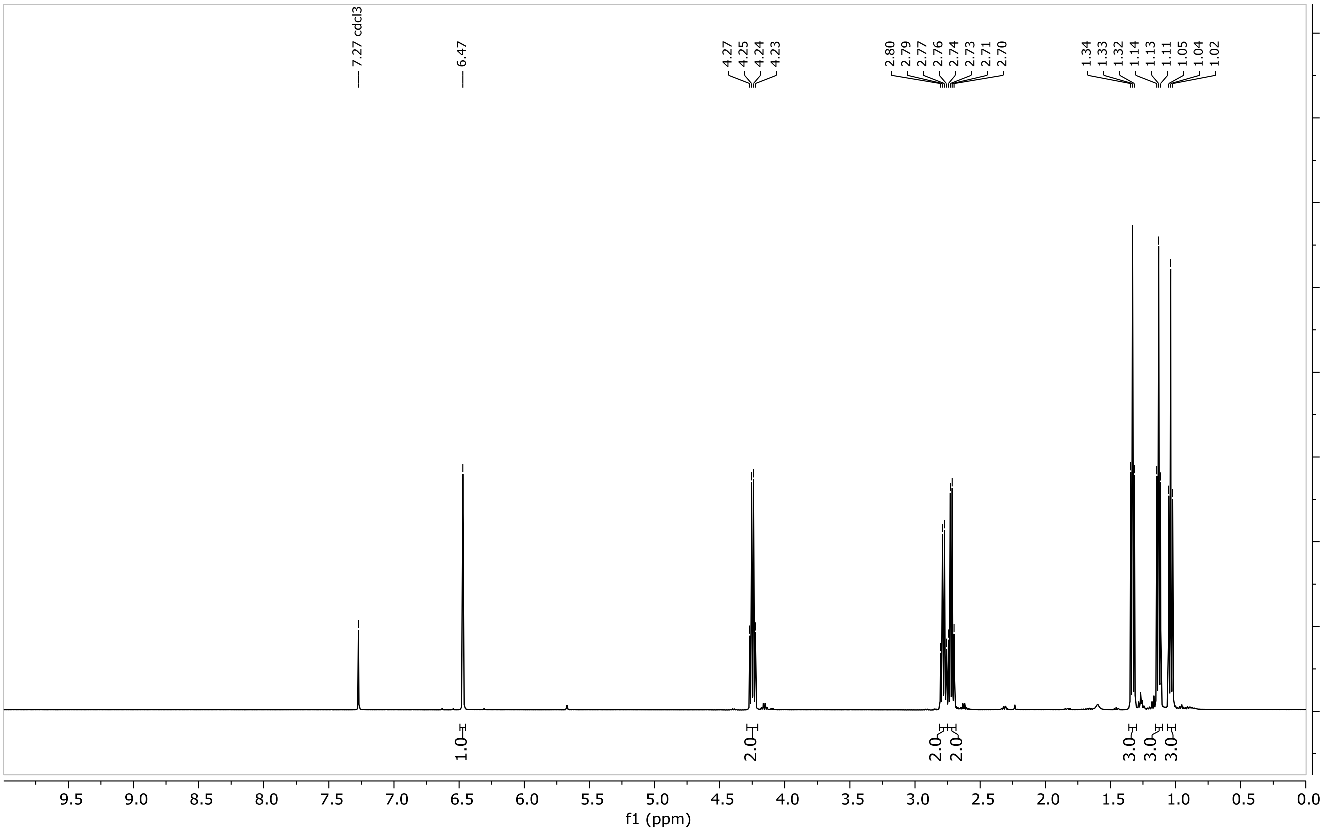

^{13}C NMR (126 MHz, $CDCl_3$)

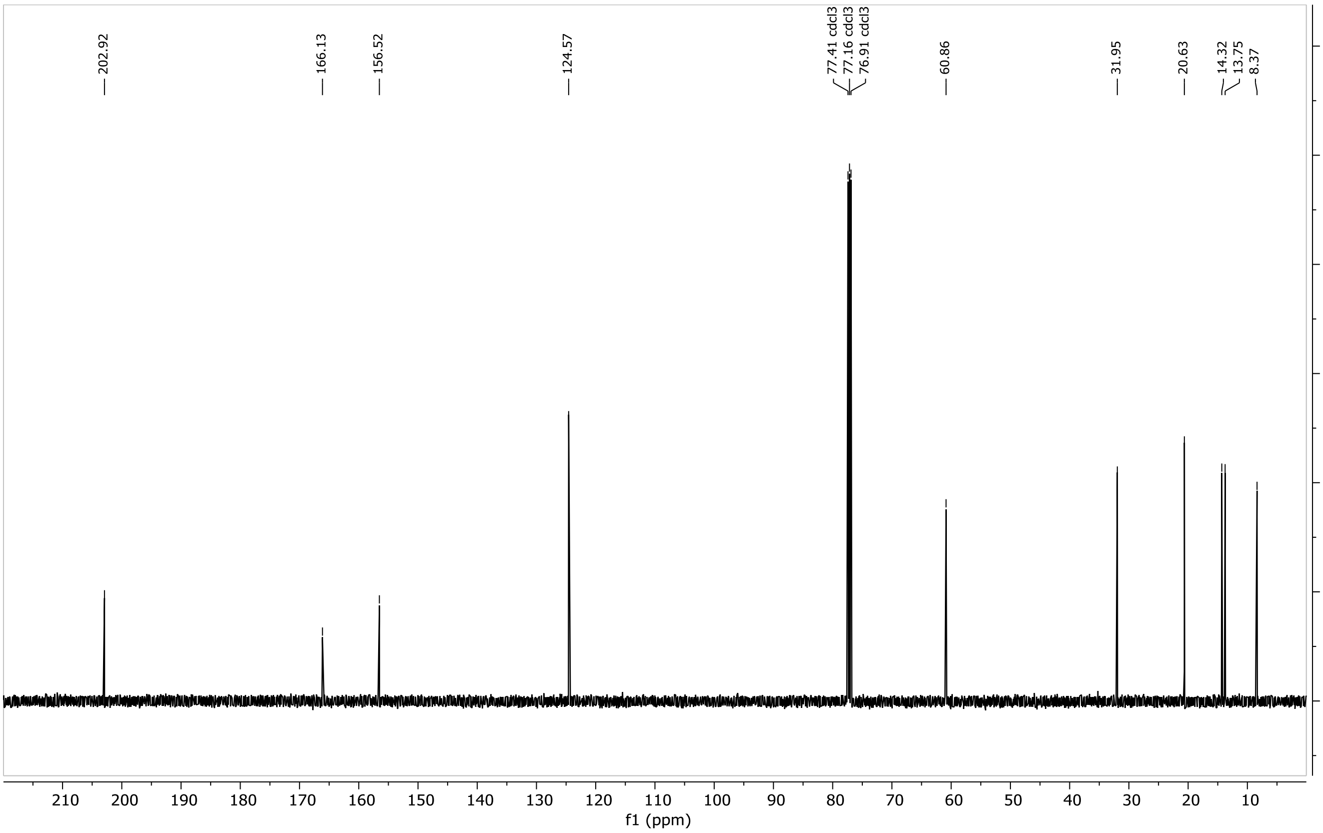

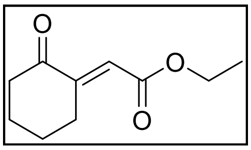

E-20

^{1}H NMR (600 MHz, $CDCl_3$)

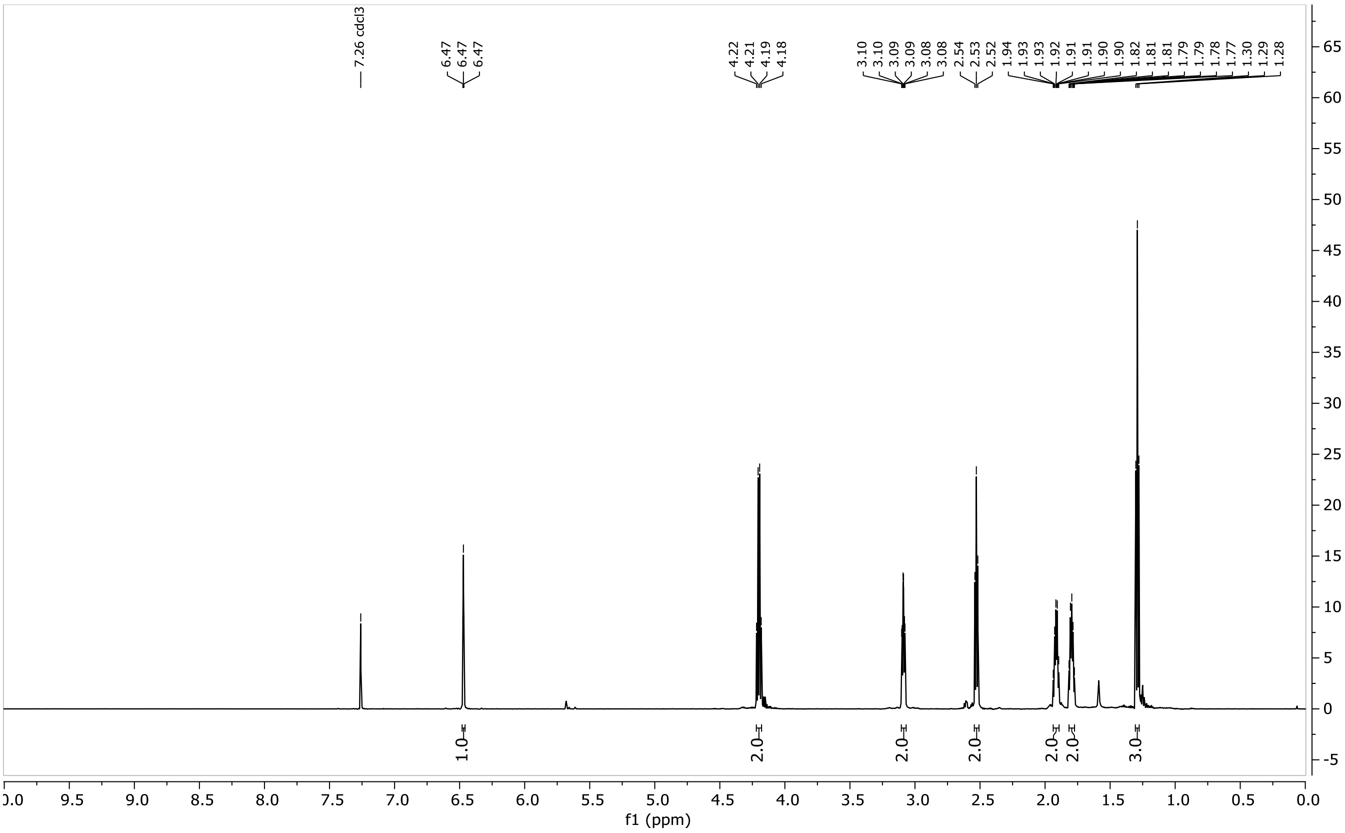

^{13}C NMR (151 MHz, $CDCl_3$)

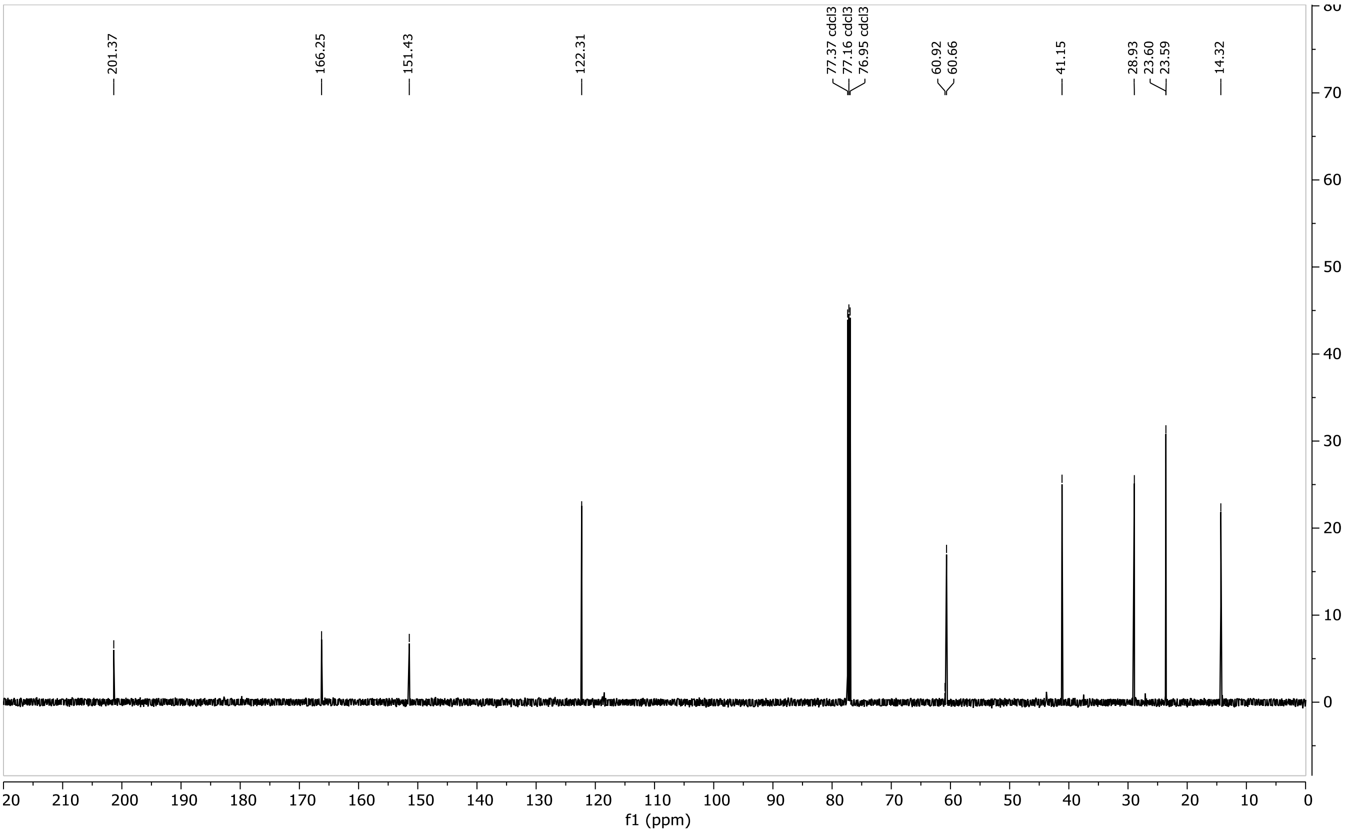

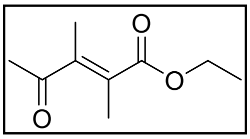

E-21

^{1}H NMR (400 MHz, $CDCl_3$)

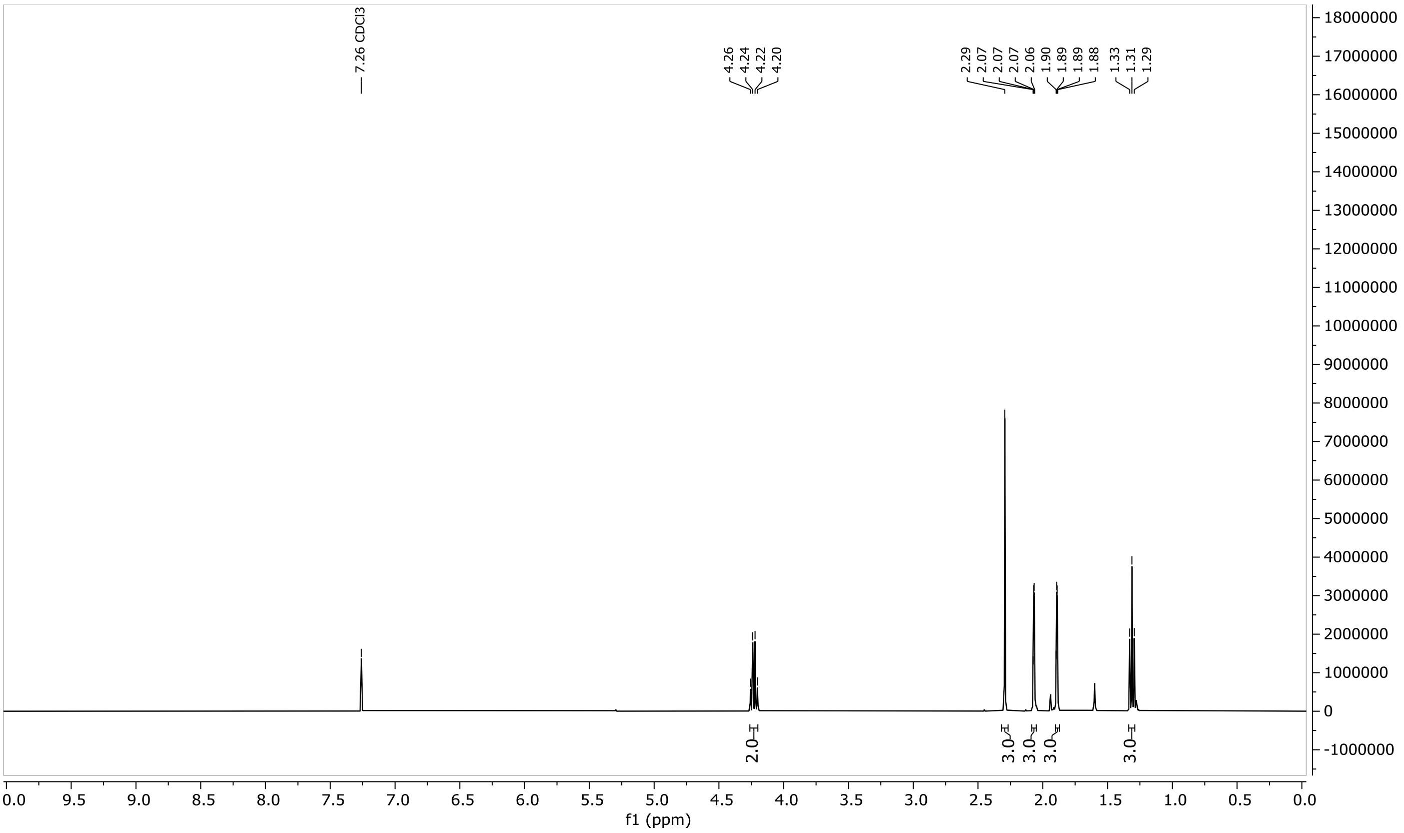

^{13}C NMR (100 MHz, $CDCl_3$)

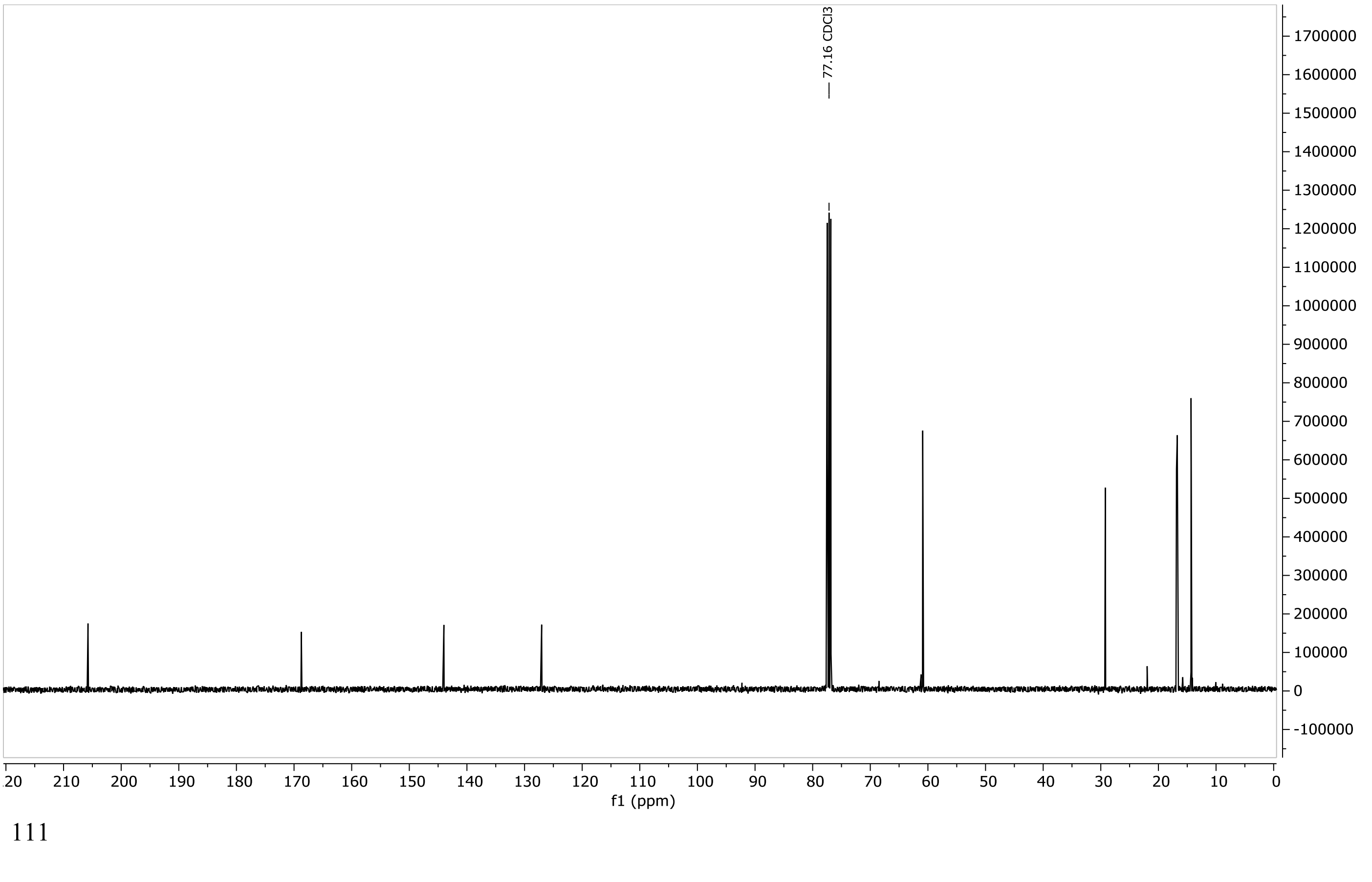

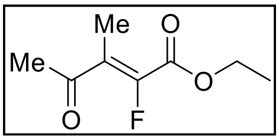

Z-22

^{1}H NMR (600 MHz, $CDCl_3$)

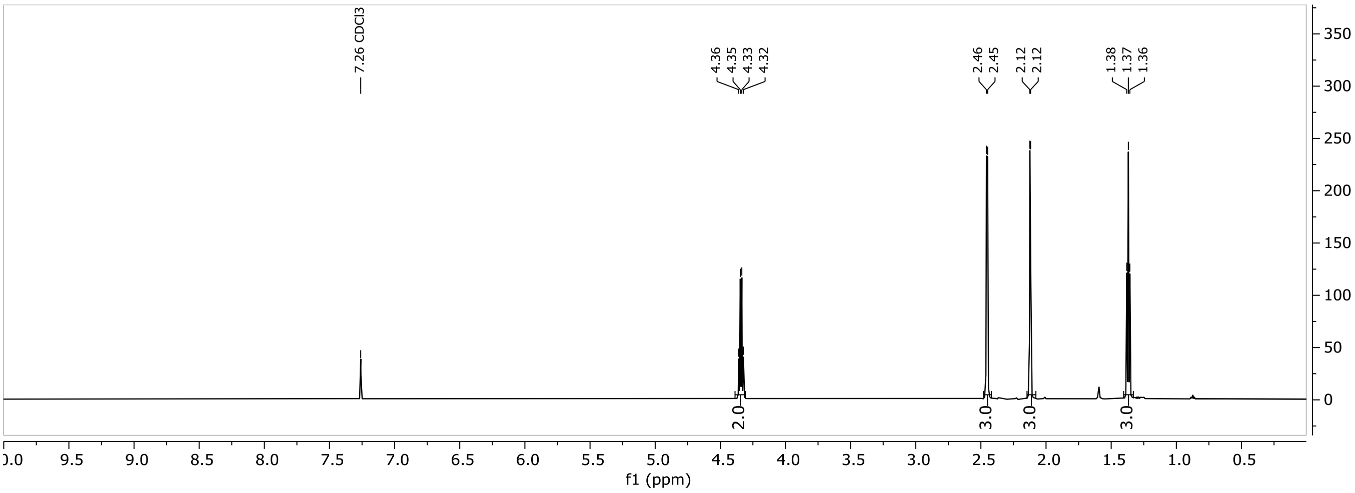

^{13}C-{^{19}F} NMR (151 MHz, $CDCl_3$)

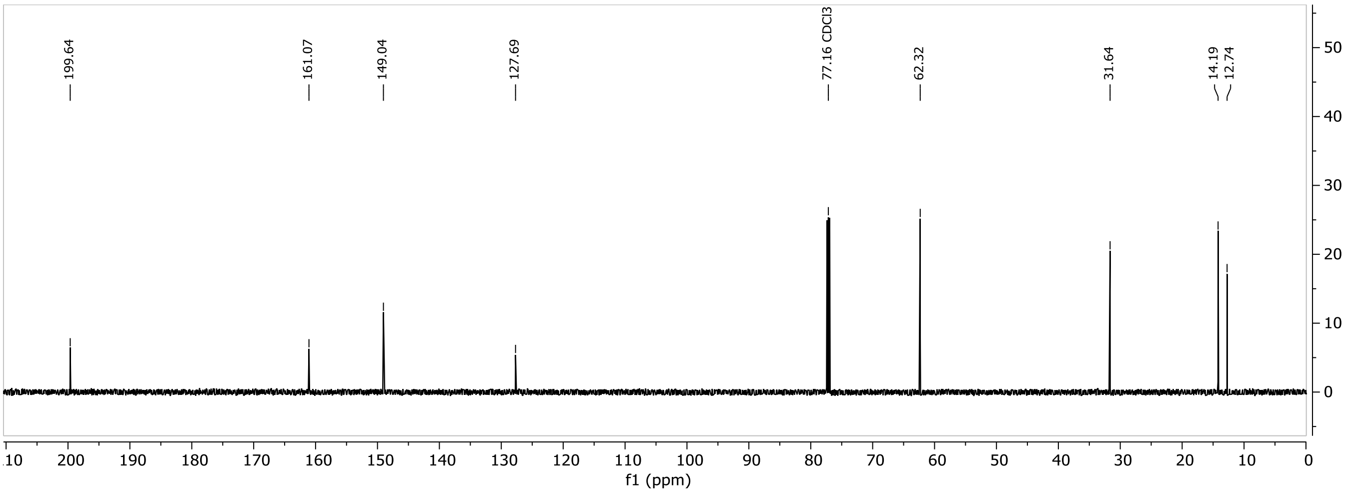

^{19}F NMR (564 MHz, $CDCl_3$)

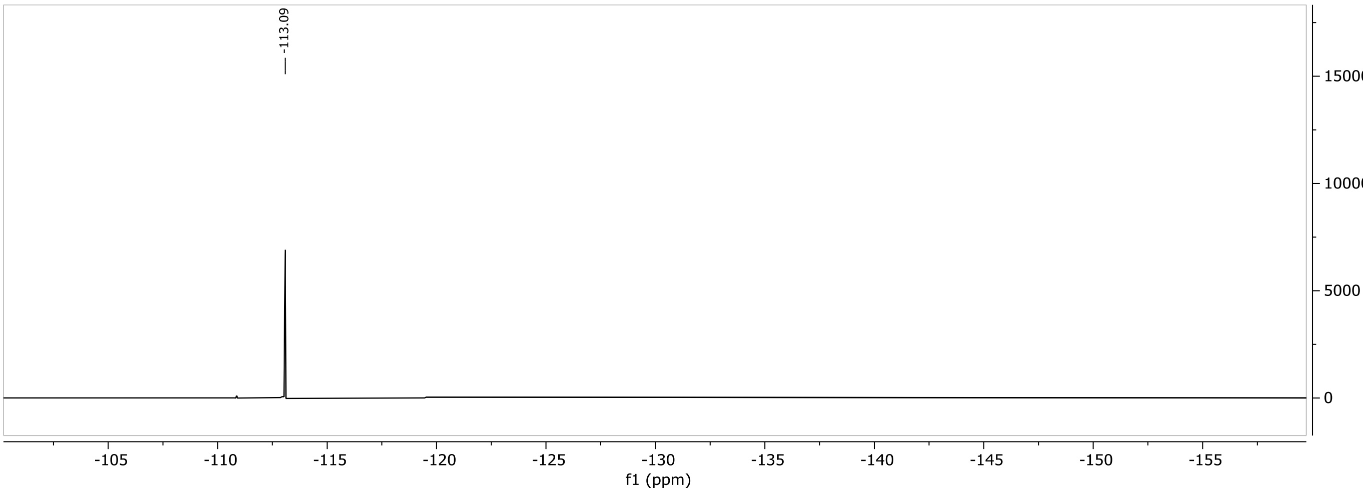

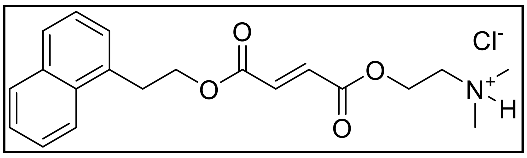

E-23

^{1}H NMR (600 MHz, $CDCl_3$)

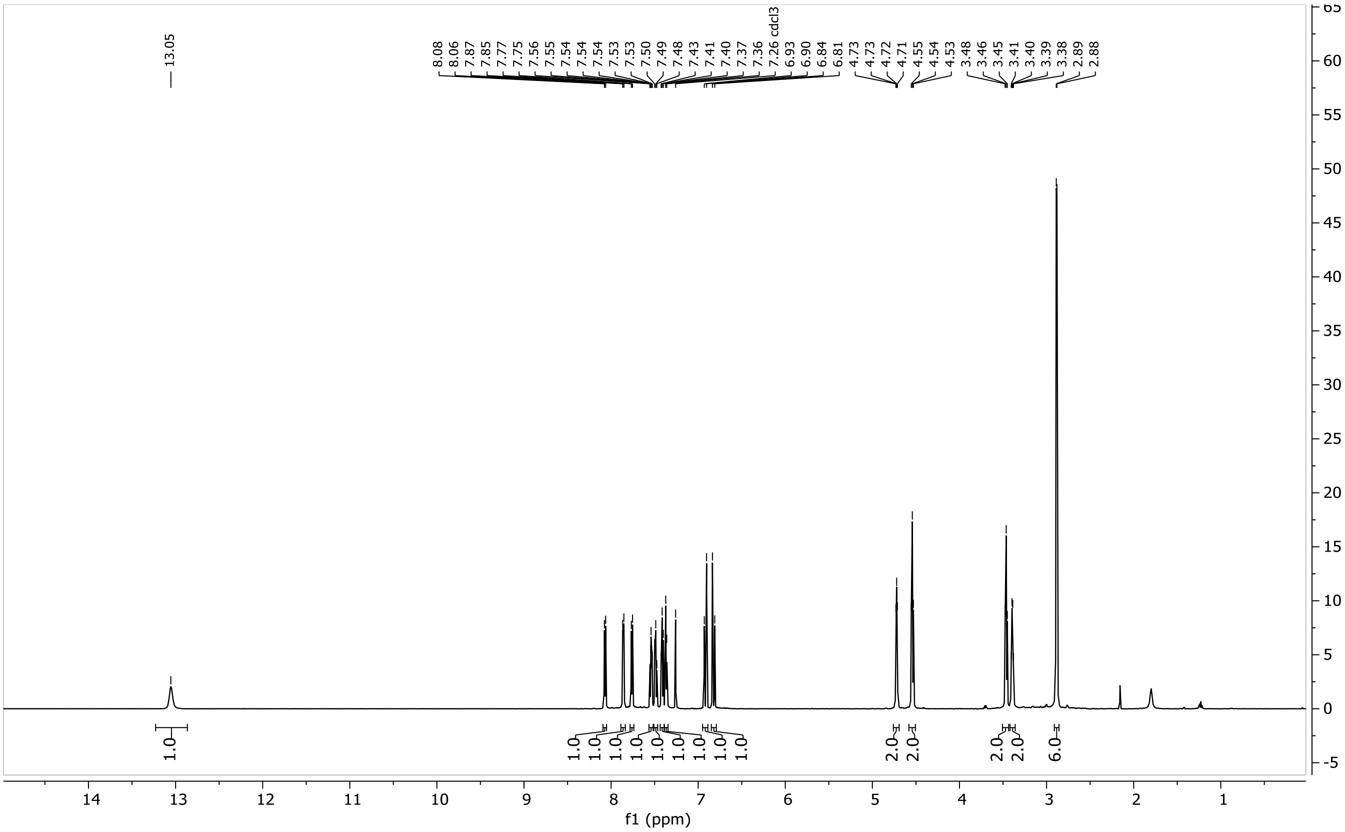

^{13}C NMR (151 MHz, $CDCl_3$)

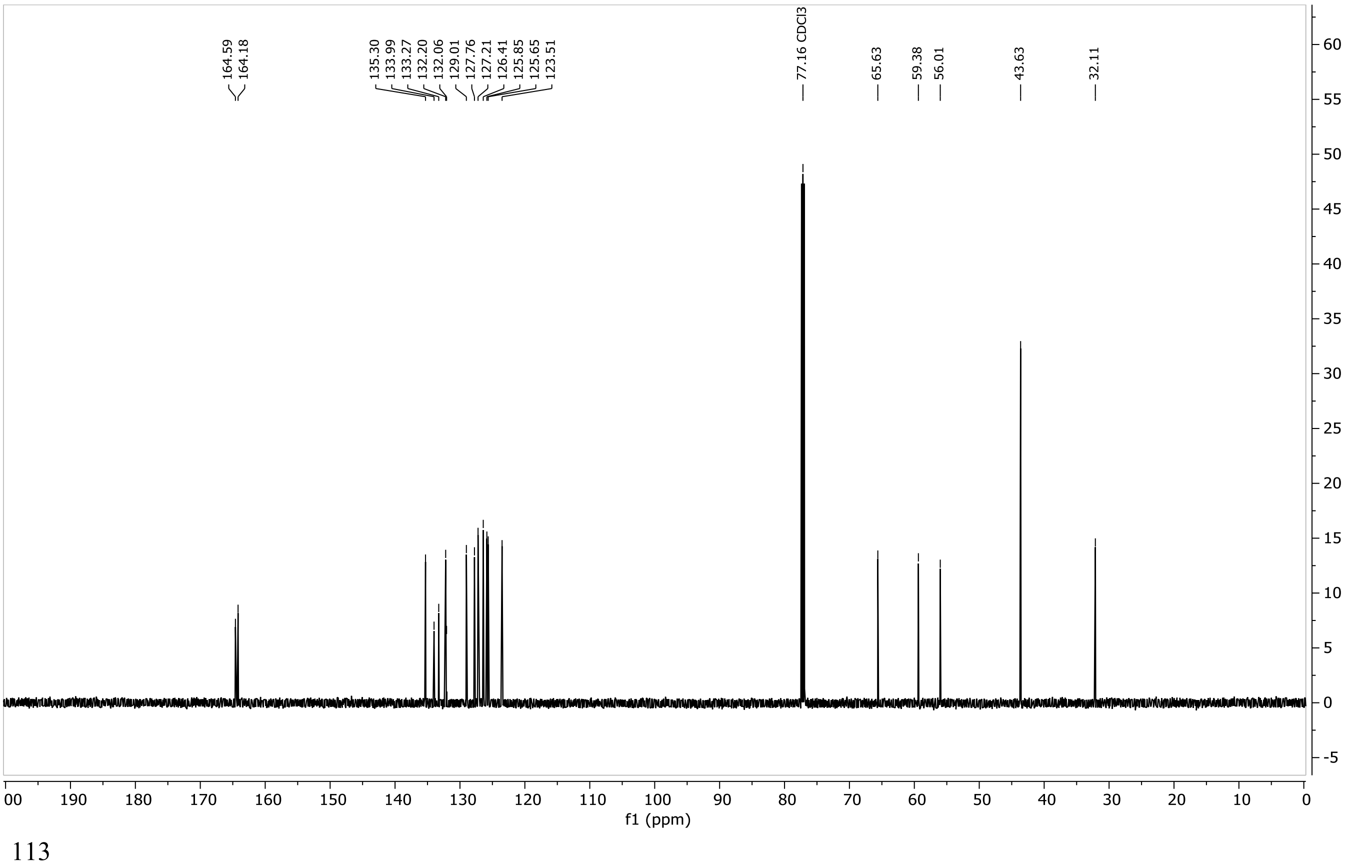

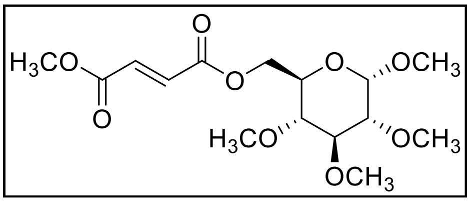

E-24

^{1}H NMR (600 MHz, $CDCl_3$)

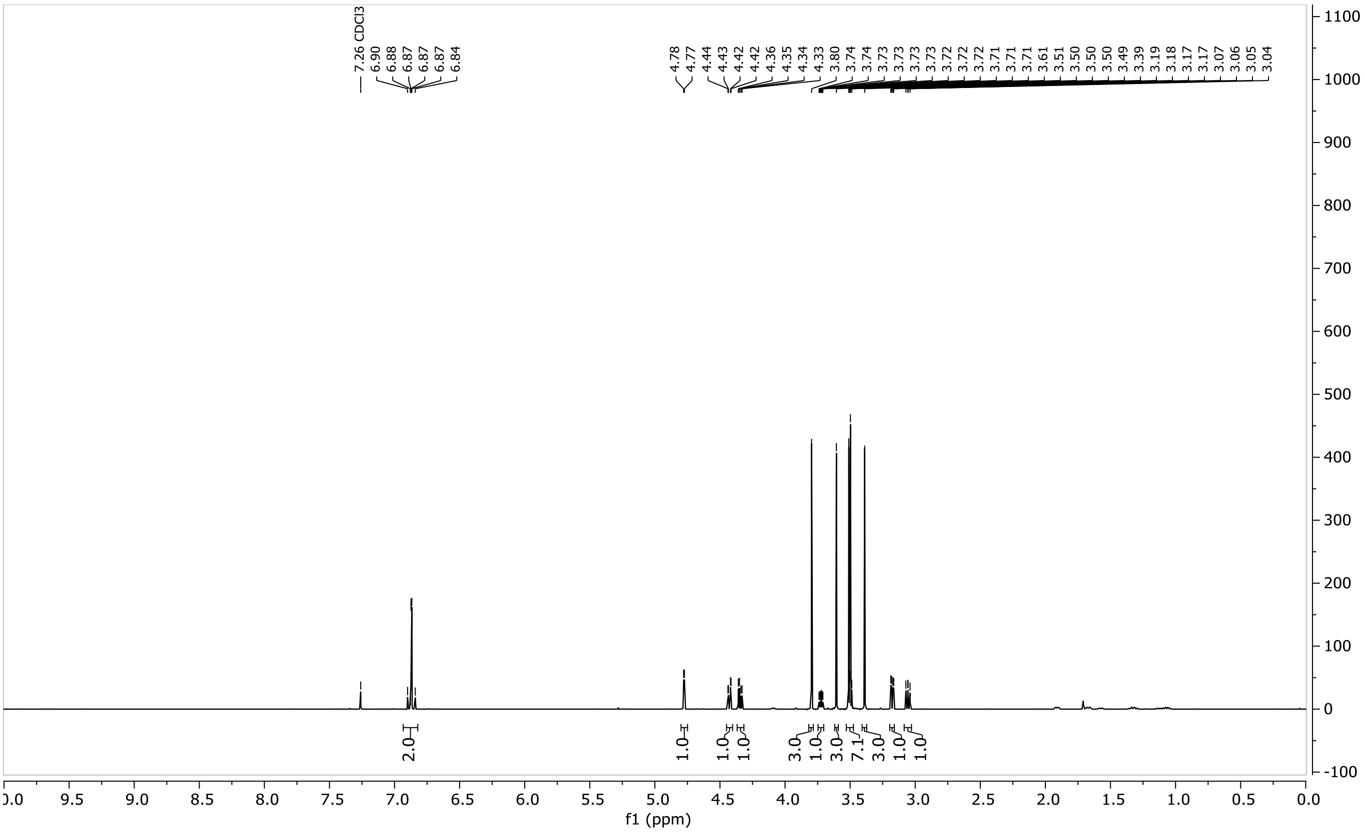

^{13}C NMR (151 MHz, $CDCl_3$)

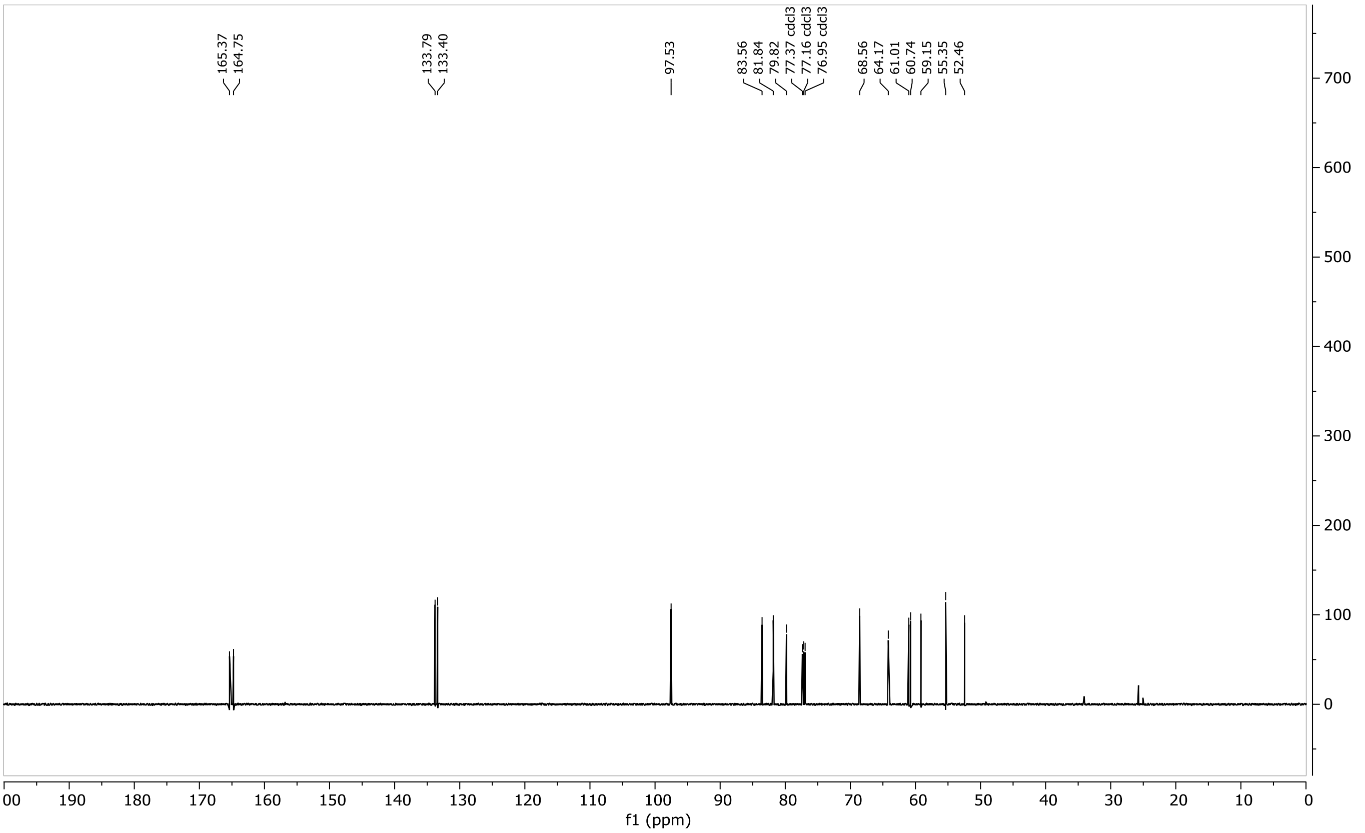

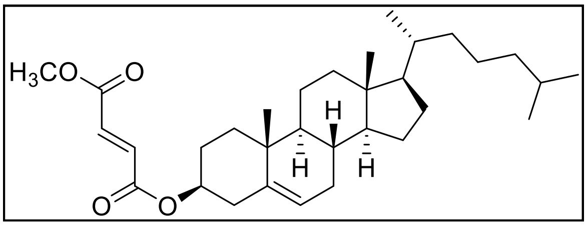

E-25

^{1}H NMR (500 MHz, $CDCl_3$)

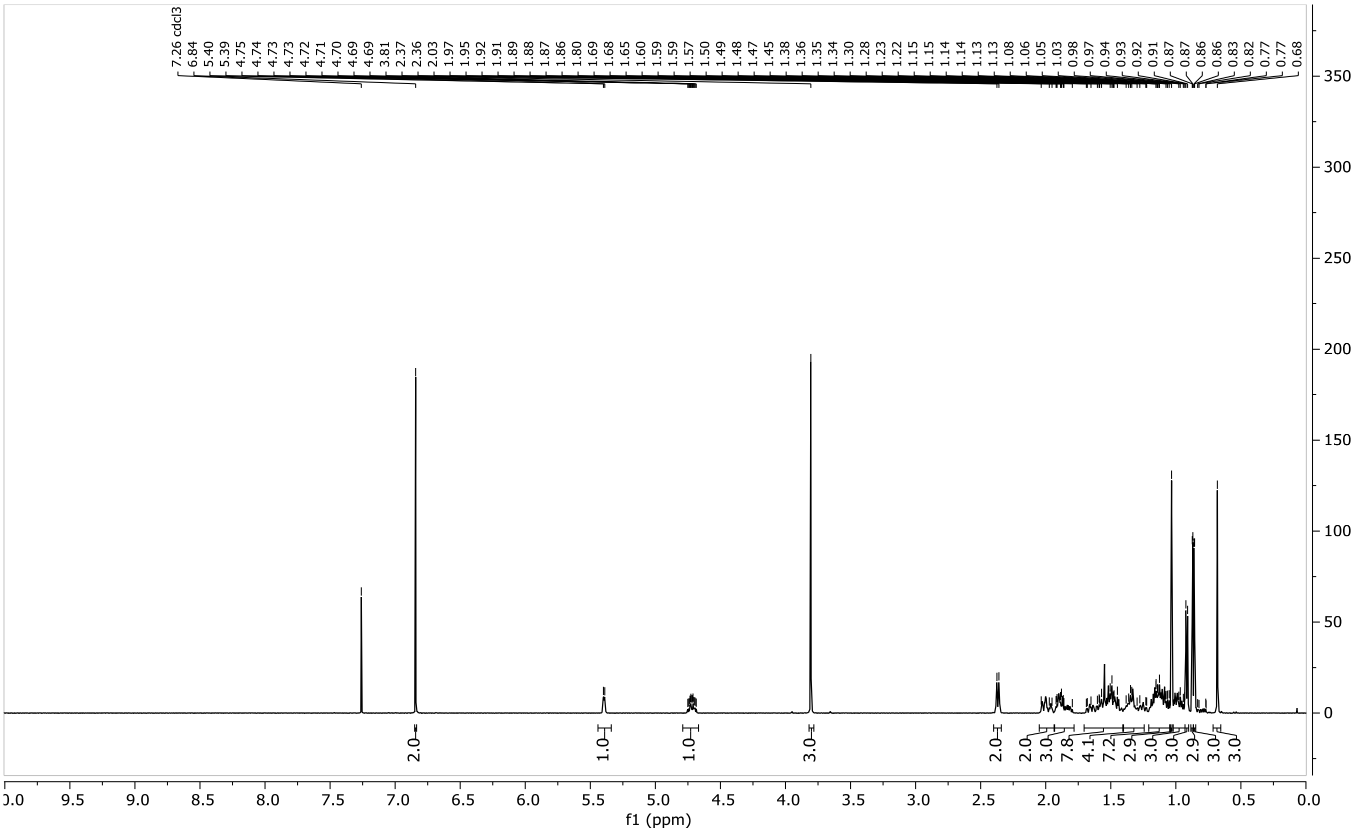

^{13}C NMR (126 MHz, $CDCl_3$)

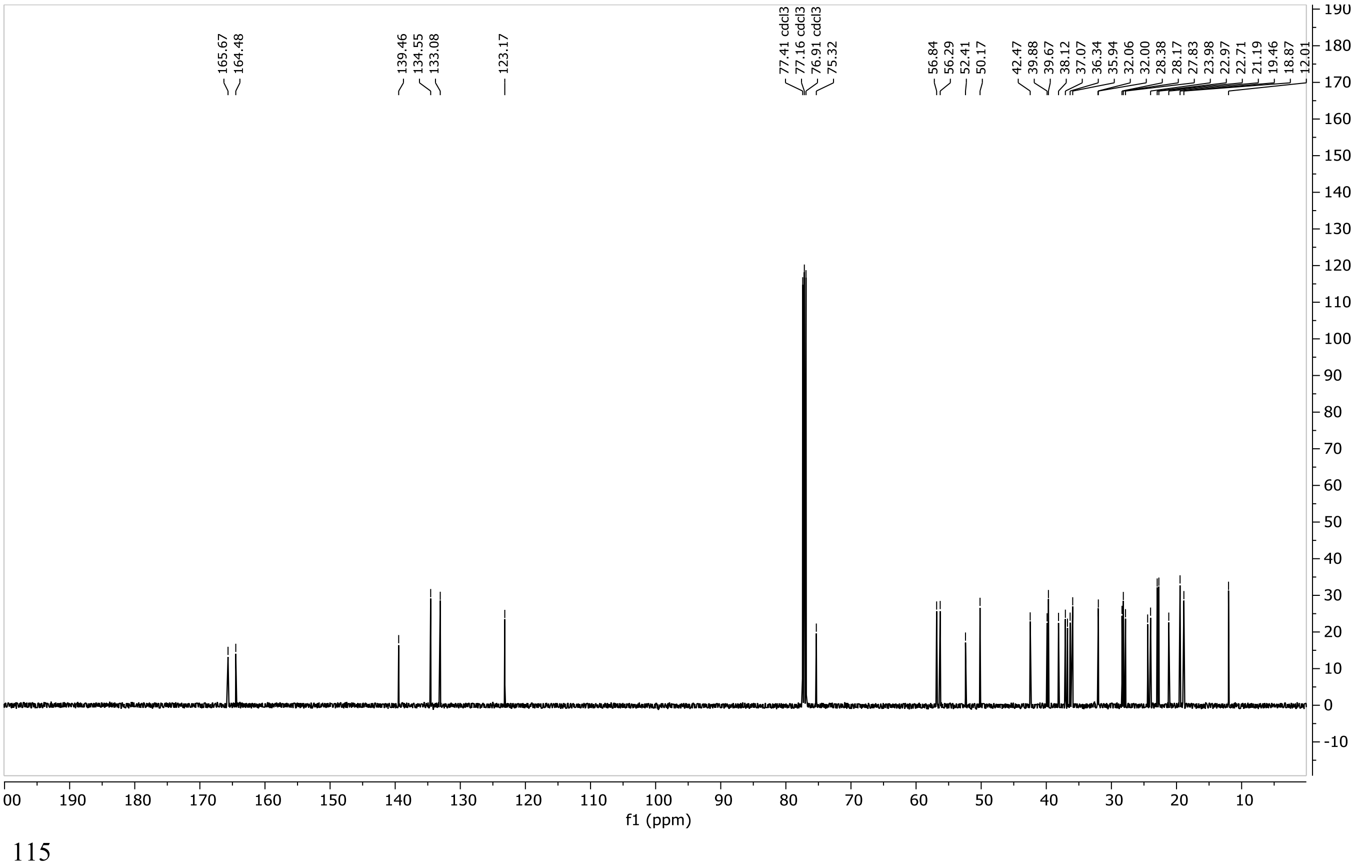

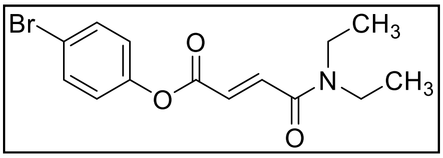

E-26

^{1}H NMR (500 MHz, $CDCl_3$)

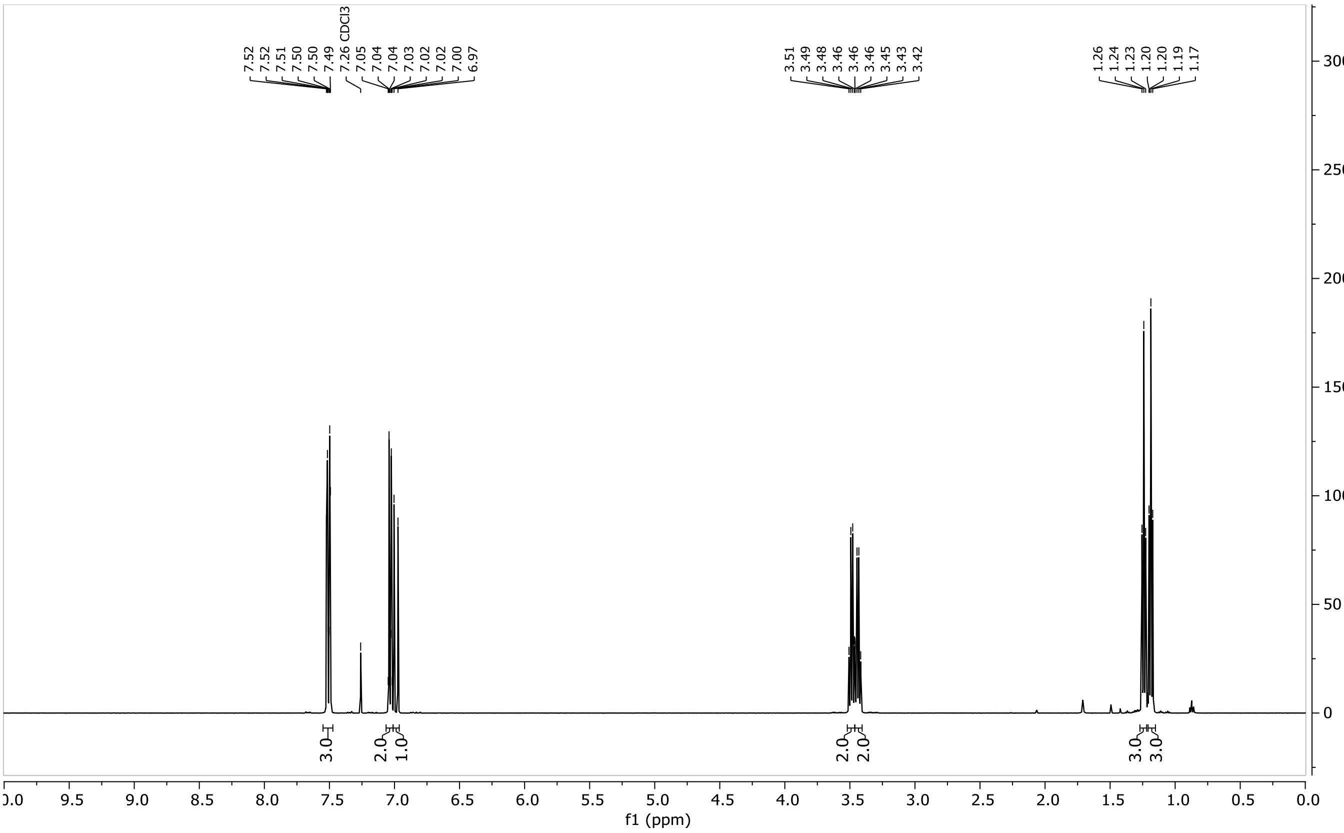

^{13}C NMR (126 MHz, $CDCl_3$)

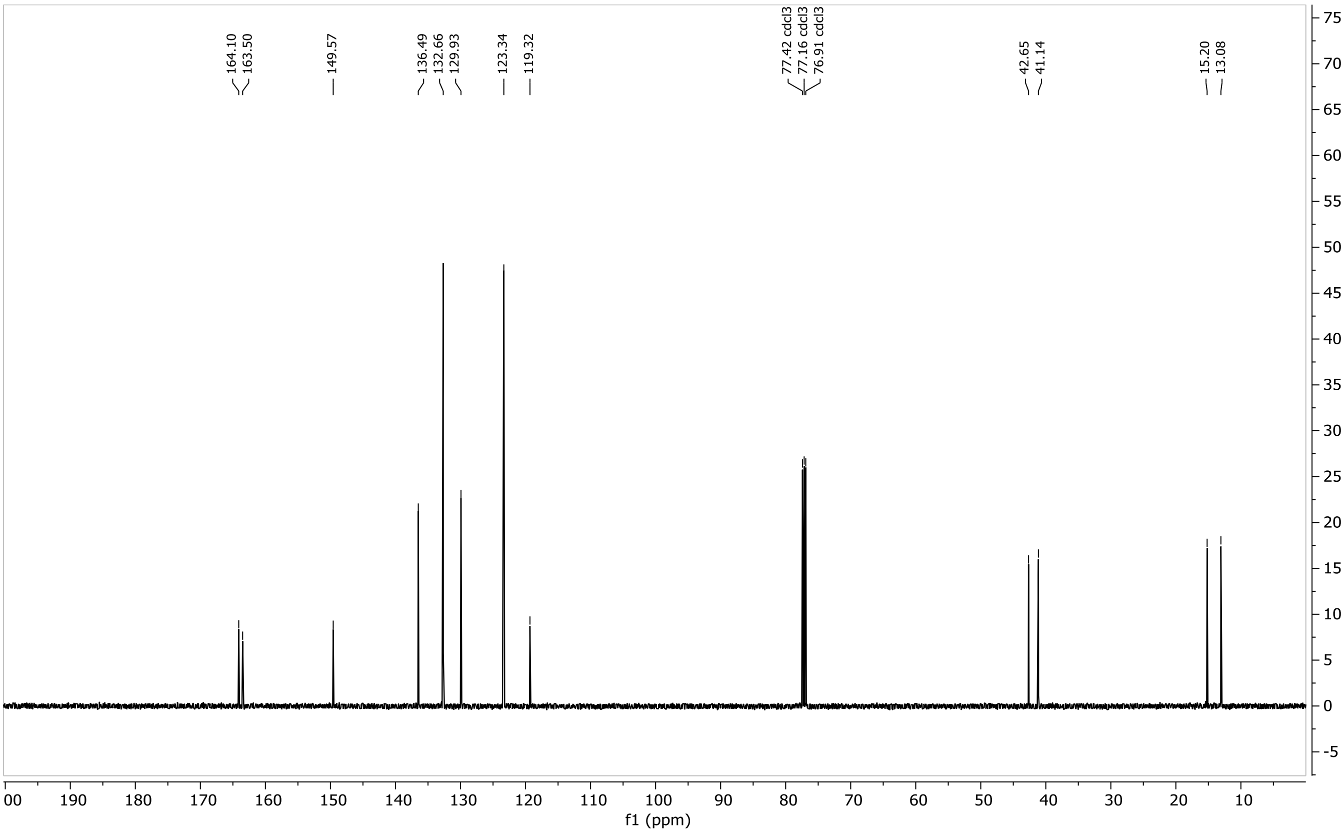

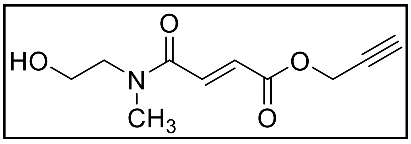

E-27

^{1}H NMR (500 MHz, $CDCl_3$)

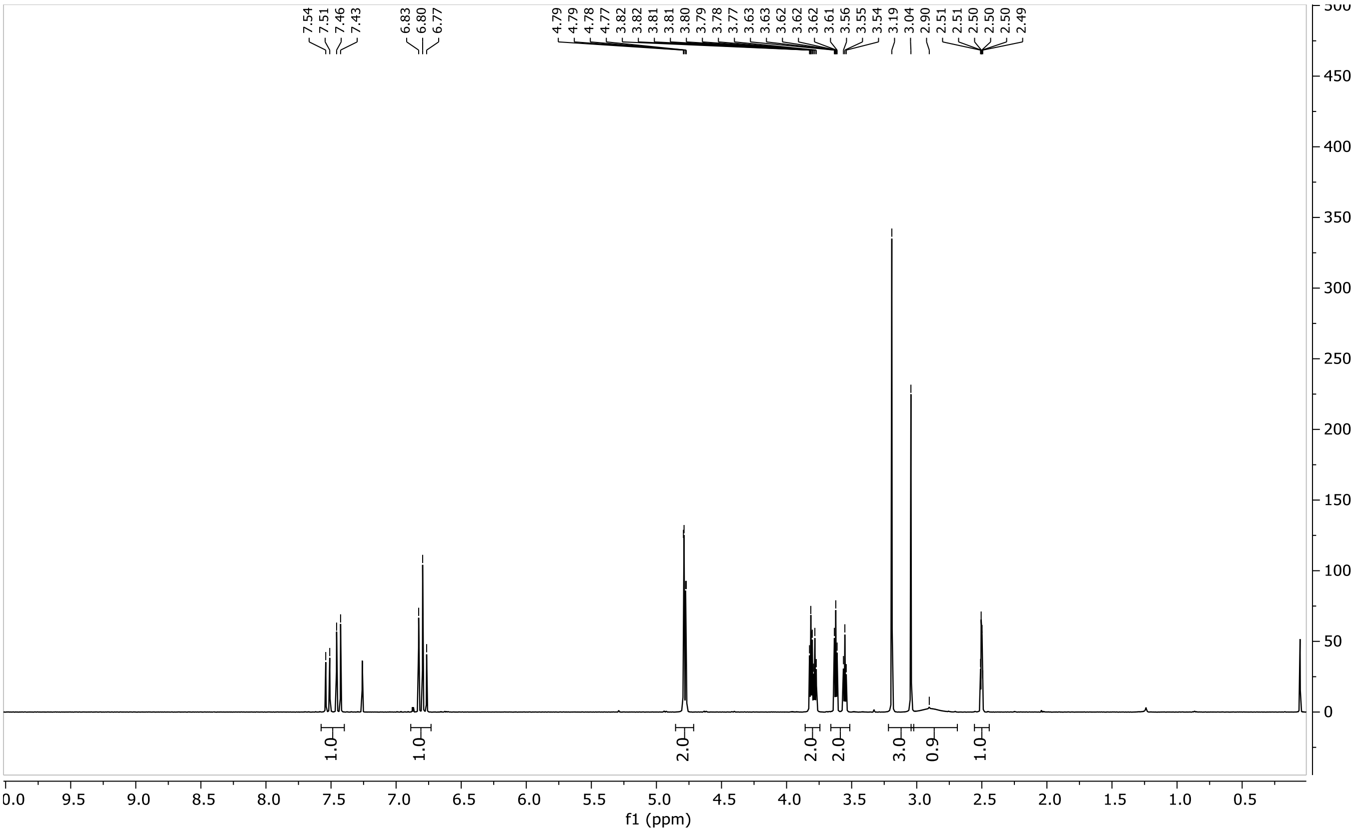

^{13}C NMR (126 MHz, $CDCl_3$)

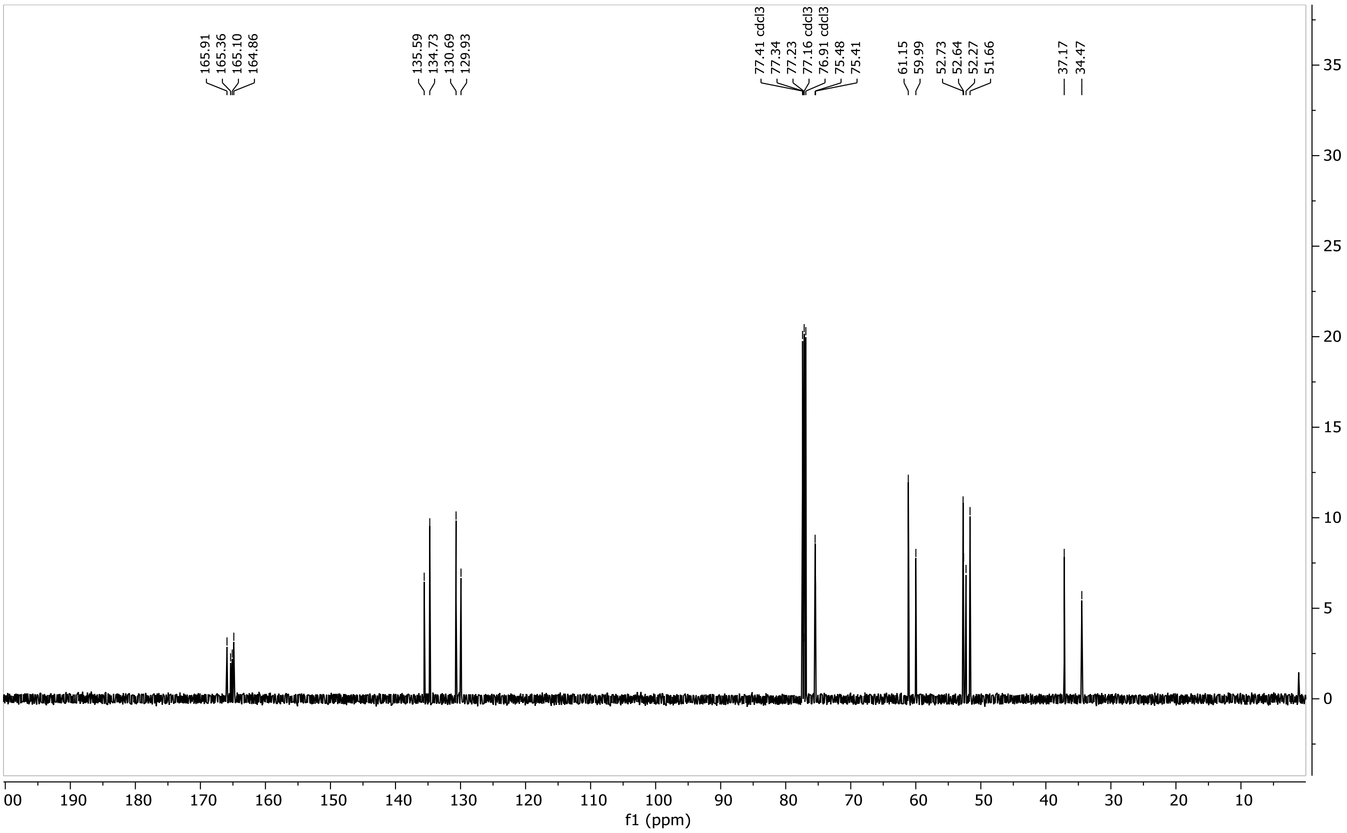

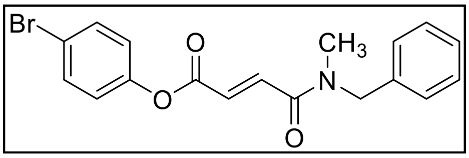

E-28

^{1}H NMR (500 MHz, $CDCl_3$)

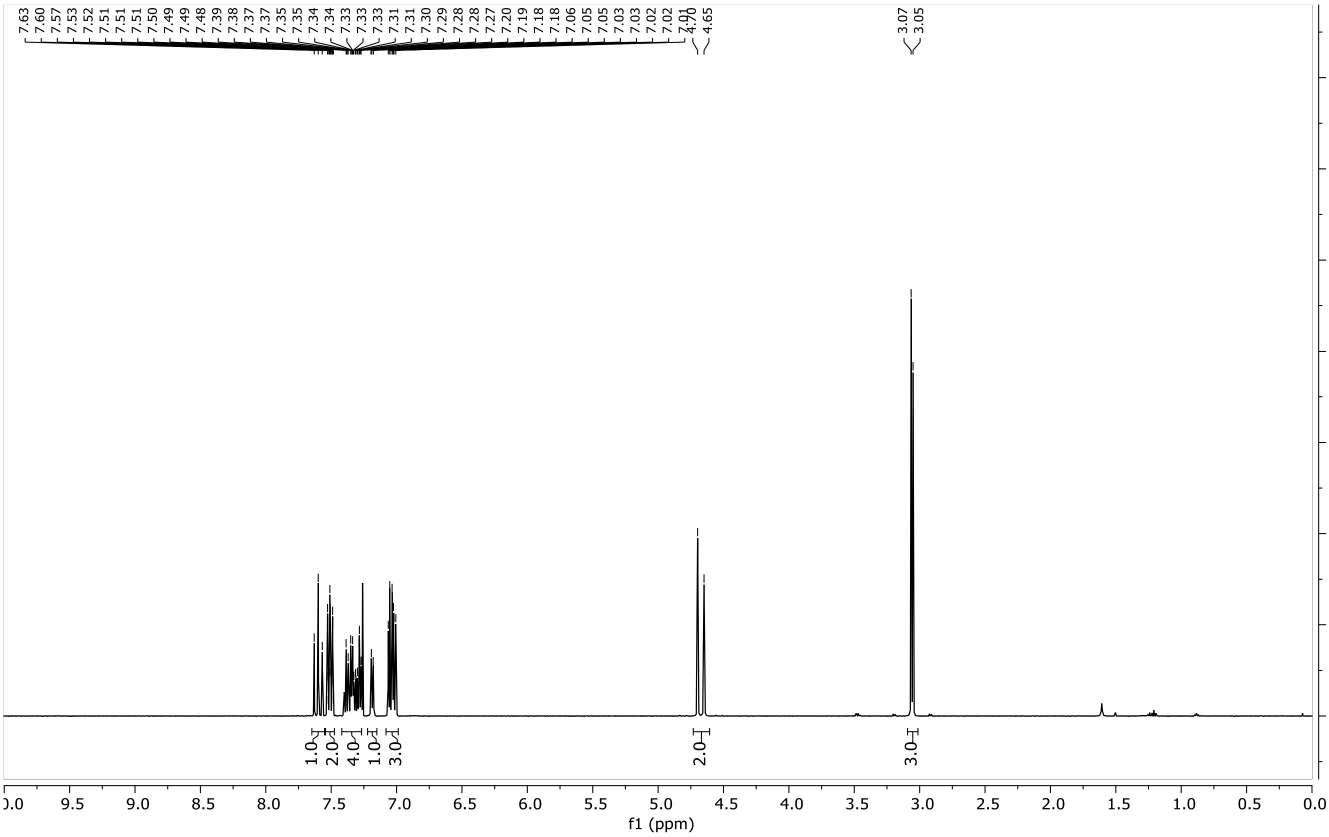

^{13}C NMR (126 MHz, $CDCl_3$)

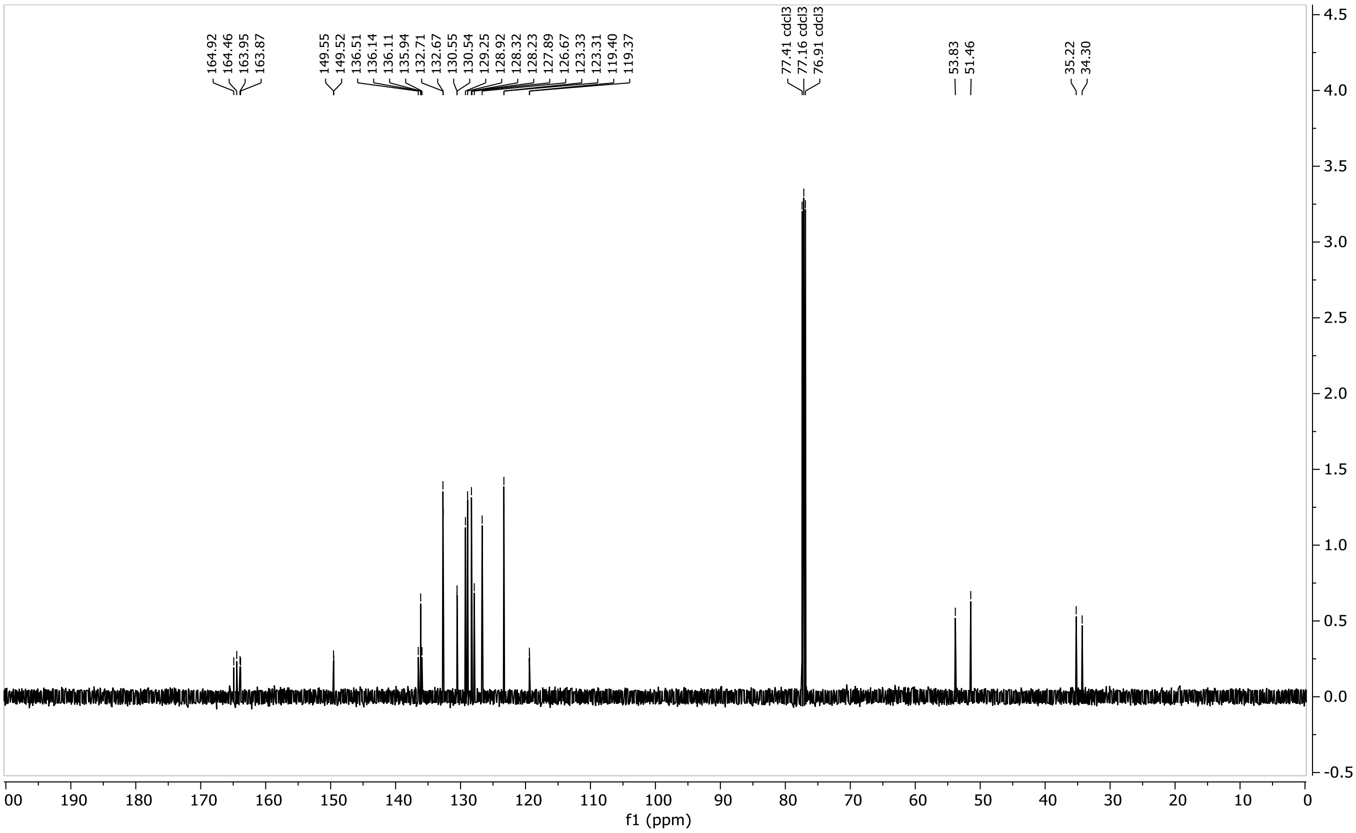

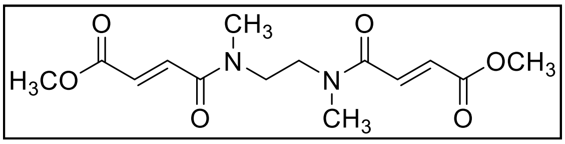

E-29

^{1}H NMR (500 MHz, DMSO-d$_6$)

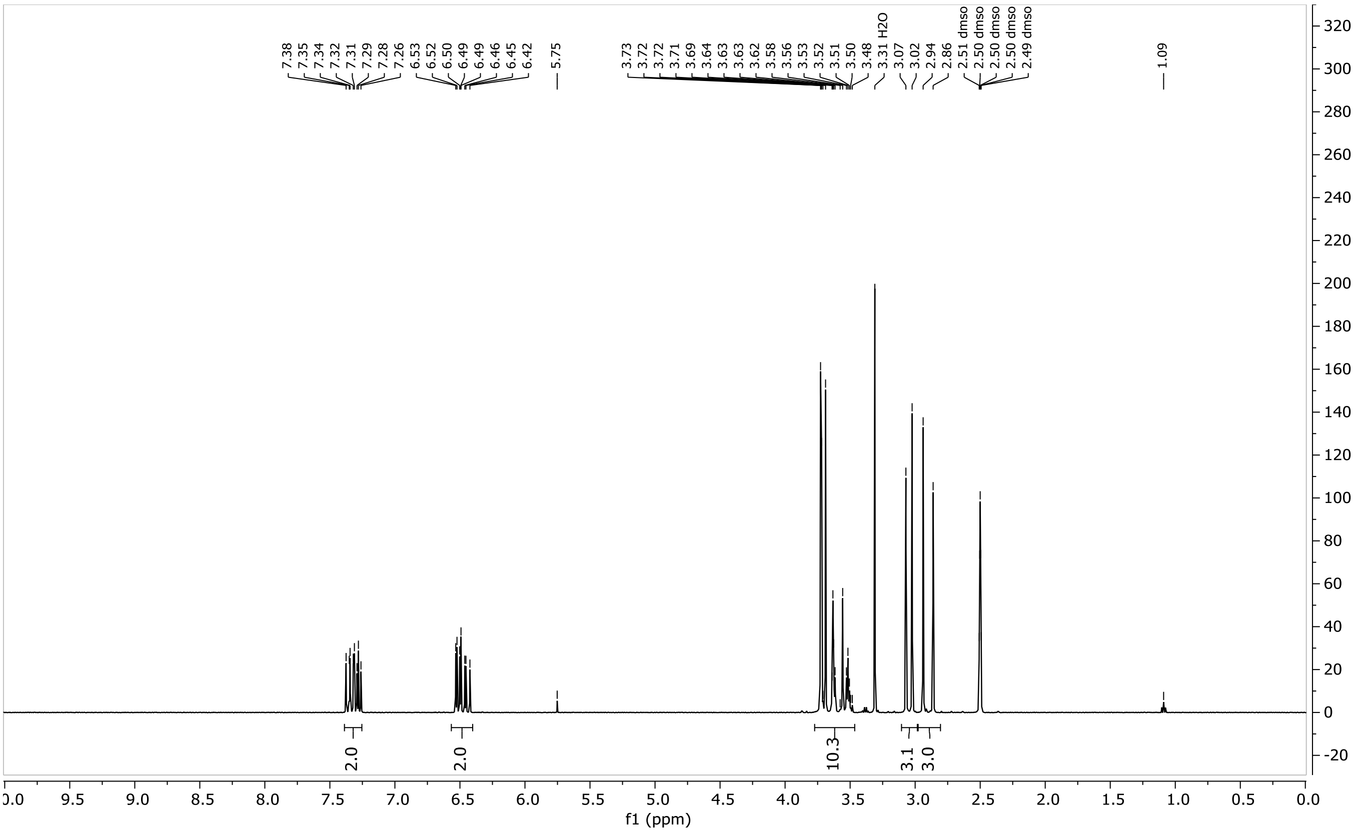

^{13}C NMR (126 MHz, DMSO-d$_6$)

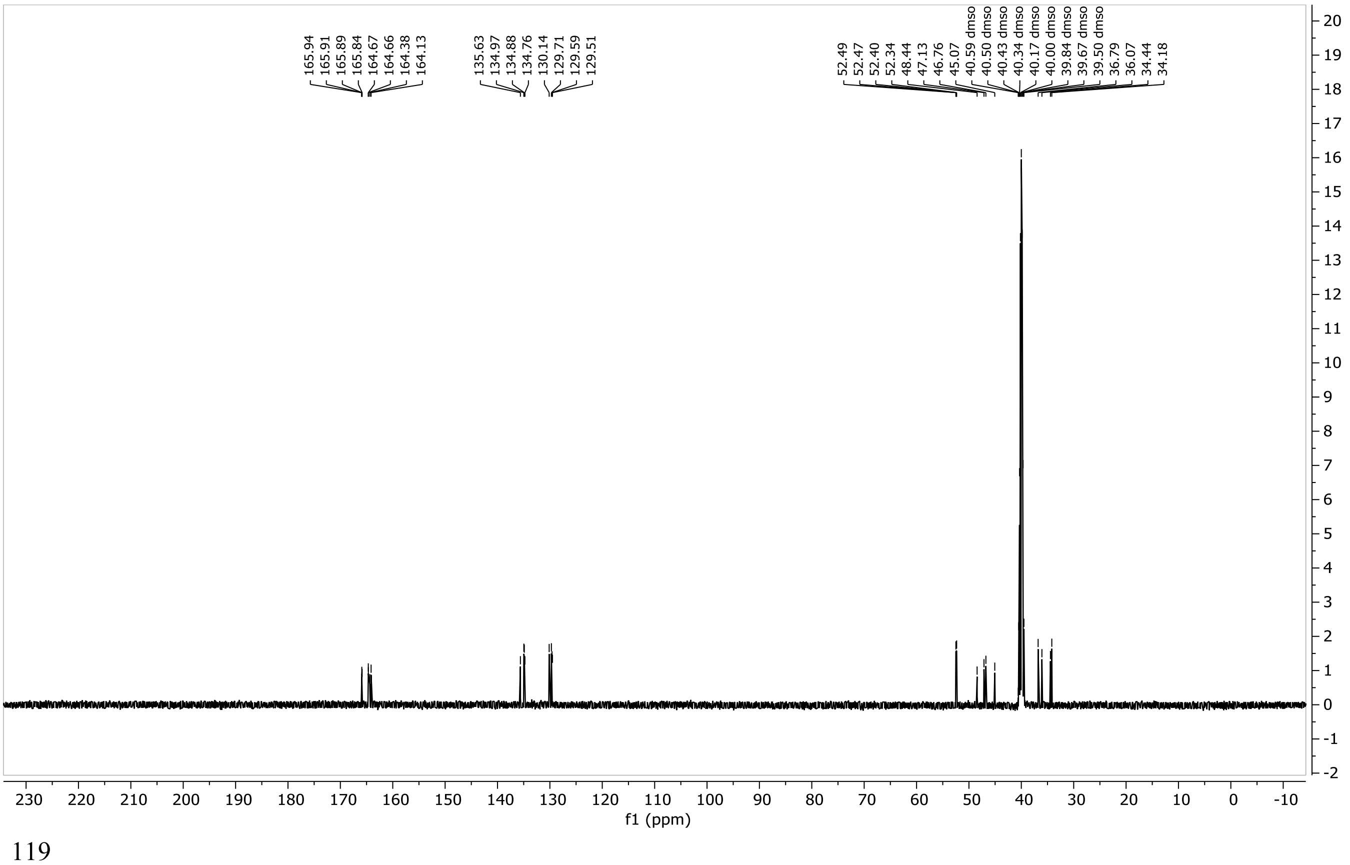

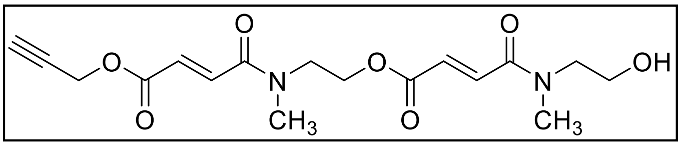

E-30

^{1}H NMR (600 MHz, $CDCl_3$)

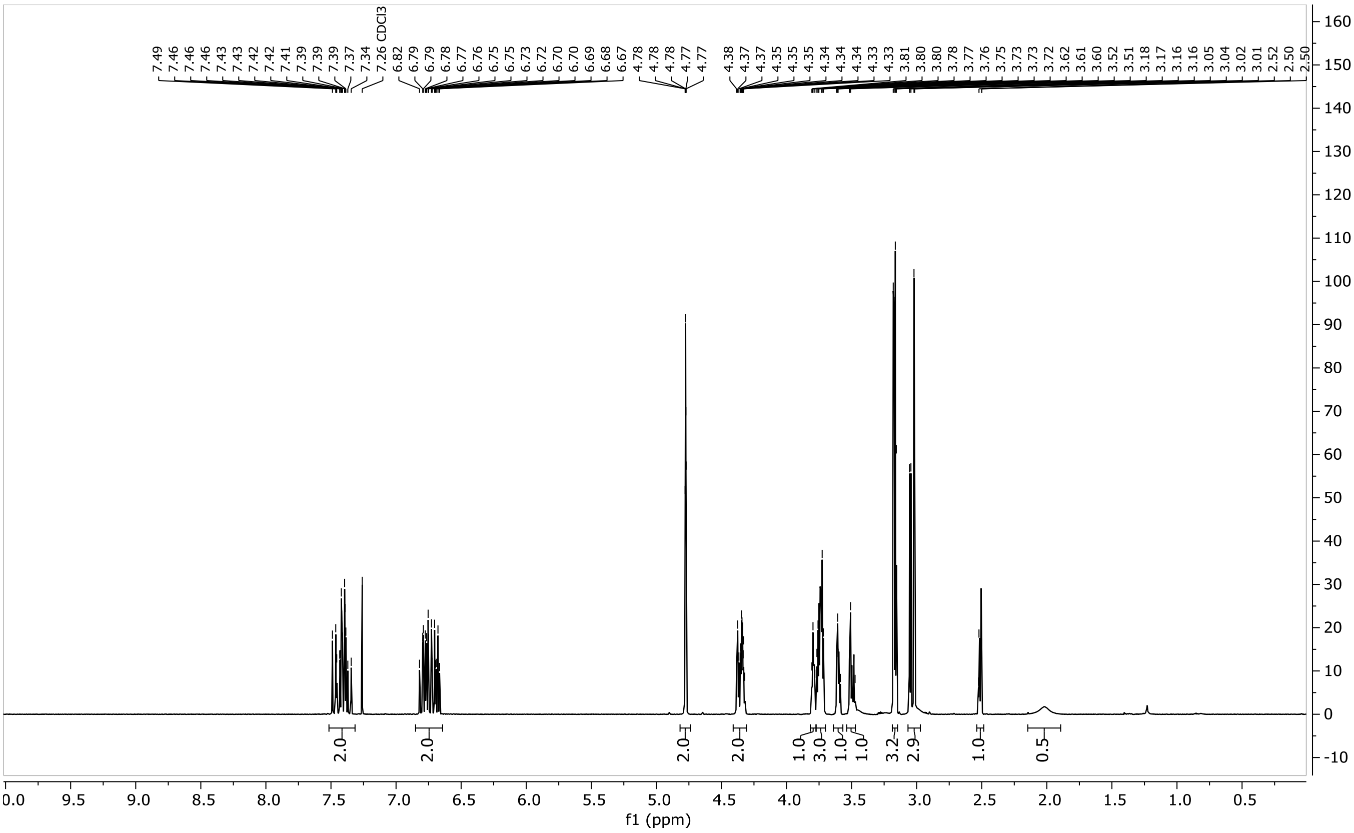

^{13}C NMR (151 MHz, $CDCl_3$)

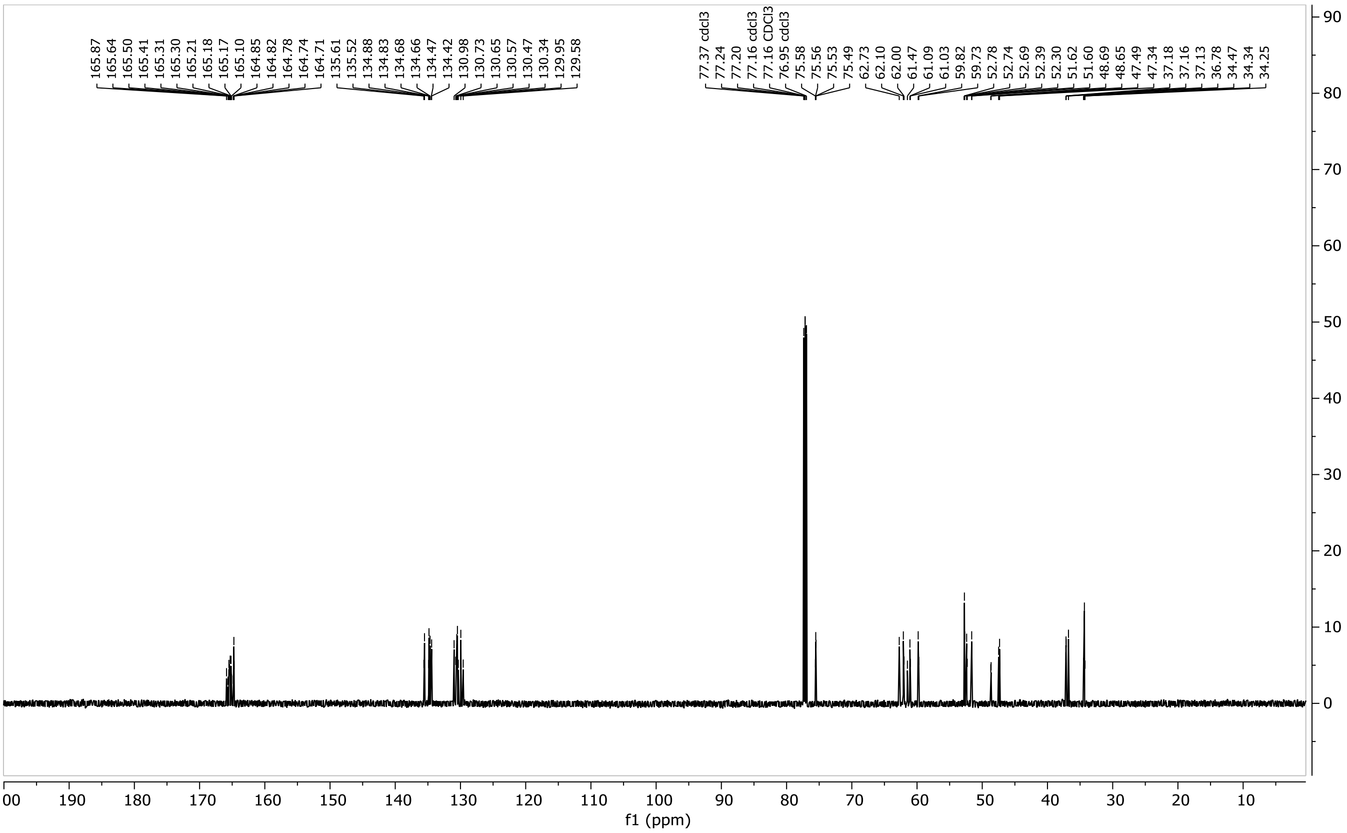

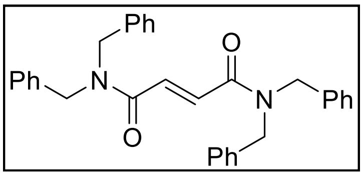

E-31

^{1}H NMR (600 MHz, $CDCl_3$)

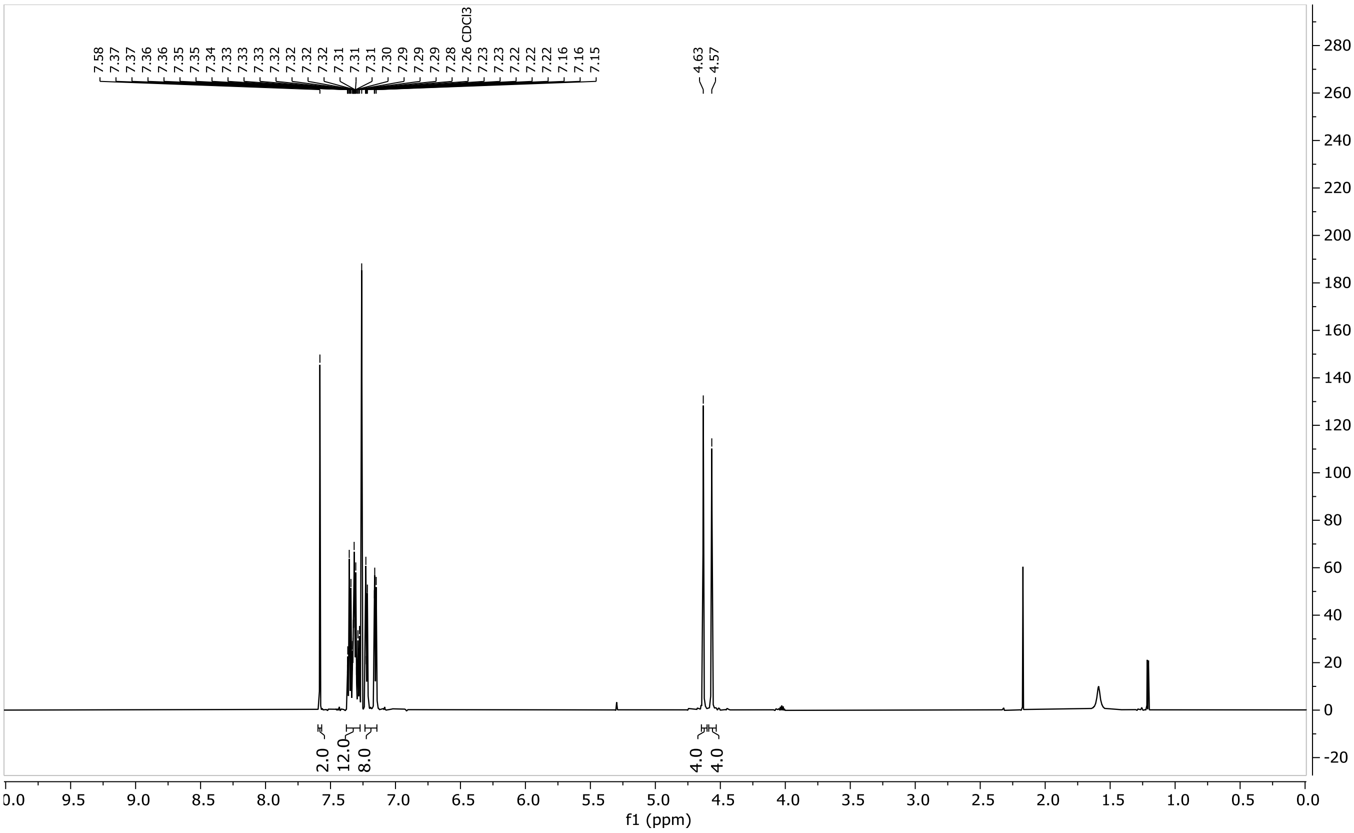

^{13}C NMR (151 MHz, $CDCl_3$)

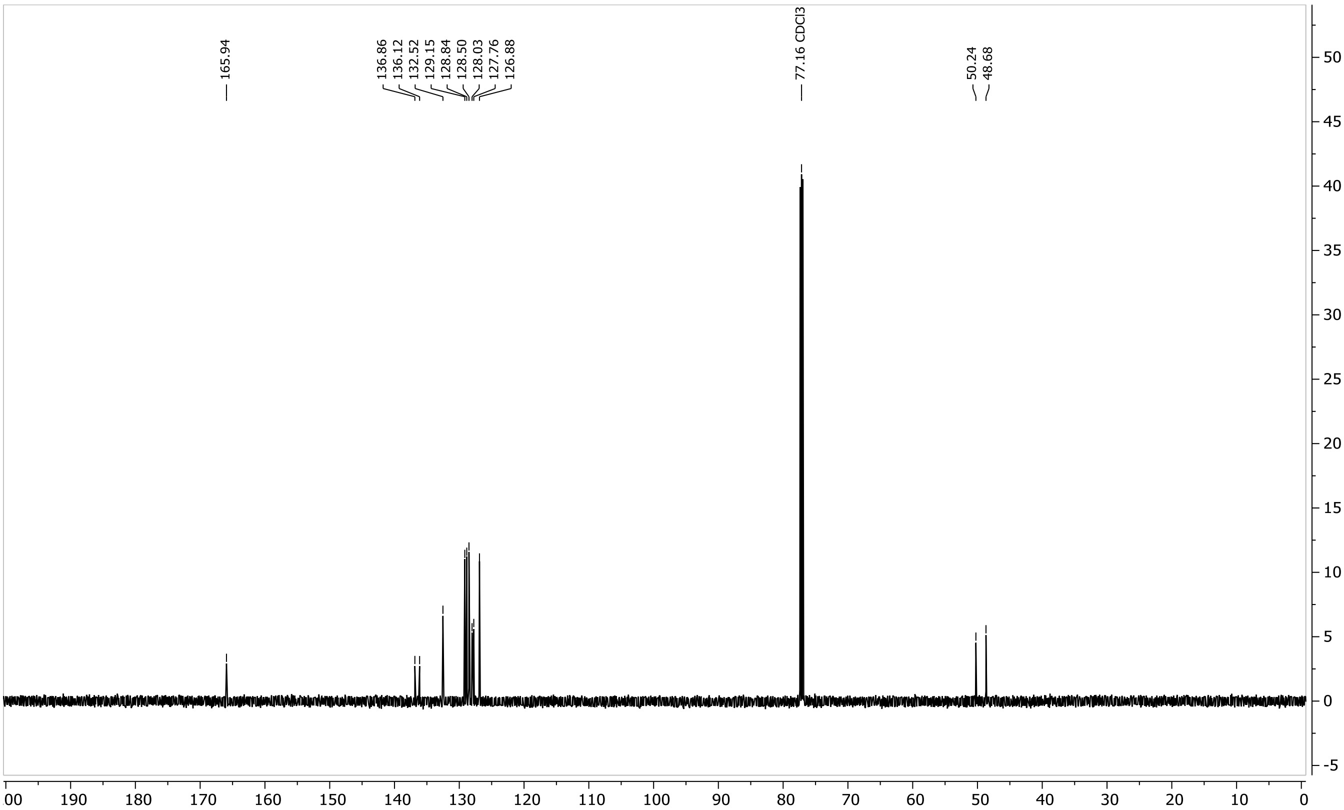

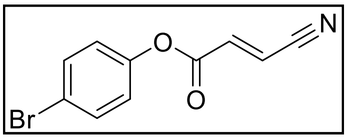

E-32

^1H NMR (400 MHz, $CDCl_3$)

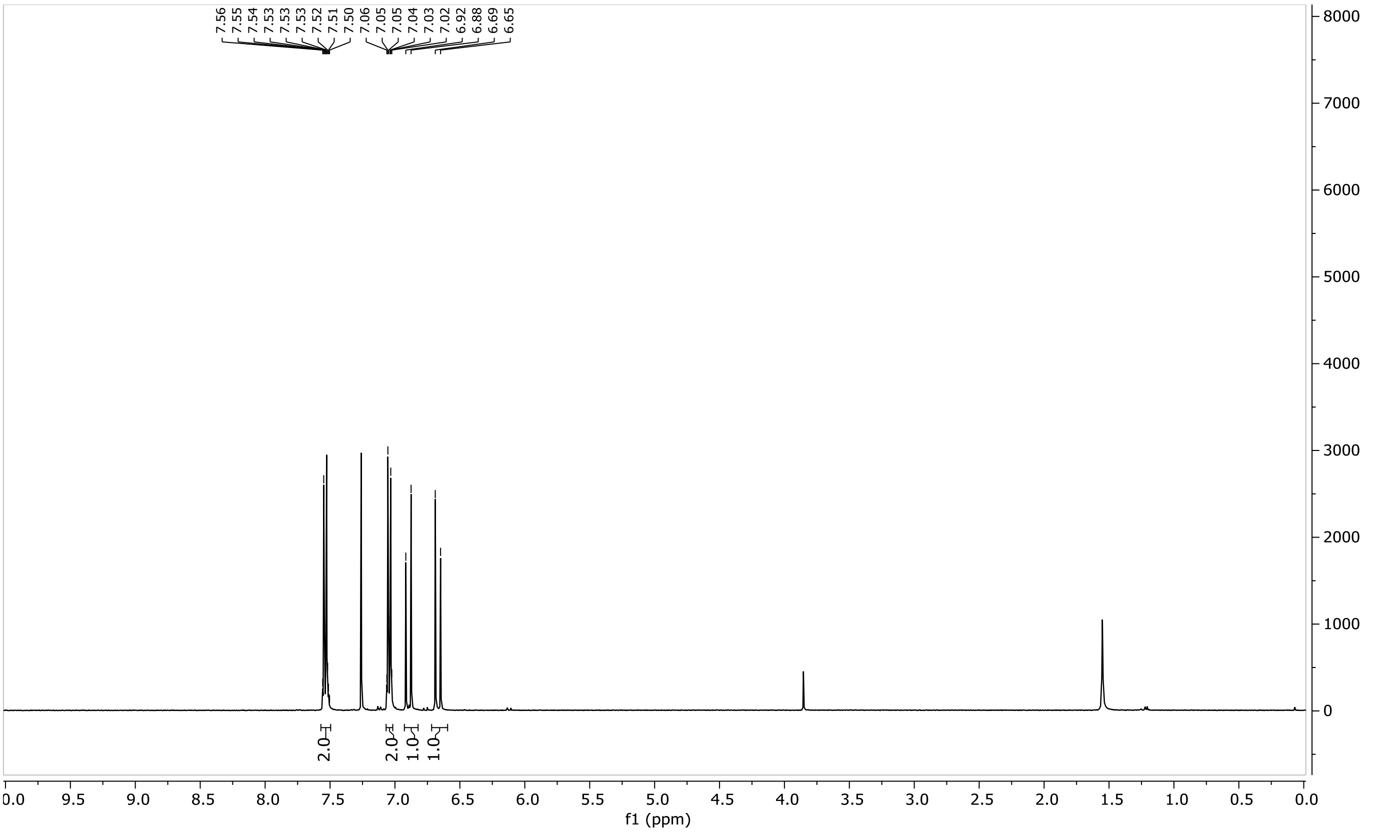

^{13}C NMR (100 MHz, $CDCl_3$)

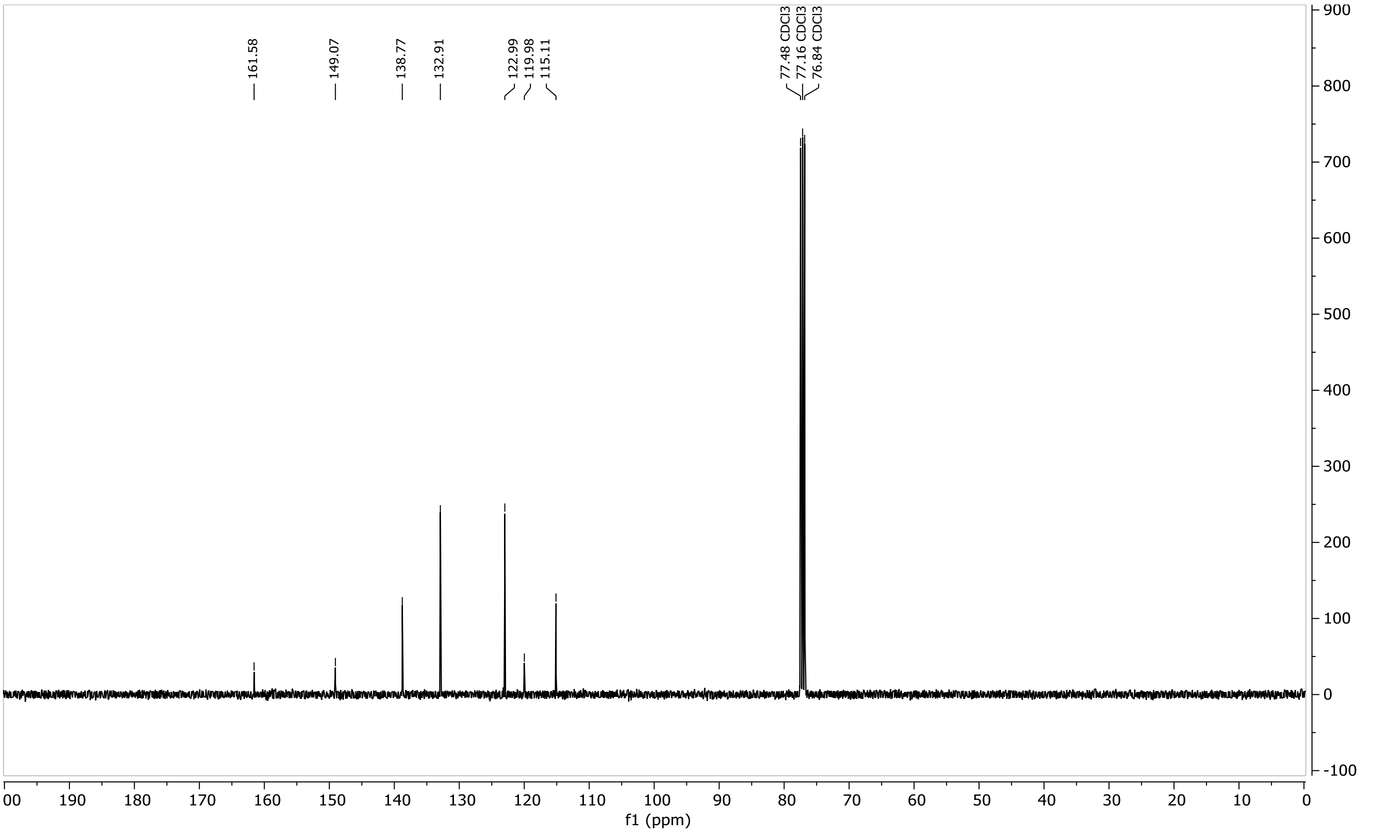

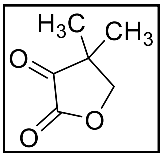

S-2

^{1}H NMR (400 MHz, $CDCl_3$)

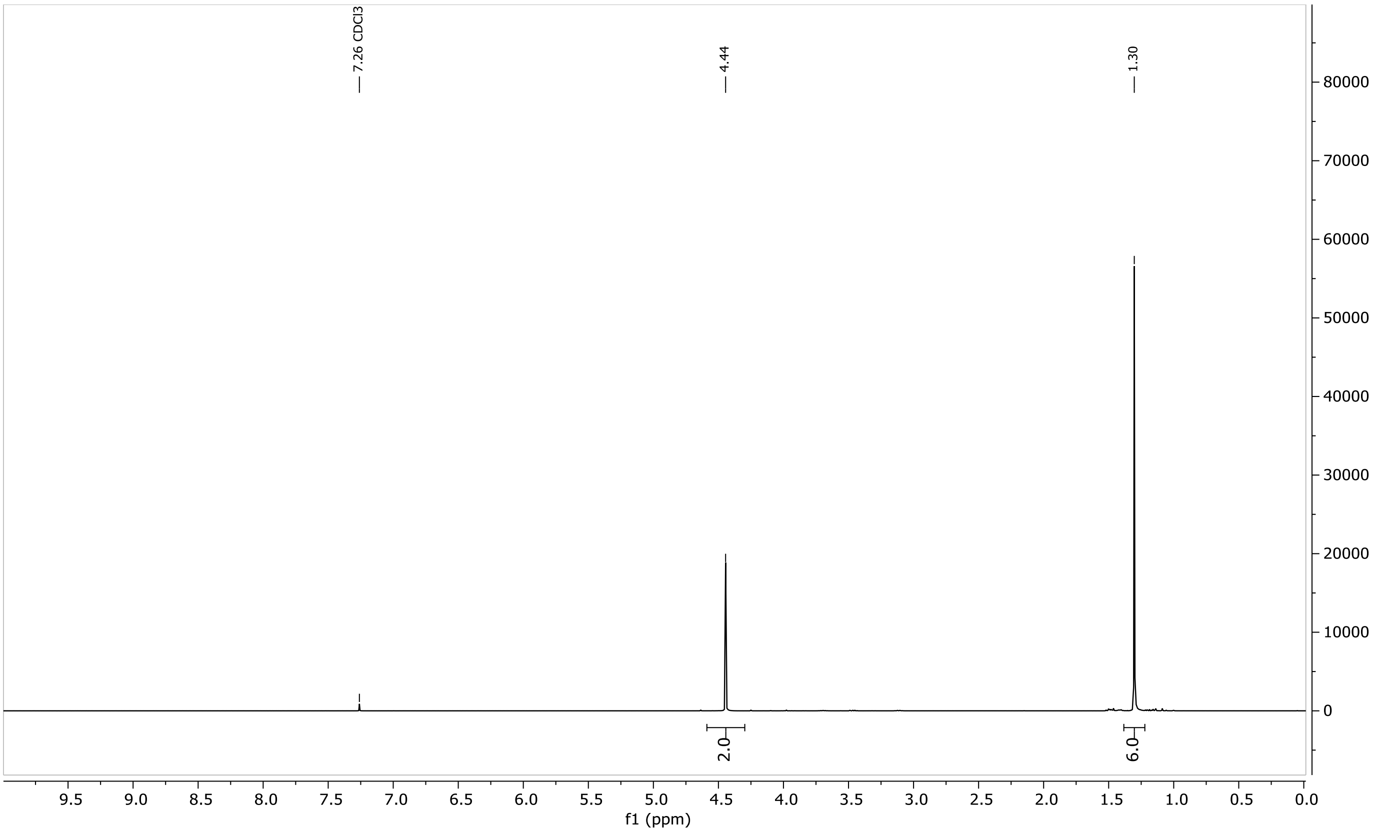

^{13}C NMR (101 MHz, $CDCl_3$)

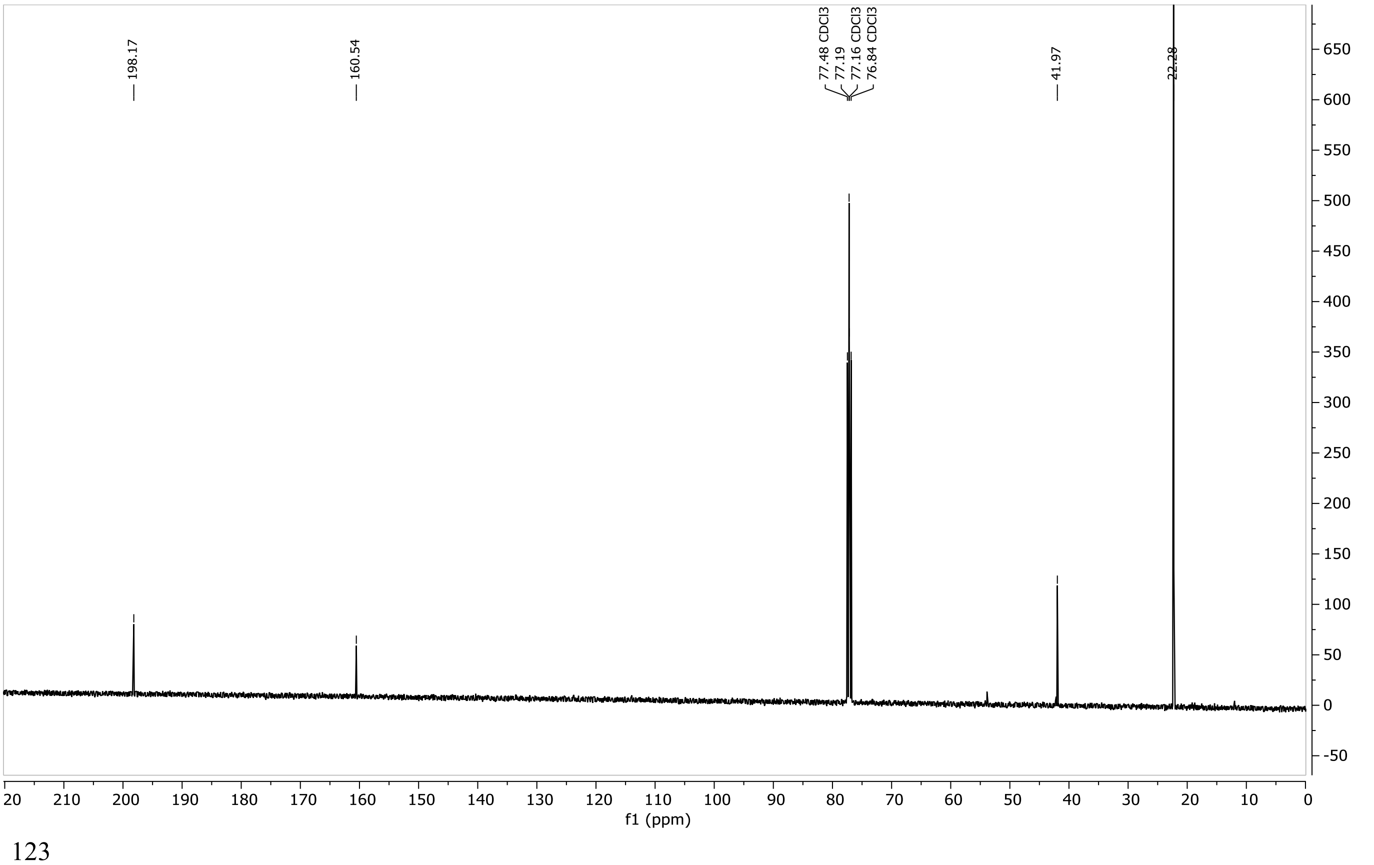

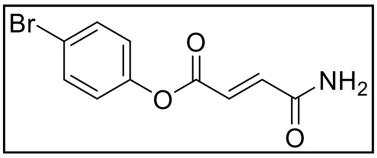

S-3

^{1}H NMR (600 MHz, DMSO-d$_6$)

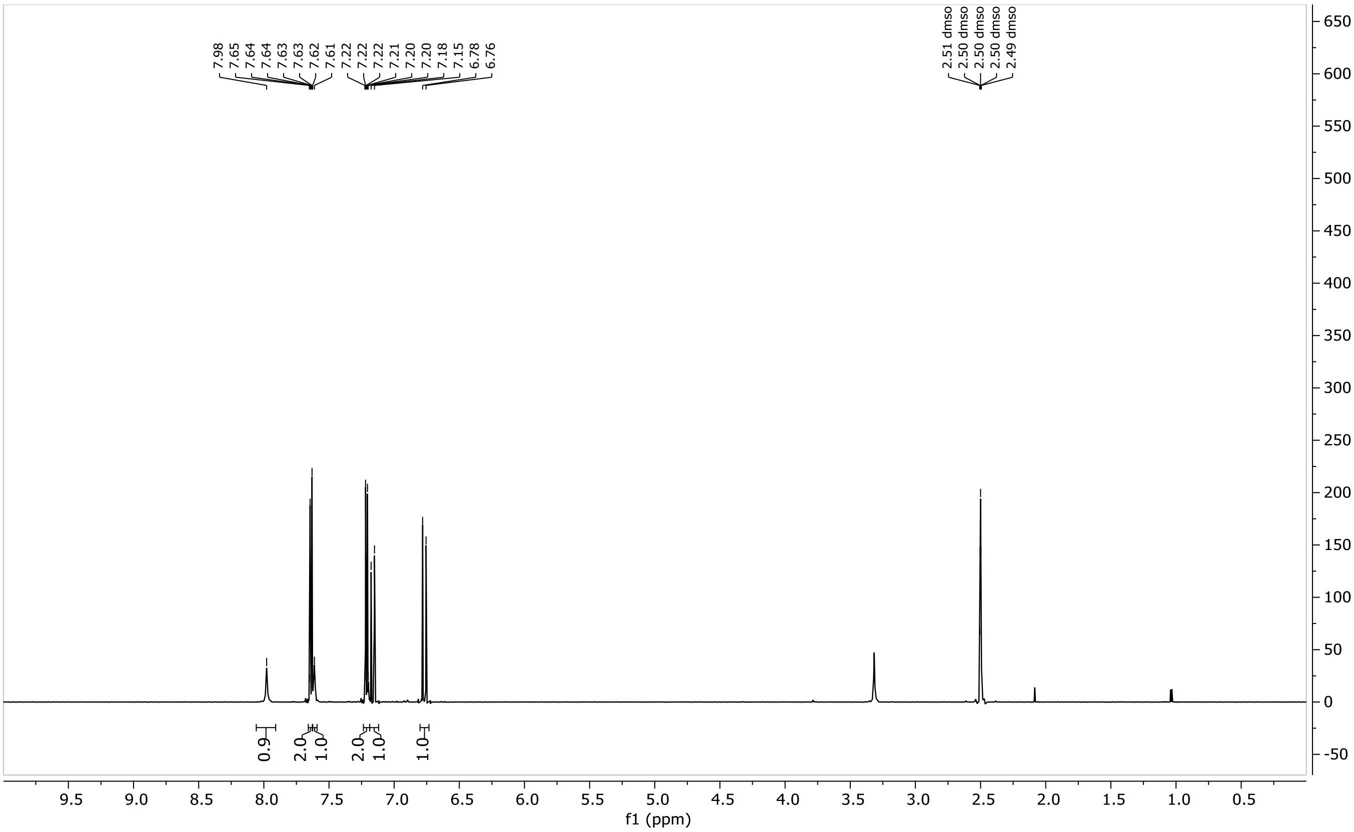

^{13}C NMR (151 MHz, DMSO-d$_6$)

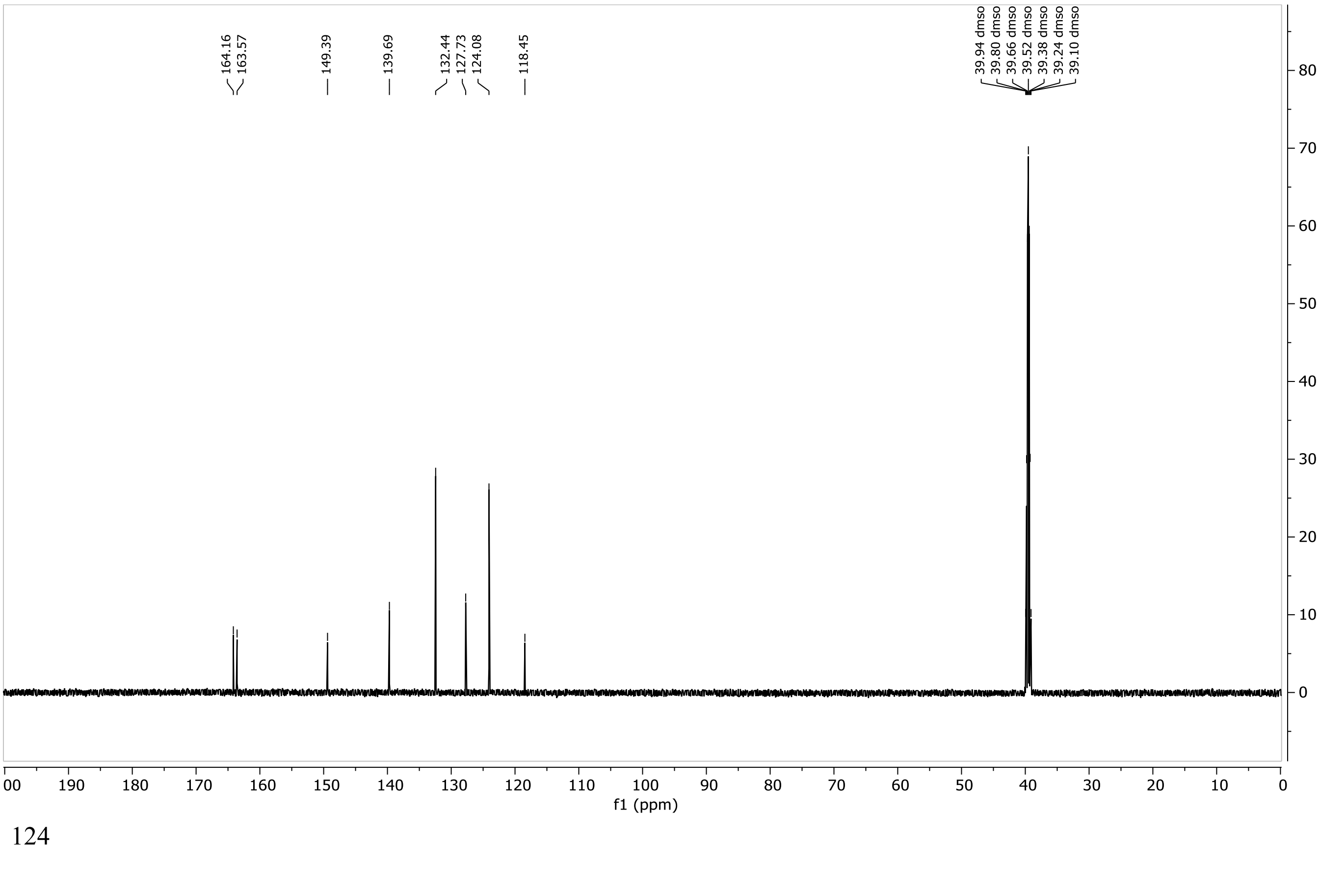

Supplement: Supplementary file 1 — Supporting Information [file ANIE-61-0-s001.pdf]
